# Supplementary material for: Tubulin Carboxypeptidase Activity Promotes Focal Gelatin Degradation in Breast Tumor Cells and Induces Apoptosis in Breast Epithelial Cells That Is Overcome by Oncogenic Signaling
Source: Cancers (Basel). 2022 Mar 28;14(7):1707. doi: 10.3390/cancers14071707 (PMC8996877; doi:10.3390/cancers14071707)
Supplement: Supplementary file 1 [file cancers-14-01707-s001.zip › cancers-1658953-Figure S8.pdf]

iBright™ Image Analysis Report

28 January 2022

Figure1B- deTyr-Tub SHORT Exposure

CHEMI_07062021_160135_280ms_10A_TCP_TIME_0_16_24_48_PUCK_DETYR

Date:	07-06-2021 04:01:35PM
Mode:	Chemi Blots
Notes:	
Model:	FL1500
Instrument name:	2462619090234
Serial No:	2462619090234
Firmware version:	1.6.0
iBA version:	4.0.1
Image size:	451px X 360px
Image area:	
Optical Zoom:	2x
Digital Zoom:	1.5x
Focus level:	455
Resolution:	5 x 5
Exposure time:	280 ms
Exposure mode:	Normal

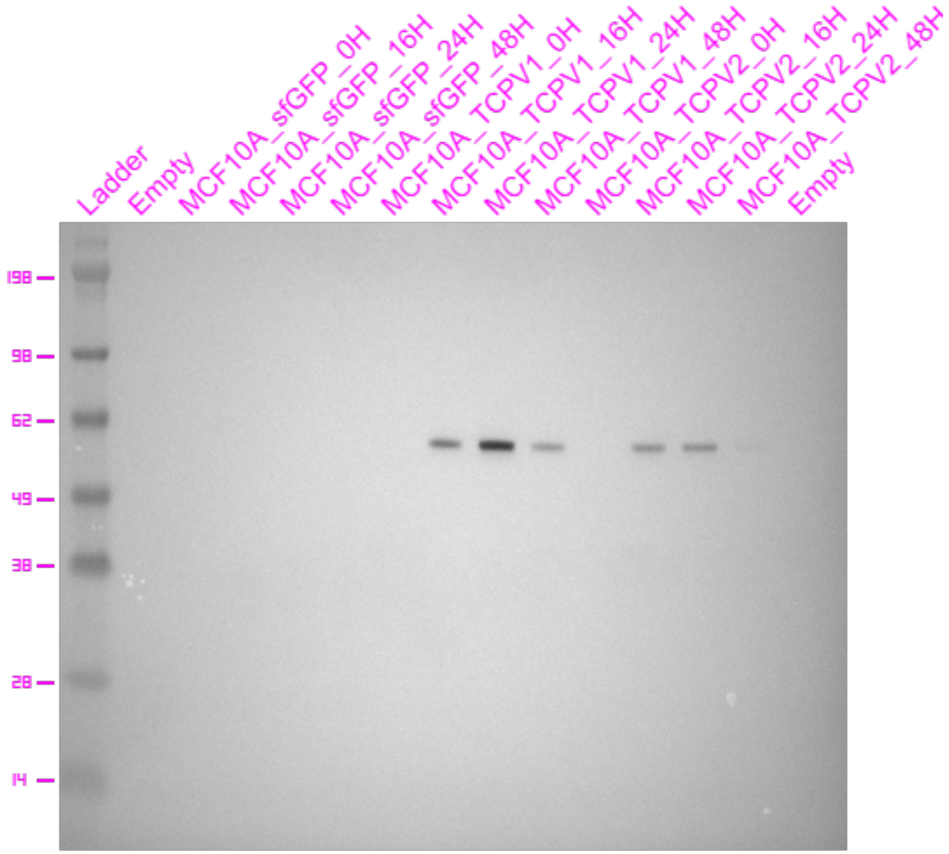

CHEMI_07062021_160135_280ms_10A_TCP _TIME_0_16_24_48_PUCK_DETYR

Date:	07-06-2021 04:01:35PM
Mode:	Chemi Blots
Notes:	
Model:	FL1500
Instrument name:	2462619090234
Serial No:	2462619090234
Firmware version:	1.6.0
iBA version:	4.0.1
Image size:	451px X 360px
Image area:	
Optical Zoom:	2x
Digital Zoom:	1.5x
Focus level:	455
Resolution:	5 x 5
Exposure time:	280 ms
Exposure mode:	Normal

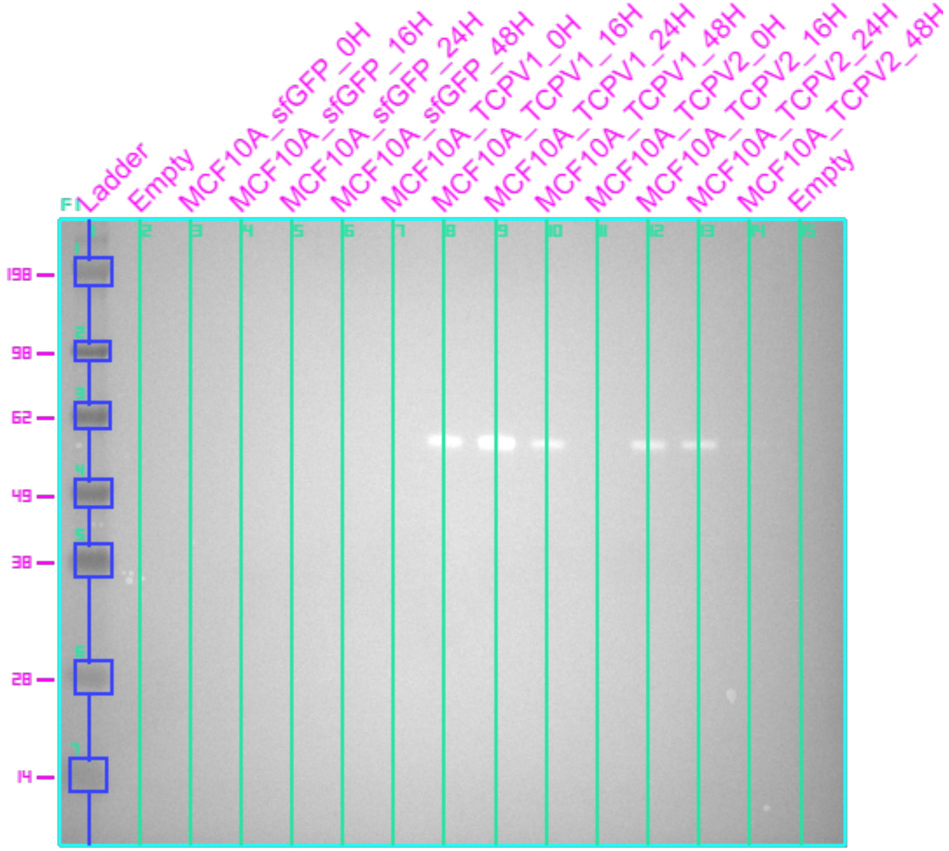

CHEMI_07062021_160135_280ms_10A_TCP_TIME_0_16_24_48_PUCK_DETYR

Date:	07-06-2021 04:01:35PM
Mode:	Chemi Blots
Notes:	
Model:	FL1500
Instrument name:	2462619090234
Serial No:	2462619090234
Firmware version:	1.6.0
iBA version:	4.0.1
Image size:	451px X 360px
Image area:	
Optical Zoom:	2x
Digital Zoom:	1.5x
Focus level:	455
Resolution:	5 x 5
Exposure time:	280 ms
Exposure mode:	Normal

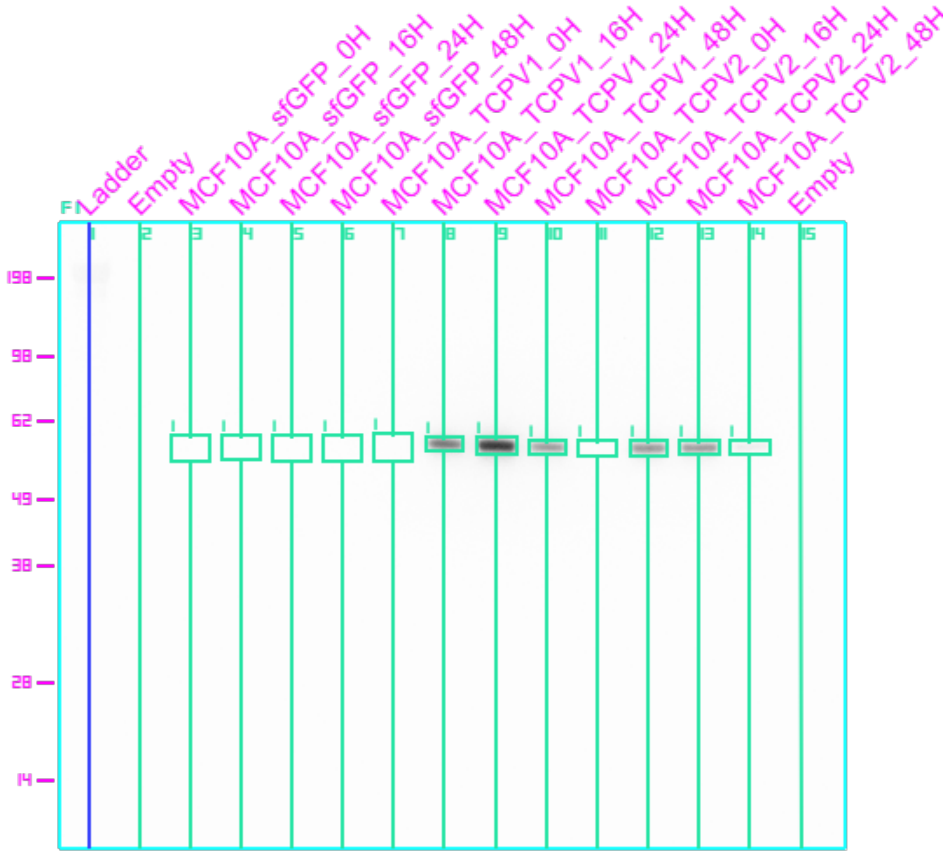

LANE AND BAND ANALYSIS DATA TABLE

CHEMI_07062021_160135_280ms_10A_TCP_TIME_0_16_24_48_PUCK_DETYR

Frame: 1
Channel: Membrane
Sensitivity: 100
Molecular Weight Analysis Regression Method : Point to Point

Lane 1 - Ladder

#	Vol. (Int.)	Local Bg. Corr. Vol.	Area	Rf	Density	Local Bg. Corr. Den.	% band purity	% lane purity	Mol. Wt.
1	12,368,433	1,276,168	374	0.084	33,070	3,412.214	11.363	5.812	198
2	8,743,892	1,504,983	252	0.209	34,697	5,972.156	13.4	4.109	98
3	11,606,737	2,029,947	336	0.312	34,543	6,041.512	18.074	5.454	62
4	12,741,139	2,144,339	374	0.437	34,067	5,733.527	19.093	5.987	49
5	15,005,169	2,481,089	440	0.543	34,102	5,638.841	22.091	7.051	38
6	13,853,325	1,009,985	440	0.73	31,484	2,295.423	8.993	6.509	28
7	14,446,320	784,758	440	0.886	32,832	1,783.542	6.987	6.788	14

Frame: 1
Channel: Chemi
Sensitivity: 100
Molecular Weight Analysis Regression Method : Point to Point

Lane 3 - MCF10A_sfGFP_0H

#	Vol. (Int.)	Local Bg. Corr. Vol.	Area	Rf	Density	Local Bg. Corr. Den.	% band purity	% lane purity	Mol. Wt.
1	24,577	13,677	368	0.359	66.785	37.168	100	8.247	57.089

Lane 4 - MCF10A_sfGFP_16H

#	Vol. (Int.)	Local Bg. Corr. Vol.	Area	Rf	Density	Local Bg. Corr. Den.	% band purity	% lane purity	Mol. Wt.
1	22,320	10,162	345	0.359	64.696	29.457	100	7.214	57.089

Lane 5 - MCF10A_sfGFP_24H

#	Vol. (Int.)	Local Bg. Corr. Vol.	Area	Rf	Density	Local Bg. Corr. Den.	% band purity	% lane purity	Mol. Wt.
1	33,325	14,192	368	0.359	90.557	38.566	100	9.418	57.089

Lane 6 - MCF10A_sfGFP_48H

#	Vol. (Int.)	Local Bg. Corr. Vol.	Area	Rf	Density	Local Bg. Corr. Den.	% band purity	% lane purity	Mol. Wt.
1	51,993	17,039	368	0.359	141.285	46.302	100	12.093	57.089

Lane 7 - MCF10A_TCPV1_0H

#	Vol. (Int.)	Local Bg. Corr. Vol.	Area	Rf	Density	Local Bg. Corr. Den.	% band purity	% lane purity	Mol. Wt.
1	130,379	12,087	391	0.359	333.45	30.914	100	18.577	57.089

Lane 8 - MCF10A_TCPV1_16H

#	Vol. (Int.)	Local Bg. Corr. Vol.	Area	Rf	Density	Local Bg. Corr. Den.	% band purity	% lane purity	Mol. Wt.
1	3,427,322	2,920,619	198	0.354	17,309	14,750	100	69.033	57.667

Lane 9 - MCF10A_TCPV1_24H

#	Vol. (Int.)	Local Bg. Corr. Vol.	Area	Rf	Density	Local Bg. Corr. Den.	% band purity	% lane purity	Mol. Wt.
1	6,682,183	5,796,772	264	0.357	25,311	21,957	100	79.769	57.378

Lane 10 - MCF10A_TCPV1_48H

#	Vol. (Int.)	Local Bg. Corr. Vol.	Area	Rf	Density	Local Bg. Corr. Den.	% band purity	% lane purity	Mol. Wt.
1	2,360,055	1,979,731	198	0.359	11,919	9,998.644	100	62.635	57.089

Lane 11 - MCF10A_TCPV2_0H

#	Vol. (Int.)	Local Bg. Corr. Vol.	Area	Rf	Density	Local Bg. Corr. Den.	% band purity	% lane purity	Mol. Wt.
1	123,584	1,593.184	230	0.359	537.322	6.927	100	12.805	57.089

Lane 12 - MCF10A_TCPV2_16H

#	Vol. (Int.)	Local Bg. Corr. Vol.	Area	Rf	Density	Local Bg. Corr. Den.	% band purity	% lane purity	Mol. Wt.
1	2,654,027	2,296,963	220	0.359	12,063	10,440	100	71.273	57.089

Lane 13 - MCF10A_TCPV2_24H

#	Vol. (Int.)	Local Bg. Corr. Vol.	Area	Rf	Density	Local Bg. Corr. Den.	% band purity	% lane purity	Mol. Wt.
1	2,528,199	2,198,746	216	0.359	11,704	10,179	100	72.82	57.089

Lane 14 - MCF10A_TCPV2_48H

#	Vol. (Int.)	Local Bg. Corr. Vol.	Area	Rf	Density	Local Bg. Corr. Den.	% band purity	% lane purity	Mol. Wt.
1	388,972	284,919	216	0.359	1,800.796	1,319.071	100	40.789	57.089

iBright™ Image Analysis Report

28 January 2022

Figure1B- deTyr-Tub LONG Exposure

CHEMI_07062021_160256_10s_10A_TCP_TIME_0_16_24_48_PUCK_DETYR

Date:	07-06-2021 04:02:56PM
Mode:	Chemi Blots
Notes:	
Model:	FL1500
Instrument name:	2462619090234
Serial No:	2462619090234
Firmware version:	1.6.0
iBA version:	4.0.1
Image size:	451px X 360px
Image area:	
Optical Zoom:	2x
Digital Zoom:	1.5x
Focus level:	455
Resolution:	5 x 5
Exposure time:	10000 ms
Exposure mode:	Normal

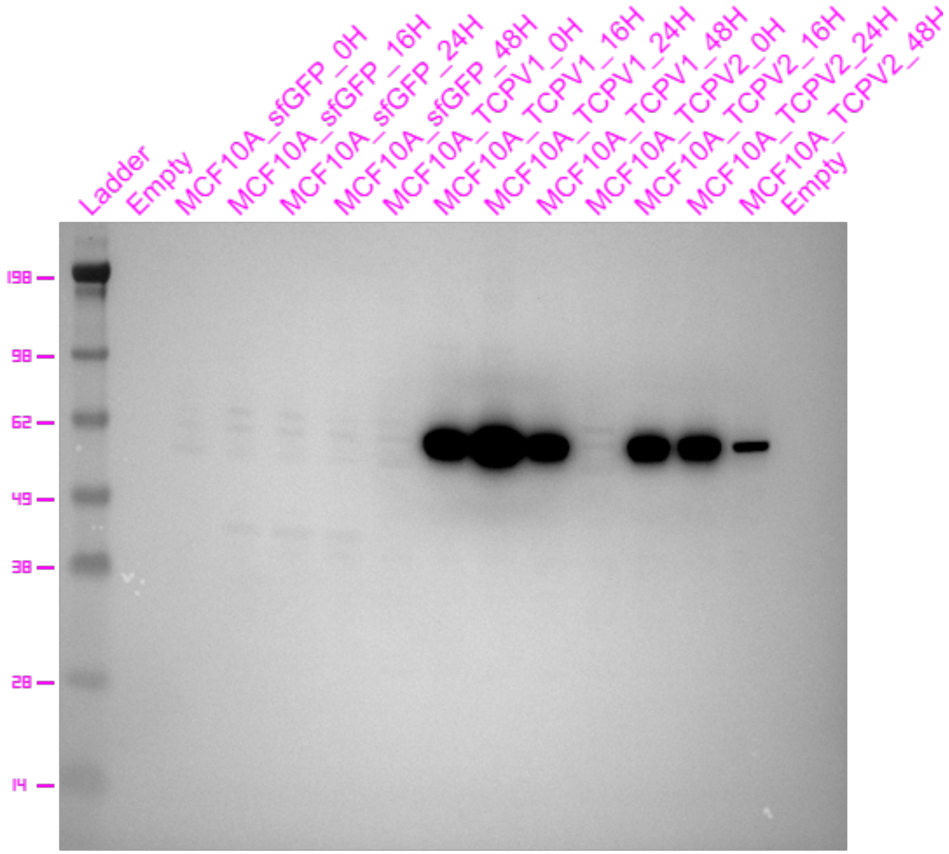

CHEMI_07062021_160256_10s_10A_TCP_TIME_0_16_24_48_PUCK_DETYR

Date:	07-06-2021 04:02:56PM
Mode:	Chemi Blots
Notes:	
Model:	FL1500
Instrument name:	2462619090234
Serial No:	2462619090234
Firmware version:	1.6.0
iBA version:	4.0.1
Image size:	451px X 360px
Image area:	
Optical Zoom:	2x
Digital Zoom:	1.5x
Focus level:	455
Resolution:	5 x 5
Exposure time:	10000 ms
Exposure mode:	Normal

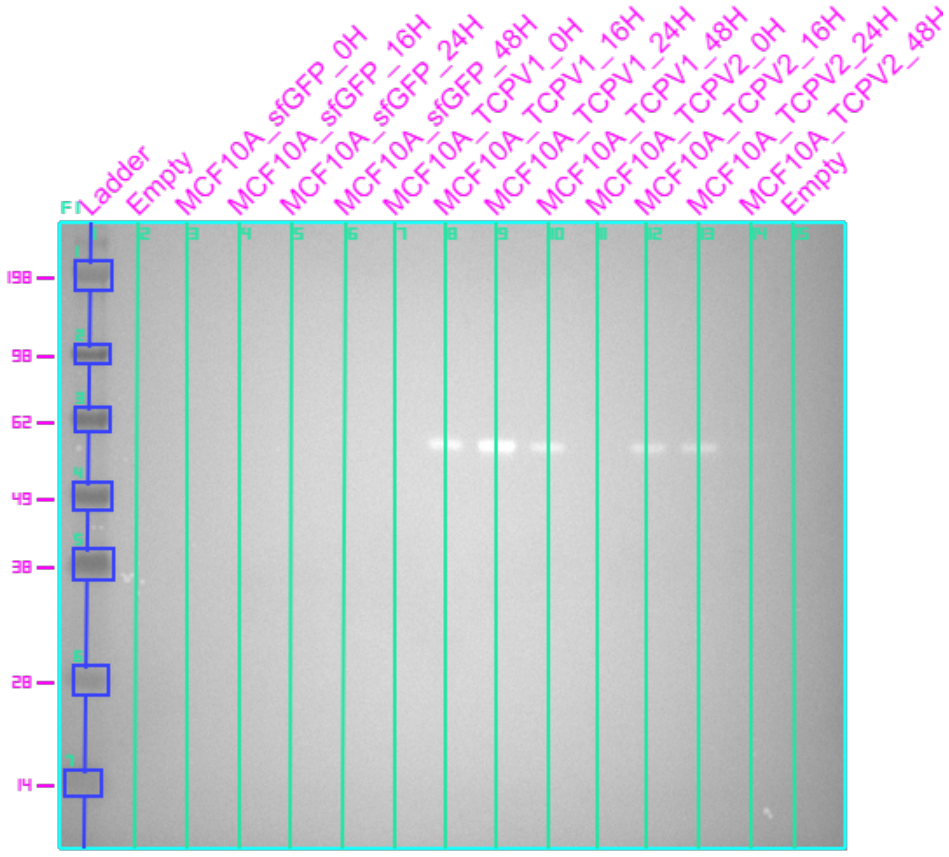

CHEMI_07062021_160256_10s_10A_TCP_TIME_0_16_24_48_PUCK_DETYR

Date:	07-06-2021 04:02:56PM
Mode:	Chemi Blots
Notes:	
Model:	FL1500
Instrument name:	2462619090234
Serial No:	2462619090234
Firmware version:	1.6.0
iBA version:	4.0.1
Image size:	451px X 360px
Image area:	
Optical Zoom:	2x
Digital Zoom:	1.5x
Focus level:	455
Resolution:	5 x 5
Exposure time:	10000 ms
Exposure mode:	Normal

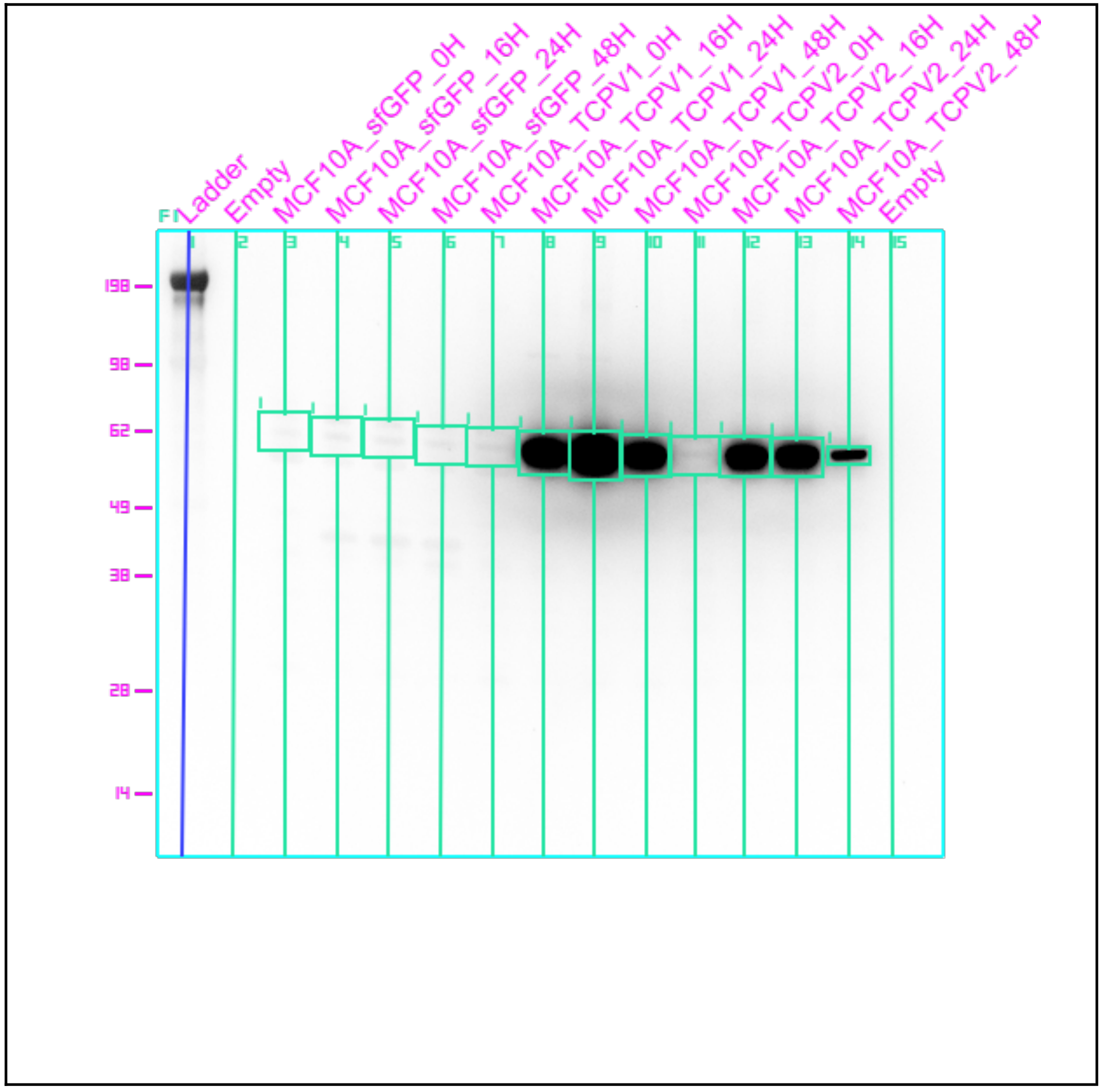

LANE AND BAND ANALYSIS DATA TABLE

CHEMI_07062021_160256_10s_10A_TCP_TIME_0_16_24_48_PUCK_DETYR

Frame: 1
Channel: Membrane
Sensitivity: 100
Molecular Weight Analysis Regression Method : Point to Point

Lane 1 - Ladder

#	Vol. (Int.)	Local Bg. Corr. Vol.	Area	Rf	Density	Local Bg. Corr. Den.	% band purity	% lane purity	Mol. Wt.
1	12,770,138	1,368,836	396	0.084	32,247	3,456.659	11.667	4.544	198
2	8,591,899	1,548,565	252	0.209	34,094	6,145.099	13.198	3.057	98
3	10,802,276	2,070,769	315	0.315	34,292	6,573.871	17.649	3.843	62
4	13,079,029	2,400,211	391	0.437	33,450	6,138.649	20.457	4.653	49
5	15,240,016	2,751,317	456	0.546	33,421	6,033.592	23.449	5.422	38
6	11,811,119	979,291	378	0.73	31,246	2,590.719	8.346	4.202	28
7	11,508,790	614,026	352	0.894	32,695	1,744.393	5.233	4.095	14

Frame: 1
Channel: Chemi
Sensitivity: 100
Molecular Weight Analysis Regression Method : Point to Point

Lane 3 - MCF10A_sfGFP_0H

#	Vol. (Int.)	Local Bg. Corr. Vol.	Area	Rf	Density	Local Bg. Corr. Den.	% band purity	% lane purity	Mol. Wt.
1	1,127,677	348,253	690	0.32	1,634.314	504.716	100	16.009	61.409

Lane 4 - MCF10A_sfGFP_16H

#	Vol. (Int.)	Local Bg. Corr. Vol.	Area	Rf	Density	Local Bg. Corr. Den.	% band purity	% lane purity	Mol. Wt.
1	1,477,245	529,278	690	0.329	2,140.935	767.07	100	18.094	60.523

Lane 5 - MCF10A_sfGFP_24H

#	Vol. (Int.)	Local Bg. Corr. Vol.	Area	Rf	Density	Local Bg. Corr. Den.	% band purity	% lane purity	Mol. Wt.
1	1,943,750	589,601	690	0.331	2,817.029	854.494	100	18.208	60.227

Lane 6 - MCF10A_sfGFP_48H

#	Vol. (Int.)	Local Bg. Corr. Vol.	Area	Rf	Density	Local Bg. Corr. Den.	% band purity	% lane purity	Mol. Wt.
1	3,098,967	511,767	690	0.343	4,491.257	741.692	100	19.855	59.045

Lane 7 - MCF10A_TCPV1_0H

#	Vol. (Int.)	Local Bg. Corr. Vol.	Area	Rf	Density	Local Bg. Corr. Den.	% band purity	% lane purity	Mol. Wt.
1	7,443,667	506,967	690	0.345	10,787	734.735	100	26.024	58.75

Lane 8 - MCF10A_TCPV1_16H

#	Vol. (Int.)	Local Bg. Corr. Vol.	Area	Rf	Density	Local Bg. Corr. Den.	% band purity	% lane purity	Mol. Wt.
1	41,617,611	20,150,844	780	0.354	53,355	25,834	100	56.886	57.864

Lane 9 - MCF10A_TCPV1_24H

#	Vol. (Int.)	Local Bg. Corr. Vol.	Area	Rf	Density	Local Bg. Corr. Den.	% band purity	% lane purity	Mol. Wt.
1	53,233,757	20,966,280	899	0.359	59,214	23,321	100	58.59	57.273

Lane 10 - MCF10A_TCPV1_48H

#	Vol. (Int.)	Local Bg. Corr. Vol.	Area	Rf	Density	Local Bg. Corr. Den.	% band purity	% lane purity	Mol. Wt.
1	38,699,613	17,329,184	750	0.359	51,599	23,105	100	52.553	57.273

Lane 11 - MCF10A_TCPV2_0H

#	Vol. (Int.)	Local Bg. Corr. Vol.	Area	Rf	Density	Local Bg. Corr. Den.	% band purity	% lane purity	Mol. Wt.
1	12,229,148	296,278	690	0.359	17,723	429.389	100	30.546	57.273

Lane 12 - MCF10A_TCPV2_16H

#	Vol. (Int.)	Local Bg. Corr. Vol.	Area	Rf	Density	Local Bg. Corr. Den.	% band purity	% lane purity	Mol. Wt.
1	35,382,345	19,729,763	744	0.359	47,556	26,518	100	59.29	57.273

Lane 13 - MCF10A_TCPV2_24H

#	Vol. (Int.)	Local Bg. Corr. Vol.	Area	Rf	Density	Local Bg. Corr. Den.	% band purity	% lane purity	Mol. Wt.
1	34,055,945	18,676,351	713	0.362	47,764	26,194	100	59.612	56.977

Lane 14 - MCF10A_TCPV2_48H

#	Vol. (Int.)	Local Bg. Corr. Vol.	Area	Rf	Density	Local Bg. Corr. Den.	% band purity	% lane purity	Mol. Wt.
1	10,850,690	7,181,401	275	0.359	39,457	26,114	100	34.481	57.273

iBright™ Image Analysis Report

28 January 2022

Figure 1B- Alpha Tubulin

CHEMI_07072021_143521_500ms_10A_TCP_TIME_0_16_24_48_ALPHA_TUB

Date:	07-07-2021 02:35:21PM
Mode:	Chemi Blots
Notes:	
Model:	FL1500
Instrument name:	2462619090234
Serial No:	2462619090234
Firmware version:	1.6.0
iBA version:	4.0.1
Image size:	451px X 360px
Image area:	
Optical Zoom:	2x
Digital Zoom:	1.5x
Focus level:	455
Resolution:	5 x 5
Exposure time:	500 ms
Exposure mode:	Normal

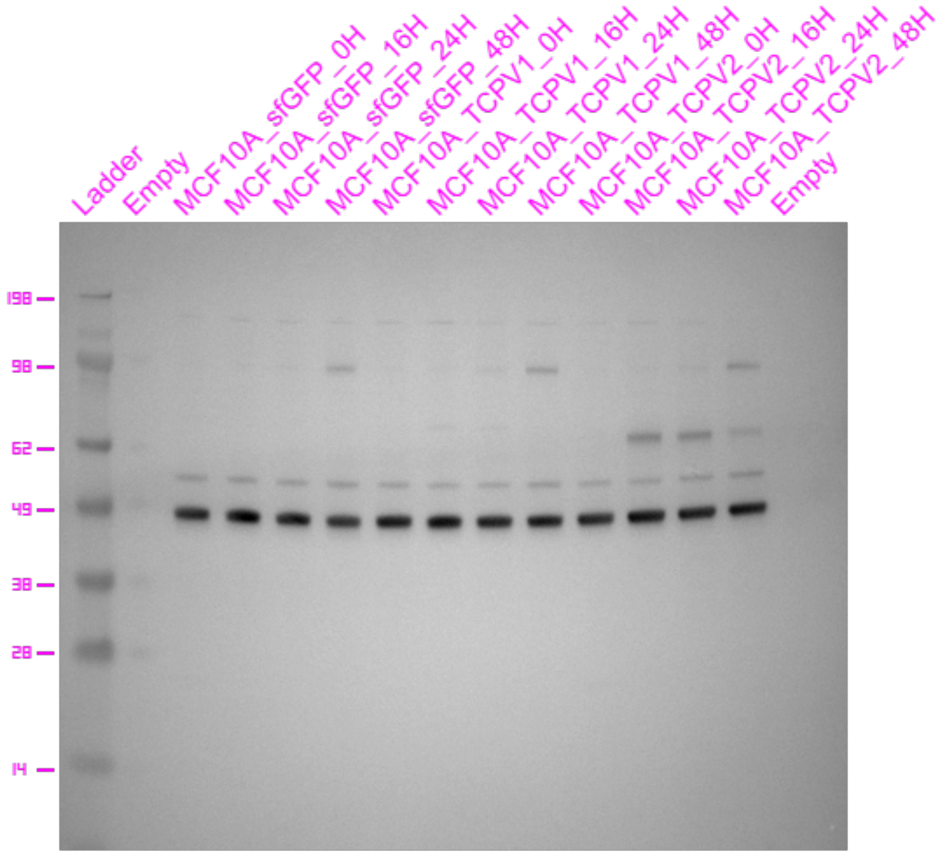

CHEMI_07072021_143521_500ms_10A_TCP_TIME_0_16_24_48_ALPHA_TUB

Date:	07-07-2021 02:35:21PM
Mode:	Chemi Blots
Notes:	
Model:	FL1500
Instrument name:	2462619090234
Serial No:	2462619090234
Firmware version:	1.6.0
iBA version:	4.0.1
Image size:	451px X 360px
Image area:	
Optical Zoom:	2x
Digital Zoom:	1.5x
Focus level:	455
Resolution:	5 x 5
Exposure time:	500 ms
Exposure mode:	Normal

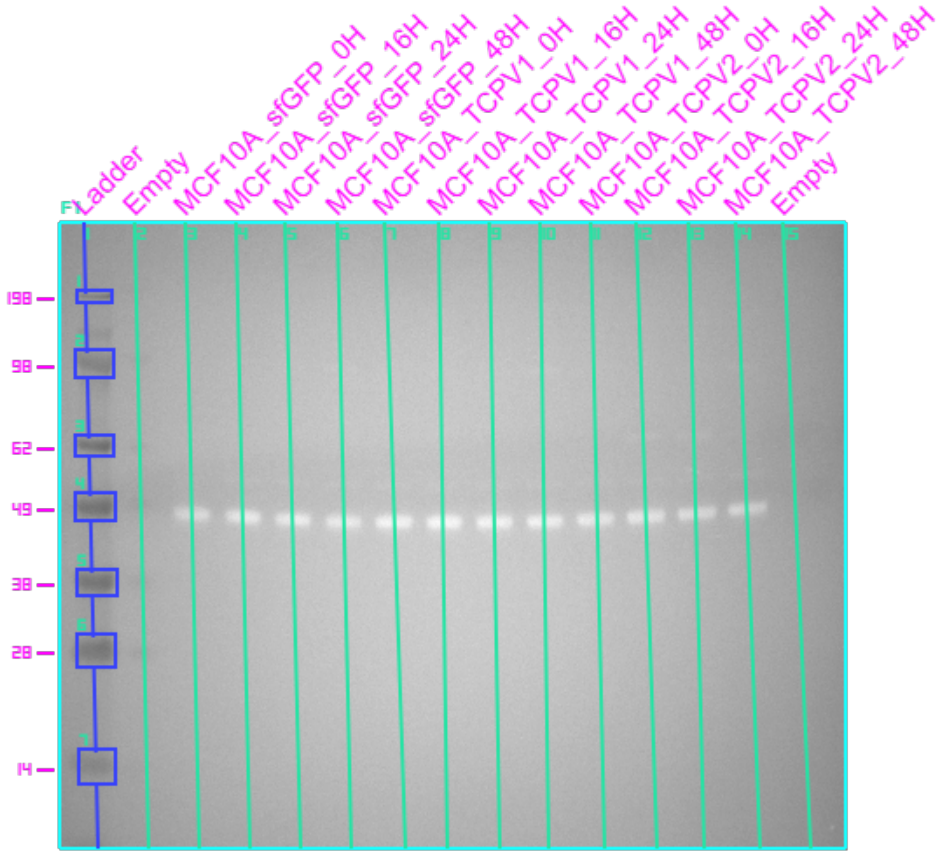

CHEMI_07072021_143521_500ms_10A_TCP_TIME_0_16_24_48_ALPHA_TUB

Date:	07-07-2021 02:35:21PM
Mode:	Chemi Blots
Notes:	
Model:	FL1500
Instrument name:	2462619090234
Serial No:	2462619090234
Firmware version:	1.6.0
iBA version:	4.0.1
Image size:	451px X 360px
Image area:	
Optical Zoom:	2x
Digital Zoom:	1.5x
Focus level:	455
Resolution:	5 x 5
Exposure time:	500 ms
Exposure mode:	Normal

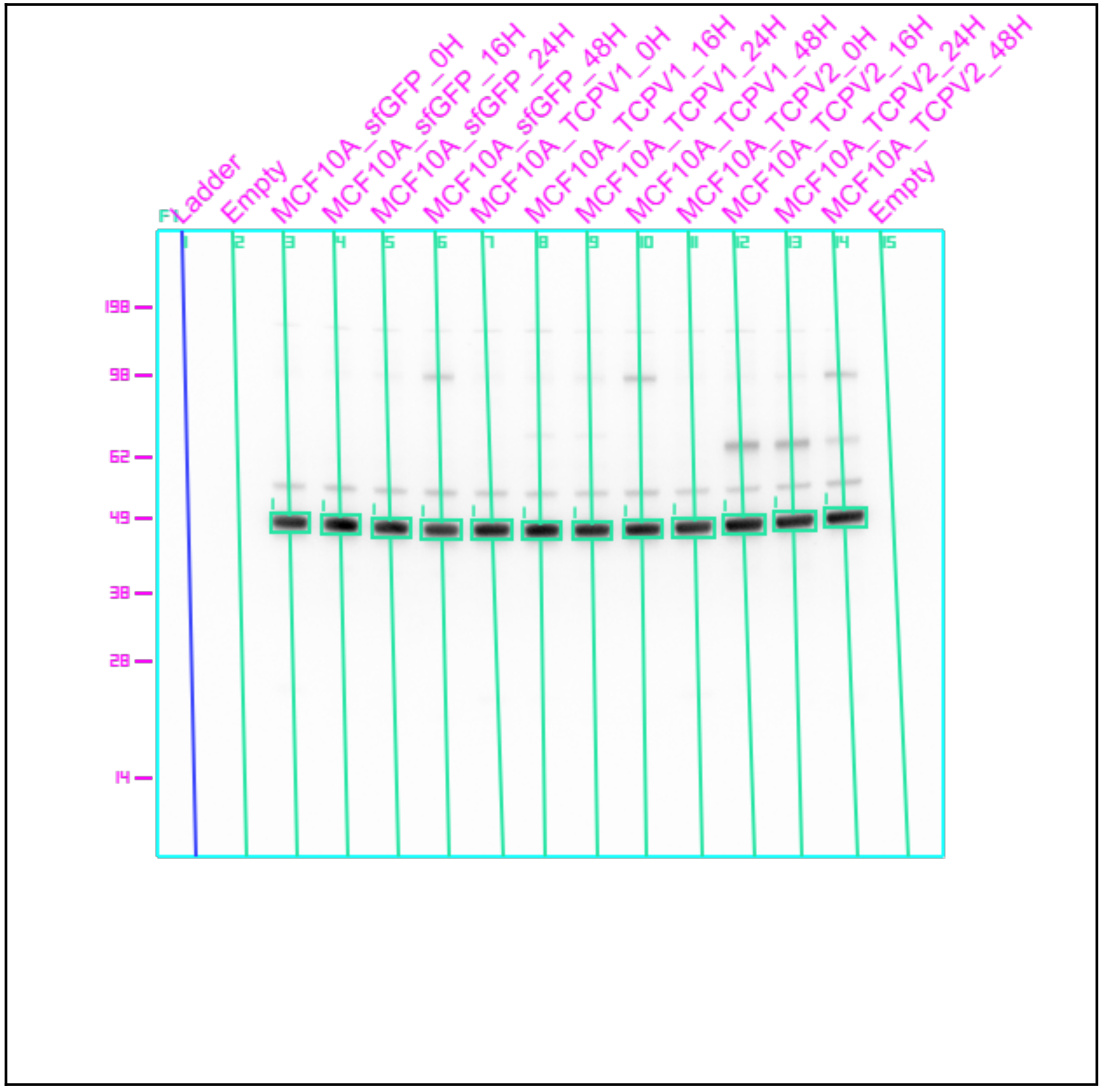

LANE AND BAND ANALYSIS DATA TABLE

CHEMI_07072021_143521_500ms_10A_TCP_TIME_0_16_24_48_ALPHA_TUB

Frame: 1
Channel: Membrane
Sensitivity: 100
Molecular Weight Analysis Regression Method : Point to Point

Lane 1 - Ladder

#	Vol. (Int.)	Local Bg. Corr. Vol.	Area	Rf	Density	Local Bg. Corr. Den.	% band purity	% lane purity	Mol. Wt.
1	5,328,251	454,163	168	0.117	31,715	2,703.355	4.059	2.453	198
2	12,343,269	1,434,038	391	0.226	31,568	3,667.618	12.817	5.682	98
3	9,710,132	1,607,096	299	0.357	32,475	5,374.904	14.364	4.469	62
4	13,638,585	2,200,673	408	0.454	33,427	5,393.808	19.67	6.278	49
5	12,767,547	2,186,086	384	0.574	33,248	5,692.933	19.539	5.877	38
6	15,179,331	2,346,674	460	0.682	32,998	5,101.466	20.975	6.987	28
7	14,517,745	959,436	462	0.869	31,423	2,076.703	8.575	6.682	14

Frame: 1
Channel: Chemi
Sensitivity: 100
Molecular Weight Analysis Regression Method : Point to Point

Lane 3 - MCF10A_sfGFP_0H

#	Vol. (Int.)	Local Bg. Corr. Vol.	Area	Rf	Density	Local Bg. Corr. Den.	% band purity	% lane purity	Mol. Wt.
1	8,178,082	6,898,846	276	0.465	29,630	24,995	100	61.235	47.977

Lane 4 - MCF10A_sfGFP_16H

#	Vol. (Int.)	Local Bg. Corr. Vol.	Area	Rf	Density	Local Bg. Corr. Den.	% band purity	% lane purity	Mol. Wt.
1	9,326,842	7,858,018	276	0.468	33,792	28,471	100	60.266	47.721

Lane 5 - MCF10A_sfGFP_24H

#	Vol. (Int.)	Local Bg. Corr. Vol.	Area	Rf	Density	Local Bg. Corr. Den.	% band purity	% lane purity	Mol. Wt.
1	8,561,457	7,116,823	276	0.474	31,019	25,785	100	56.479	47.209

Lane 6 - MCF10A_sfGFP_48H

#	Vol. (Int.)	Local Bg. Corr. Vol.	Area	Rf	Density	Local Bg. Corr. Den.	% band purity	% lane purity	Mol. Wt.
1	7,834,492	6,329,448	288	0.476	27,203	21,977	100	49.045	46.953

Lane 7 - MCF10A_TCPV1_0H

#	Vol. (Int.)	Local Bg. Corr. Vol.	Area	Rf	Density	Local Bg. Corr. Den.	% band purity	% lane purity	Mol. Wt.
1	9,125,705	7,636,883	288	0.476	31,686	26,516	100	57.245	46.953

Lane 8 - MCF10A_TCPV1_16H

#	Vol. (Int.)	Local Bg. Corr. Vol.	Area	Rf	Density	Local Bg. Corr. Den.	% band purity	% lane purity	Mol. Wt.
1	8,939,859	7,501,863	253	0.479	35,335	29,651	100	56.016	46.698

Lane 9 - MCF10A_TCPV1_24H

#	Vol. (Int.)	Local Bg. Corr. Vol.	Area	Rf	Density	Local Bg. Corr. Den.	% band purity	% lane purity	Mol. Wt.
1	8,393,798	6,934,233	253	0.479	33,177	27,408	100	53.153	46.698

Lane 10 - MCF10A_TCPV1_48H

#	Vol. (Int.)	Local Bg. Corr. Vol.	Area	Rf	Density	Local Bg. Corr. Den.	% band purity	% lane purity	Mol. Wt.
1	8,462,798	6,988,520	253	0.476	33,449	27,622	100	49.584	46.953

Lane 11 - MCF10A_TCPV2_0H

#	Vol. (Int.)	Local Bg. Corr. Vol.	Area	Rf	Density	Local Bg. Corr. Den.	% band purity	% lane purity	Mol. Wt.
1	8,726,632	7,066,552	300	0.474	29,088	23,555	100	57.46	47.209

Lane 12 - MCF10A_TCPV2_16H

#	Vol. (Int.)	Local Bg. Corr. Vol.	Area	Rf	Density	Local Bg. Corr. Den.	% band purity	% lane purity	Mol. Wt.
1	10,032,861	8,149,757	300	0.468	33,442	27,165	100	49.843	47.721

Lane 13 - MCF10A_TCPV2_24H

#	Vol. (Int.)	Local Bg. Corr. Vol.	Area	Rf	Density	Local Bg. Corr. Den.	% band purity	% lane purity	Mol. Wt.
1	9,259,612	7,473,471	300	0.462	30,865	24,911	100	49.337	48.233

Lane 14 - MCF10A_TCPV2_48H

#	Vol. (Int.)	Local Bg. Corr. Vol.	Area	Rf	Density	Local Bg. Corr. Den.	% band purity	% lane purity	Mol. Wt.
1	9,274,504	7,862,494	300	0.457	30,915	26,208	100	54.356	48.744

iBright™ Image Analysis Report

28 January 2022

Figure 1B- FLAG

CHEMI_07222021_153812

Date:	07-22-2021 03:38:12PM
Mode:	Chemi Blots
Notes:	
Model:	FL1500
Instrument name:	2462619090234
Serial No:	2462619090234
Firmware version:	1.6.0
iBA version:	4.0.1
Image size:	483px X 386px
Image area:	
Optical Zoom:	2x
Digital Zoom:	1.4x
Focus level:	455
Resolution:	5 x 5
Exposure time:	2750 ms
Exposure mode:	Normal

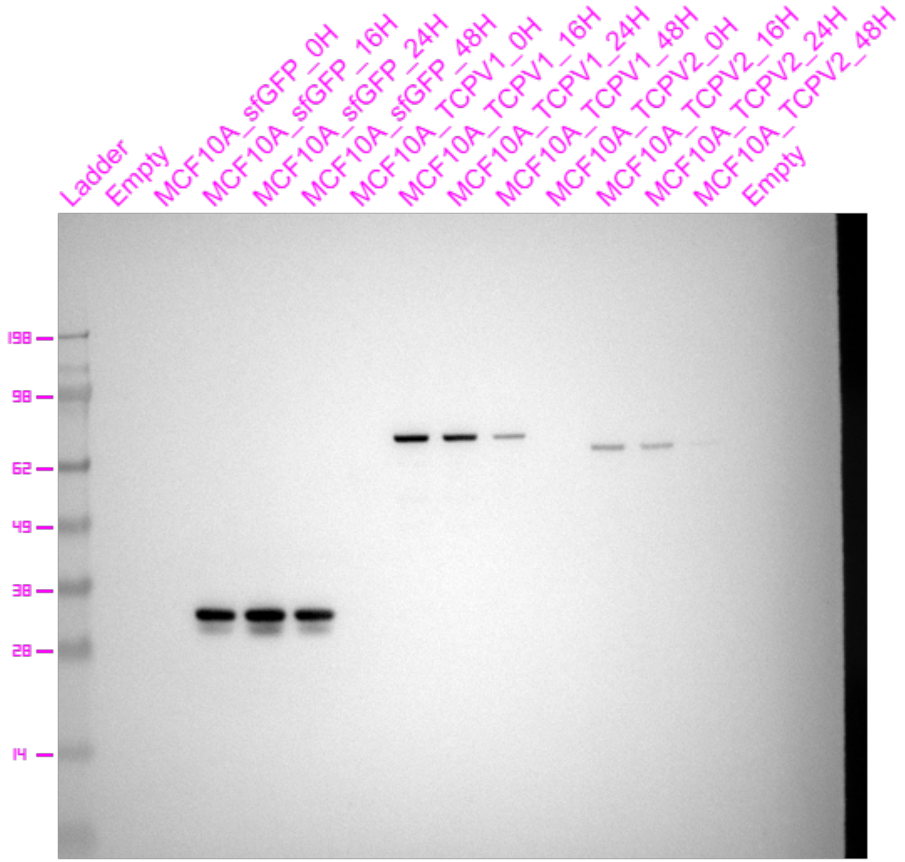

CHEMI_07222021_153812

Date:	07-22-2021 03:38:12PM
Mode:	Chemi Blots
Notes:	
Model:	FL1500
Instrument name:	2462619090234
Serial No:	2462619090234
Firmware version:	1.6.0
iBA version:	4.0.1
Image size:	483px X 386px
Image area:	
Optical Zoom:	2x
Digital Zoom:	1.4x
Focus level:	455
Resolution:	5 x 5
Exposure time:	2750 ms
Exposure mode:	Normal

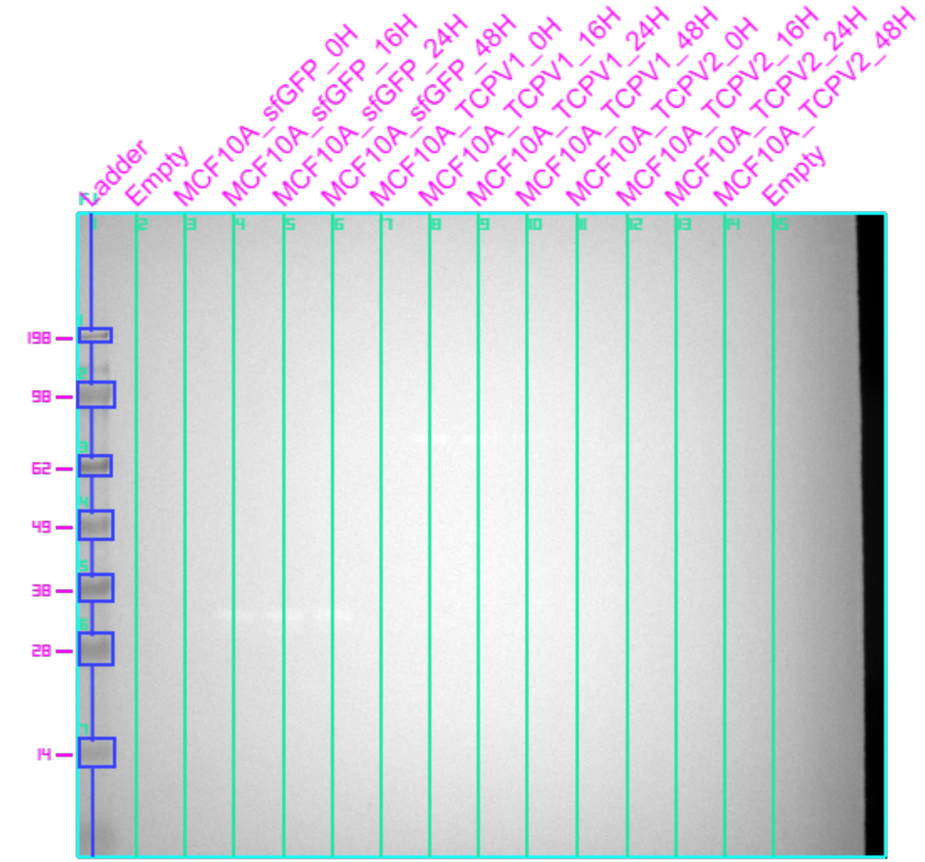

CHEMI_07222021_153812

Date:	07-22-2021 03:38:12PM
Mode:	Chemi Blots
Notes:	
Model:	FL1500
Instrument name:	2462619090234
Serial No:	2462619090234
Firmware version:	1.6.0
iBA version:	4.0.1
Image size:	483px X 386px
Image area:	
Optical Zoom:	2x
Digital Zoom:	1.4x
Focus level:	455
Resolution:	5 x 5
Exposure time:	2750 ms
Exposure mode:	Normal

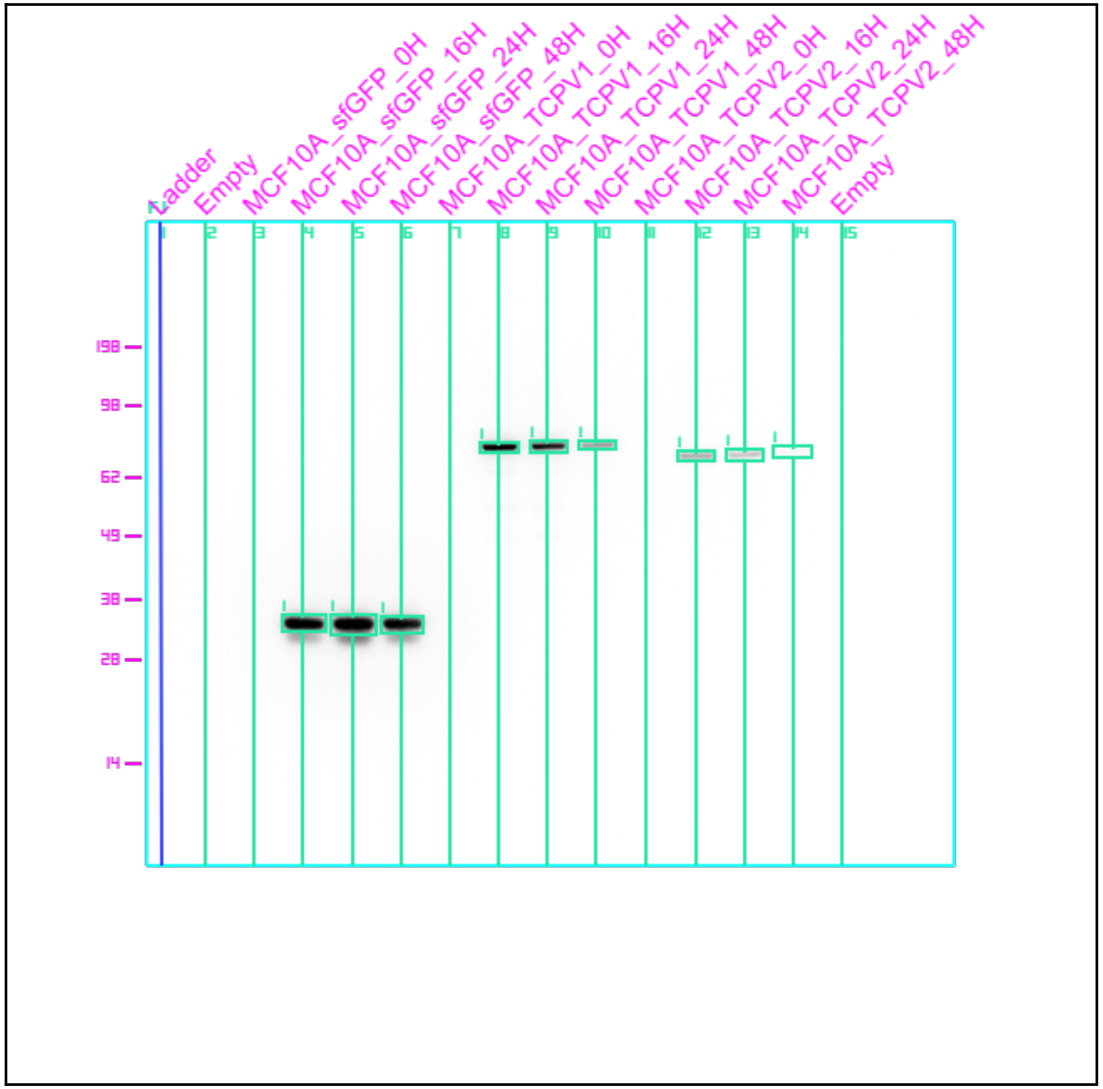

LANE AND BAND ANALYSIS DATA TABLE

CHEMI_07222021_153812

Frame: 1
Channel: Membrane
Sensitivity: 100
Molecular Weight Analysis Regression Method : Point to Point

Lane 1 - Ladder

#	Vol. (Int.)	Local Bg. Corr. Vol.	Area	Rf	Density	Local Bg. Corr. Den.	% band purity	% lane purity	Mol. Wt.
1	5,834,414	596,464	180	0.19	32,413	3,313.692	6.131	2.441	198
2	12,286,875	1,499,510	368	0.281	33,388	4,074.756	15.412	5.141	98
3	9,045,010	1,464,720	260	0.392	34,788	5,633.541	15.055	3.784	62
4	12,490,027	1,576,208	378	0.483	33,042	4,169.863	16.201	5.226	49
5	12,329,902	1,787,023	357	0.582	34,537	5,005.667	18.367	5.159	38
6	14,316,507	1,812,116	420	0.675	34,086	4,314.564	18.625	5.99	28
7	13,056,421	993,246	396	0.836	32,970	2,508.198	10.209	5.463	14

Frame: 1
Channel: Chemi
Sensitivity: 100
Molecular Weight Analysis Regression Method : Point to Point

Lane 4 - MCF10A_sfGFP_16H

#	Vol. (Int.)	Local Bg. Corr. Vol.	Area	Rf	Density	Local Bg. Corr. Den.	% band purity	% lane purity	Mol. Wt.
1	9,501,149	7,257,670	297	0.623	31,990	24,436	100	NA	33.556

Lane 5 - MCF10A_sfGFP_24H

#	Vol. (Int.)	Local Bg. Corr. Vol.	Area	Rf	Density	Local Bg. Corr. Den.	% band purity	% lane purity	Mol. Wt.
1	11,969,893	8,499,310	364	0.626	32,884	23,349	100	NA	33.278

Lane 6 - MCF10A_sfGFP_48H

#	Vol. (Int.)	Local Bg. Corr. Vol.	Area	Rf	Density	Local Bg. Corr. Den.	% band purity	% lane purity	Mol. Wt.
1	8,582,847	6,548,309	286	0.626	30,009	22,896	100	NA	33.278

Lane 8 - MCF10A_TCPV1_16H

#	Vol. (Int.)	Local Bg. Corr. Vol.	Area	Rf	Density	Local Bg. Corr. Den.	% band purity	% lane purity	Mol. Wt.
1	4,643,843	4,112,351	168	0.351	27,641	24,478	100	NA	75.395

Lane 9 - MCF10A_TCPV1_24H

#	Vol. (Int.)	Local Bg. Corr. Vol.	Area	Rf	Density	Local Bg. Corr. Den.	% band purity	% lane purity	Mol. Wt.
1	3,901,183	3,441,929	184	0.348	21,202	18,706	100	NA	76.233

Lane 10 - MCF10A_TCPV1_48H

#	Vol. (Int.)	Local Bg. Corr. Vol.	Area	Rf	Density	Local Bg. Corr. Den.	% band purity	% lane purity	Mol. Wt.
1	1,248,175	1,076,479	138	0.345	9,044.746	7,800.577	100	NA	77.07

Lane 12 - MCF10A_TCPV2_16H

#	Vol. (Int.)	Local Bg. Corr. Vol.	Area	Rf	Density	Local Bg. Corr. Den.	% band purity	% lane purity	Mol. Wt.
1	1,277,117	1,132,592	161	0.364	7,932.404	7,034.736	100	NA	71.209

Lane 13 - MCF10A_TCPV2_24H

#	Vol. (Int.)	Local Bg. Corr. Vol.	Area	Rf	Density	Local Bg. Corr. Den.	% band purity	% lane purity	Mol. Wt.
1	891,505	779,411	184	0.361	4,845.136	4,235.933	100	NA	72.047

Lane 14 - MCF10A_TCPV2_48H

#	Vol. (Int.)	Local Bg. Corr. Vol.	Area	Rf	Density	Local Bg. Corr. Den.	% band purity	% lane purity	Mol. Wt.
1	158,625	114,599	192	0.356	826.172	596.872	100	NA	73.721

iBright™ Image Analysis Report

28 January 2022

Figure 1B- GAPDH

CHEMI_07072021_144034_960ms_10A_TCP _TIME_0_16_24_48_GAPDH

Date:	07-07-2021 02:40:34PM
Mode:	Chemi Blots
Notes:	
Model:	FL1500
Instrument name:	2462619090234
Serial No:	2462619090234
Firmware version:	1.6.0
iBA version:	4.0.1
Image size:	451px X 360px
Image area:	
Optical Zoom:	2x
Digital Zoom:	1.5x
Focus level:	455
Resolution:	5 x 5
Exposure time:	960 ms
Exposure mode:	Normal

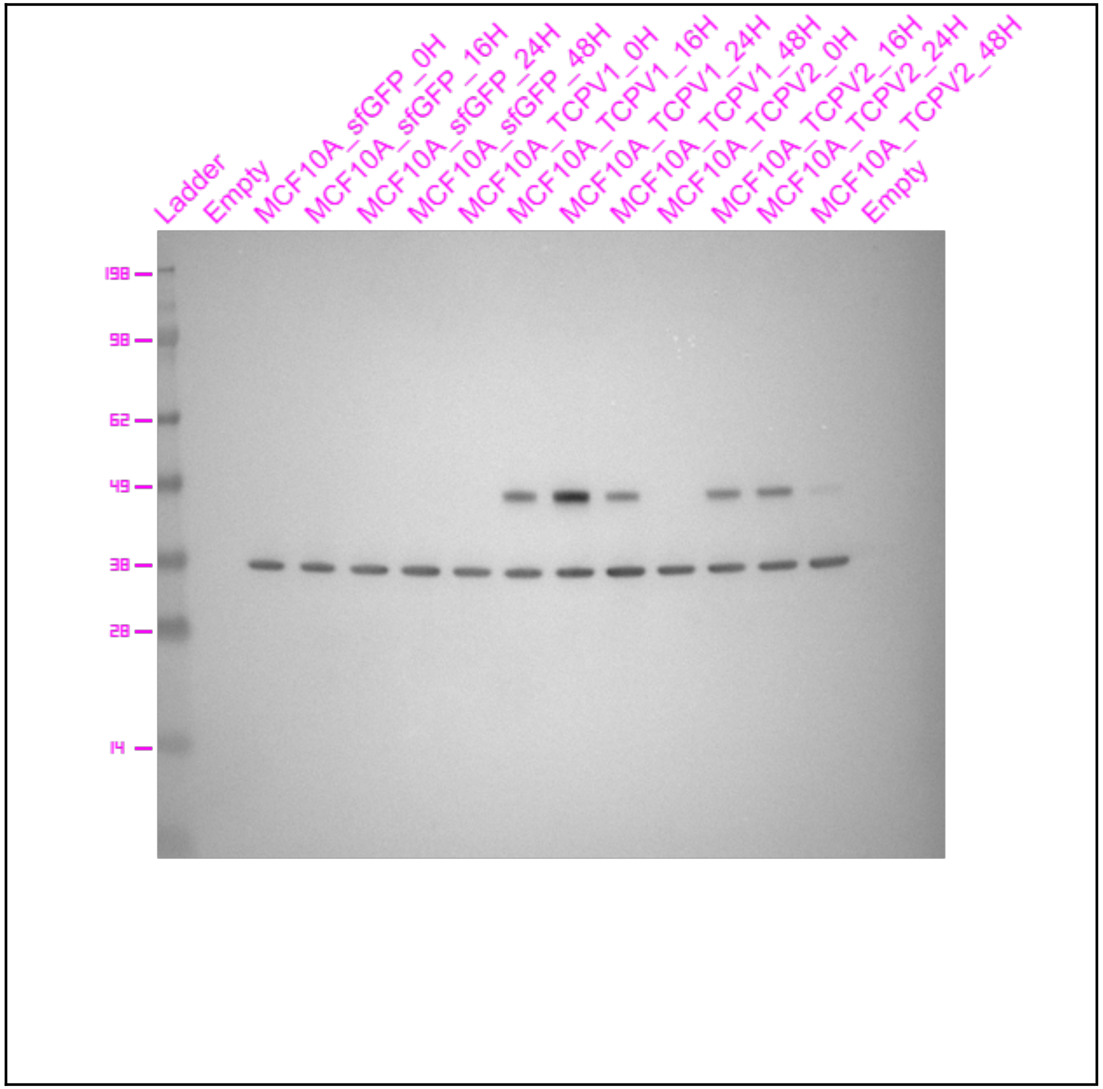

CHEMI_07072021_144034_960ms_10A_TCP_TIME_0_16_24_48_GAPDH

Date:	07-07-2021 02:40:34PM
Mode:	Chemi Blots
Notes:	
Model:	FL1500
Instrument name:	2462619090234
Serial No:	2462619090234
Firmware version:	1.6.0
iBA version:	4.0.1
Image size:	451px X 360px
Image area:	
Optical Zoom:	2x
Digital Zoom:	1.5x
Focus level:	455
Resolution:	5 x 5
Exposure time:	960 ms
Exposure mode:	Normal

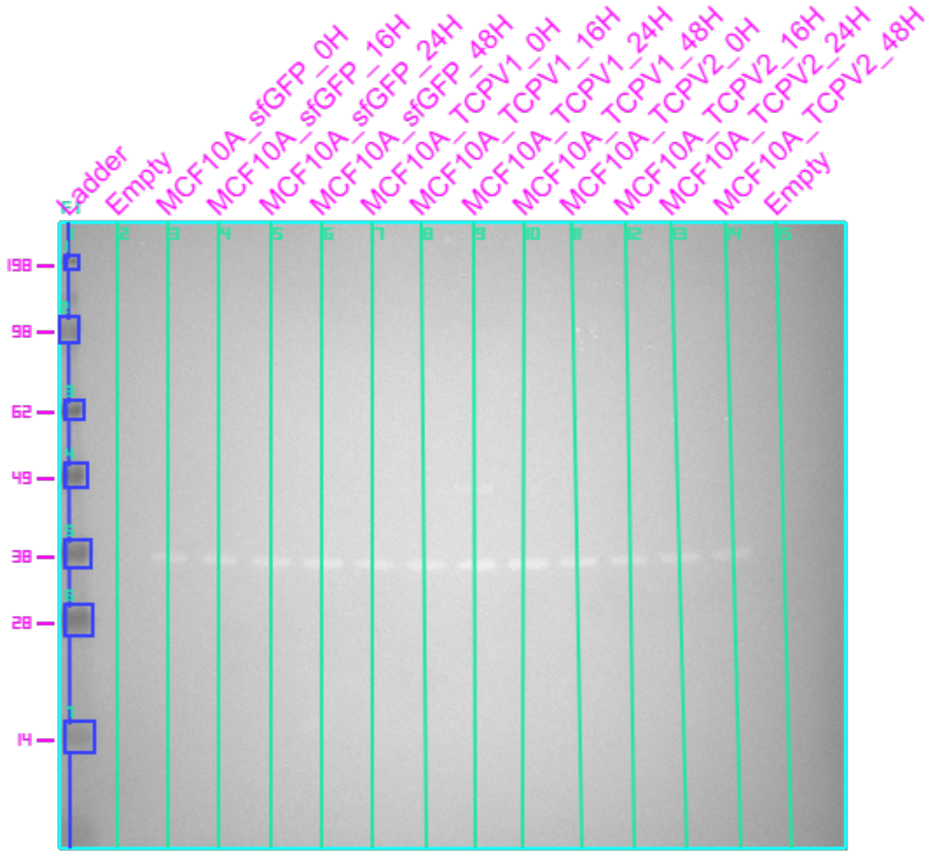

CHEMI_07072021_144034_960ms_10A_TCP_TIME_0_16_24_48_GAPDH

Date:	07-07-2021 02:40:34PM
Mode:	Chemi Blots
Notes:	
Model:	FL1500
Instrument name:	2462619090234
Serial No:	2462619090234
Firmware version:	1.6.0
iBA version:	4.0.1
Image size:	451px X 360px
Image area:	
Optical Zoom:	2x
Digital Zoom:	1.5x
Focus level:	455
Resolution:	5 x 5
Exposure time:	960 ms
Exposure mode:	Normal

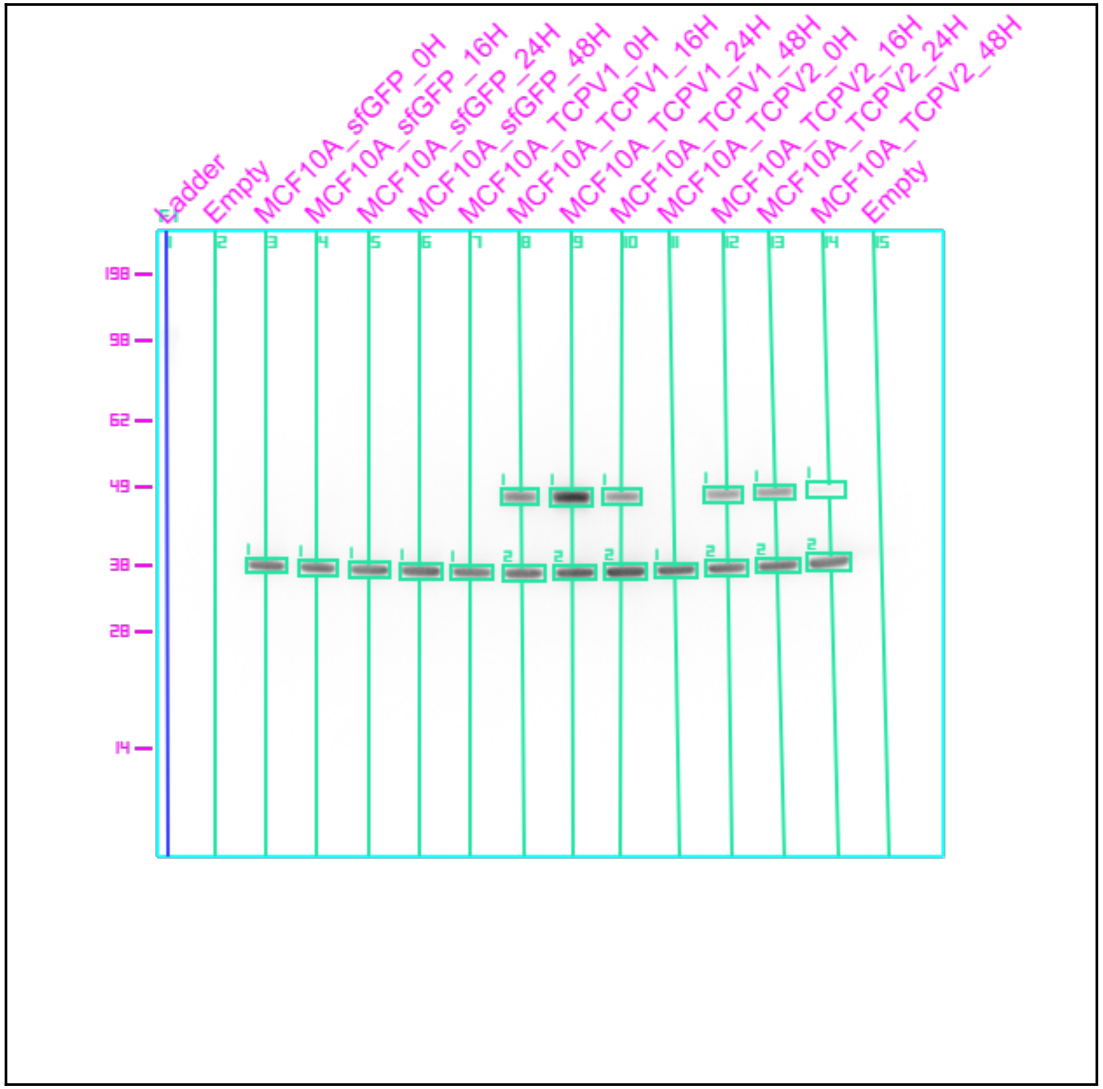

LANE AND BAND ANALYSIS DATA TABLE

CHEMI_07072021_144034_960ms_10A_TCP_TIME_0_16_24_48_GAPDH

Frame: 1
Channel: Membrane
Sensitivity: 100
Molecular Weight Analysis Regression Method : Point to Point

Lane 1 - Ladder

#	Vol. (Int.)	Local Bg. Corr. Vol.	Area	Rf	Density	Local Bg. Corr. Den.	% band purity	% lane purity	Mol. Wt.
1	2,664,454	210,544	81	0.064	32,894	2,599.317	3.201	1.438	198
2	6,456,977	796,932	192	0.17	33,630	4,150.688	12.116	3.484	98
3	4,908,054	747,094	144	0.298	34,083	5,188.154	11.359	2.649	62
4	7,167,478	1,084,246	210	0.404	34,130	5,163.079	16.485	3.868	49
5	9,211,939	1,317,877	272	0.529	33,867	4,845.139	20.037	4.971	38
6	11,157,472	1,636,506	323	0.635	34,543	5,066.584	24.881	6.021	28
7	11,115,339	784,148	342	0.822	32,500	2,292.831	11.922	5.998	14

Frame: 1
Channel: Chemi
Sensitivity: 100
Molecular Weight Analysis Regression Method : Point to Point

Lane 3 - MCF10A_sfGFP_0H

#	Vol. (Int.)	Local Bg. Corr. Vol.	Area	Rf	Density	Local Bg. Corr. Den.	% band purity	% lane purity	Mol. Wt.
1	3,809,277	3,278,117	216	0.535	17,635	15,176	100	61.382	37.474

Lane 4 - MCF10A_sfGFP_16H

#	Vol. (Int.)	Local Bg. Corr. Vol.	Area	Rf	Density	Local Bg. Corr. Den.	% band purity	% lane purity	Mol. Wt.
1	3,773,303	3,172,017	230	0.538	16,405	13,791	100	58.127	37.211

Lane 5 - MCF10A_sfGFP_24H

#	Vol. (Int.)	Local Bg. Corr. Vol.	Area	Rf	Density	Local Bg. Corr. Den.	% band purity	% lane purity	Mol. Wt.
1	4,098,989	3,412,030	240	0.54	17,079	14,216	100	58.323	36.947

Lane 6 - MCF10A_sfGFP_48H

#	Vol. (Int.)	Local Bg. Corr. Vol.	Area	Rf	Density	Local Bg. Corr. Den.	% band purity	% lane purity	Mol. Wt.
1	4,670,602	3,874,230	250	0.543	18,682	15,496	100	59.448	36.684

Lane 7 - MCF10A_TCPV1_0H

#	Vol. (Int.)	Local Bg. Corr. Vol.	Area	Rf	Density	Local Bg. Corr. Den.	% band purity	% lane purity	Mol. Wt.
1	3,902,230	3,207,610	225	0.546	17,343	14,256	100	54.016	36.421

Lane 8 - MCF10A_TCPV1_16H

#	Vol. (Int.)	Local Bg. Corr. Vol.	Area	Rf	Density	Local Bg. Corr. Den.	% band purity	% lane purity	Mol. Wt.
1	3,447,894	2,819,050	220	0.423	15,672	12,813	43.816	29.502	47.289
2	4,397,315	3,614,845	250	0.546	17,589	14,459	56.184	37.625	36.421

Lane 9 - MCF10A_TCPV1_24H

#	Vol. (Int.)	Local Bg. Corr. Vol.	Area	Rf	Density	Local Bg. Corr. Den.	% band purity	% lane purity	Mol. Wt.
1	7,093,626	5,914,391	275	0.426	25,795	21,506	59.782	41.928	47.044
2	4,880,766	3,978,839	225	0.546	21,692	17,683	40.218	28.849	36.421

Lane 10 - MCF10A_TCPV1_48H

#	Vol. (Int.)	Local Bg. Corr. Vol.	Area	Rf	Density	Local Bg. Corr. Den.	% band purity	% lane purity	Mol. Wt.
1	2,988,089	2,352,528	230	0.423	12,991	10,228	33.261	23	47.289
2	5,760,979	4,720,356	250	0.543	23,043	18,881	66.739	44.343	36.684

Lane 11 - MCF10A_TCPV2_0H

#	Vol. (Int.)	Local Bg. Corr. Vol.	Area	Rf	Density	Local Bg. Corr. Den.	% band purity	% lane purity	Mol. Wt.
1	4,410,858	3,576,060	225	0.543	19,603	15,893	100	52.211	36.684

Lane 12 - MCF10A_TCPV2_16H

#	Vol. (Int.)	Local Bg. Corr. Vol.	Area	Rf	Density	Local Bg. Corr. Den.	% band purity	% lane purity	Mol. Wt.
1	2,738,560	2,209,624	230	0.421	11,906	9,607.064	38.842	26.824	47.533
2	4,258,114	3,479,057	250	0.538	17,032	13,916	61.158	41.709	37.211

Lane 13 - MCF10A_TCPV2_24H

#	Vol. (Int.)	Local Bg. Corr. Vol.	Area	Rf	Density	Local Bg. Corr. Den.	% band purity	% lane purity	Mol. Wt.
1	2,755,935	2,262,954	216	0.418	12,758	10,476	39.395	26.967	47.778
2	4,279,317	3,481,245	234	0.535	18,287	14,877	60.605	41.874	37.474

Lane 14 - MCF10A_TCPV2_48H

#	Vol. (Int.)	Local Bg. Corr. Vol.	Area	Rf	Density	Local Bg. Corr. Den.	% band purity	% lane purity	Mol. Wt.
1	488,418	315,414	230	0.412	2,123.557	1,371.367	7.401	7.132	48.267
2	4,700,638	3,946,201	286	0.529	16,435	13,797	92.599	68.636	38

iBright™ Image Analysis Report

28 February 2022

Supplemental Figure 2- Tyr-Tub

CHEMI_02252022_145454_2s_900ms_10A_ TCP_TIMECOURSE_TYRTUB

Date:	02-25-2022 02:54:54PM
Mode:	Chemi Blots
Notes:	
Model:	FL1500
Instrument name:	2462619090234
Serial No:	2462619090234
Firmware version:	1.6.0
iBA version:	4.0.1
Image size:	563px X 450px
Image area:	
Optical Zoom:	2x
Digital Zoom:	1.2x
Focus level:	455
Resolution:	5 x 5
Exposure time:	2900 ms
Exposure mode:	Normal

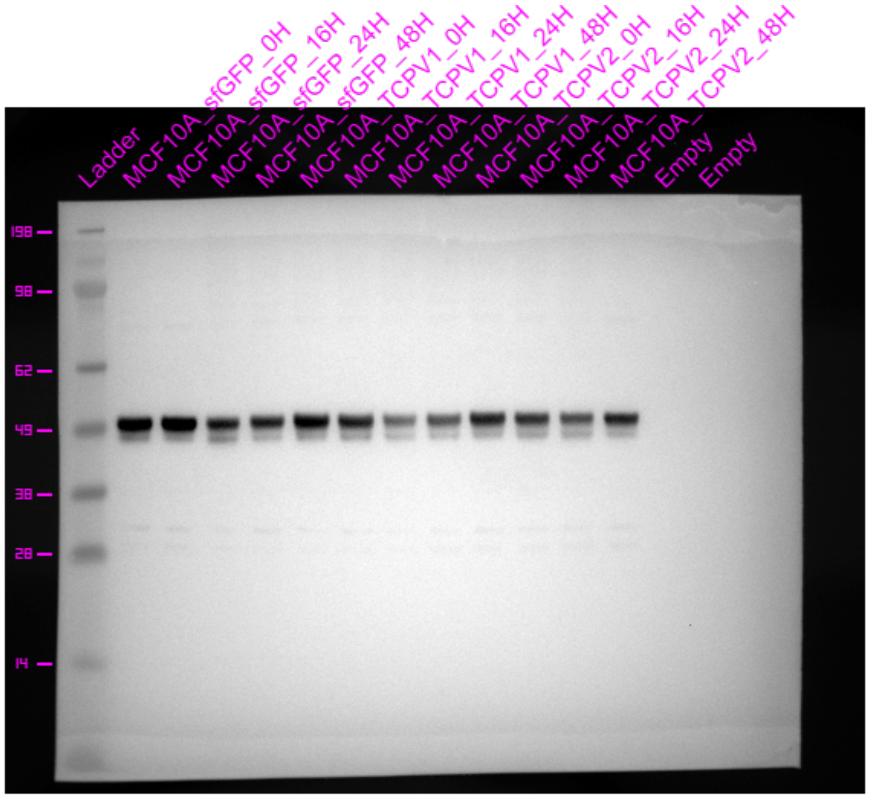

CHEMI_02252022_145454_2s_900ms_10A_ TCP_TIMECOURSE_TYRTUB

Date:	02-25-2022 02:54:54PM
Mode:	Chemi Blots
Notes:	
Model:	FL1500
Instrument name:	2462619090234
Serial No:	2462619090234
Firmware version:	1.6.0
iBA version:	4.0.1
Image size:	563px X 450px
Image area:	
Optical Zoom:	2x
Digital Zoom:	1.2x
Focus level:	455
Resolution:	5 x 5
Exposure time:	2900 ms
Exposure mode:	Normal

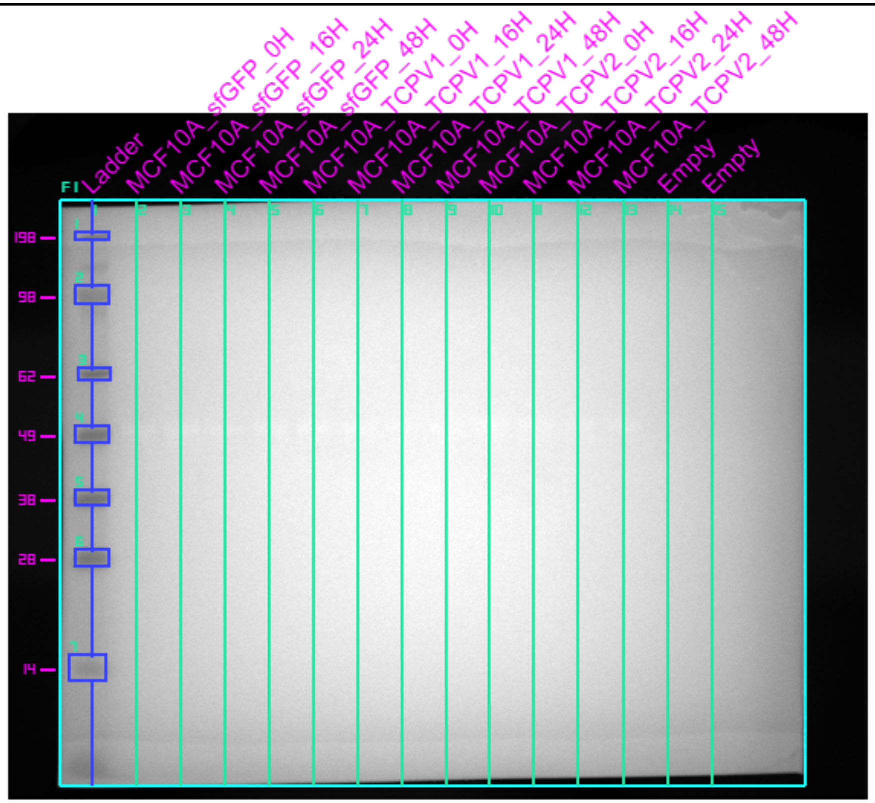

CHEMI_02252022_145454_2s_900ms_10A_ TCP_TIMECOURSE_TYRTUB

Date:	02-25-2022 02:54:54PM
Mode:	Chemi Blots
Notes:	
Model:	FL1500
Instrument name:	2462619090234
Serial No:	2462619090234
Firmware version:	1.6.0
iBA version:	4.0.1
Image size:	563px X 450px
Image area:	
Optical Zoom:	2x
Digital Zoom:	1.2x
Focus level:	455
Resolution:	5 x 5
Exposure time:	2900 ms
Exposure mode:	Normal

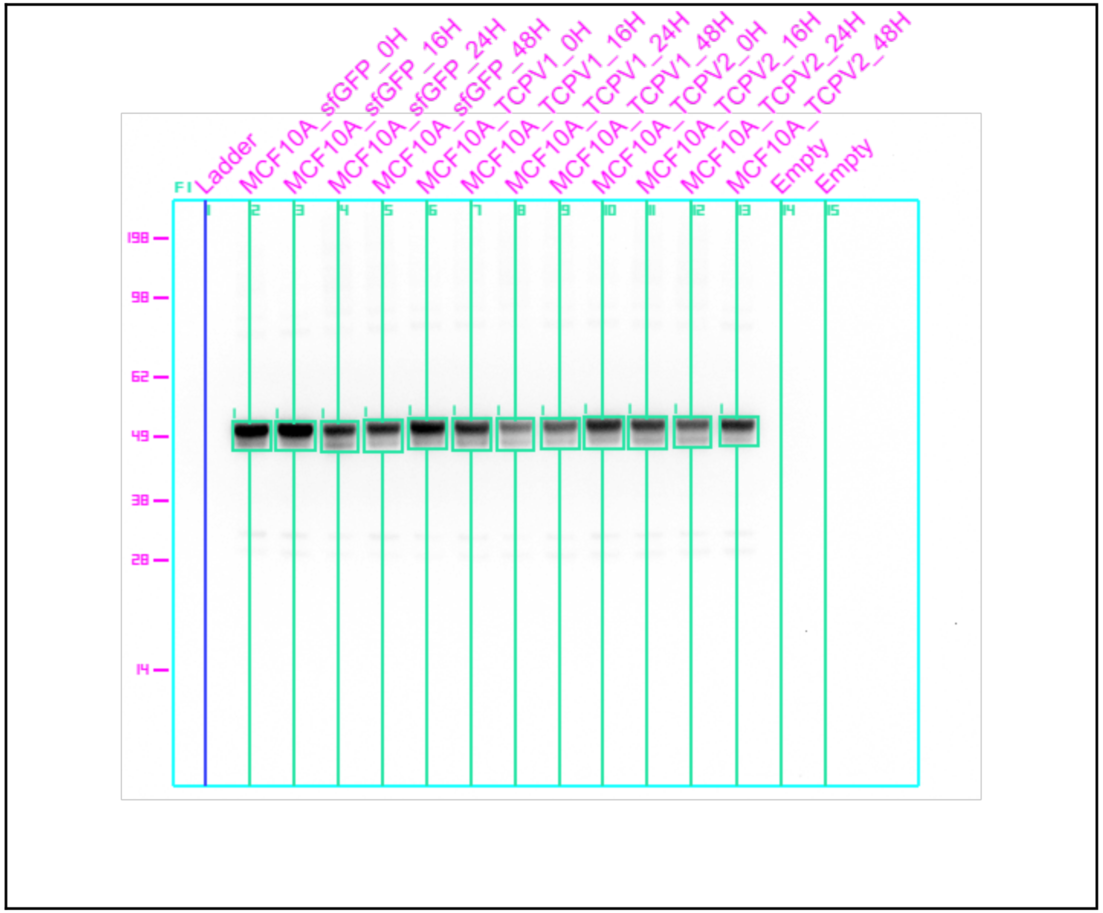

LANE AND BAND ANALYSIS DATA TABLE

CHEMI_02252022_145454_2s_900ms_10A_TCP_TIMECOURSE_TYRTUB

Frame: 1
Channel: Membrane
Sensitivity: 100
Molecular Weight Analysis Regression Method : Point to Point

Lane 1 - Ladder

#	Vol. (Int.)	Local Bg. Corr. Vol.	Area	Rf	Density	Local Bg. Corr. Den.	% band purity	% lane purity	Mol. Wt.
1	5,110,689	387,310	138	0.06	37,033	2,806.598	4.597	1.703	198
2	11,231,541	1,218,214	299	0.161	37,563	4,074.296	14.458	3.743	98
3	7,689,710	1,189,380	198	0.297	38,836	6,006.97	14.116	2.563	62
4	10,830,821	1,666,335	276	0.398	39,242	6,037.447	19.777	3.61	49
5	10,036,900	1,663,533	253	0.508	39,671	6,575.23	19.744	3.345	38
6	10,780,902	1,532,586	276	0.609	39,061	5,552.849	18.189	3.593	28
7	16,102,990	768,349	450	0.797	35,784	1,707.444	9.119	5.367	14

Frame: 1
Channel: Chemi
Sensitivity: 100
Molecular Weight Analysis Regression Method : Point to Point

Lane 2 - MCF10A_sfGFP_0H

#	Vol. (Int.)	Local Bg. Corr. Vol.	Area	Rf	Density	Local Bg. Corr. Den.	% band purity	% lane purity	Mol. Wt.
1	13,514,923	10,967,355	520	0.401	25,990	21,091	100	76.831	48.738

Lane 3 - MCF10A_sfGFP_16H

#	Vol. (Int.)	Local Bg. Corr. Vol.	Area	Rf	Density	Local Bg. Corr. Den.	% band purity	% lane purity	Mol. Wt.
1	14,302,698	11,301,863	540	0.401	26,486	20,929	100	75.281	48.738

Lane 4 - MCF10A_sfGFP_24H

#	Vol. (Int.)	Local Bg. Corr. Vol.	Area	Rf	Density	Local Bg. Corr. Den.	% band purity	% lane purity	Mol. Wt.
1	10,348,066	7,885,999	525	0.404	19,710	15,020	100	64.326	48.476

Lane 5 - MCF10A_sfGFP_48H

#	Vol. (Int.)	Local Bg. Corr. Vol.	Area	Rf	Density	Local Bg. Corr. Den.	% band purity	% lane purity	Mol. Wt.
1	9,809,083	7,186,902	572	0.401	17,148	12,564	100	65.086	48.738

Lane 6 - MCF10A_TCPV1_0H

#	Vol. (Int.)	Local Bg. Corr. Vol.	Area	Rf	Density	Local Bg. Corr. Den.	% band purity	% lane purity	Mol. Wt.
1	12,324,403	9,634,981	546	0.398	22,572	17,646	100	72.284	49

Lane 7 - MCF10A_TCPV1_16H

#	Vol. (Int.)	Local Bg. Corr. Vol.	Area	Rf	Density	Local Bg. Corr. Den.	% band purity	% lane purity	Mol. Wt.
1	10,832,045	8,405,065	572	0.398	18,937	14,694	100	70.261	49

Lane 8 - MCF10A_TCPV1_24H

#	Vol. (Int.)	Local Bg. Corr. Vol.	Area	Rf	Density	Local Bg. Corr. Den.	% band purity	% lane purity	Mol. Wt.
1	6,700,044	4,799,120	550	0.398	12,181	8,725.674	100	58.839	49

Lane 9 - MCF10A_TCPV1_48H

#	Vol. (Int.)	Local Bg. Corr. Vol.	Area	Rf	Density	Local Bg. Corr. Den.	% band purity	% lane purity	Mol. Wt.
1	7,599,091	5,394,763	546	0.398	13,917	9,880.52	100	62.556	49

Lane 10 - MCF10A_TCPV2_0H

#	Vol. (Int.)	Local Bg. Corr. Vol.	Area	Rf	Density	Local Bg. Corr. Den.	% band purity	% lane purity	Mol. Wt.
1	10,621,507	8,034,365	594	0.396	17,881	13,525	100	71.958	49.333

Lane 11 - MCF10A_TCPV2_16H

#	Vol. (Int.)	Local Bg. Corr. Vol.	Area	Rf	Density	Local Bg. Corr. Den.	% band purity	% lane purity	Mol. Wt.
1	9,749,170	7,464,196	572	0.396	17,044	13,049	100	67.797	49.333

Lane 12 - MCF10A_TCPV2_24H

#	Vol. (Int.)	Local Bg. Corr. Vol.	Area	Rf	Density	Local Bg. Corr. Den.	% band purity	% lane purity	Mol. Wt.
1	7,995,423	6,099,189	525	0.396	15,229	11,617	100	62.996	49.333

Lane 13 - MCF10A_TCPV2_48H

#	Vol. (Int.)	Local Bg. Corr. Vol.	Area	Rf	Density	Local Bg. Corr. Den.	% band purity	% lane purity	Mol. Wt.
1	8,962,816	7,495,719	520	0.393	17,236	14,414	100	69.318	49.667

iBright™ Image Analysis Report

28 February 2022

Supplemental Figure 2- GAPDH for Tyr-Tub

CHEMI_02272022_185512_2s_MCF10A_TCP _TIMECOURSE_TYRTUB_GAPDH

Date:	02-27-2022 06:55:12PM
Mode:	Chemi Blots
Notes:	
Model:	FL1500
Instrument name:	2462619090234
Serial No:	2462619090234
Firmware version:	1.6.0
iBA version:	4.0.1
Image size:	676px X 540px
Image area:	
Optical Zoom:	2x
Digital Zoom:	1x
Focus level:	455
Resolution:	5 x 5
Exposure time:	2000 ms
Exposure mode:	Normal

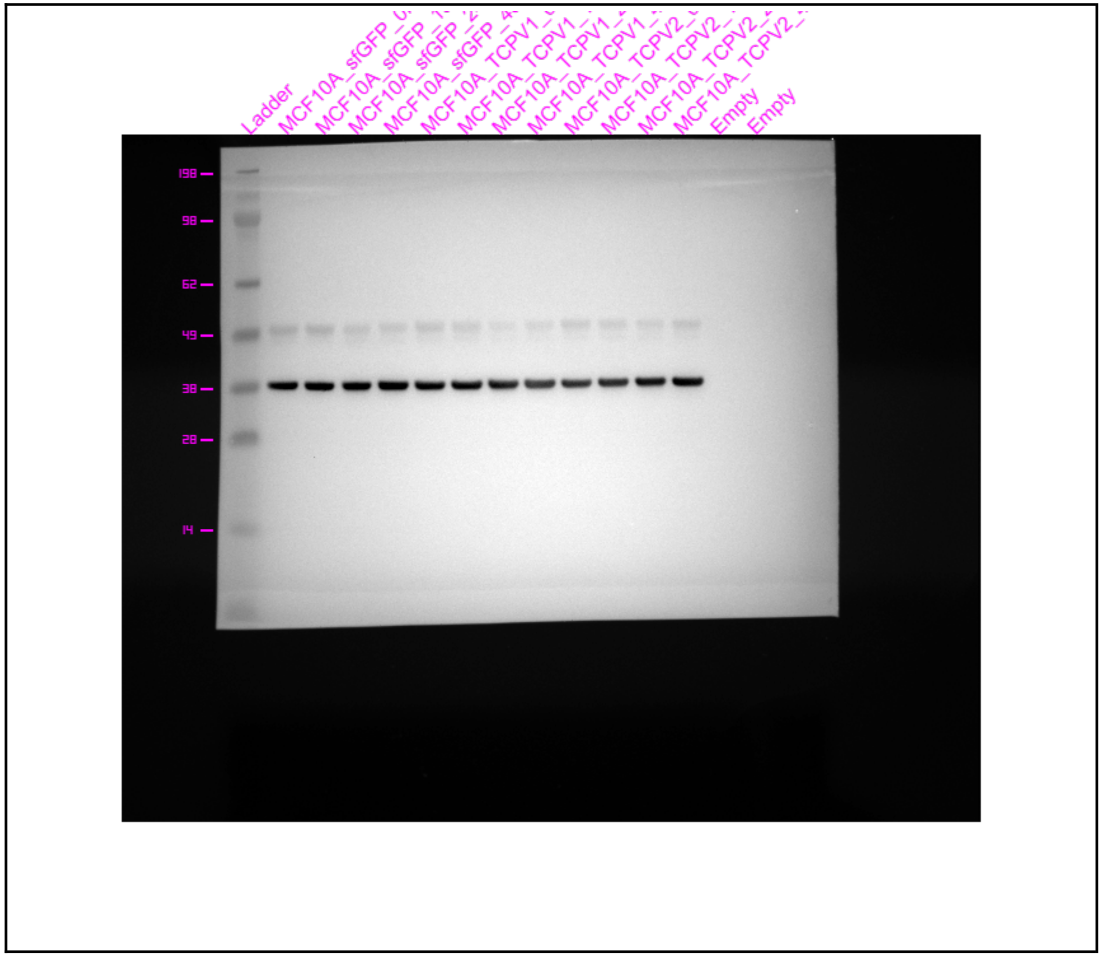

CHEMI_02272022_185512_2s_MCF10A_TCP _TIMECOURSE_TYRTUB_GAPDH

Date:	02-27-2022 06:55:12PM
Mode:	Chemi Blots
Notes:	
Model:	FL1500
Instrument name:	2462619090234
Serial No:	2462619090234
Firmware version:	1.6.0
iBA version:	4.0.1
Image size:	676px X 540px
Image area:	
Optical Zoom:	2x
Digital Zoom:	1x
Focus level:	455
Resolution:	5 x 5
Exposure time:	2000 ms
Exposure mode:	Normal

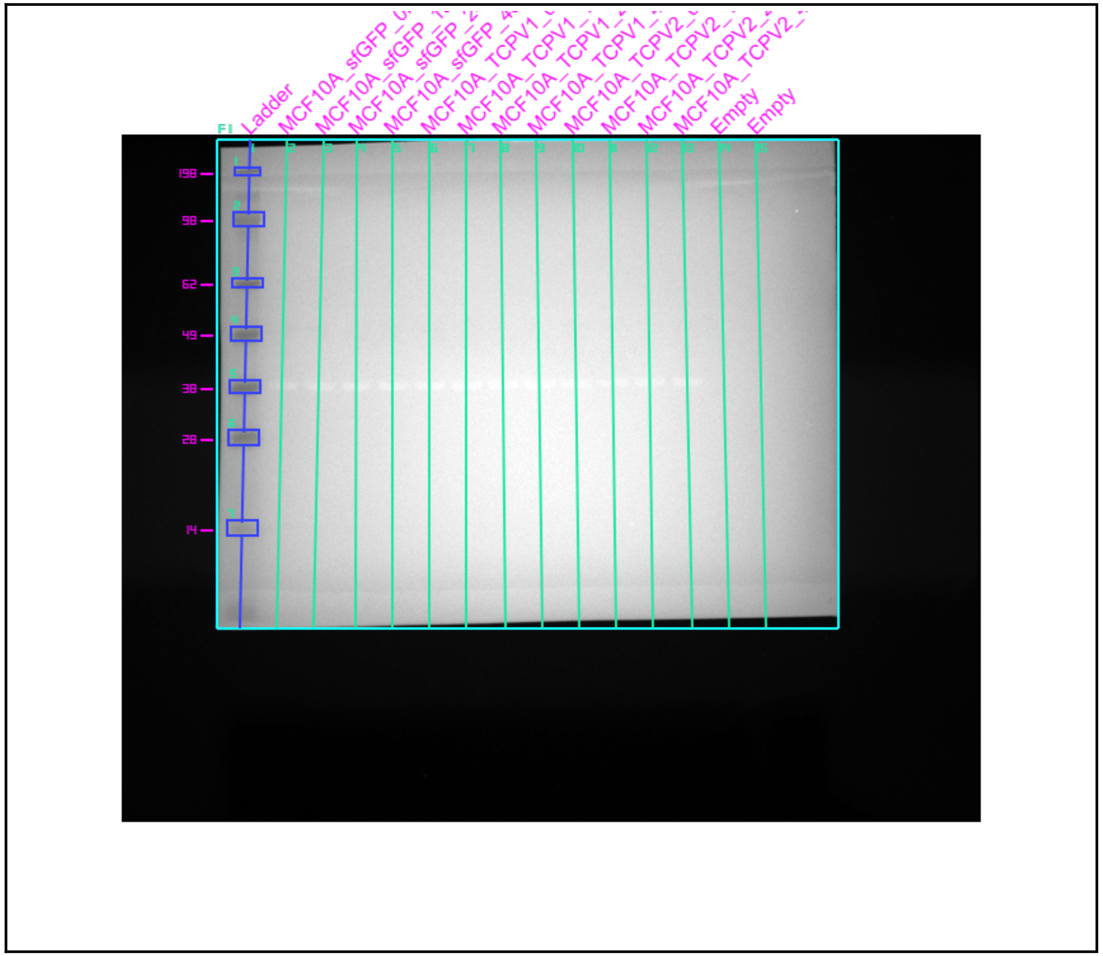

CHEMI_02272022_185512_2s_MCF10A_TCP_TIMECOURSE_TYRTUB_GAPDH

Date:	02-27-2022 06:55:12PM
Mode:	Chemi Blots
Notes:	
Model:	FL1500
Instrument name:	2462619090234
Serial No:	2462619090234
Firmware version:	1.6.0
iBA version:	4.0.1
Image size:	676px X 540px
Image area:	
Optical Zoom:	2x
Digital Zoom:	1x
Focus level:	455
Resolution:	5 x 5
Exposure time:	2000 ms
Exposure mode:	Normal

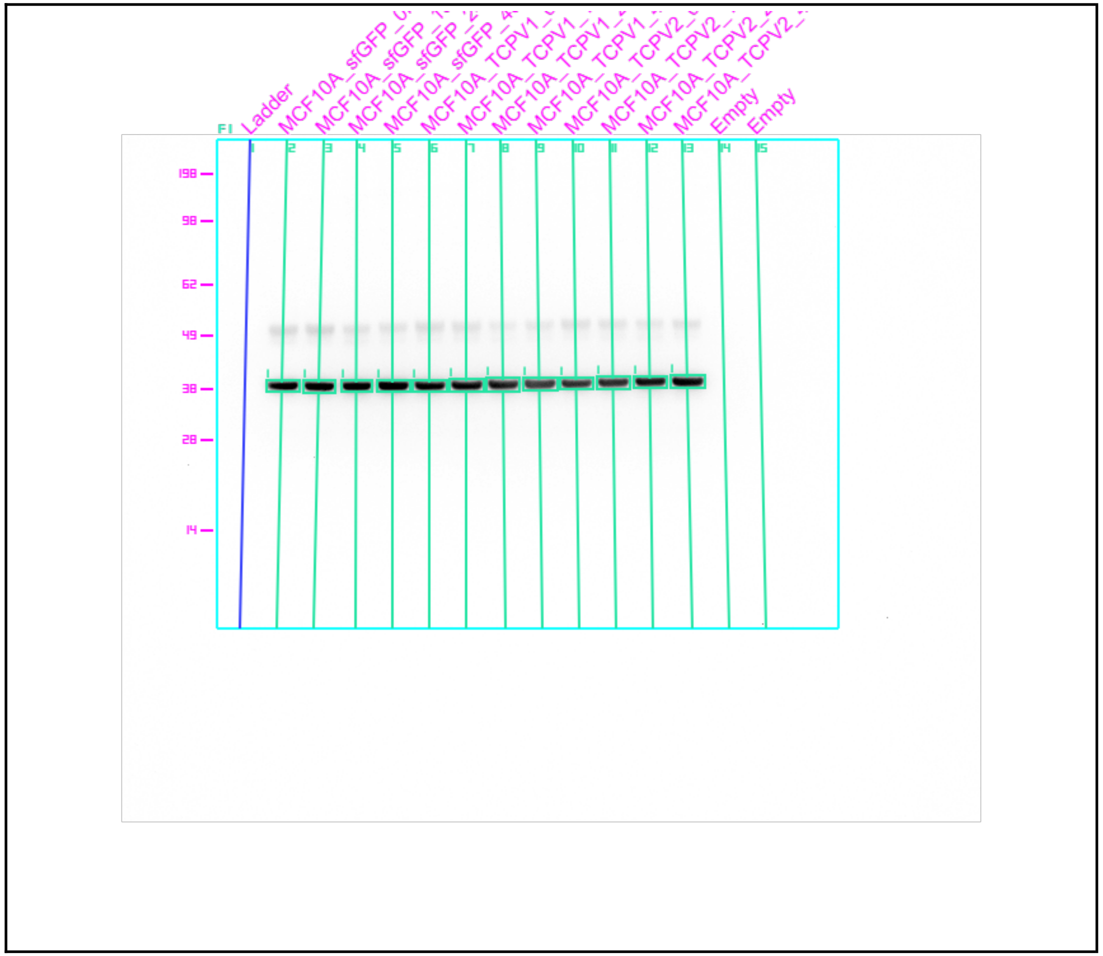

LANE AND BAND ANALYSIS DATA TABLE

CHEMI_02272022_185512_2s_MCF10A_TCP_TIMECOURSE_TYRTUB_GAPDH

Frame: 1
Channel: Membrane
Sensitivity: 100
Molecular Weight Analysis Regression Method : Point to Point

Lane 1 - Ladder

#	Vol. (Int.)	Local Bg. Corr. Vol.	Area	Rf	Density	Local Bg. Corr. Den.	% band purity	% lane purity	Mol. Wt.
1	5,854,737	380,592	147	0.065	39,828	2,589.065	4.76	1.742	198
2	11,740,048	1,166,154	300	0.161	39,133	3,887.181	14.586	3.494	98
3	7,970,203	1,032,054	200	0.292	39,851	5,160.274	12.909	2.372	62
4	11,887,971	1,549,596	300	0.396	39,626	5,165.321	19.382	3.538	49
5	10,900,545	1,579,415	275	0.505	39,638	5,743.329	19.755	3.244	38
6	12,593,174	1,585,354	325	0.609	38,748	4,878.013	19.829	3.748	28
7	11,590,875	701,844	325	0.794	35,664	2,159.523	8.779	3.45	14

Frame: 1
Channel: Chemi
Sensitivity: 100
Molecular Weight Analysis Regression Method : Point to Point

Lane 2 - MCF10A_sfGFP_0H

#	Vol. (Int.)	Local Bg. Corr. Vol.	Area	Rf	Density	Local Bg. Corr. Den.	% band purity	% lane purity	Mol. Wt.
1	7,823,637	6,531,524	270	0.503	28,976	24,190	100	58.628	38.262

Lane 3 - MCF10A_sfGFP_16H

#	Vol. (Int.)	Local Bg. Corr. Vol.	Area	Rf	Density	Local Bg. Corr. Den.	% band purity	% lane purity	Mol. Wt.
1	8,627,736	7,107,872	286	0.505	30,166	24,852	100	56.802	38

Lane 4 - MCF10A_sfGFP_24H

#	Vol. (Int.)	Local Bg. Corr. Vol.	Area	Rf	Density	Local Bg. Corr. Den.	% band purity	% lane purity	Mol. Wt.
1	8,256,292	6,690,896	250	0.503	33,025	26,763	100	54.045	38.262

Lane 5 - MCF10A_sfGFP_48H

#	Vol. (Int.)	Local Bg. Corr. Vol.	Area	Rf	Density	Local Bg. Corr. Den.	% band purity	% lane purity	Mol. Wt.
1	9,052,575	7,090,064	270	0.503	33,528	26,259	100	56.607	38.262

Lane 6 - MCF10A_TCPV1_0H

#	Vol. (Int.)	Local Bg. Corr. Vol.	Area	Rf	Density	Local Bg. Corr. Den.	% band purity	% lane purity	Mol. Wt.
1	7,869,209	5,826,811	280	0.503	28,104	20,810	100	53.197	38.262

Lane 7 - MCF10A_TCPV1_16H

#	Vol. (Int.)	Local Bg. Corr. Vol.	Area	Rf	Density	Local Bg. Corr. Den.	% band purity	% lane purity	Mol. Wt.
1	8,072,999	6,016,843	308	0.503	26,211	19,535	100	55.885	38.262

Lane 8 - MCF10A_TCPV1_24H

#	Vol. (Int.)	Local Bg. Corr. Vol.	Area	Rf	Density	Local Bg. Corr. Den.	% band purity	% lane purity	Mol. Wt.
1	7,647,382	5,695,238	324	0.5	23,603	17,577	100	56.869	38.524

Lane 9 - MCF10A_TCPV1_48H

#	Vol. (Int.)	Local Bg. Corr. Vol.	Area	Rf	Density	Local Bg. Corr. Den.	% band purity	% lane purity	Mol. Wt.
1	6,853,358	5,004,063	308	0.5	22,251	16,246	100	54.877	38.524

Lane 10 - MCF10A_TCPV2_0H

#	Vol. (Int.)	Local Bg. Corr. Vol.	Area	Rf	Density	Local Bg. Corr. Den.	% band purity	% lane purity	Mol. Wt.
1	6,225,486	4,698,822	270	0.497	23,057	17,403	100	50.608	38.786

Lane 11 - MCF10A_TCPV2_16H

#	Vol. (Int.)	Local Bg. Corr. Vol.	Area	Rf	Density	Local Bg. Corr. Den.	% band purity	% lane purity	Mol. Wt.
1	6,732,652	5,172,657	297	0.497	22,668	17,416	100	53.353	38.786

Lane 12 - MCF10A_TCPV2_24H

#	Vol. (Int.)	Local Bg. Corr. Vol.	Area	Rf	Density	Local Bg. Corr. Den.	% band purity	% lane purity	Mol. Wt.
1	7,379,747	5,733,289	270	0.495	27,332	21,234	100	55.057	39.048

Lane 13 - MCF10A_TCPV2_48H

#	Vol. (Int.)	Local Bg. Corr. Vol.	Area	Rf	Density	Local Bg. Corr. Den.	% band purity	% lane purity	Mol. Wt.
1	8,282,732	6,983,242	308	0.495	26,891	22,672	100	60.885	39.048

iBright™ Image Analysis Report

28 January 2022

Figure 1D- deTyr-Tub SHORT Exposure

CHEMI_12162021_162148_651ms_MCF7_TCP_TIMECOURSE_DETYR

Date:	12-16-2021 04:21:48PM
Mode:	Chemi Blots
Notes:	Figure 1D- deTyr-Tub short
Model:	FL1500
Instrument name:	2462619090234
Serial No:	2462619090234
Firmware version:	1.6.0
iBA version:	4.0.1
Image size:	676px X 540px
Image area:	
Optical Zoom:	2x
Digital Zoom:	1x
Focus level:	455
Resolution:	5 x 5
Exposure time:	651 ms
Exposure mode:	Normal

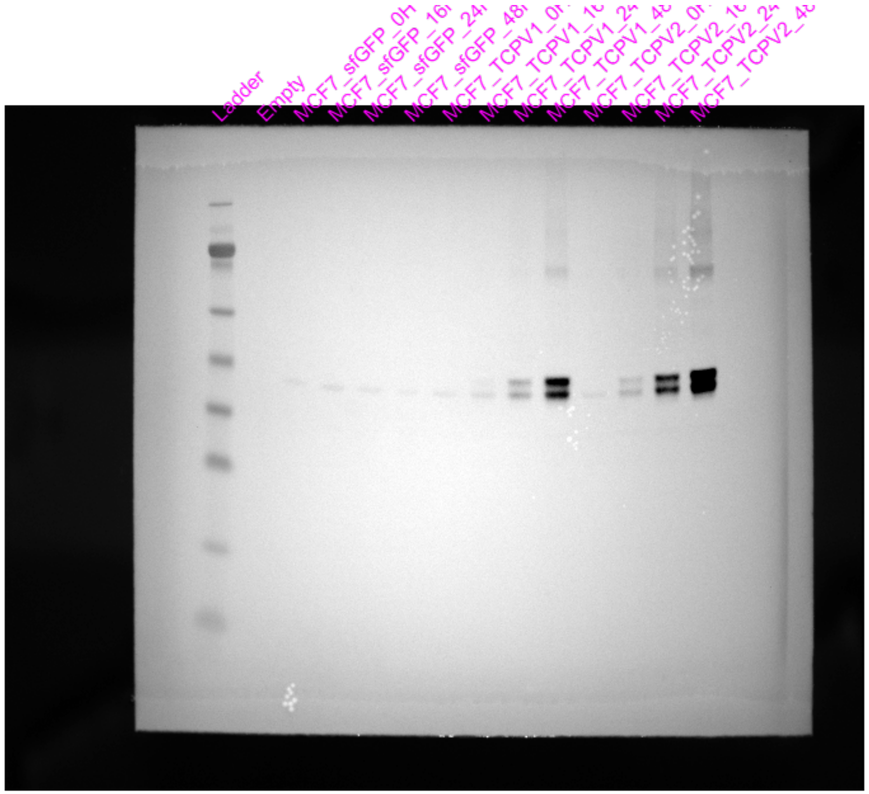

CHEMI_12162021_162148_651ms_MCF7_TCP_TIMECOURSE_DETYR

Date:	12-16-2021 04:21:48PM
Mode:	Chemi Blots
Notes:	Figure 1D- deTyr-Tub short
Model:	FL1500
Instrument name:	2462619090234
Serial No:	2462619090234
Firmware version:	1.6.0
iBA version:	4.0.1
Image size:	676px X 540px
Image area:	
Optical Zoom:	2x
Digital Zoom:	1x
Focus level:	455
Resolution:	5 x 5
Exposure time:	651 ms
Exposure mode:	Normal

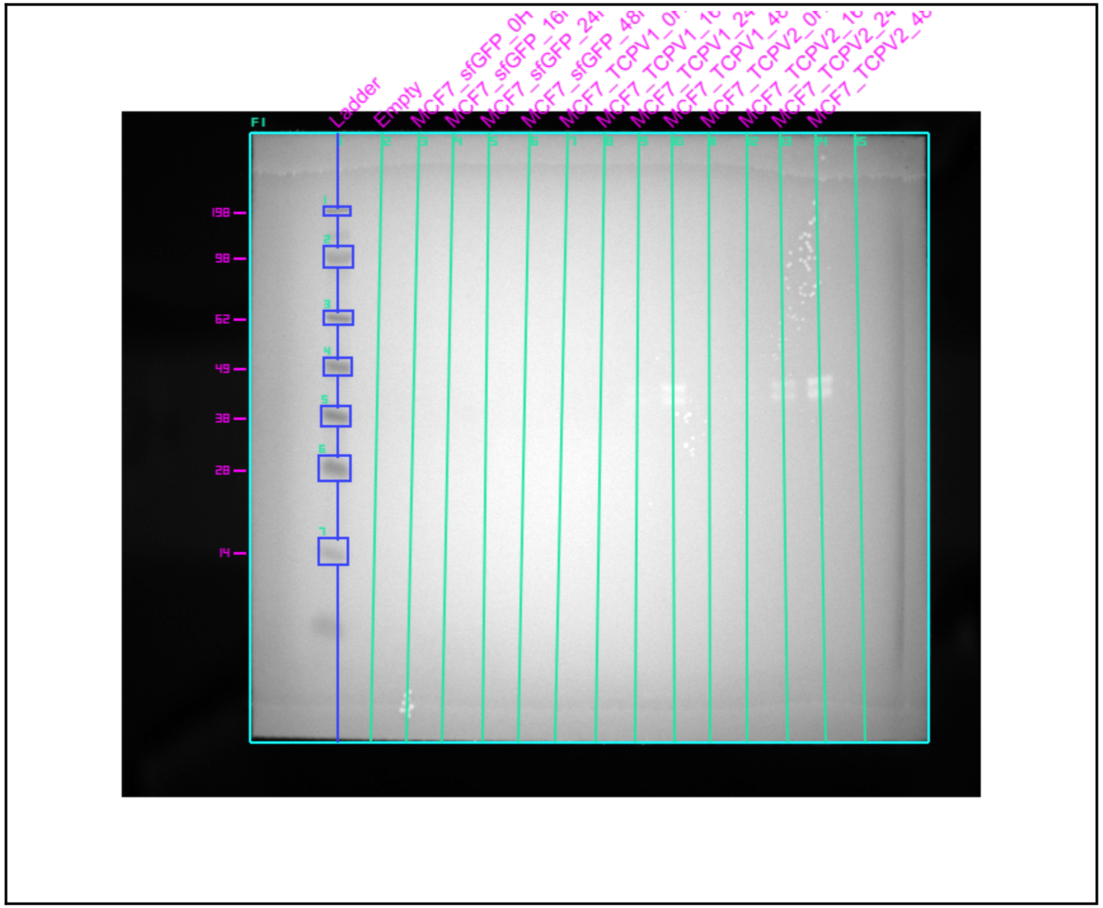

CHEMI_12162021_162148_651ms_MCF7_TCP_TIMECOURSE_DETYR

Date:	12-16-2021 04:21:48PM
Mode:	Chemi Blots
Notes:	Figure 1D- deTyr-Tub short
Model:	FL1500
Instrument name:	2462619090234
Serial No:	2462619090234
Firmware version:	1.6.0
iBA version:	4.0.1
Image size:	676px X 540px
Image area:	
Optical Zoom:	2x
Digital Zoom:	1x
Focus level:	455
Resolution:	5 x 5
Exposure time:	651 ms
Exposure mode:	Normal

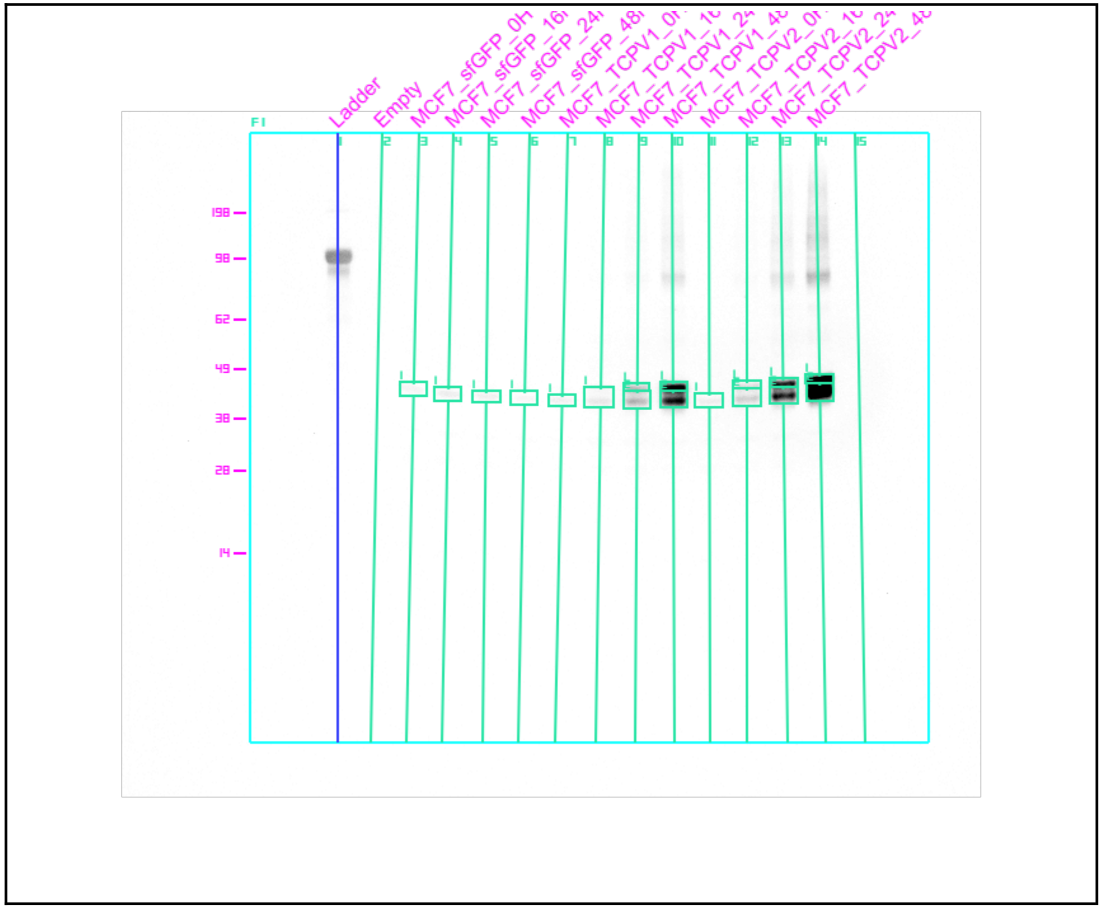

LANE AND BAND ANALYSIS DATA TABLE

CHEMI_12162021_162148_651ms_MCF7_TCP_TIMECOURSE_DETYR

Frame: 1
Channel: Membrane
Sensitivity: 100
Molecular Weight Analysis Regression Method : Point to Point

Lane 1 - Ladder

#	Vol. (Int.)	Local Bg. Corr. Vol.	Area	Rf	Density	Local Bg. Corr. Den.	% band purity	% lane purity	Mol. Wt.
1	5,527,153	464,095	176	0.127	31,404	2,636.908	4.414	2.044	198
2	12,997,224	1,215,895	432	0.202	30,086	2,814.573	11.563	4.806	98
3	8,824,780	1,330,455	288	0.302	30,641	4,619.636	12.653	3.263	62
4	10,849,376	1,785,335	345	0.383	31,447	5,174.886	16.979	4.012	49
5	12,856,034	2,114,281	408	0.465	31,509	5,182.063	20.107	4.754	38
6	16,626,634	2,510,603	546	0.55	30,451	4,598.175	23.876	6.149	28
7	14,957,075	1,094,404	528	0.685	28,327	2,072.736	10.408	5.531	14

Frame: 1
Channel: Chemi
Sensitivity: 100
Molecular Weight Analysis Regression Method : Point to Point

Lane 3 - MCF7_sfGFP_0H

#	Vol. (Int.)	Local Bg. Corr. Vol.	Area	Rf	Density	Local Bg. Corr. Den.	% band purity	% lane purity	Mol. Wt.
1	112,380	85,618	264	0.419	425.682	324.311	100	12.837	44.205

Lane 4 - MCF7_sfGFP_16H

#	Vol. (Int.)	Local Bg. Corr. Vol.	Area	Rf	Density	Local Bg. Corr. Den.	% band purity	% lane purity	Mol. Wt.
1	157,651	126,069	264	0.427	597.163	477.535	100	17.217	43.077

Lane 5 - MCF7_sfGFP_24H

#	Vol. (Int.)	Local Bg. Corr. Vol.	Area	Rf	Density	Local Bg. Corr. Den.	% band purity	% lane purity	Mol. Wt.
1	137,638	107,491	230	0.431	598.426	467.352	100	15.184	42.513

Lane 6 - MCF7_sfGFP_48H

#	Vol. (Int.)	Local Bg. Corr. Vol.	Area	Rf	Density	Local Bg. Corr. Den.	% band purity	% lane purity	Mol. Wt.
1	95,957	63,392	264	0.433	363.473	240.125	100	11.914	42.231

Lane 7 - MCF7_TCPV1_0H

#	Vol. (Int.)	Local Bg. Corr. Vol.	Area	Rf	Density	Local Bg. Corr. Den.	% band purity	% lane purity	Mol. Wt.
1	126,681	92,152	220	0.438	575.823	418.873	100	14.1	41.667

Lane 8 - MCF7_TCPV1_16H

#	Vol. (Int.)	Local Bg. Corr. Vol.	Area	Rf	Density	Local Bg. Corr. Den.	% band purity	% lane purity	Mol. Wt.
1	351,907	232,697	408	0.433	862.517	570.337	100	26.362	42.231

Lane 9 - MCF7_TCPV1_24H

#	Vol. (Int.)	Local Bg. Corr. Vol.	Area	Rf	Density	Local Bg. Corr. Den.	% band purity	% lane purity	Mol. Wt.
1	500,149	335,764	147	0.417	3,402.374	2,284.115	35.532	17.643	44.487
2	1,004,951	609,191	330	0.438	3,045.306	1,846.035	64.468	35.45	41.667

Lane 10 - MCF7_TCPV1_48H

#	Vol. (Int.)	Local Bg. Corr. Vol.	Area	Rf	Density	Local Bg. Corr. Den.	% band purity	% lane purity	Mol. Wt.
1	2,703,639	2,027,641	176	0.415	15,361	11,520	48.713	31.233	44.769
2	3,711,260	2,134,793	308	0.435	12,049	6,931.148	51.287	42.873	41.949

Lane 11 - MCF7_TCPV2_0H

#	Vol. (Int.)	Local Bg. Corr. Vol.	Area	Rf	Density	Local Bg. Corr. Den.	% band purity	% lane purity	Mol. Wt.
1	206,942	85,551	276	0.438	749.79	309.97	100	14.392	41.667

Lane 12 - MCF7_TCPV2_16H

#	Vol. (Int.)	Local Bg. Corr. Vol.	Area	Rf	Density	Local Bg. Corr. Den.	% band purity	% lane purity	Mol. Wt.
1	281,371	153,338	161	0.412	1,747.646	952.416	27.958	11.36	45.051
2	679,960	395,116	345	0.433	1,970.899	1,145.266	72.042	27.452	42.231

Lane 13 - MCF7_TCPV2_24H

#	Vol. (Int.)	Local Bg. Corr. Vol.	Area	Rf	Density	Local Bg. Corr. Den.	% band purity	% lane purity	Mol. Wt.
1	2,023,017	1,434,347	184	0.408	10,994	7,795.369	41.733	26.073	45.615
2	3,190,455	2,002,634	322	0.429	9,908.245	6,219.361	58.267	41.12	42.795

Lane 14 - MCF7_TCPV2_48H

#	Vol. (Int.)	Local Bg. Corr. Vol.	Area	Rf	Density	Local Bg. Corr. Den.	% band purity	% lane purity	Mol. Wt.
1	4,976,039	3,601,469	184	0.402	27,043	19,573	40.233	28.678	46.462
2	8,127,046	5,350,019	330	0.425	24,627	16,212	59.767	46.837	43.359

iBright™ Image Analysis Report

28 January 2022

Figure 1D- deTyr-Tub LONG Exposure

CHEMI_12162021_162228_5s_MCF7_TCP_T IMECOURSE_DETYR

Date:	12-16-2021 04:22:28PM
Mode:	Chemi Blots
Notes:	Figure 1D- deTyr-Tub LONG
Model:	FL1500
Instrument name:	2462619090234
Serial No:	2462619090234
Firmware version:	1.6.0
iBA version:	4.0.1
Image size:	676px X 540px
Image area:	
Optical Zoom:	2x
Digital Zoom:	1x
Focus level:	455
Resolution:	5 x 5
Exposure time:	5000 ms
Exposure mode:	Normal

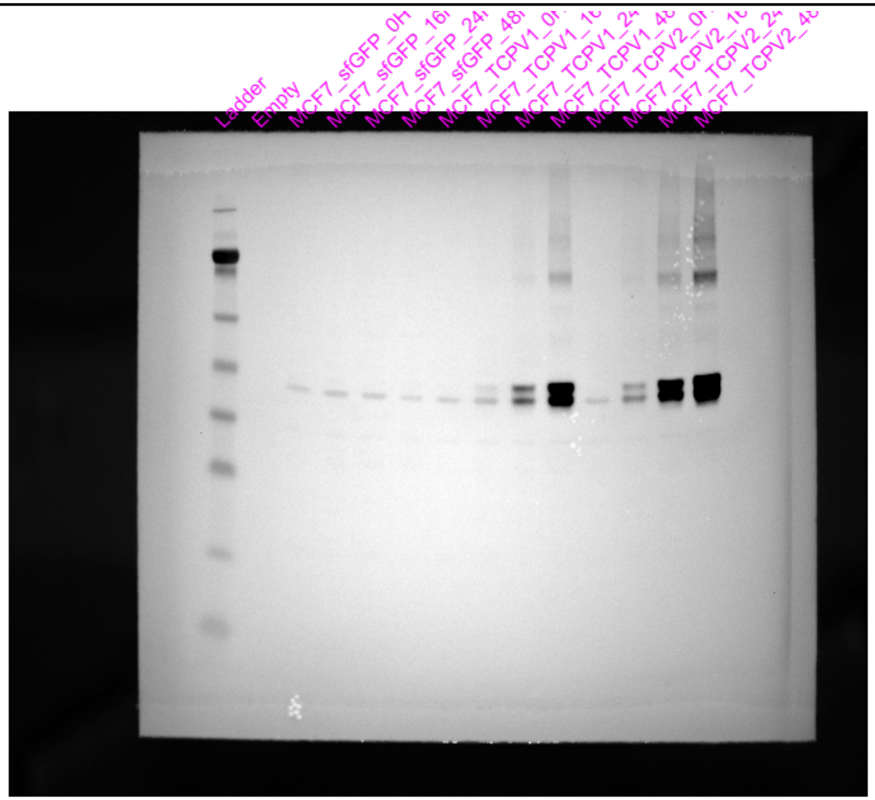

CHEMI_12162021_162228_5s_MCF7_TCP_T IMECOURSE_DETYR

Date:	12-16-2021 04:22:28PM
Mode:	Chemi Blots
Notes:	Figure 1D- deTyr-Tub LONG
Model:	FL1500
Instrument name:	2462619090234
Serial No:	2462619090234
Firmware version:	1.6.0
iBA version:	4.0.1
Image size:	676px X 540px
Image area:	
Optical Zoom:	2x
Digital Zoom:	1x
Focus level:	455
Resolution:	5 x 5
Exposure time:	5000 ms
Exposure mode:	Normal

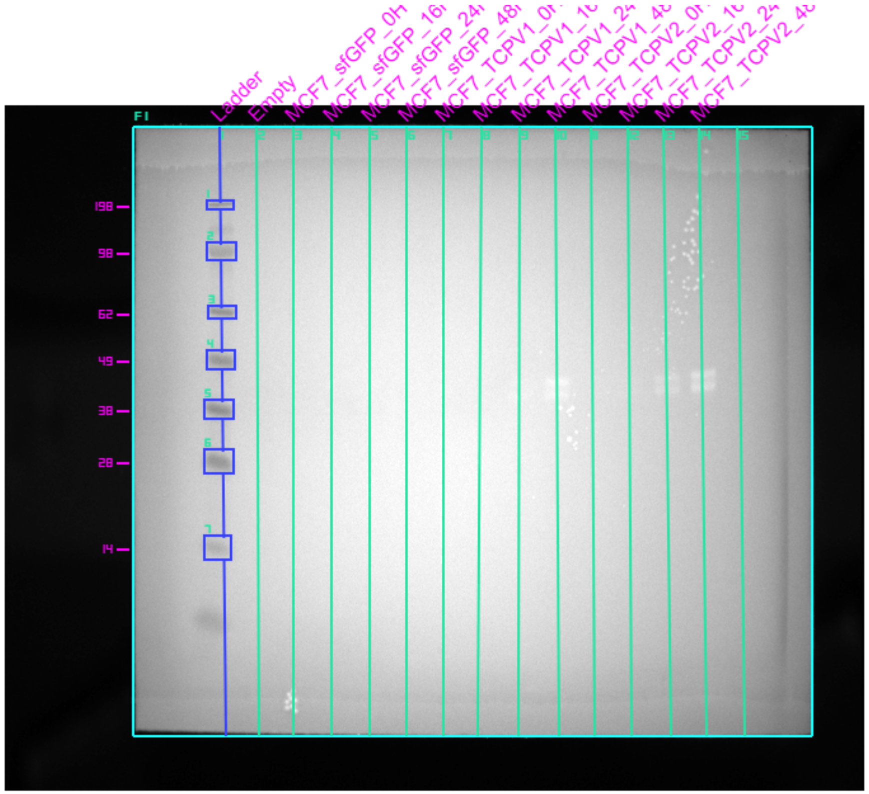

CHEMI_12162021_162228_5s_MCF7_TCP_TIMECOURSE_DETYR

Date:	12-16-2021 04:22:28PM
Mode:	Chemi Blots
Notes:	Figure 1D- deTyr-Tub LONG
Model:	FL1500
Instrument name:	2462619090234
Serial No:	2462619090234
Firmware version:	1.6.0
iBA version:	4.0.1
Image size:	676px X 540px
Image area:	
Optical Zoom:	2x
Digital Zoom:	1x
Focus level:	455
Resolution:	5 x 5
Exposure time:	5000 ms
Exposure mode:	Normal

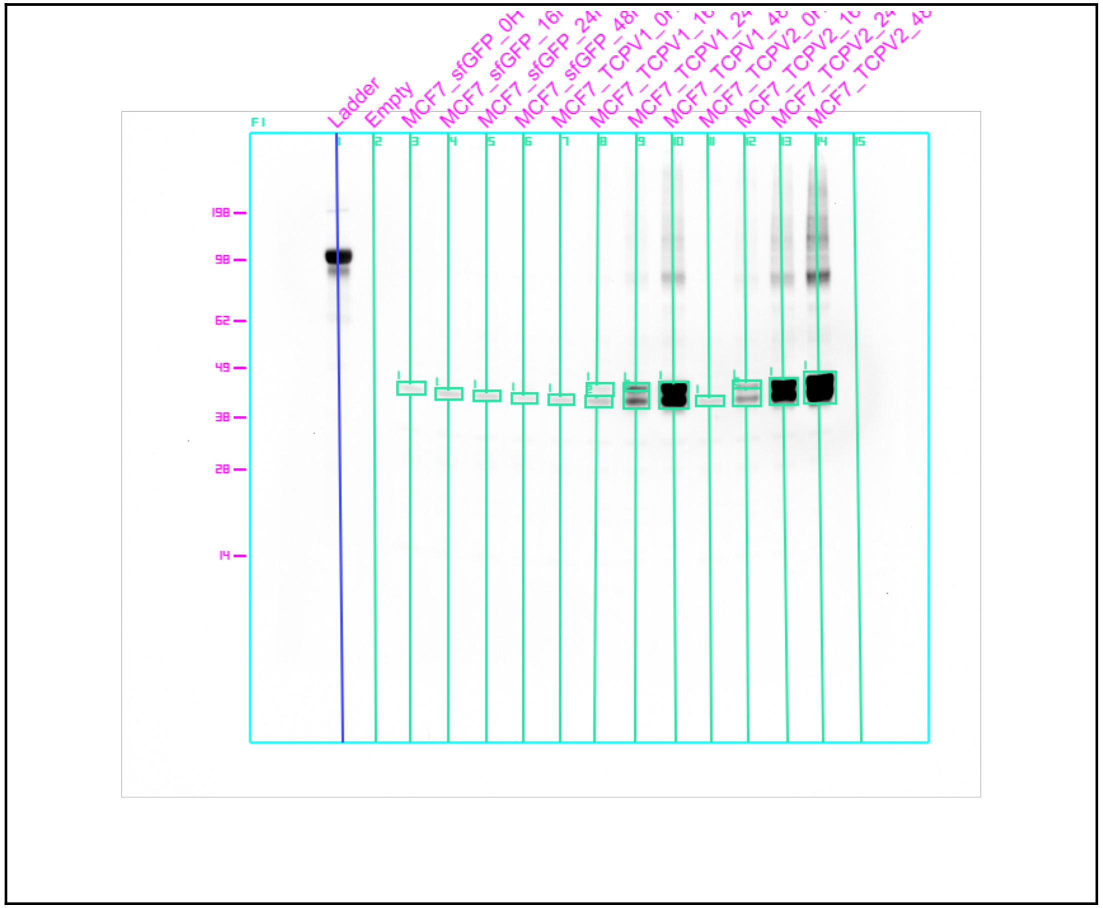

LANE AND BAND ANALYSIS DATA TABLE

CHEMI_12162021_162228_5s_MCF7_TCP_TIMECOURSE_DETYR

Frame: 1
Channel: Membrane
Sensitivity: 100
Molecular Weight Analysis Regression Method : Point to Point

Lane 1 - Ladder

#	Vol. (Int.)	Local Bg. Corr. Vol.	Area	Rf	Density	Local Bg. Corr. Den.	% band purity	% lane purity	Mol. Wt.
1	5,473,873	469,888	176	0.127	31,101	2,669.822	4.585	2.06	198
2	10,846,249	1,145,546	360	0.204	30,128	3,182.073	11.177	4.082	98
3	7,790,151	1,285,430	253	0.304	30,791	5,080.753	12.542	2.932	62
4	11,378,533	1,862,911	368	0.381	30,919	5,062.26	18.177	4.283	49
5	12,089,118	2,079,523	384	0.463	31,482	5,415.426	20.29	4.55	38
6	14,687,050	2,419,578	480	0.548	30,598	5,040.789	23.608	5.528	28
7	12,422,328	985,916	440	0.69	28,232	2,240.718	9.62	4.676	14

Frame: 1
Channel: Chemi
Sensitivity: 100
Molecular Weight Analysis Regression Method : Point to Point

Lane 3 - MCF7_sfGFP_0H

#	Vol. (Int.)	Local Bg. Corr. Vol.	Area	Rf	Density	Local Bg. Corr. Den.	% band purity	% lane purity	Mol. Wt.
1	788,115	621,262	253	0.419	3,115.079	2,455.583	100	15.513	43.923

Lane 4 - MCF7_sfGFP_16H

#	Vol. (Int.)	Local Bg. Corr. Vol.	Area	Rf	Density	Local Bg. Corr. Den.	% band purity	% lane purity	Mol. Wt.
1	1,044,385	849,976	220	0.427	4,747.205	3,863.528	100	19.375	42.795

Lane 5 - MCF7_sfGFP_24H

#	Vol. (Int.)	Local Bg. Corr. Vol.	Area	Rf	Density	Local Bg. Corr. Den.	% band purity	% lane purity	Mol. Wt.
1	971,418	777,981	198	0.431	4,906.152	3,929.201	100	18.331	42.231

Lane 6 - MCF7_sfGFP_48H

#	Vol. (Int.)	Local Bg. Corr. Vol.	Area	Rf	Density	Local Bg. Corr. Den.	% band purity	% lane purity	Mol. Wt.
1	605,681	431,041	189	0.435	3,204.661	2,280.644	100	13.403	41.667

Lane 7 - MCF7_TCPV1_0H

#	Vol. (Int.)	Local Bg. Corr. Vol.	Area	Rf	Density	Local Bg. Corr. Den.	% band purity	% lane purity	Mol. Wt.
1	880,568	657,469	189	0.438	4,659.09	3,478.675	100	16.548	41.385

Lane 8 - MCF7_TCPV1_16H

#	Vol. (Int.)	Local Bg. Corr. Vol.	Area	Rf	Density	Local Bg. Corr. Den.	% band purity	% lane purity	Mol. Wt.
1	1,138,922	274,765	242	0.421	4,706.289	1,135.395	19.988	13.884	43.641
2	1,672,173	1,099,906	220	0.44	7,600.786	4,999.573	80.012	20.384	41.103

Lane 9 - MCF7_TCPV1_24H

#	Vol. (Int.)	Local Bg. Corr. Vol.	Area	Rf	Density	Local Bg. Corr. Den.	% band purity	% lane purity	Mol. Wt.
1	3,530,269	2,395,304	147	0.417	24,015	16,294	35.807	18.519	44.205
2	6,996,008	4,294,090	315	0.438	22,209	13,632	64.193	36.699	41.385

Lane 10 - MCF7_TCPV1_48H

#	Vol. (Int.)	Local Bg. Corr. Vol.	Area	Rf	Density	Local Bg. Corr. Den.	% band purity	% lane purity	Mol. Wt.
1	26,862,142	24,059,481	528	0.429	50,875	45,567	100	62.448	42.513

Lane 11 - MCF7_TCPV2_0H

#	Vol. (Int.)	Local Bg. Corr. Vol.	Area	Rf	Density	Local Bg. Corr. Den.	% band purity	% lane purity	Mol. Wt.
1	1,232,068	642,536	207	0.44	5,952.019	3,104.04	100	13.822	41.103

Lane 12 - MCF7_TCPV2_16H

#	Vol. (Int.)	Local Bg. Corr. Vol.	Area	Rf	Density	Local Bg. Corr. Den.	% band purity	% lane purity	Mol. Wt.
1	1,937,132	1,062,163	161	0.412	12,031	6,597.288	28.016	11.941	44.769
2	4,677,398	2,729,178	345	0.433	13,557	7,910.661	71.984	28.833	41.949

Lane 13 - MCF7_TCPV2_24H

#	Vol. (Int.)	Local Bg. Corr. Vol.	Area	Rf	Density	Local Bg. Corr. Den.	% band purity	% lane purity	Mol. Wt.
1	24,755,008	22,288,702	506	0.423	48,922	44,048	100	57.235	43.359

Lane 14 - MCF7_TCPV2_48H

#	Vol. (Int.)	Local Bg. Corr. Vol.	Area	Rf	Density	Local Bg. Corr. Den.	% band purity	% lane purity	Mol. Wt.
1	32,534,728	29,708,217	676	0.417	48,128	43,947	100	50.064	44.205

iBright™ Image Analysis Report

28 January 2022

Figure 1D- Alpha Tubulin

CHEMI_12162021_161715_24s_189ms_MCF7_TCP_TIMECOURSE_ALPHA

Date:	12-16-2021 04:17:15PM
Mode:	Chemi Blots
Notes:	Figure 1D- alpha tubulin
Model:	FL1500
Instrument name:	2462619090234
Serial No:	2462619090234
Firmware version:	1.6.0
iBA version:	4.0.1
Image size:	615px X 491px
Image area:	
Optical Zoom:	2x
Digital Zoom:	1.1x
Focus level:	455
Resolution:	5 x 5
Exposure time:	24189 ms
Exposure mode:	Normal

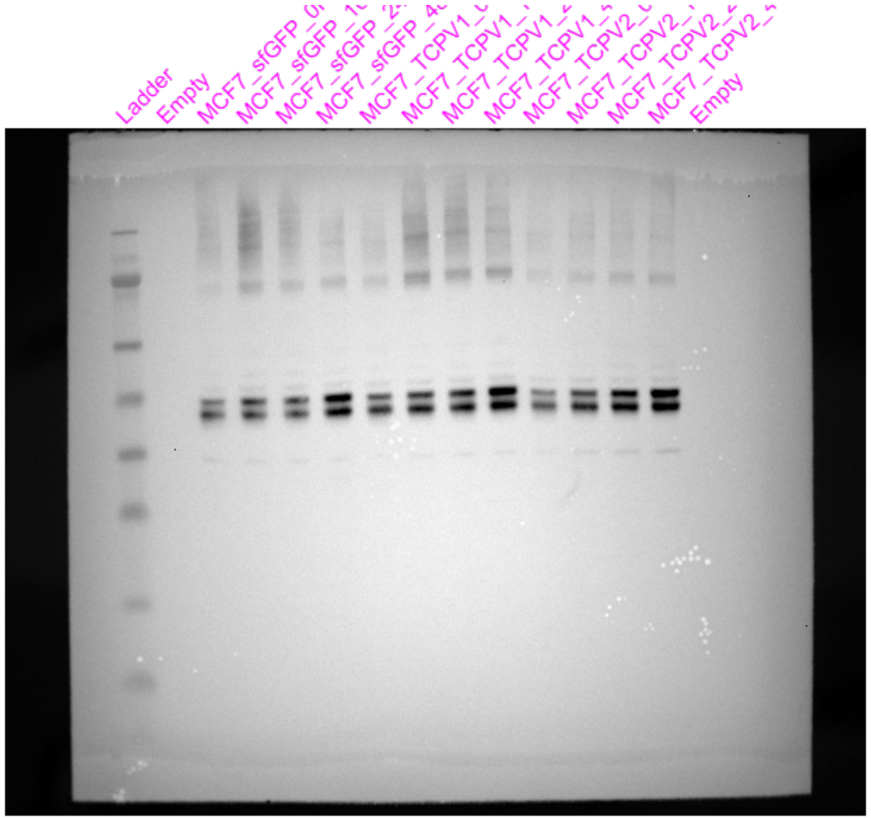

CHEMI_12162021_161715_24s_189ms_MCF7_TCP_TIMECOURSE_ALPHA

Date:	12-16-2021 04:17:15PM
Mode:	Chemi Blots
Notes:	Figure 1D- alpha tubulin
Model:	FL1500
Instrument name:	2462619090234
Serial No:	2462619090234
Firmware version:	1.6.0
iBA version:	4.0.1
Image size:	615px X 491px
Image area:	
Optical Zoom:	2x
Digital Zoom:	1.1x
Focus level:	455
Resolution:	5 x 5
Exposure time:	24189 ms
Exposure mode:	Normal

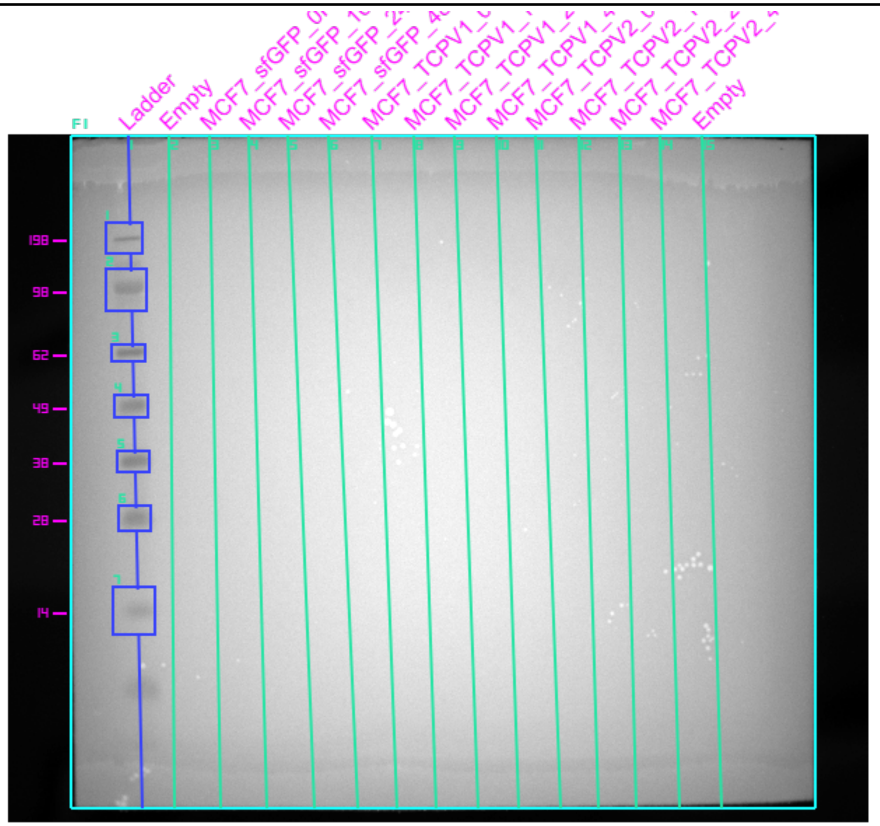

CHEMI_12162021_161715_24s_189ms_MCF7_TCP_TIMECOURSE_ALPHA

Date:	12-16-2021 04:17:15PM
Mode:	Chemi Blots
Notes:	Figure 1D- alpha tubulin
Model:	FL1500
Instrument name:	2462619090234
Serial No:	2462619090234
Firmware version:	1.6.0
iBA version:	4.0.1
Image size:	615px X 491px
Image area:	
Optical Zoom:	2x
Digital Zoom:	1.1x
Focus level:	455
Resolution:	5 x 5
Exposure time:	24189 ms
Exposure mode:	Normal

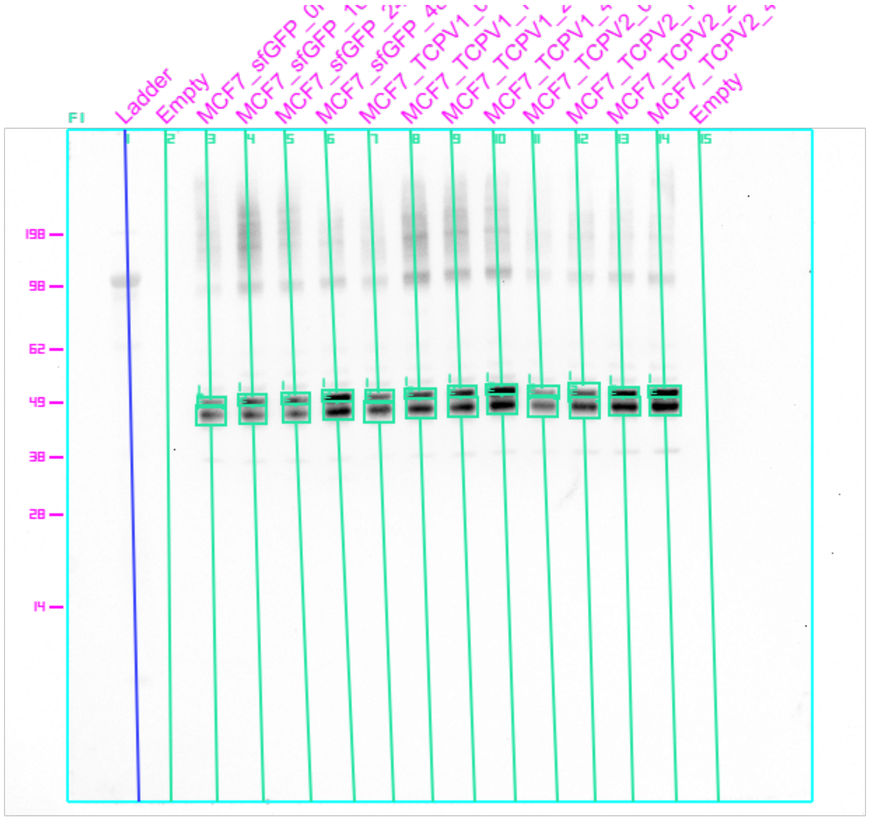

LANE AND BAND ANALYSIS DATA TABLE

CHEMI_12162021_161715_24s_189ms_MCF7_TCP_TIMECOURSE_ALPHA

Frame: 1
Channel: Membrane
Sensitivity: 50
Molecular Weight Analysis Regression Method : Point to Point

Lane 1 - Ladder

#	Vol. (Int.)	Local Bg. Corr. Vol.	Area	Rf	Density	Local Bg. Corr. Den.	% band purity	% lane purity	Mol. Wt.
1	20,412,526	612,964	621	0.152	32,870	987.06	6.24	6.905	198
2	30,412,619	1,699,212	930	0.229	32,701	1,827.11	17.298	10.288	98
3	11,005,014	1,283,737	325	0.323	33,861	3,949.961	13.068	3.723	62
4	14,084,972	1,603,013	425	0.402	33,141	3,771.797	16.319	4.765	49
5	13,003,250	1,683,634	384	0.483	33,862	4,384.464	17.139	4.399	38
6	15,107,840	1,718,470	456	0.569	33,131	3,768.576	17.494	5.111	28
7	33,383,180	1,222,123	1,085	0.706	30,767	1,126.381	12.441	11.293	14

Frame: 1
Channel: Chemi
Sensitivity: 50
Molecular Weight Analysis Regression Method : Point to Point

Lane 3 - MCF7_sfGFP_0H

#	Vol. (Int.)	Local Bg. Corr. Vol.	Area	Rf	Density	Local Bg. Corr. Den.	% band purity	% lane purity	Mol. Wt.
1	1,910,562	929,064	189	0.406	10,108	4,915.685	28.48	14.217	48.436
2	3,545,915	2,333,077	330	0.425	10,745	7,069.933	71.52	26.386	45.897

Lane 4 - MCF7_sfGFP_16H

#	Vol. (Int.)	Local Bg. Corr. Vol.	Area	Rf	Density	Local Bg. Corr. Den.	% band purity	% lane purity	Mol. Wt.
1	2,583,444	1,618,681	189	0.402	13,669	8,564.452	44.016	12.499	49
2	3,661,818	2,058,806	280	0.423	13,077	7,352.881	55.984	17.717	46.179

Lane 5 - MCF7_sfGFP_24H

#	Vol. (Int.)	Local Bg. Corr. Vol.	Area	Rf	Density	Local Bg. Corr. Den.	% band purity	% lane purity	Mol. Wt.

#	Vol. (Int.)	Local Bg. Corr. Vol.	Area	Rf	Density	Local Bg. Corr. Den.	% band purity	% lane purity	Mol. Wt.
1	2,324,263	1,493,728	189	0.4	12,297	7,903.325	36.303	14.772	49.342
2	3,343,923	1,892,696	280	0.421	11,942	6,759.629	45.999	21.253	46.462

Lane 6 - MCF7_sfGFP_48H

#	Vol. (Int.)	Local Bg. Corr. Vol.	Area	Rf	Density	Local Bg. Corr. Den.	% band purity	% lane purity	Mol. Wt.
1	4,335,141	3,127,712	207	0.396	20,942	15,109	49.452	24.585	50.026
2	5,730,923	3,197,074	308	0.417	18,606	10,380	50.548	32.501	47.026

Lane 7 - MCF7_TCPV1_0H

#	Vol. (Int.)	Local Bg. Corr. Vol.	Area	Rf	Density	Local Bg. Corr. Den.	% band purity	% lane purity	Mol. Wt.
1	2,450,284	1,279,450	210	0.396	11,668	6,092.62	27.232	17.443	50.026
2	4,189,864	2,742,323	352	0.419	11,903	7,790.692	58.369	29.826	46.744

Lane 8 - MCF7_TCPV1_16H

#	Vol. (Int.)	Local Bg. Corr. Vol.	Area	Rf	Density	Local Bg. Corr. Den.	% band purity	% lane purity	Mol. Wt.
1	2,743,865	1,787,966	184	0.392	14,912	9,717.21	36.5	12.588	50.711
2	4,980,937	3,110,512	330	0.415	15,093	9,425.797	63.5	22.852	47.308

Lane 9 - MCF7_TCPV1_24H

#	Vol. (Int.)	Local Bg. Corr. Vol.	Area	Rf	Density	Local Bg. Corr. Den.	% band purity	% lane purity	Mol. Wt.
1	3,143,741	2,051,249	189	0.39	16,633	10,853	40.874	15.166	51.053
2	5,058,138	2,967,203	330	0.412	15,327	8,991.526	59.126	24.402	47.59

Lane 10 - MCF7_TCPV1_48H

#	Vol. (Int.)	Local Bg. Corr. Vol.	Area	Rf	Density	Local Bg. Corr. Den.	% band purity	% lane purity	Mol. Wt.
1	5,004,589	3,618,055	184	0.385	27,198	19,663	49.888	22.773	51.737
2	6,767,852	3,634,345	330	0.408	20,508	11,013	50.112	30.797	48.154

Lane 11 - MCF7_TCPV2_0H

#	Vol. (Int.)	Local Bg. Corr. Vol.	Area	Rf	Density	Local Bg. Corr. Den.	% band purity	% lane purity	Mol. Wt.
1	2,009,135	938,708	230	0.39	8,735.37	4,081.342	27.912	17.213	51.053

#	Vol. (Int.)	Local Bg. Corr. Vol.	Area	Rf	Density	Local Bg. Corr. Den.	% band purity	% lane purity	Mol. Wt.
2	3,762,225	2,424,432	352	0.41	10,688	6,887.592	72.088	32.232	47.872

Lane 12 - MCF7_TCPV2_16H

#	Vol. (Int.)	Local Bg. Corr. Vol.	Area	Rf	Density	Local Bg. Corr. Den.	% band purity	% lane purity	Mol. Wt.
1	3,617,689	1,828,686	322	0.39	11,235	5,679.15	36.851	25.574	51.053
2	4,916,093	3,133,640	352	0.412	13,966	8,902.388	63.149	34.753	47.59

Lane 13 - MCF7_TCPV2_24H

#	Vol. (Int.)	Local Bg. Corr. Vol.	Area	Rf	Density	Local Bg. Corr. Den.	% band purity	% lane purity	Mol. Wt.
1	3,641,887	2,513,361	176	0.39	20,692	14,280	37.56	23.062	51.053
2	5,775,311	3,517,006	322	0.41	17,935	10,922	52.559	36.572	47.872

Lane 14 - MCF7_TCPV2_48H

#	Vol. (Int.)	Local Bg. Corr. Vol.	Area	Rf	Density	Local Bg. Corr. Den.	% band purity	% lane purity	Mol. Wt.
1	5,357,280	3,944,440	216	0.39	24,802	18,261	46.323	29.729	51.053
2	6,706,778	3,880,626	322	0.41	20,828	12,051	45.573	37.217	47.872

iBright™ Image Analysis Report

28 January 2022

Figure 1D- FLAG

CHEMI_12162021_161353_98ms_MCF7_TCP _TIMECOURSE_FLAG

Date:	12-16-2021 04:13:53PM
Mode:	Chemi Blots
Notes:	Figure 1D-FLAG
Model:	FL1500
Instrument name:	2462619090234
Serial No:	2462619090234
Firmware version:	1.6.0
iBA version:	4.0.1
Image size:	615px X 491px
Image area:	
Optical Zoom:	2x
Digital Zoom:	1.1x
Focus level:	455
Resolution:	5 x 5
Exposure time:	98 ms
Exposure mode:	Normal

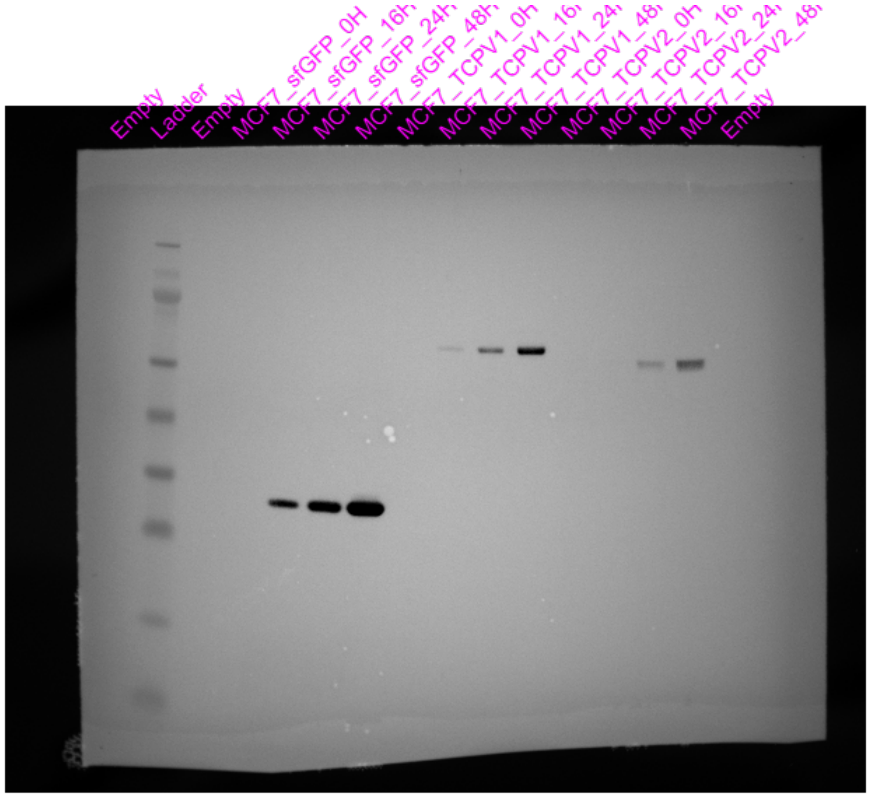

CHEMI_12162021_161353_98ms_MCF7_TCP _TIMECOURSE_FLAG

Date:	12-16-2021 04:13:53PM
Mode:	Chemi Blots
Notes:	Figure 1D-FLAG
Model:	FL1500
Instrument name:	2462619090234
Serial No:	2462619090234
Firmware version:	1.6.0
iBA version:	4.0.1
Image size:	615px X 491px
Image area:	
Optical Zoom:	2x
Digital Zoom:	1.1x
Focus level:	455
Resolution:	5 x 5
Exposure time:	98 ms
Exposure mode:	Normal

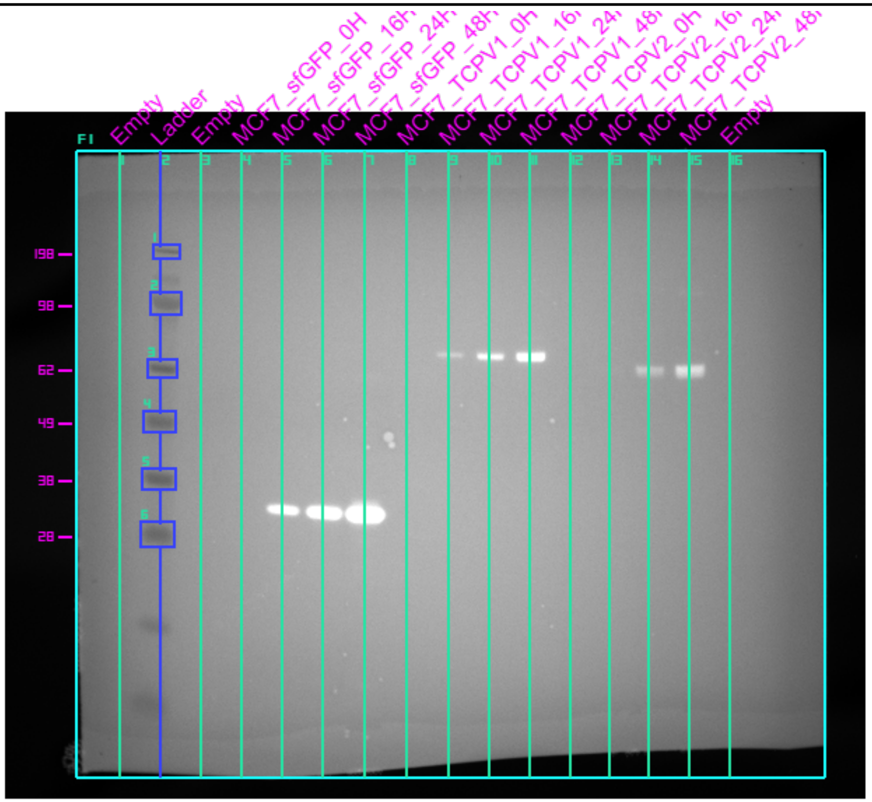

CHEMI_12162021_161353_98ms_MCF7_TCP_TIMECOURSE_FLAG

Date:	12-16-2021 04:13:53PM
Mode:	Chemi Blots
Notes:	Figure 1D-FLAG
Model:	FL1500
Instrument name:	2462619090234
Serial No:	2462619090234
Firmware version:	1.6.0
iBA version:	4.0.1
Image size:	615px X 491px
Image area:	
Optical Zoom:	2x
Digital Zoom:	1.1x
Focus level:	455
Resolution:	5 x 5
Exposure time:	98 ms
Exposure mode:	Normal

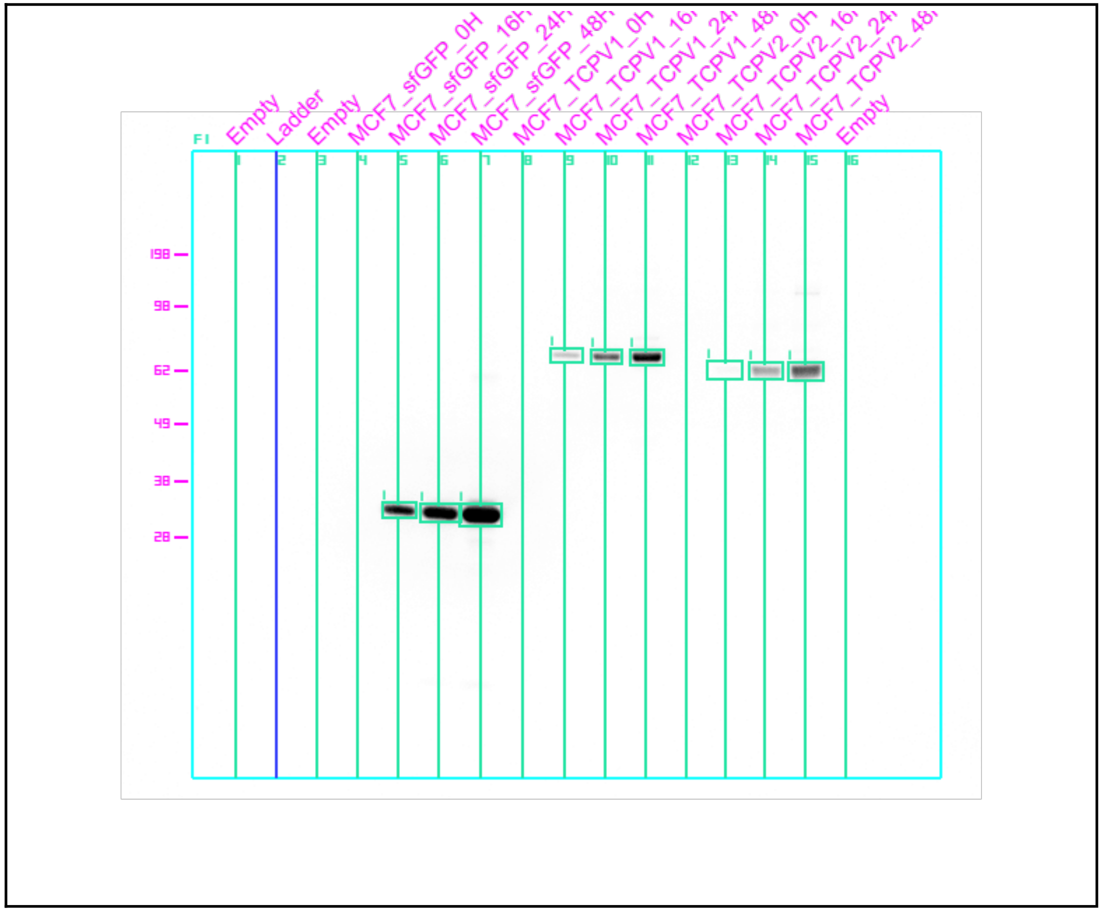

LANE AND BAND ANALYSIS DATA TABLE

CHEMI_12162021_161353_98ms_MCF7_TCP_TIMECOURSE_FLAG

Frame: 1
Channel: Membrane
Sensitivity: 50
Molecular Weight Analysis Regression Method : Point to Point

Lane 2 - Ladder

#	Vol. (Int.)	Local Bg. Corr. Vol.	Area	Rf	Density	Local Bg. Corr. Den.	% band purity	% lane purity	Mol. Wt.
1	6,940,246	486,947	220	0.161	31,546	2,213.396	5.382	2.283	198
2	12,594,643	1,379,580	391	0.243	32,211	3,528.34	15.247	4.143	98
3	10,139,475	1,361,758	308	0.346	32,920	4,421.294	15.05	3.335	62
4	12,861,629	1,684,607	384	0.431	33,493	4,386.998	18.618	4.231	49
5	13,449,052	2,003,983	400	0.522	33,622	5,009.96	22.148	4.424	38
6	15,924,638	2,131,344	475	0.612	33,525	4,487.041	23.555	5.239	28

Frame: 1
Channel: Chemi
Sensitivity: 50
Molecular Weight Analysis Regression Method : Point to Point

Lane 5 - MCF7_sfGFP_16H

#	Vol. (Int.)	Local Bg. Corr. Vol.	Area	Rf	Density	Local Bg. Corr. Den.	% band purity	% lane purity	Mol. Wt.
1	4,908,257	4,354,568	300	0.571	16,360	14,515	100	78.601	32.5

Lane 6 - MCF7_sfGFP_24H

#	Vol. (Int.)	Local Bg. Corr. Vol.	Area	Rf	Density	Local Bg. Corr. Den.	% band purity	% lane purity	Mol. Wt.
1	8,749,152	6,570,941	420	0.576	20,831	15,645	100	90.917	32

Lane 7 - MCF7_sfGFP_48H

#	Vol. (Int.)	Local Bg. Corr. Vol.	Area	Rf	Density	Local Bg. Corr. Den.	% band purity	% lane purity	Mol. Wt.
1	13,283,288	11,252,444	527	0.58	25,205	21,351	100	94.896	31.5

Lane 9 - MCF7_TCPV1_16H

#	Vol. (Int.)	Local Bg. Corr. Vol.	Area	Rf	Density	Local Bg. Corr. Den.	% band purity	% lane purity	Mol. Wt.
1	713,708	618,637	264	0.326	2,703.439	2,343.322	100	36.476	69.043

Lane 10 - MCF7_TCPV1_24H

#	Vol. (Int.)	Local Bg. Corr. Vol.	Area	Rf	Density	Local Bg. Corr. Den.	% band purity	% lane purity	Mol. Wt.
1	2,270,597	2,091,134	253	0.328	8,974.692	8,265.355	100	64.786	68.261

Lane 11 - MCF7_TCPV1_48H

#	Vol. (Int.)	Local Bg. Corr. Vol.	Area	Rf	Density	Local Bg. Corr. Den.	% band purity	% lane purity	Mol. Wt.
1	4,691,552	4,415,086	300	0.328	15,638	14,716	100	78.97	68.261

Lane 13 - MCF7_TCPV2_16H

#	Vol. (Int.)	Local Bg. Corr. Vol.	Area	Rf	Density	Local Bg. Corr. Den.	% band purity	% lane purity	Mol. Wt.
1	196,116	100,110	364	0.348	538.78	275.03	100	20.212	61.658

Lane 14 - MCF7_TCPV2_24H

#	Vol. (Int.)	Local Bg. Corr. Vol.	Area	Rf	Density	Local Bg. Corr. Den.	% band purity	% lane purity	Mol. Wt.
1	1,789,868	1,600,457	325	0.35	5,507.286	4,924.486	100	65.625	61.316

Lane 15 - MCF7_TCPV2_48H

#	Vol. (Int.)	Local Bg. Corr. Vol.	Area	Rf	Density	Local Bg. Corr. Den.	% band purity	% lane purity	Mol. Wt.
1	4,081,467	3,852,442	364	0.35	11,212	10,583	100	78.745	61.316

iBright™ Image Analysis Report

28 January 2022

Figure 1D- GAPDH

CHEMI_12172021_162518_445ms_MCF7_TCP_TIMECOURSE_GAPDH

Date:	12-17-2021 04:25:18PM
Mode:	Chemi Blots
Notes:	Fig1D_GAPDH
Model:	FL1500
Instrument name:	2462619090234
Serial No:	2462619090234
Firmware version:	1.6.0
iBA version:	4.0.1
Image size:	676px X 540px
Image area:	
Optical Zoom:	2x
Digital Zoom:	1x
Focus level:	455
Resolution:	5 x 5
Exposure time:	445 ms
Exposure mode:	Normal

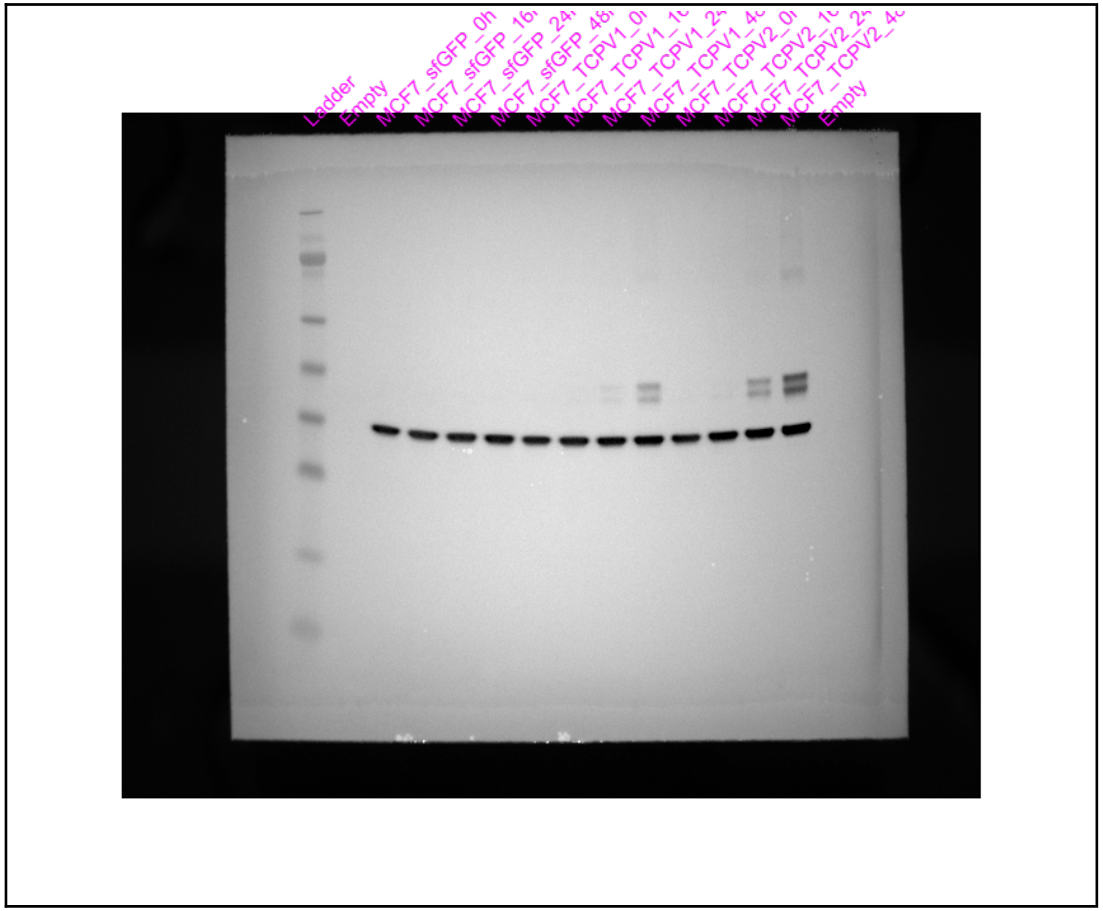

CHEMI_12172021_162518_445ms_MCF7_TCP_TIMECOURSE_GAPDH

Date:	12-17-2021 04:25:18PM
Mode:	Chemi Blots
Notes:	Fig1D_GAPDH
Model:	FL1500
Instrument name:	2462619090234
Serial No:	2462619090234
Firmware version:	1.6.0
iBA version:	4.0.1
Image size:	676px X 540px
Image area:	
Optical Zoom:	2x
Digital Zoom:	1x
Focus level:	455
Resolution:	5 x 5
Exposure time:	445 ms
Exposure mode:	Normal

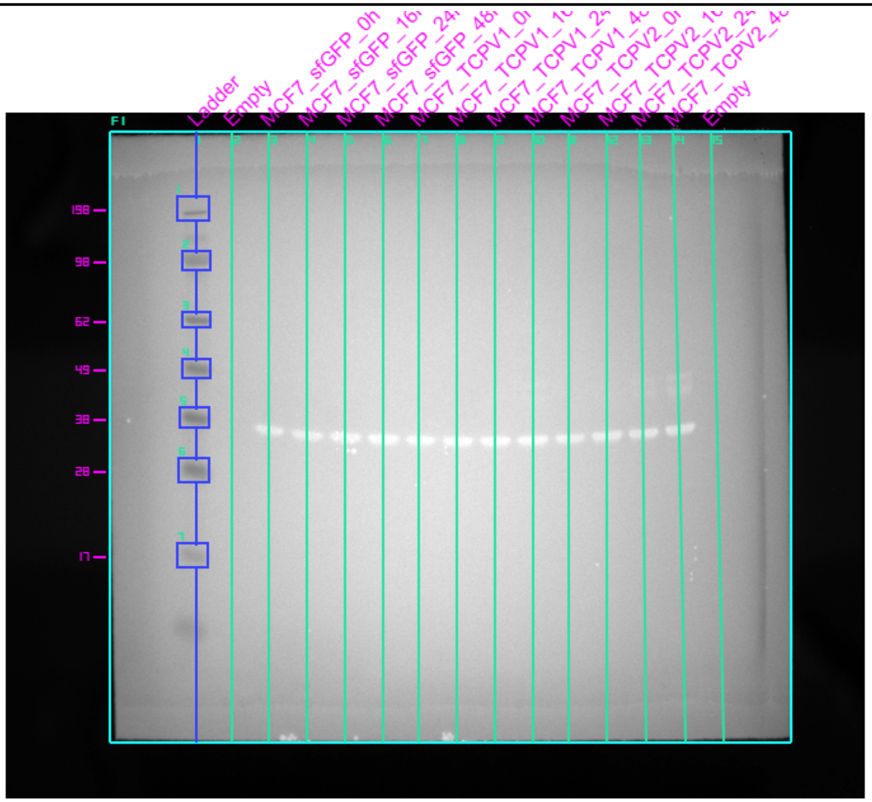

CHEMI_12172021_162518_445ms_MCF7_TCP_TIMECOURSE_GAPDH

Date:	12-17-2021 04:25:18PM
Mode:	Chemi Blots
Notes:	Fig1D_GAPDH
Model:	FL1500
Instrument name:	2462619090234
Serial No:	2462619090234
Firmware version:	1.6.0
iBA version:	4.0.1
Image size:	676px X 540px
Image area:	
Optical Zoom:	2x
Digital Zoom:	1x
Focus level:	455
Resolution:	5 x 5
Exposure time:	445 ms
Exposure mode:	Normal

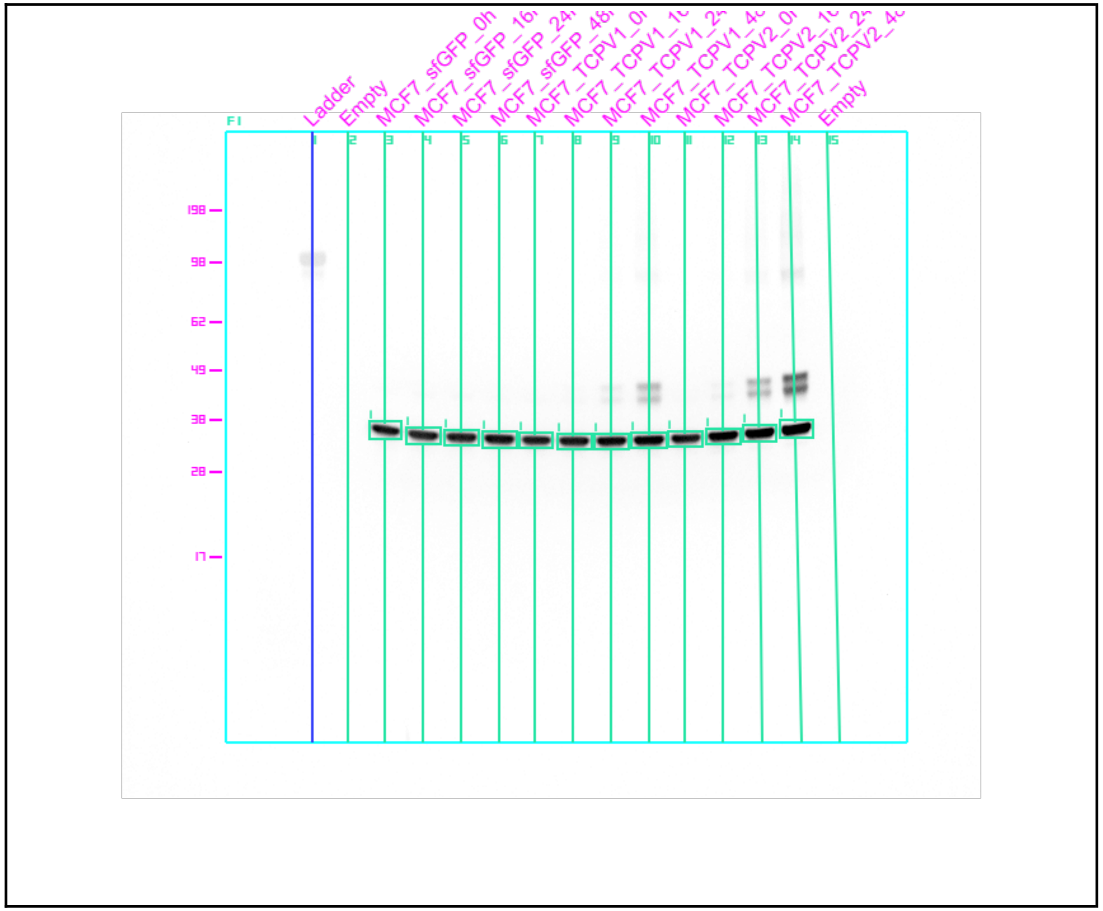

LANE AND BAND ANALYSIS DATA TABLE

CHEMI_12172021_162518_445ms_MCF7_TCP_TIMECOURSE_GAPDH

Frame: 1
Channel: Membrane
Sensitivity: 50
Molecular Weight Analysis Regression Method : Point to Point

Lane 1 - Ladder

#	Vol. (Int.)	Local Bg. Corr. Vol.	Area	Rf	Density	Local Bg. Corr. Den.	% band purity	% lane purity	Mol. Wt.
1	15,879,782	568,869	520	0.125	30,538	1,093.981	5.592	4.731	198
2	11,584,110	1,270,751	368	0.21	31,478	3,453.128	12.492	3.452	98
3	9,356,520	1,298,444	299	0.308	31,292	4,342.622	12.764	2.788	62
4	11,712,337	1,734,639	368	0.387	31,827	4,713.694	17.052	3.49	49
5	13,105,894	1,982,711	408	0.468	32,122	4,859.587	19.491	3.905	38
6	15,735,295	2,310,695	500	0.553	31,470	4,621.391	22.715	4.688	28
7	14,630,759	1,006,432	500	0.692	29,261	2,012.865	9.894	4.359	17

Frame: 1
Channel: Chemi
Sensitivity: 50
Molecular Weight Analysis Regression Method : Point to Point

Lane 3 - MCF7_sfGFP_0h

#	Vol. (Int.)	Local Bg. Corr. Vol.	Area	Rf	Density	Local Bg. Corr. Den.	% band purity	% lane purity	Mol. Wt.
1	5,249,067	4,729,797	390	0.489	13,459	12,127	100	75.919	35.561

Lane 4 - MCF7_sfGFP_16H

#	Vol. (Int.)	Local Bg. Corr. Vol.	Area	Rf	Density	Local Bg. Corr. Den.	% band purity	% lane purity	Mol. Wt.
1	5,486,588	4,681,035	392	0.497	13,996	11,941	100	73.625	34.585

Lane 5 - MCF7_sfGFP_24H

#	Vol. (Int.)	Local Bg. Corr. Vol.	Area	Rf	Density	Local Bg. Corr. Den.	% band purity	% lane purity	Mol. Wt.
1	5,683,962	4,836,823	364	0.501	15,615	13,287	100	72.308	34.098

Lane 6 - MCF7_sfGFP_48H

#	Vol. (Int.)	Local Bg. Corr. Vol.	Area	Rf	Density	Local Bg. Corr. Den.	% band purity	% lane purity	Mol. Wt.
1	6,191,589	5,254,325	392	0.503	15,794	13,403	100	73.909	33.854

Lane 7 - MCF7_TCPV1_0H

#	Vol. (Int.)	Local Bg. Corr. Vol.	Area	Rf	Density	Local Bg. Corr. Den.	% band purity	% lane purity	Mol. Wt.
1	5,321,537	4,430,612	351	0.505	15,161	12,622	100	68.779	33.61

Lane 8 - MCF7_TCPV1_16H

#	Vol. (Int.)	Local Bg. Corr. Vol.	Area	Rf	Density	Local Bg. Corr. Den.	% band purity	% lane purity	Mol. Wt.
1	5,871,468	4,822,293	364	0.507	16,130	13,248	100	69.631	33.366

Lane 9 - MCF7_TCPV1_24H

#	Vol. (Int.)	Local Bg. Corr. Vol.	Area	Rf	Density	Local Bg. Corr. Den.	% band purity	% lane purity	Mol. Wt.
1	6,191,719	5,062,349	364	0.507	17,010	13,907	100	66.388	33.366

Lane 10 - MCF7_TCPV1_48H

#	Vol. (Int.)	Local Bg. Corr. Vol.	Area	Rf	Density	Local Bg. Corr. Den.	% band purity	% lane purity	Mol. Wt.
1	6,790,756	5,530,299	377	0.505	18,012	14,669	100	56.497	33.61

Lane 11 - MCF7_TCPV2_0H

#	Vol. (Int.)	Local Bg. Corr. Vol.	Area	Rf	Density	Local Bg. Corr. Den.	% band purity	% lane purity	Mol. Wt.
1	5,654,492	4,523,141	364	0.503	15,534	12,426	100	68.491	33.854

Lane 12 - MCF7_TCPV2_16H

#	Vol. (Int.)	Local Bg. Corr. Vol.	Area	Rf	Density	Local Bg. Corr. Den.	% band purity	% lane purity	Mol. Wt.
1	6,767,433	5,621,006	364	0.499	18,591	15,442	100	69.686	34.341

Lane 13 - MCF7_TCPV2_24H

#	Vol. (Int.)	Local Bg. Corr. Vol.	Area	Rf	Density	Local Bg. Corr. Den.	% band purity	% lane purity	Mol. Wt.
1	7,271,636	6,317,973	378	0.493	19,237	16,714	100	58.252	35.073

Lane 14 - MCF7_TCPV2_48H

#	Vol. (Int.)	Local Bg. Corr. Vol.	Area	Rf	Density	Local Bg. Corr. Den.	% band purity	% lane purity	Mol. Wt.
1	8,336,539	7,846,617	405	0.486	20,584	19,374	100	45.751	35.805

iBright™ Image Analysis Report

28 February 2022

Supplemental Figure 2- Tyr-Tub for MCF7s

CHEMI_02252022_145830_6s_MCFF7_TCP_TIMECOURSE_TYRTUB

Date:	02-25-2022 02:58:30PM
Mode:	Chemi Blots
Notes:	
Model:	FL1500
Instrument name:	2462619090234
Serial No:	2462619090234
Firmware version:	1.6.0
iBA version:	4.0.1
Image size:	563px X 450px
Image area:	
Optical Zoom:	2x
Digital Zoom:	1.2x
Focus level:	455
Resolution:	5 x 5
Exposure time:	6000 ms
Exposure mode:	Normal

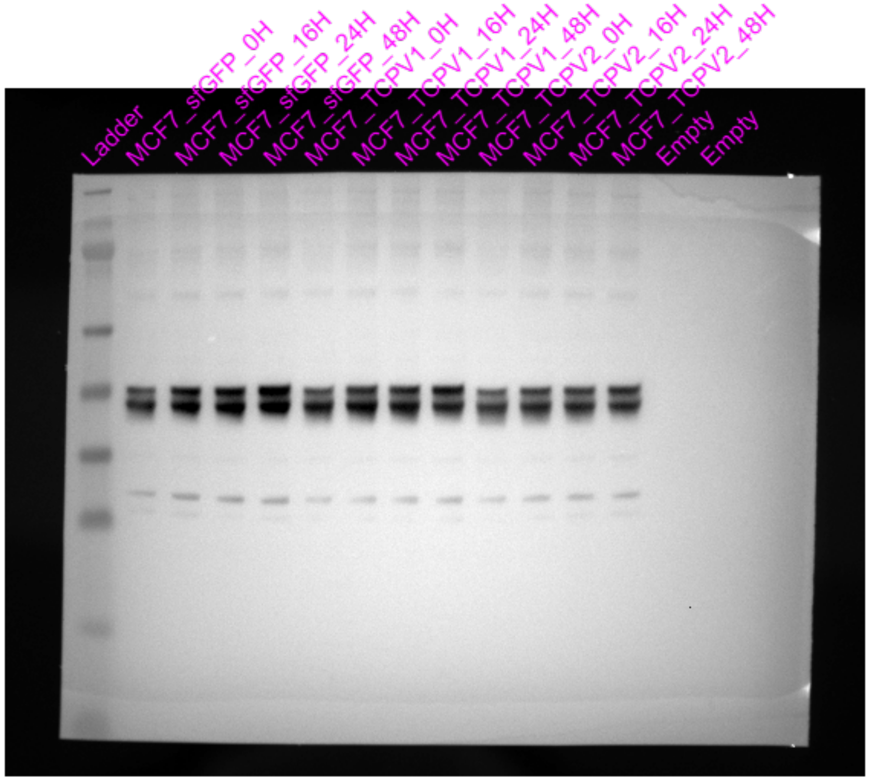

CHEMI_02252022_145830_6s_MCFF7_TCP_TIMECOURSE_TYRTUB

Date:	02-25-2022 02:58:30PM
Mode:	Chemi Blots
Notes:	
Model:	FL1500
Instrument name:	2462619090234
Serial No:	2462619090234
Firmware version:	1.6.0
iBA version:	4.0.1
Image size:	563px X 450px
Image area:	
Optical Zoom:	2x
Digital Zoom:	1.2x
Focus level:	455
Resolution:	5 x 5
Exposure time:	6000 ms
Exposure mode:	Normal

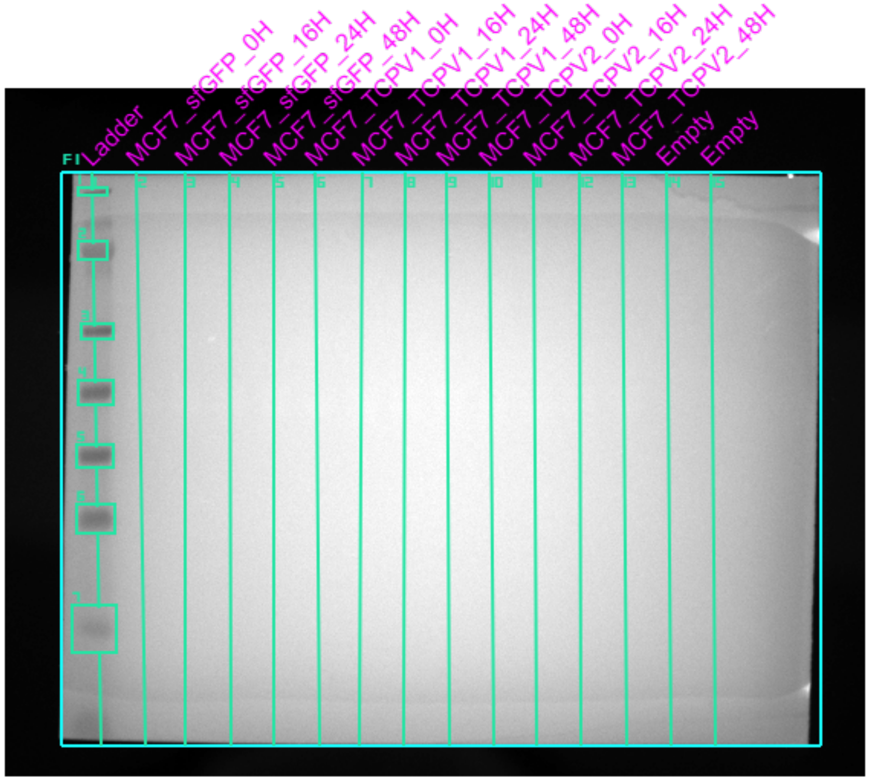

CHEMI_02252022_145830_6s_MCFF7_TCP_TIMECOURSE_TYRTUB

Date:	02-25-2022 02:58:30PM
Mode:	Chemi Blots
Notes:	
Model:	FL1500
Instrument name:	2462619090234
Serial No:	2462619090234
Firmware version:	1.6.0
iBA version:	4.0.1
Image size:	563px X 450px
Image area:	
Optical Zoom:	2x
Digital Zoom:	1.2x
Focus level:	455
Resolution:	5 x 5
Exposure time:	6000 ms
Exposure mode:	Normal

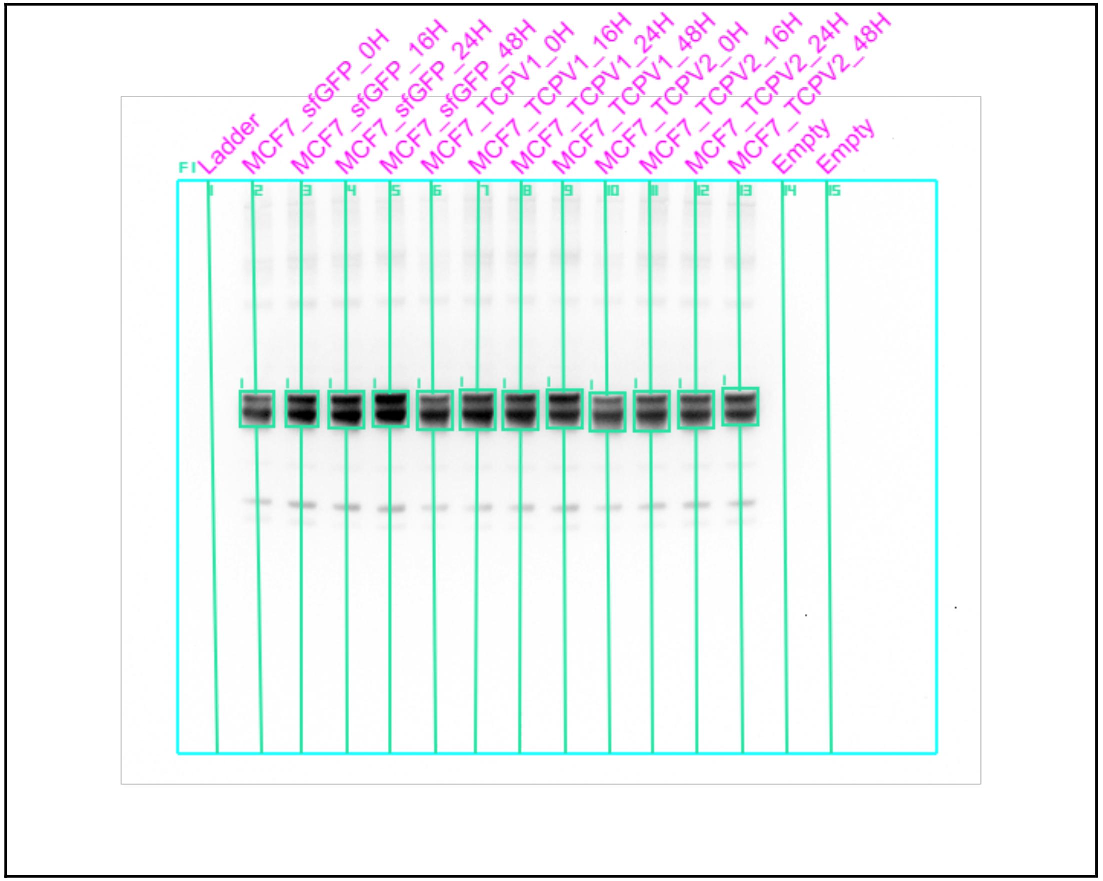

LANE AND BAND ANALYSIS DATA TABLE

CHEMI_02252022_145830_6s_MCFF7_TCP_TIMECOURSE_TYRTUB

Frame: 1
Channel: Membrane
Sensitivity: 100

Lane 1 - Ladder

#	Vol. (Int.)	Local Bg. Corr. Vol.	Area	Rf	Density	Local Bg. Corr. Den.	% band purity	% lane purity
1	4,768,905	215,038	120	0.032	39,740	1,791.985	1.954	1.795
2	10,319,503	699,837	260	0.136	39,690	2,691.684	6.358	3.885
3	9,617,800	1,429,359	242	0.277	39,742	5,906.442	12.986	3.621
4	15,964,590	2,261,545	408	0.384	39,128	5,543.003	20.547	6.01
5	15,836,857	2,320,601	400	0.493	39,592	5,801.504	21.083	5.962
6	20,037,706	2,573,640	520	0.603	38,534	4,949.308	23.382	7.543
7	34,739,180	1,506,773	960	0.795	36,186	1,569.555	13.689	13.077

Frame: 1
Channel: Chemi
Sensitivity: 100

Lane 2 - MCF7_sfGFP_0H

#	Vol. (Int.)	Local Bg. Corr. Vol.	Area	Rf	Density	Local Bg. Corr. Den.	% band purity	% lane purity
1	12,618,797	10,748,928	552	0.397	22,860	19,472	100	64.9

Lane 3 - MCF7_sfGFP_16H

#	Vol. (Int.)	Local Bg. Corr. Vol.	Area	Rf	Density	Local Bg. Corr. Den.	% band purity	% lane purity
1	16,510,347	13,658,941	528	0.397	31,269	25,869	100	61.218

Lane 4 - MCF7_sfGFP_24H

#	Vol. (Int.)	Local Bg. Corr. Vol.	Area	Rf	Density	Local Bg. Corr. Den.	% band purity	% lane purity
1	18,608,061	15,025,511	600	0.4	31,013	25,042	100	65.696

Lane 5 - MCF7_sfGFP_48H

#	Vol. (Int.)	Local Bg. Corr. Vol.	Area	Rf	Density	Local Bg. Corr. Den.	% band purity	% lane purity
1	20,408,765	16,718,702	576	0.397	35,431	29,025	100	68.439

Lane 6 - MCF7_TCPV1_0H

#	Vol. (Int.)	Local Bg. Corr. Vol.	Area	Rf	Density	Local Bg. Corr. Den.	% band purity	% lane purity
1	15,673,886	12,121,076	624	0.4	25,118	19,424	100	67.249

Lane 7 - MCF7_TCPV1_16H

#	Vol. (Int.)	Local Bg. Corr. Vol.	Area	Rf	Density	Local Bg. Corr. Den.	% band purity	% lane purity
1	18,390,139	14,671,280	648	0.4	28,379	22,640	100	66.7

Lane 8 - MCF7_TCPV1_24H

#	Vol. (Int.)	Local Bg. Corr. Vol.	Area	Rf	Density	Local Bg. Corr. Den.	% band purity	% lane purity
1	17,738,353	13,978,361	624	0.4	28,426	22,401	100	65.313

Lane 9 - MCF7_TCPV1_48H

#	Vol. (Int.)	Local Bg. Corr. Vol.	Area	Rf	Density	Local Bg. Corr. Den.	% band purity	% lane purity
1	17,527,486	13,978,462	624	0.397	28,088	22,401	100	67.15

Lane 10 - MCF7_TCPV2_0H

#	Vol. (Int.)	Local Bg. Corr. Vol.	Area	Rf	Density	Local Bg. Corr. Den.	% band purity	% lane purity
1	14,229,471	11,033,826	624	0.403	22,803	17,682	100	67.448

Lane 11 - MCF7_TCPV2_16H

#	Vol. (Int.)	Local Bg. Corr. Vol.	Area	Rf	Density	Local Bg. Corr. Den.	% band purity	% lane purity
1	15,855,736	12,729,340	648	0.403	24,468	19,644	100	65.357

Lane 12 - MCF7_TCPV2_24H

#	Vol. (Int.)	Local Bg. Corr. Vol.	Area	Rf	Density	Local Bg. Corr. Den.	% band purity	% lane purity
1	15,064,419	12,035,671	600	0.4	25,107	20,059	100	64.465

Lane 13 - MCF7_TCPV2_48H

#	Vol. (Int.)	Local Bg. Corr. Vol.	Area	Rf	Density	Local Bg. Corr. Den.	% band purity	% lane purity
1	14,759,527	12,343,132	600	0.395	24,599	20,571	100	67.458

iBright™ Image Analysis Report

28 February 2022

Supplemental Figure 2- GADH for Tyr-Tub (MCF7s)

CHEMI_02272022_185805_1s_750ms_MCF7 _TCP_TIMECOURSE_TYRTUB_GAPDH

Date:	02-27-2022 06:58:05PM
Mode:	Chemi Blots
Notes:	
Model:	FL1500
Instrument name:	2462619090234
Serial No:	2462619090234
Firmware version:	1.6.0
iBA version:	4.0.1
Image size:	676px X 540px
Image area:	
Optical Zoom:	2x
Digital Zoom:	1x
Focus level:	455
Resolution:	5 x 5
Exposure time:	1750 ms
Exposure mode:	Normal

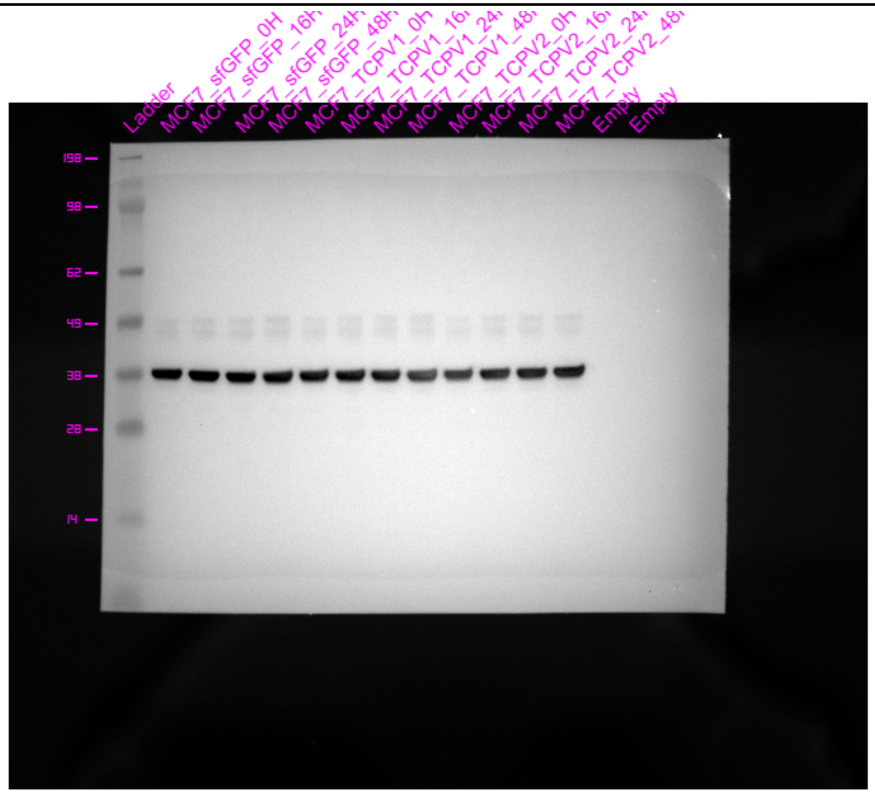

CHEMI_02272022_185805_1s_750ms_MCF7 _TCP_TIMECOURSE_TYRTUB_GAPDH

Date:	02-27-2022 06:58:05PM
Mode:	Chemi Blots
Notes:	
Model:	FL1500
Instrument name:	2462619090234
Serial No:	2462619090234
Firmware version:	1.6.0
iBA version:	4.0.1
Image size:	676px X 540px
Image area:	
Optical Zoom:	2x
Digital Zoom:	1x
Focus level:	455
Resolution:	5 x 5
Exposure time:	1750 ms
Exposure mode:	Normal

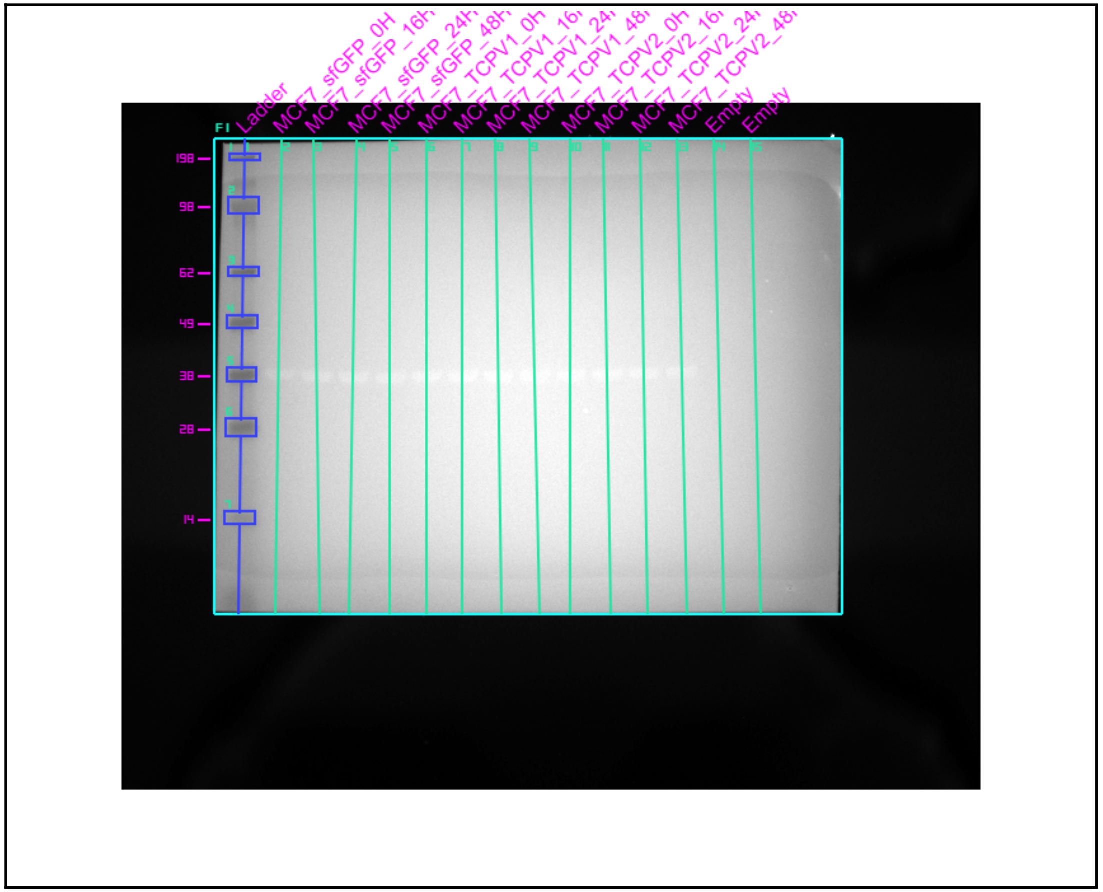

CHEMI_02272022_185805_1s_750ms_MCF7_TCP_TIMECOURSE_TYRTUB_GAPDH

Date:	02-27-2022 06:58:05PM
Mode:	Chemi Blots
Notes:	
Model:	FL1500
Instrument name:	2462619090234
Serial No:	2462619090234
Firmware version:	1.6.0
iBA version:	4.0.1
Image size:	676px X 540px
Image area:	
Optical Zoom:	2x
Digital Zoom:	1x
Focus level:	455
Resolution:	5 x 5
Exposure time:	1750 ms
Exposure mode:	Normal

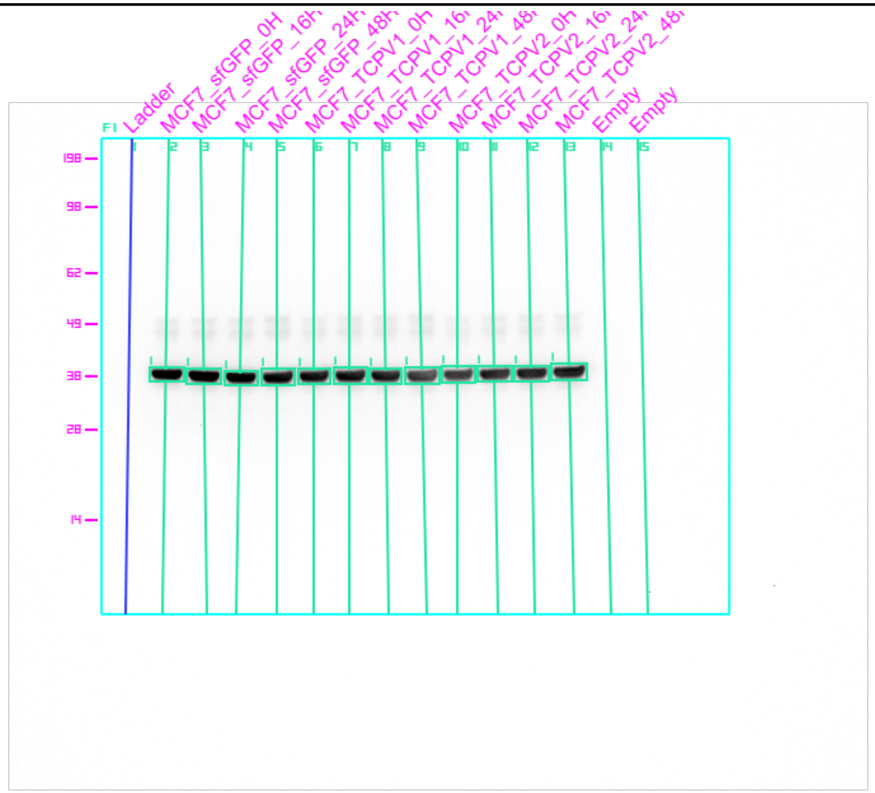

LANE AND BAND ANALYSIS DATA TABLE

CHEMI_02272022_185805_1s_750ms_MCF7_TCP_TIMECOURSE_TYRTUB_GAPDH

Frame: 1
Channel: Membrane
Sensitivity: 100
Molecular Weight Analysis Regression Method : Point to Point

Lane 1 - Ladder

#	Vol. (Int.)	Local Bg. Corr. Vol.	Area	Rf	Density	Local Bg. Corr. Den.	% band purity	% lane purity	Mol. Wt.
1	6,054,567	391,650	150	0.037	40,363	2,611.004	4.749	1.807	198
2	13,981,568	1,106,402	350	0.139	39,947	3,161.149	13.415	4.172	98
3	8,078,384	1,086,871	200	0.278	40,391	5,434.359	13.178	2.411	62
4	11,087,841	1,516,312	275	0.385	40,319	5,513.865	18.385	3.309	49
5	11,679,374	1,736,032	288	0.495	40,553	6,027.892	21.05	3.485	38
6	14,716,862	1,861,440	375	0.607	39,244	4,963.842	22.57	4.392	28
7	10,169,649	548,648	275	0.797	36,980	1,995.086	6.652	3.035	14

Frame: 1
Channel: Chemi
Sensitivity: 100
Molecular Weight Analysis Regression Method : Point to Point

Lane 2 - MCF7_sfGFP_0H

#	Vol. (Int.)	Local Bg. Corr. Vol.	Area	Rf	Density	Local Bg. Corr. Den.	% band purity	% lane purity	Mol. Wt.
1	9,485,854	7,677,680	336	0.495	28,231	22,850	100	64.98	38

Lane 3 - MCF7_sfGFP_16H

#	Vol. (Int.)	Local Bg. Corr. Vol.	Area	Rf	Density	Local Bg. Corr. Den.	% band purity	% lane purity	Mol. Wt.
1	10,392,289	8,022,428	364	0.5	28,550	22,039	100	64.288	37.524

Lane 4 - MCF7_sfGFP_24H

#	Vol. (Int.)	Local Bg. Corr. Vol.	Area	Rf	Density	Local Bg. Corr. Den.	% band purity	% lane purity	Mol. Wt.
1	9,170,095	7,130,204	324	0.503	28,302	22,006	100	59.694	37.286

Lane 5 - MCF7_sfGFP_48H

#	Vol. (Int.)	Local Bg. Corr. Vol.	Area	Rf	Density	Local Bg. Corr. Den.	% band purity	% lane purity	Mol. Wt.
1	9,191,363	7,175,503	378	0.5	24,315	18,982	100	61.774	37.524

Lane 6 - MCF7_TCPV1_0H

#	Vol. (Int.)	Local Bg. Corr. Vol.	Area	Rf	Density	Local Bg. Corr. Den.	% band purity	% lane purity	Mol. Wt.
1	8,277,838	6,381,251	338	0.5	24,490	18,879	100	59.557	37.524

Lane 7 - MCF7_TCPV1_16H

#	Vol. (Int.)	Local Bg. Corr. Vol.	Area	Rf	Density	Local Bg. Corr. Den.	% band purity	% lane purity	Mol. Wt.
1	8,758,291	6,562,163	378	0.497	23,170	17,360	100	61.218	37.762

Lane 8 - MCF7_TCPV1_24H

#	Vol. (Int.)	Local Bg. Corr. Vol.	Area	Rf	Density	Local Bg. Corr. Den.	% band purity	% lane purity	Mol. Wt.
1	7,991,171	6,097,250	338	0.5	23,642	18,039	100	58.334	37.524

Lane 9 - MCF7_TCPV1_48H

#	Vol. (Int.)	Local Bg. Corr. Vol.	Area	Rf	Density	Local Bg. Corr. Den.	% band purity	% lane purity	Mol. Wt.
1	7,419,884	5,449,908	392	0.497	18,928	13,902	100	59.002	37.762

Lane 10 - MCF7_TCPV2_0H

#	Vol. (Int.)	Local Bg. Corr. Vol.	Area	Rf	Density	Local Bg. Corr. Den.	% band purity	% lane purity	Mol. Wt.
1	6,910,890	5,116,727	378	0.495	18,282	13,536	100	60.104	38

Lane 11 - MCF7_TCPV2_16H

#	Vol. (Int.)	Local Bg. Corr. Vol.	Area	Rf	Density	Local Bg. Corr. Den.	% band purity	% lane purity	Mol. Wt.
1	7,725,834	5,888,968	364	0.495	21,224	16,178	100	61.09	38

Lane 12 - MCF7_TCPV2_24H

#	Vol. (Int.)	Local Bg. Corr. Vol.	Area	Rf	Density	Local Bg. Corr. Den.	% band purity	% lane purity	Mol. Wt.
1	8,034,459	6,068,574	364	0.495	22,072	16,671	100	61.677	38

Lane 13 - MCF7_TCPV2_48H

#	Vol. (Int.)	Local Bg. Corr. Vol.	Area	Rf	Density	Local Bg. Corr. Den.	% band purity	% lane purity	Mol. Wt.
1	8,826,835	7,272,485	406	0.489	21,740	17,912	100	66.833	38.537

iBright™ Image Analysis Report

28 January 2022

Figure 2A- deTyr-Tub

CHEMI_04072021_183824_180ms_10A_ALL _TCP_CONSTRUCTS_deTyr_Abcam

Date:	04-07-2021 06:38:24PM
Mode:	Chemi Blots
Notes:	Figure2A- deTyr-Tub
Model:	FL1500
Instrument name:	2462619090234
Serial No:	2462619090234
Firmware version:	1.5.0
iBA version:	4.0.1
Image size:	676px X 540px
Image area:	
Optical Zoom:	1.7x
Digital Zoom:	1x
Focus level:	380
Resolution:	5 x 5
Exposure time:	180 ms
Exposure mode:	Normal

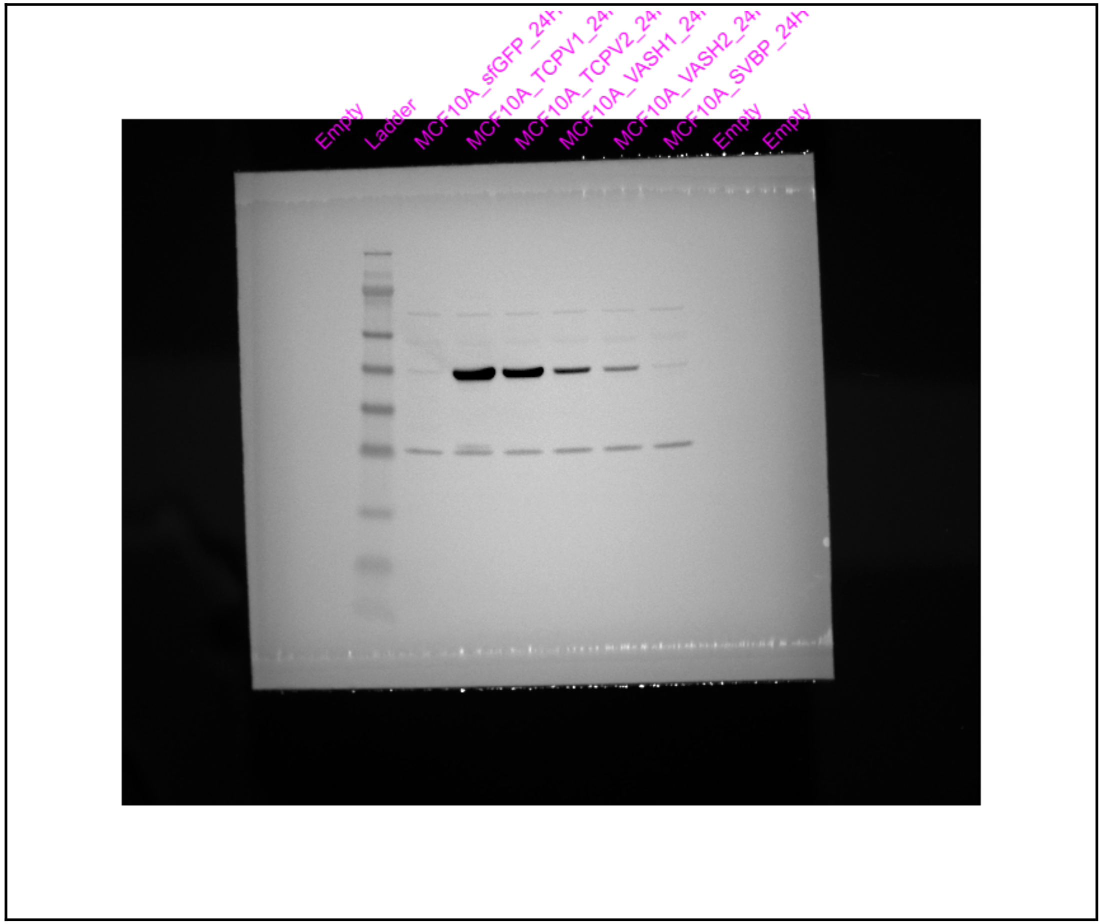

CHEMI_04072021_183824_180ms_10A_ALL _TCP_CONSTRUCTS_deTyr_Abcam

Date:	04-07-2021 06:38:24PM
Mode:	Chemi Blots
Notes:	Figure2A- deTyr-Tub
Model:	FL1500
Instrument name:	2462619090234
Serial No:	2462619090234
Firmware version:	1.5.0
iBA version:	4.0.1
Image size:	676px X 540px
Image area:	
Optical Zoom:	1.7x
Digital Zoom:	1x
Focus level:	380
Resolution:	5 x 5
Exposure time:	180 ms
Exposure mode:	Normal

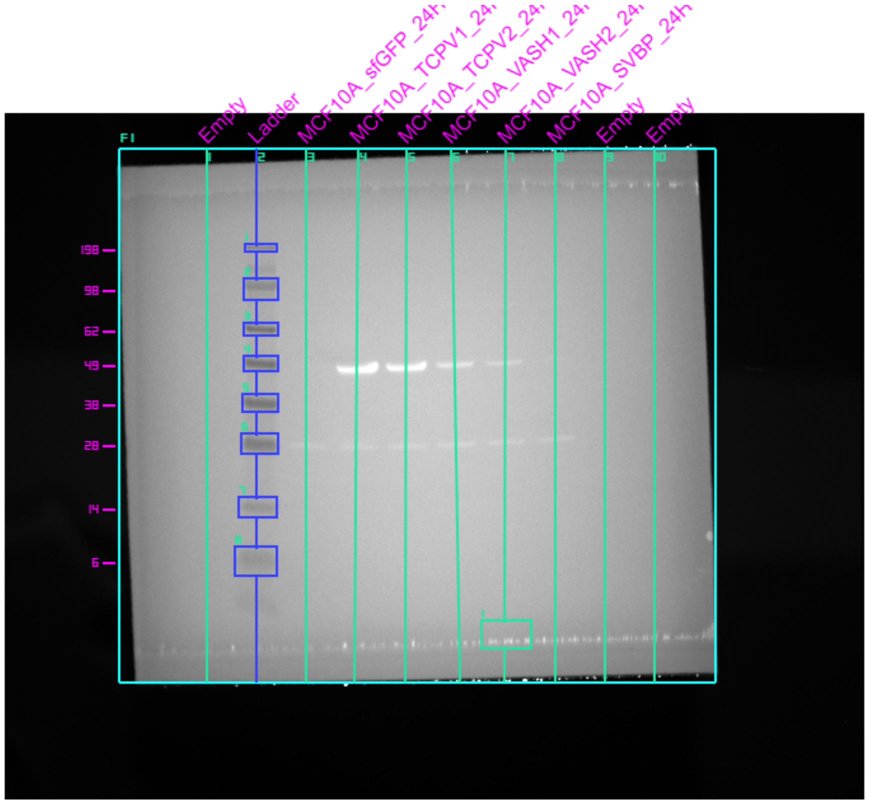

CHEMI_04072021_183824_180ms_10A_ALL _TCP_CONSTRUCTS_deTyr_Abcam

Date:	04-07-2021 06:38:24PM
Mode:	Chemi Blots
Notes:	Figure2A- deTyr-Tub
Model:	FL1500
Instrument name:	2462619090234
Serial No:	2462619090234
Firmware version:	1.5.0
iBA version:	4.0.1
Image size:	676px X 540px
Image area:	
Optical Zoom:	1.7x
Digital Zoom:	1x
Focus level:	380
Resolution:	5 x 5
Exposure time:	180 ms
Exposure mode:	Normal

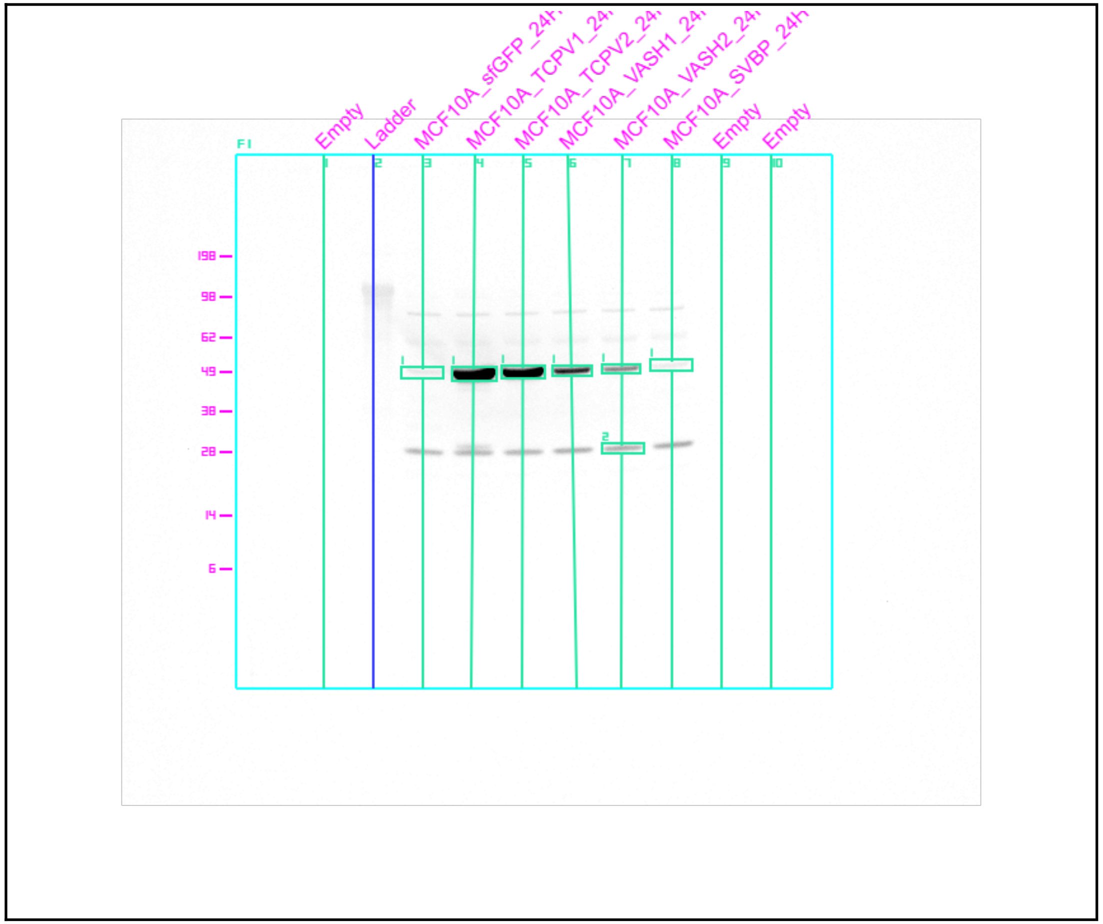

LANE AND BAND ANALYSIS DATA TABLE

CHEMI_04072021_183824_180ms_10A_ALL_TCP_CONSTRUCTS_deTyr_Abcam

Frame: 1
Channel: Membrane
Sensitivity: 100
Molecular Weight Analysis Regression Method : Point to Point

Lane 2 - Ladder

#	Vol. (Int.)	Local Bg. Corr. Vol.	Area	Rf	Density	Local Bg. Corr. Den.	% band purity	% lane purity	Mol. Wt.
1	5,117,155	627,034	182	0.186	28,116	3,445.243	3.764	1.406	198
2	13,992,726	1,926,834	504	0.262	27,763	3,823.084	11.567	3.845	98
3	9,080,486	1,918,354	308	0.338	29,482	6,228.425	11.516	2.495	62
4	11,020,899	2,592,713	364	0.402	30,277	7,122.839	15.564	3.028	49
5	13,182,911	3,017,890	435	0.476	30,305	6,937.679	18.116	3.623	38
6	15,025,383	3,096,801	510	0.552	29,461	6,072.161	18.59	4.129	28
7	14,006,581	1,526,765	527	0.671	26,577	2,897.089	9.165	3.849	14
8	22,283,164	1,952,007	816	0.771	27,307	2,392.167	11.718	6.123	6

Lane 7 - MCF10A_VASH2_24H

#	Vol. (Int.)	Local Bg. Corr. Vol.	Area	Rf	Density	Local Bg. Corr. Den.	% band purity	% lane purity	Mol. Wt.
1	22,500,923	194,113	920	0.91	24,457	210.993	100	7.751	NA

Frame: 1
Channel: Chemi
Sensitivity: 100
Molecular Weight Analysis Regression Method : Point to Point

Lane 3 - MCF10A_sfGFP_24H

#	Vol. (Int.)	Local Bg. Corr. Vol.	Area	Rf	Density	Local Bg. Corr. Den.	% band purity	% lane purity	Mol. Wt.
1	271,778	123,736	340	0.407	799.347	363.93	100	8.158	48.29

Lane 4 - MCF10A_TCPV1_24H

#	Vol. (Int.)	Local Bg. Corr. Vol.	Area	Rf	Density	Local Bg. Corr. Den.	% band purity	% lane purity	Mol. Wt.
1	7,974,257	7,329,870	432	0.41	18,458	16,967	100	67.944	47.935

Lane 5 - MCF10A_TCPV2_24H

#	Vol. (Int.)	Local Bg. Corr. Vol.	Area	Rf	Density	Local Bg. Corr. Den.	% band purity	% lane purity	Mol. Wt.
1	5,533,671	5,156,913	385	0.407	14,373	13,394	100	63.975	48.29

Lane 6 - MCF10A_VASH1_24H

#	Vol. (Int.)	Local Bg. Corr. Vol.	Area	Rf	Density	Local Bg. Corr. Den.	% band purity	% lane purity	Mol. Wt.
1	1,812,901	1,672,489	288	0.405	6,294.795	5,807.255	100	39.679	48.645

Lane 7 - MCF10A_VASH2_24H

#	Vol. (Int.)	Local Bg. Corr. Vol.	Area	Rf	Density	Local Bg. Corr. Den.	% band purity	% lane purity	Mol. Wt.
1	847,765	772,919	248	0.4	3,418.407	3,116.61	57.822	26.318	49.481
2	613,052	563,808	306	0.55	2,003.438	1,842.511	42.178	19.031	28.313

Lane 8 - MCF10A_SVBP_24H

#	Vol. (Int.)	Local Bg. Corr. Vol.	Area	Rf	Density	Local Bg. Corr. Den.	% band purity	% lane purity	Mol. Wt.
1	186,934	126,373	340	0.393	549.806	371.686	100	7.941	50.926

iBright™ Image Analysis Report

28 January 2022

Figure 2A- Alpha Tubulin

CHEMI_04072021_184204_629ms_10A_ALL _TCP_CONSTRUCTS_polyALPHA

Date:	04-07-2021 06:42:04PM
Mode:	Chemi Blots
Notes:	Figure2A- Alpha
Model:	FL1500
Instrument name:	2462619090234
Serial No:	2462619090234
Firmware version:	1.5.0
iBA version:	4.0.1
Image size:	676px X 540px
Image area:	
Optical Zoom:	1.9x
Digital Zoom:	1x
Focus level:	430
Resolution:	5 x 5
Exposure time:	629 ms
Exposure mode:	Normal

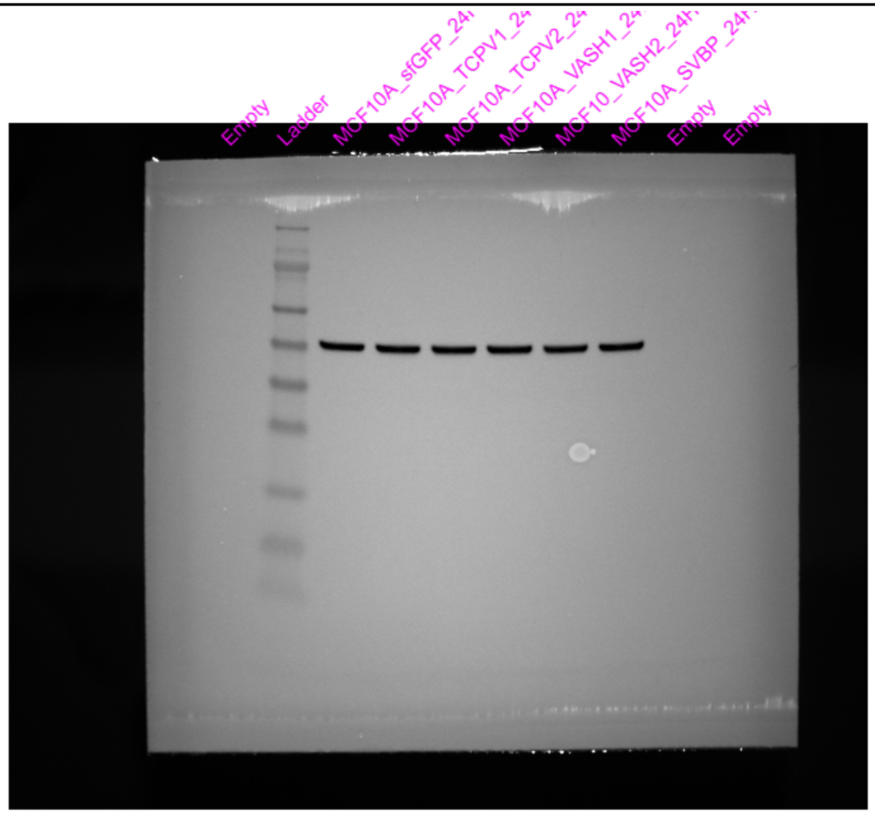

CHEMI_04072021_184204_629ms_10A_ALL_TCP_CONSTRUCTS_polyALPHA

Date:	04-07-2021 06:42:04PM
Mode:	Chemi Blots
Notes:	Figure2A- Alpha
Model:	FL1500
Instrument name:	2462619090234
Serial No:	2462619090234
Firmware version:	1.5.0
iBA version:	4.0.1
Image size:	676px X 540px
Image area:	
Optical Zoom:	1.9x
Digital Zoom:	1x
Focus level:	430
Resolution:	5 x 5
Exposure time:	629 ms
Exposure mode:	Normal

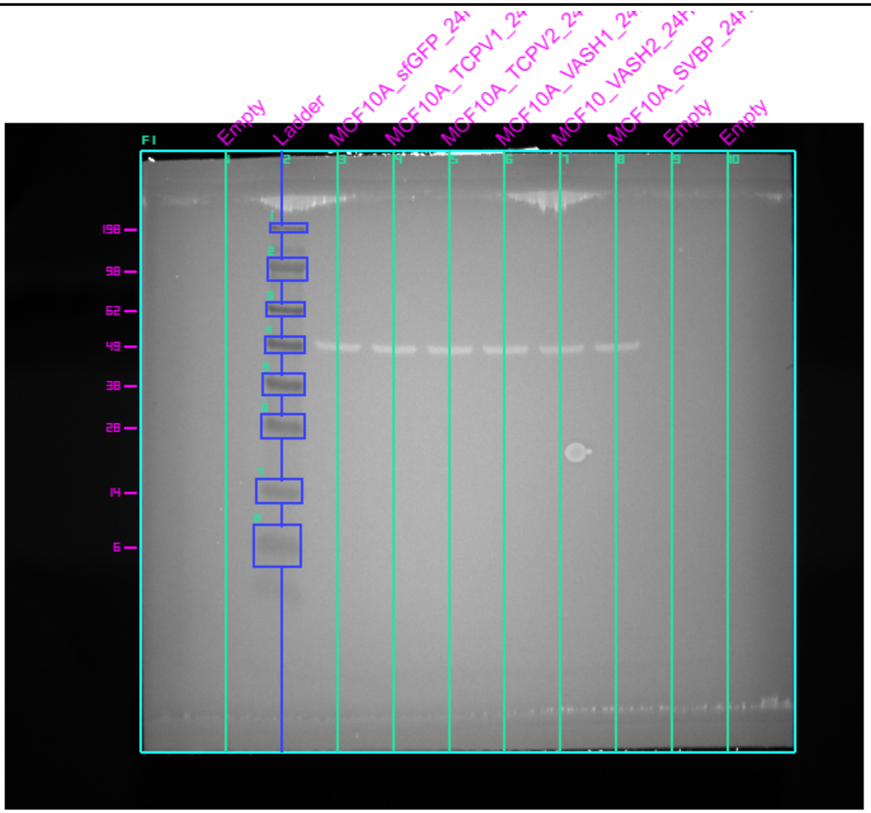

CHEMI_04072021_184204_629ms_10A_ALL _TCP_CONSTRUCTS_polyALPHA

Date:	04-07-2021 06:42:04PM
Mode:	Chemi Blots
Notes:	Figure2A- Alpha
Model:	FL1500
Instrument name:	2462619090234
Serial No:	2462619090234
Firmware version:	1.5.0
iBA version:	4.0.1
Image size:	676px X 540px
Image area:	
Optical Zoom:	1.9x
Digital Zoom:	1x
Focus level:	430
Resolution:	5 x 5
Exposure time:	629 ms
Exposure mode:	Normal

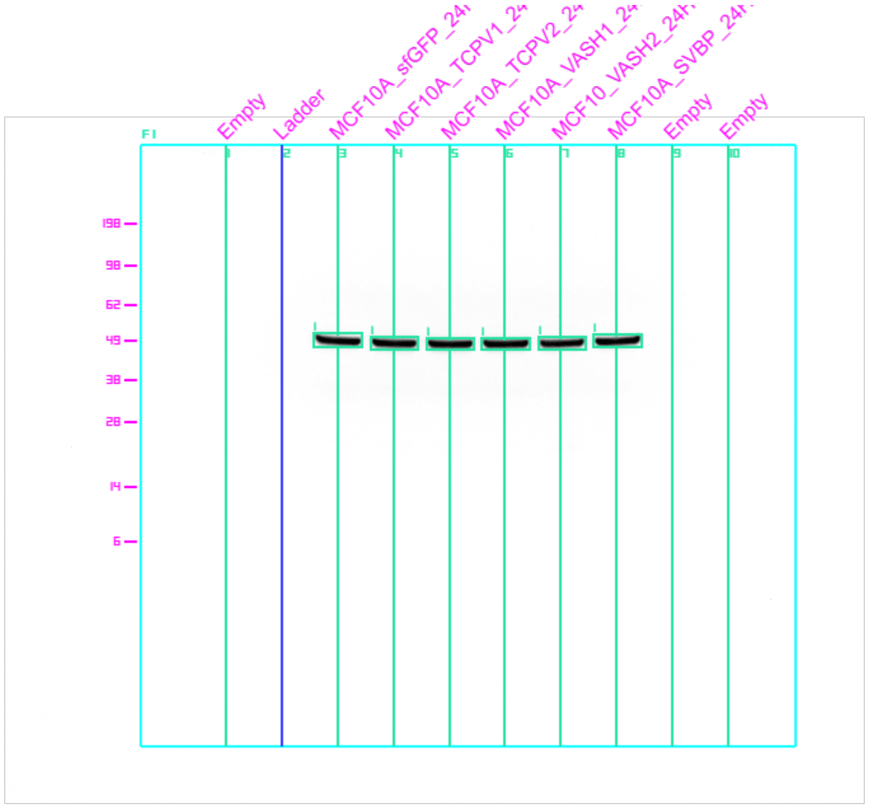

LANE AND BAND ANALYSIS DATA TABLE

CHEMI_04072021_184204_629ms_10A_ALL_TCP_CONSTRUCTS_polyALPHA

Frame: 1
Channel: Membrane
Sensitivity: 100
Molecular Weight Analysis Regression Method : Point to Point

Lane 2 - Ladder

#	Vol. (Int.)	Local Bg. Corr. Vol.	Area	Rf	Density	Local Bg. Corr. Den.	% band purity	% lane purity	Mol. Wt.
1	7,532,988	806,512	240	0.127	31,387	3,360.47	4.09	1.558	198
2	18,556,308	2,353,350	608	0.197	30,520	3,870.643	11.935	3.837	98
3	11,764,065	2,242,321	372	0.262	31,623	6,027.747	11.372	2.433	62
4	14,152,926	2,910,911	448	0.321	31,591	6,497.57	14.763	2.926	49
5	18,453,571	3,490,242	612	0.387	30,152	5,703.01	17.701	3.816	38
6	20,017,005	3,110,325	700	0.457	28,595	4,443.323	15.774	4.139	28
7	20,098,658	2,082,803	740	0.564	27,160	2,814.599	10.563	4.156	14
8	35,089,737	2,721,363	1,292	0.655	27,159	2,106.318	13.802	7.256	6

Frame: 1
Channel: Chemi
Sensitivity: 100
Molecular Weight Analysis Regression Method : Point to Point

Lane 3 - MCF10A_sfGFP_24H

#	Vol. (Int.)	Local Bg. Corr. Vol.	Area	Rf	Density	Local Bg. Corr. Den.	% band purity	% lane purity	Mol. Wt.
1	10,155,664	9,740,861	468	0.323	21,700	20,813	100	79.94	48.645

Lane 4 - MCF10A_TCPV1_24H

#	Vol. (Int.)	Local Bg. Corr. Vol.	Area	Rf	Density	Local Bg. Corr. Den.	% band purity	% lane purity	Mol. Wt.
1	10,037,044	9,436,593	418	0.33	24,012	22,575	100	74.681	47.581

Lane 5 - MCF10A_TCPV2_24H

#	Vol. (Int.)	Local Bg. Corr. Vol.	Area	Rf	Density	Local Bg. Corr. Den.	% band purity	% lane purity	Mol. Wt.
1	9,773,914	9,150,599	380	0.33	25,720	24,080	100	73.358	47.581

Lane 6 - MCF10A_VASH1_24H

#	Vol. (Int.)	Local Bg. Corr. Vol.	Area	Rf	Density	Local Bg. Corr. Den.	% band purity	% lane purity	Mol. Wt.
1	9,943,767	9,364,034	390	0.33	25,496	24,010	100	75.132	47.581

Lane 7 - MCF10_VASH2_24H

#	Vol. (Int.)	Local Bg. Corr. Vol.	Area	Rf	Density	Local Bg. Corr. Den.	% band purity	% lane purity	Mol. Wt.
1	9,163,749	8,705,262	418	0.33	21,922	20,825	100	75.467	47.581

Lane 8 - MCF10A_SVBP_24H

#	Vol. (Int.)	Local Bg. Corr. Vol.	Area	Rf	Density	Local Bg. Corr. Den.	% band purity	% lane purity	Mol. Wt.
1	9,931,946	9,561,651	429	0.326	23,151	22,288	100	81.042	48.29

iBright™ Image Analysis Report

28 January 2022

Figure 2A- myc-tag

CHEMI_04072021_185249_511ms_10A_ALL_TCP_CONSTRUCTS_MYCtag

Date:	04-07-2021 06:52:49PM
Mode:	Chemi Blots
Notes:	Figure2A- Myc-tag
Model:	FL1500
Instrument name:	2462619090234
Serial No:	2462619090234
Firmware version:	1.5.0
iBA version:	4.0.1
Image size:	676px X 540px
Image area:	
Optical Zoom:	2x
Digital Zoom:	1x
Focus level:	455
Resolution:	5 x 5
Exposure time:	511 ms
Exposure mode:	Normal

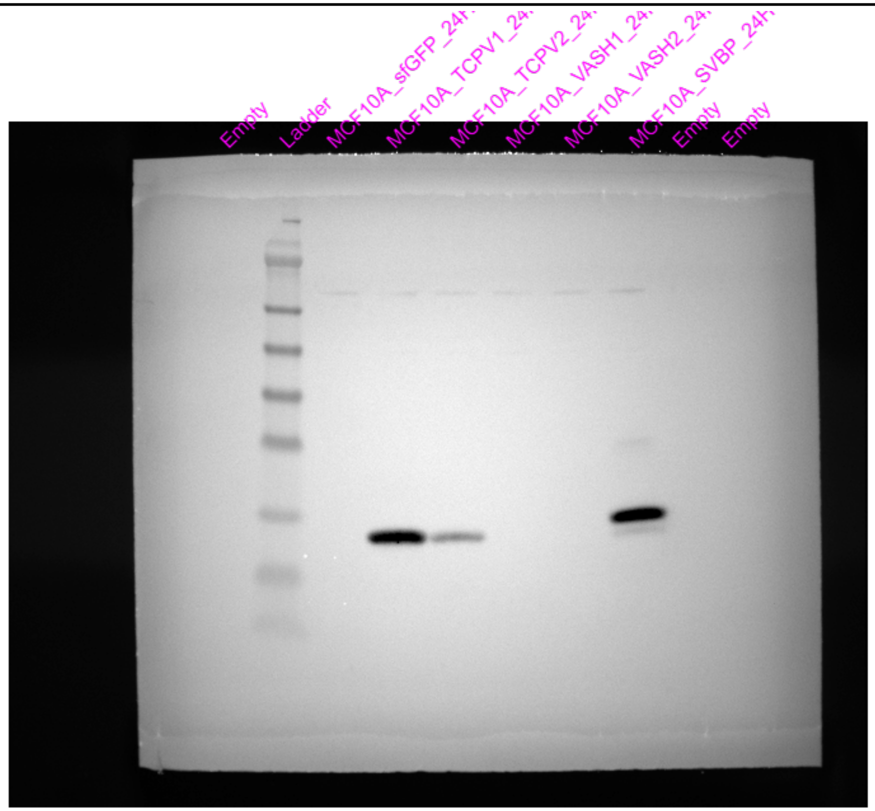

CHEMI_04072021_185249_511ms_10A_ALL _TCP_CONSTRUCTS_MYCtag

Date:	04-07-2021 06:52:49PM
Mode:	Chemi Blots
Notes:	Figure2A- Myc-tag
Model:	FL1500
Instrument name:	2462619090234
Serial No:	2462619090234
Firmware version:	1.5.0
iBA version:	4.0.1
Image size:	676px X 540px
Image area:	
Optical Zoom:	2x
Digital Zoom:	1x
Focus level:	455
Resolution:	5 x 5
Exposure time:	511 ms
Exposure mode:	Normal

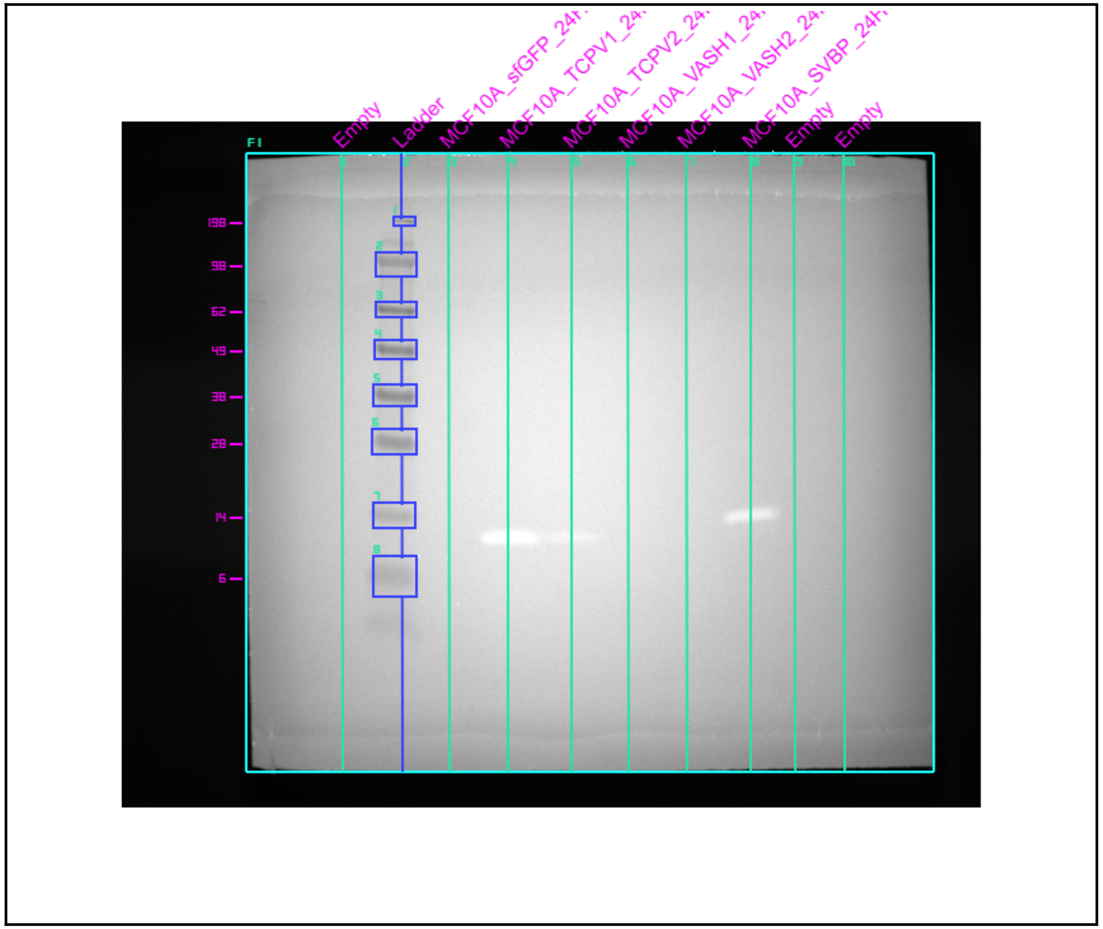

CHEMI_04072021_185249_511ms_10A_ALL _TCP_CONSTRUCTS_MYCtag

Date:	04-07-2021 06:52:49PM
Mode:	Chemi Blots
Notes:	Figure2A- Myc-tag
Model:	FL1500
Instrument name:	2462619090234
Serial No:	2462619090234
Firmware version:	1.5.0
iBA version:	4.0.1
Image size:	676px X 540px
Image area:	
Optical Zoom:	2x
Digital Zoom:	1x
Focus level:	455
Resolution:	5 x 5
Exposure time:	511 ms
Exposure mode:	Normal

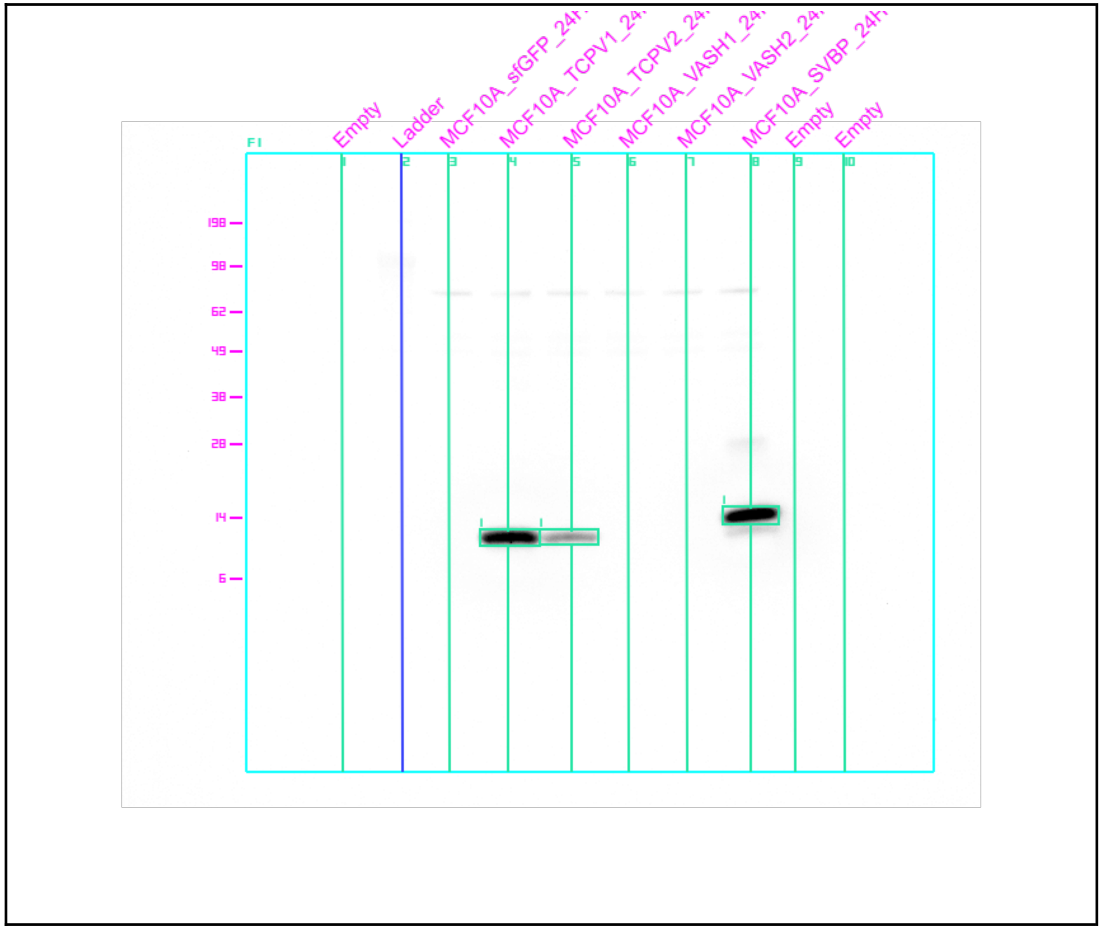

LANE AND BAND ANALYSIS DATA TABLE

CHEMI_04072021_185249_511ms_10A_ALL_TCP_CONSTRUCTS_MYCtag

Frame: 1
Channel: Membrane
Sensitivity: 100
Molecular Weight Analysis Regression Method : Point to Point

Lane 2 - Ladder

#	Vol. (Int.)	Local Bg. Corr. Vol.	Area	Rf	Density	Local Bg. Corr. Den.	% band purity	% lane purity	Mol. Wt.
1	4,357,228	431,351	144	0.109	30,258	2,995.496	1.967	0.752	198
2	20,028,462	2,542,732	660	0.179	30,346	3,852.625	11.597	3.455	98
3	13,501,548	2,530,559	429	0.253	31,472	5,898.739	11.541	2.329	62
4	16,693,836	3,506,807	544	0.316	30,687	6,446.338	15.994	2.88	49
5	19,041,340	4,143,804	630	0.39	30,224	6,577.467	18.899	3.285	38
6	21,799,627	4,099,315	756	0.466	28,835	5,422.374	18.696	3.761	28
7	19,191,341	2,108,673	714	0.585	26,878	2,953.325	9.617	3.311	14
8	31,714,448	2,563,076	1,155	0.684	27,458	2,219.114	11.689	5.471	6

Frame: 1
Channel: Chemi
Sensitivity: 100
Molecular Weight Analysis Regression Method : Point to Point

Lane 4 - MCF10A_TCPV1_24H

#	Vol. (Int.)	Local Bg. Corr. Vol.	Area	Rf	Density	Local Bg. Corr. Den.	% band purity	% lane purity	Mol. Wt.
1	13,537,199	12,123,344	672	0.62	20,144	18,040	100	79.736	11.167

Lane 5 - MCF10A_TCPV2_24H

#	Vol. (Int.)	Local Bg. Corr. Vol.	Area	Rf	Density	Local Bg. Corr. Den.	% band purity	% lane purity	Mol. Wt.
1	4,212,920	3,054,799	611	0.62	6,895.123	4,999.672	100	59.298	11.167

Lane 8 - MCF10A_SVBP_24H

#	Vol. (Int.)	Local Bg. Corr. Vol.	Area	Rf	Density	Local Bg. Corr. Den.	% band purity	% lane purity	Mol. Wt.
1	15,213,765	13,011,354	675	0.585	22,538	19,276	100	70.835	14

iBright™ Image Analysis Report

28 January 2022

Figure 2A- FLAG

CHEMI_04142021_180625_450ms_10A_ALL _TCP_CONSTRUCTS_FLAGtag

Date:	04-14-2021 06:06:25PM
Mode:	Chemi Blots
Notes:	Figure 2A- FLAG
Model:	FL1500
Instrument name:	2462619090234
Serial No:	2462619090234
Firmware version:	1.5.0
iBA version:	4.0.1
Image size:	676px X 540px
Image area:	
Optical Zoom:	1.9x
Digital Zoom:	1x
Focus level:	430
Resolution:	5 x 5
Exposure time:	450 ms
Exposure mode:	Normal

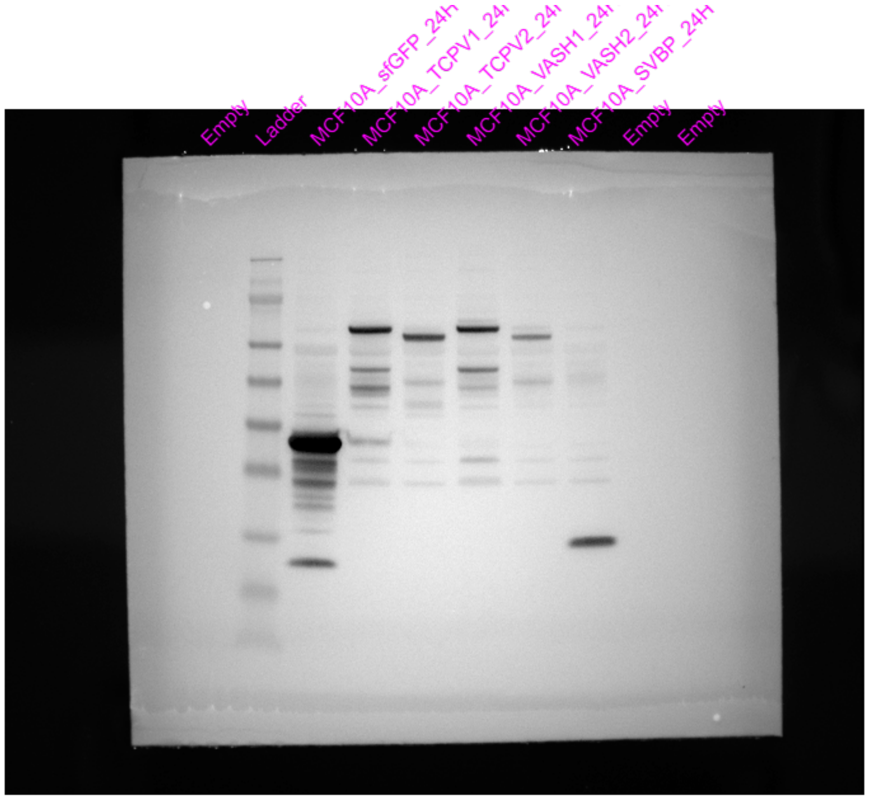

CHEMI_04142021_180625_450ms_10A_ALL _TCP_CONSTRUCTS_FLAGtag

Date:	04-14-2021 06:06:25PM
Mode:	Chemi Blots
Notes:	Figure 2A- FLAG
Model:	FL1500
Instrument name:	2462619090234
Serial No:	2462619090234
Firmware version:	1.5.0
iBA version:	4.0.1
Image size:	676px X 540px
Image area:	
Optical Zoom:	1.9x
Digital Zoom:	1x
Focus level:	430
Resolution:	5 x 5
Exposure time:	450 ms
Exposure mode:	Normal

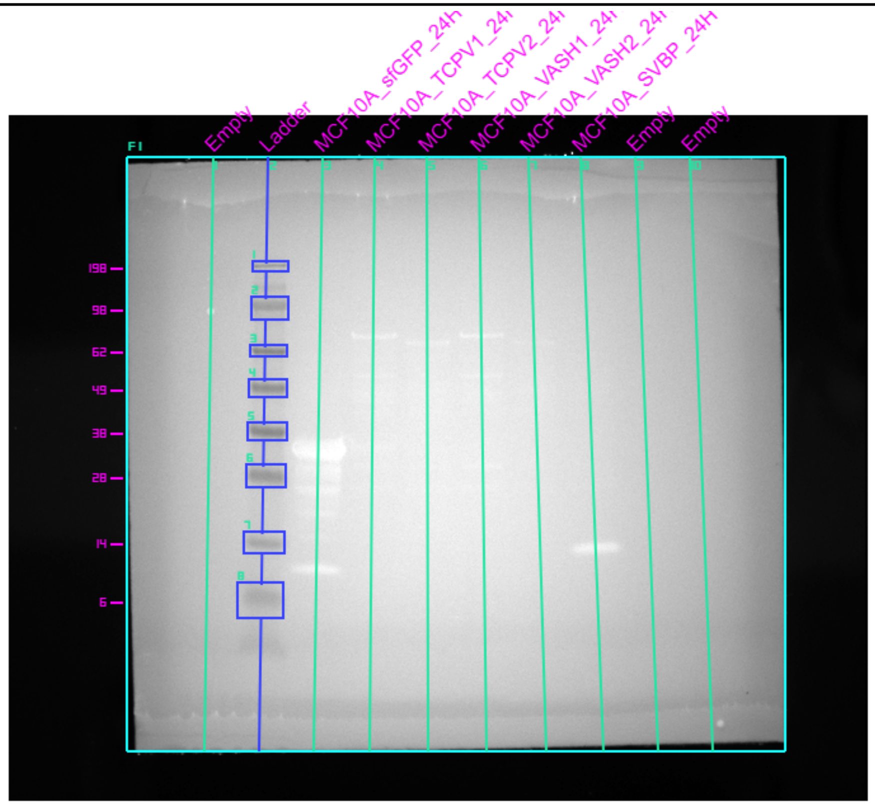

CHEMI_04142021_180625_450ms_10A_ALL _TCP_CONSTRUCTS_FLAGtag

Date:	04-14-2021 06:06:25PM
Mode:	Chemi Blots
Notes:	Figure 2A- FLAG
Model:	FL1500
Instrument name:	2462619090234
Serial No:	2462619090234
Firmware version:	1.5.0
iBA version:	4.0.1
Image size:	676px X 540px
Image area:	
Optical Zoom:	1.9x
Digital Zoom:	1x
Focus level:	430
Resolution:	5 x 5
Exposure time:	450 ms
Exposure mode:	Normal

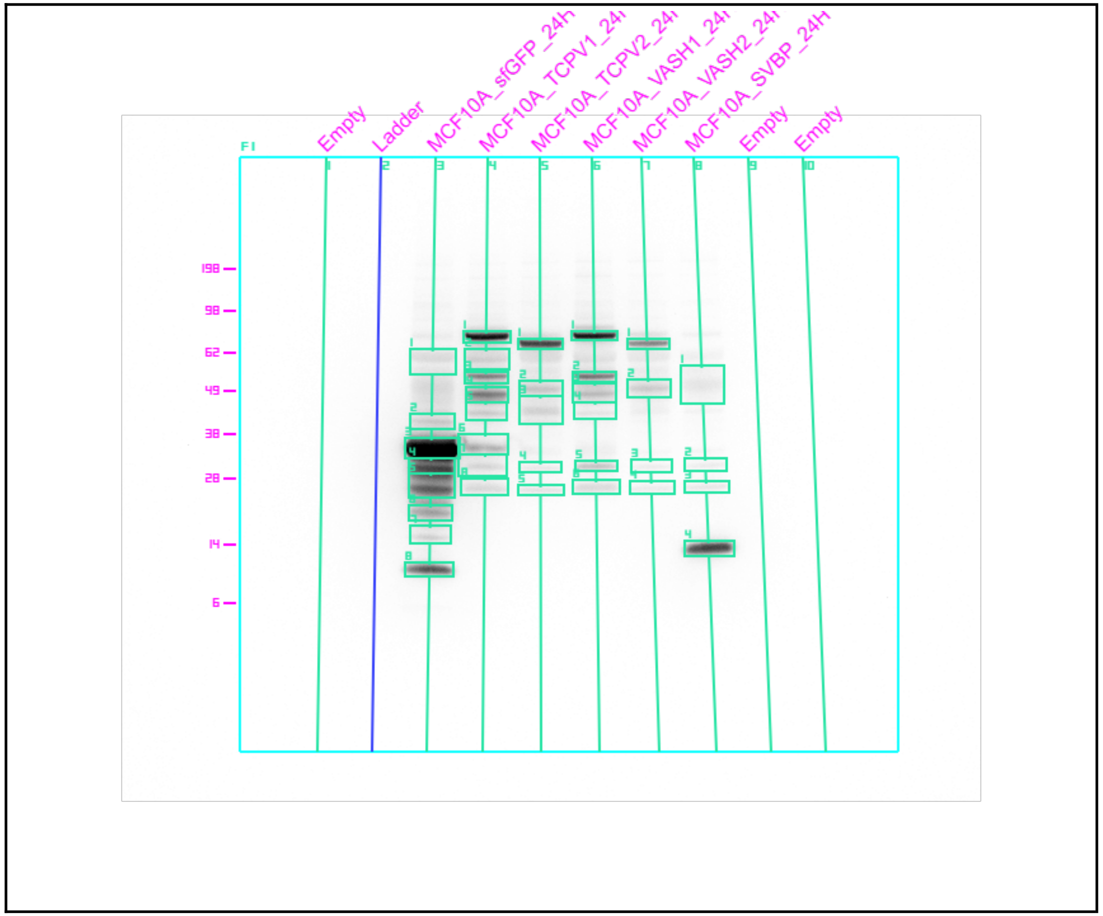

LANE AND BAND ANALYSIS DATA TABLE

CHEMI_04142021_180625_450ms_10A_ALL_TCP_CONSTRUCTS_FLAGtag

Frame: 1
Channel: Membrane
Sensitivity: 100
Molecular Weight Analysis Regression Method : Point to Point

Lane 2 - Ladder

#	Vol. (Int.)	Local Bg. Corr. Vol.	Area	Rf	Density	Local Bg. Corr. Den.	% band purity	% lane purity	Mol. Wt.
1	7,466,261	693,533	261	0.184	28,606	2,657.218	3.646	1.722	198
2	16,525,026	2,162,714	570	0.254	28,991	3,794.235	11.37	3.812	98
3	9,403,824	2,009,827	300	0.325	31,346	6,699.426	10.566	2.169	62
4	13,984,688	2,824,718	465	0.389	30,074	6,074.664	14.851	3.226	49
5	14,840,559	3,396,725	480	0.462	30,917	7,076.512	17.858	3.423	38
6	17,882,127	3,472,549	608	0.536	29,411	5,711.43	18.256	4.125	28
7	16,274,717	1,951,077	594	0.647	27,398	3,284.643	10.258	3.754	14
8	29,771,641	2,509,833	1,073	0.746	27,746	2,339.081	13.195	6.868	6

Frame: 1
Channel: Chemi
Sensitivity: 100
Molecular Weight Analysis Regression Method : Point to Point

Lane 3 - MCF10A_sfGFP_24H

#	Vol. (Int.)	Local Bg. Corr. Vol.	Area	Rf	Density	Local Bg. Corr. Den.	% band purity	% lane purity	Mol. Wt.
1	3,310,213	1,318,078	777	0.344	4,260.248	1,696.368	3.558	3.91	58.1
2	3,087,273	356,941	468	0.444	6,596.737	762.696	0.964	3.647	40.588
3	29,616,368	20,097,932	748	0.489	39,594	26,868	54.257	34.985	34.286
4	12,972,727	1,551,498	481	0.519	26,970	3,225.569	4.188	15.324	30.286
5	14,766,139	5,568,587	740	0.551	19,954	7,525.118	15.033	17.443	26.115
6	4,627,351	1,871,955	455	0.598	10,170	4,114.188	5.054	5.466	20.192
7	2,070,465	698,289	495	0.635	4,182.758	1,410.686	1.885	2.446	15.615
8	6,640,732	5,578,643	468	0.692	14,189	11,920	15.06	7.844	10.348

Lane 4 - MCF10A_TCPV1_24H

#	Vol. (Int.)	Local Bg. Corr. Vol.	Area	Rf	Density	Local Bg. Corr. Den.	% band purity	% lane purity	Mol. Wt.
1	8,171,376	6,450,855	380	0.301	21,503	16,975	44.618	19.387	74
2	3,483,558	146,281	612	0.34	5,692.088	239.022	1.012	8.265	58.967
3	4,243,766	2,229,027	350	0.37	12,125	6,368.651	15.417	10.069	52.9
4	6,228,659	3,748,595	442	0.4	14,091	8,480.985	25.927	14.778	47.382
5	2,968,818	350,429	495	0.427	5,997.612	707.938	2.424	7.044	43.176
6	4,934,306	786,196	680	0.483	7,256.332	1,156.172	5.438	11.707	35.143
7	2,939,976	2,225.852	702	0.517	4,188	3.171	0.015	6.975	30.571
8	2,025,056	744,516	532	0.553	3,806.496	1,399.467	5.149	4.805	25.846

Lane 5 - MCF10A_TCPV2_24H

#	Vol. (Int.)	Local Bg. Corr. Vol.	Area	Rf	Density	Local Bg. Corr. Den.	% band purity	% lane purity	Mol. Wt.
1	6,254,074	4,772,644	324	0.314	19,302	14,730	57.848	24.952	67.455
2	2,818,902	1,386,099	455	0.389	6,195.389	3,046.372	16.801	11.247	49
3	3,609,216	1,294,395	805	0.425	4,483.498	1,607.945	15.689	14.4	43.5
4	687,769	289,533	306	0.521	2,247.611	946.187	3.509	2.744	30
5	858,207	507,616	333	0.56	2,577.198	1,524.373	6.153	3.424	25.038

Lane 6 - MCF10A_VASH1_24H

#	Vol. (Int.)	Local Bg. Corr. Vol.	Area	Rf	Density	Local Bg. Corr. Den.	% band purity	% lane purity	Mol. Wt.
1	6,621,579	5,282,145	296	0.299	22,370	17,845	44.873	21.608	75.091
2	4,032,278	2,606,316	315	0.37	12,800	8,274.021	22.141	13.158	52.9
3	4,020,005	1,430,629	560	0.395	7,178.58	2,554.695	12.154	13.118	48.029
4	1,492,152	244,594	476	0.425	3,134.773	513.854	2.078	4.869	43.5
5	1,610,533	1,120,084	306	0.519	5,263.18	3,660.406	9.515	5.256	30.286
6	1,575,508	1,087,518	456	0.553	3,455.061	2,384.909	9.239	5.141	25.846

Lane 7 - MCF10A_VASH2_24H

#	Vol. (Int.)	Local Bg. Corr. Vol.	Area	Rf	Density	Local Bg. Corr. Den.	% band purity	% lane purity	Mol. Wt.
1	3,190,795	2,288,898	306	0.314	10,427	7,480.06	51.386	19.148	67.455
2	2,562,402	1,474,997	525	0.389	4,880.766	2,809.519	33.114	15.377	49
3	574,492	261,495	363	0.519	1,582.623	720.372	5.871	3.448	30.286

#	Vol. (Int.)	Local Bg. Corr. Vol.	Area	Rf	Density	Local Bg. Corr. Den.	% band purity	% lane purity	Mol. Wt.
4	719,817	428,899	396	0.556	1,817.72	1,083.078	9.629	4.32	25.577

Lane 8 - MCF10A_SVBP_24H

#	Vol. (Int.)	Local Bg. Corr. Vol.	Area	Rf	Density	Local Bg. Corr. Den.	% band purity	% lane purity	Mol. Wt.
1	2,691,559	1,585,587	1,085	0.382	2,480.7	1,461.371	16.128	14.152	50.3
2	592,031	293,457	374	0.517	1,582.971	784.646	2.985	3.113	30.571
3	756,199	462,996	360	0.553	2,100.553	1,286.103	4.709	3.976	25.846
4	8,636,855	7,489,122	520	0.658	16,609	14,402	76.177	45.413	13.13

iBright™ Image Analysis Report

28 January 2022

Figure 2A- GAPDH

CHEMI_04082021_193007_661ms_10A_ALL _TCP_CONSTRUCTS_GAPDH

Date:	04-08-2021 07:30:07PM
Mode:	Chemi Blots
Notes:	Figure 2A- GAPDH on ALPHA
Model:	FL1500
Instrument name:	2462619090234
Serial No:	2462619090234
Firmware version:	1.5.0
iBA version:	4.0.1
Image size:	615px X 491px
Image area:	
Optical Zoom:	2x
Digital Zoom:	1.1x
Focus level:	455
Resolution:	5 x 5
Exposure time:	661 ms
Exposure mode:	Normal

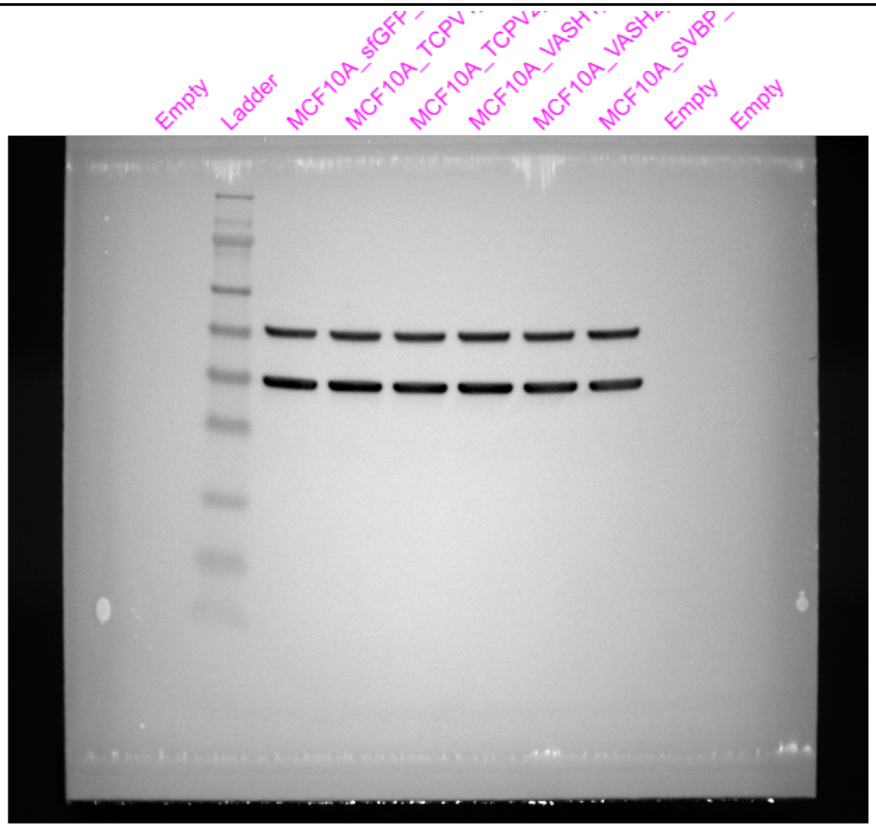

CHEMI_04082021_193007_661ms_10A_ALL _TCP_CONSTRUCTS_GAPDH

Date: 04-08-2021 07:30:07PM
Mode: Chemi Blots
Notes: Figure 2A- GAPDH on ALPHA
Model: FL1500
Instrument name: 2462619090234
Serial No: 2462619090234
Firmware version: 1.5.0
iBA version: 4.0.1
Image size: 615px X 491px
Image area:
Optical Zoom: 2x
Digital Zoom: 1.1x
Focus level: 455
Resolution: 5 x 5
Exposure time: 661 ms
Exposure mode: Normal

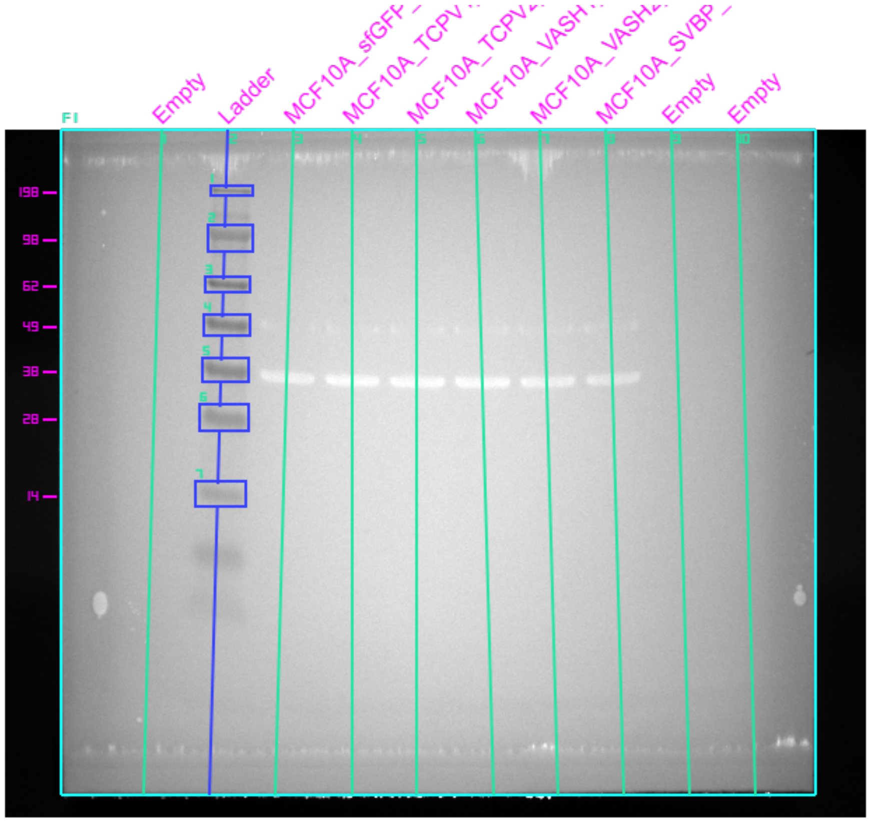

CHEMI_04082021_193007_661ms_10A_ALL _TCP_CONSTRUCTS_GAPDH

Date:	04-08-2021 07:30:07PM
Mode:	Chemi Blots
Notes:	Figure 2A- GAPDH on ALPHA
Model:	FL1500
Instrument name:	2462619090234
Serial No:	2462619090234
Firmware version:	1.5.0
iBA version:	4.0.1
Image size:	615px X 491px
Image area:	
Optical Zoom:	2x
Digital Zoom:	1.1x
Focus level:	455
Resolution:	5 x 5
Exposure time:	661 ms
Exposure mode:	Normal

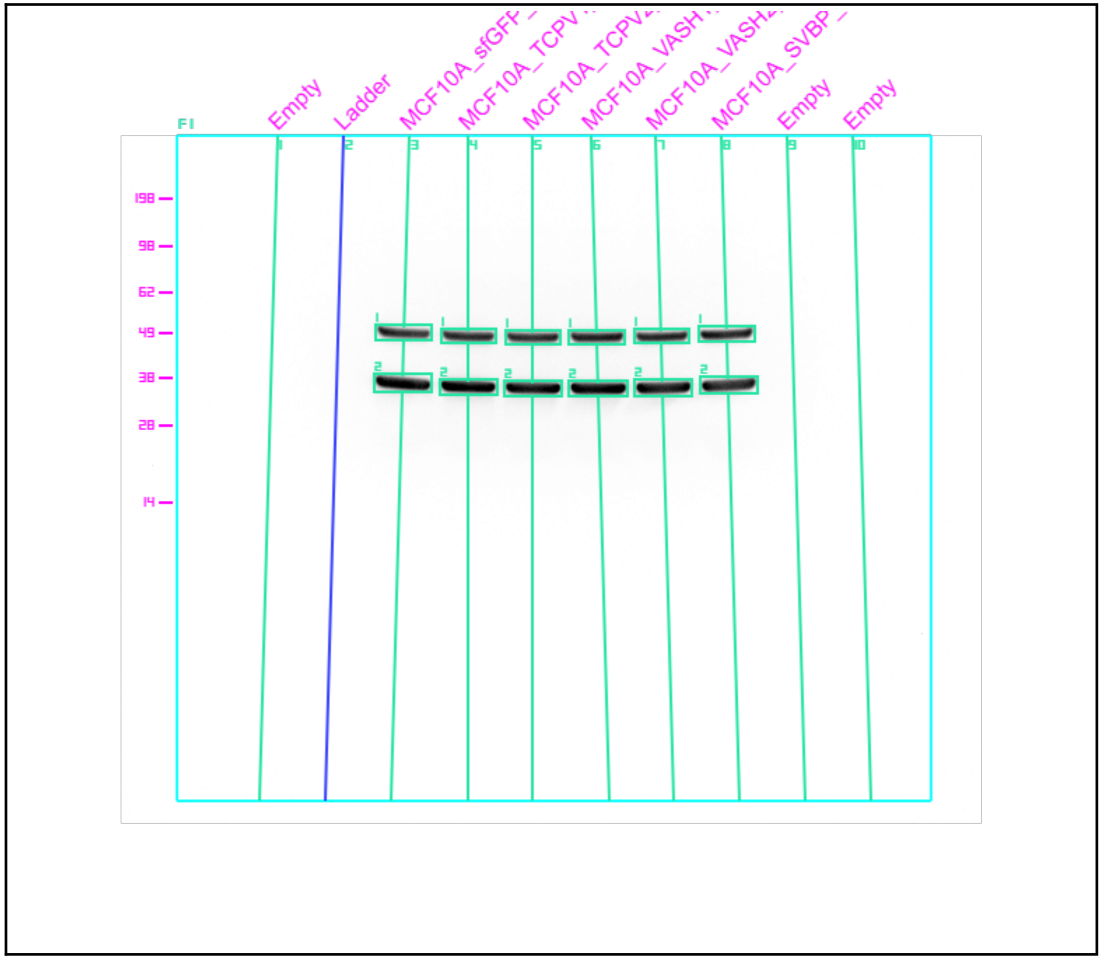

LANE AND BAND ANALYSIS DATA TABLE

CHEMI_04082021_193007_661ms_10A_ALL_TCP_CONSTRUCTS_GAPDH

Frame: 1
Channel: Membrane
Sensitivity: 100
Molecular Weight Analysis Regression Method : Point to Point

Lane 2 - Ladder

#	Vol. (Int.)	Local Bg. Corr. Vol.	Area	Rf	Density	Local Bg. Corr. Den.	% band purity	% lane purity	Mol. Wt.
1	7,883,646	839,974	248	0.091	31,788	3,386.995	4.858	1.535	198
2	20,456,165	2,456,730	660	0.162	30,994	3,722.319	14.209	3.983	98
3	12,676,523	2,258,443	396	0.232	32,011	5,703.14	13.062	2.468	62
4	17,082,784	3,132,325	544	0.293	31,402	5,757.951	18.116	3.326	49
5	18,946,293	3,468,512	612	0.36	30,957	5,667.504	20.061	3.689	38
6	21,084,722	3,144,685	720	0.432	29,284	4,367.618	18.188	4.105	28
7	19,540,522	1,989,258	703	0.547	27,795	2,829.671	11.505	3.805	14

Frame: 1
Channel: Chemi
Sensitivity: 100
Molecular Weight Analysis Regression Method : Point to Point

Lane 3 - MCF10A_sfGFP_24H

#	Vol. (Int.)	Local Bg. Corr. Vol.	Area	Rf	Density	Local Bg. Corr. Den.	% band purity	% lane purity	Mol. Wt.
1	8,514,635	7,771,197	492	0.295	17,306	15,795	39.415	31.991	48.656
2	13,102,503	11,944,932	588	0.371	22,283	20,314	60.585	49.228	36.529

Lane 4 - MCF10A_TCPV1_24H

#	Vol. (Int.)	Local Bg. Corr. Vol.	Area	Rf	Density	Local Bg. Corr. Den.	% band purity	% lane purity	Mol. Wt.
1	8,648,332	7,686,036	440	0.301	19,655	17,468	39.918	29.819	47.625
2	13,114,490	11,568,707	504	0.377	26,020	22,953	60.082	45.218	35.647

Lane 5 - MCF10A_TCPV2_24H

#	Vol. (Int.)	Local Bg. Corr. Vol.	Area	Rf	Density	Local Bg. Corr. Den.	% band purity	% lane purity	Mol. Wt.

#	Vol. (Int.)	Local Bg. Corr. Vol.	Area	Rf	Density	Local Bg. Corr. Den.	% band purity	% lane purity	Mol. Wt.
1	8,604,114	7,670,309	440	0.303	19,554	17,432	41.164	30.733	47.281
2	12,382,868	10,963,021	504	0.379	24,569	21,752	58.836	44.23	35.353

Lane 6 - MCF10A_VASH1_24H

#	Vol. (Int.)	Local Bg. Corr. Vol.	Area	Rf	Density	Local Bg. Corr. Den.	% band purity	% lane purity	Mol. Wt.
1	9,311,824	8,377,815	451	0.303	20,647	18,576	41.616	31.63	47.281
2	13,215,941	11,753,477	516	0.379	25,612	22,778	58.384	44.891	35.353

Lane 7 - MCF10A_VASH2_24H

#	Vol. (Int.)	Local Bg. Corr. Vol.	Area	Rf	Density	Local Bg. Corr. Den.	% band purity	% lane purity	Mol. Wt.
1	8,575,944	7,775,360	440	0.301	19,490	17,671	41.836	31.87	47.625
2	12,134,791	10,810,046	546	0.379	22,224	19,798	58.164	45.095	35.353

Lane 8 - MCF10A_SVBP_24H

#	Vol. (Int.)	Local Bg. Corr. Vol.	Area	Rf	Density	Local Bg. Corr. Den.	% band purity	% lane purity	Mol. Wt.
1	8,947,071	8,324,979	492	0.297	18,185	16,920	45.358	36.813	48.312
2	10,843,657	10,029,087	546	0.375	19,860	18,368	54.642	44.616	35.941

iBright™ Image Analysis Report

28 February 2022

Supplemental Figure 3- Tyr-Tub (GAPDH lower band)

CHEMI_02232022_150731_2s_MCF10A_TCP_ALL24H_TYR_GAPDH

Date:	02-23-2022 03:07:31PM
Mode:	Chemi Blots
Notes:	
Model:	FL1500
Instrument name:	2462619090234
Serial No:	2462619090234
Firmware version:	1.6.0
iBA version:	4.0.1
Image size:	563px X 450px
Image area:	
Optical Zoom:	2x
Digital Zoom:	1.2x
Focus level:	455
Resolution:	5 x 5
Exposure time:	2000 ms
Exposure mode:	Normal

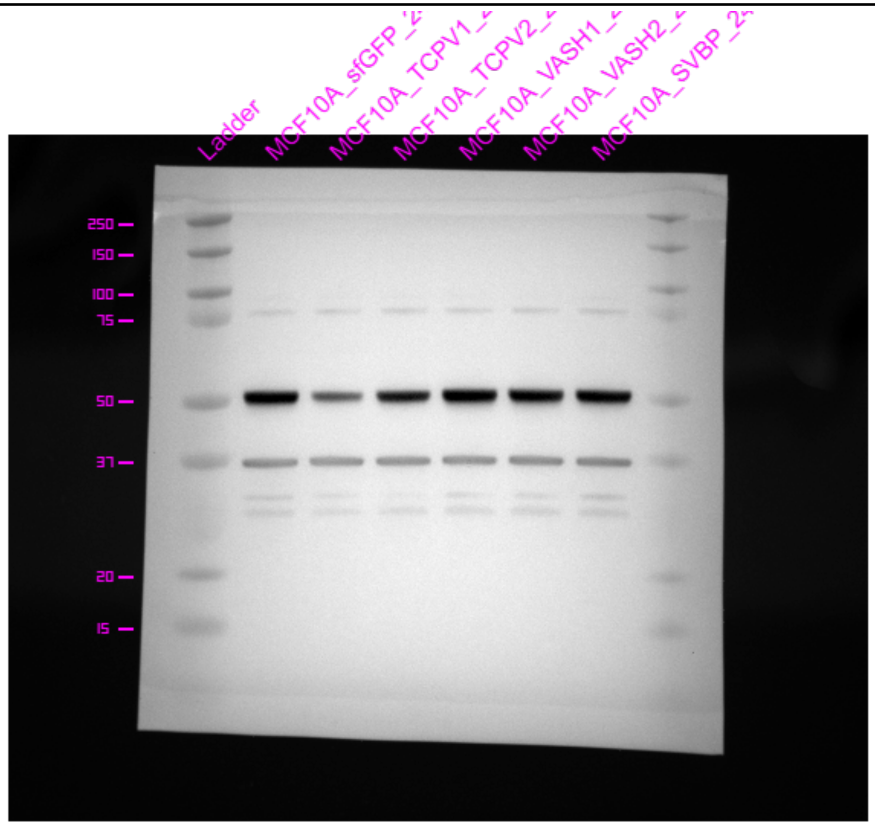

CHEMI_02232022_150731_2s_MCF10A_TCP _ALL24H_TYR_GAPDH

Date:	02-23-2022 03:07:31PM
Mode:	Chemi Blots
Notes:	
Model:	FL1500
Instrument name:	2462619090234
Serial No:	2462619090234
Firmware version:	1.6.0
iBA version:	4.0.1
Image size:	563px X 450px
Image area:	
Optical Zoom:	2x
Digital Zoom:	1.2x
Focus level:	455
Resolution:	5 x 5
Exposure time:	2000 ms
Exposure mode:	Normal

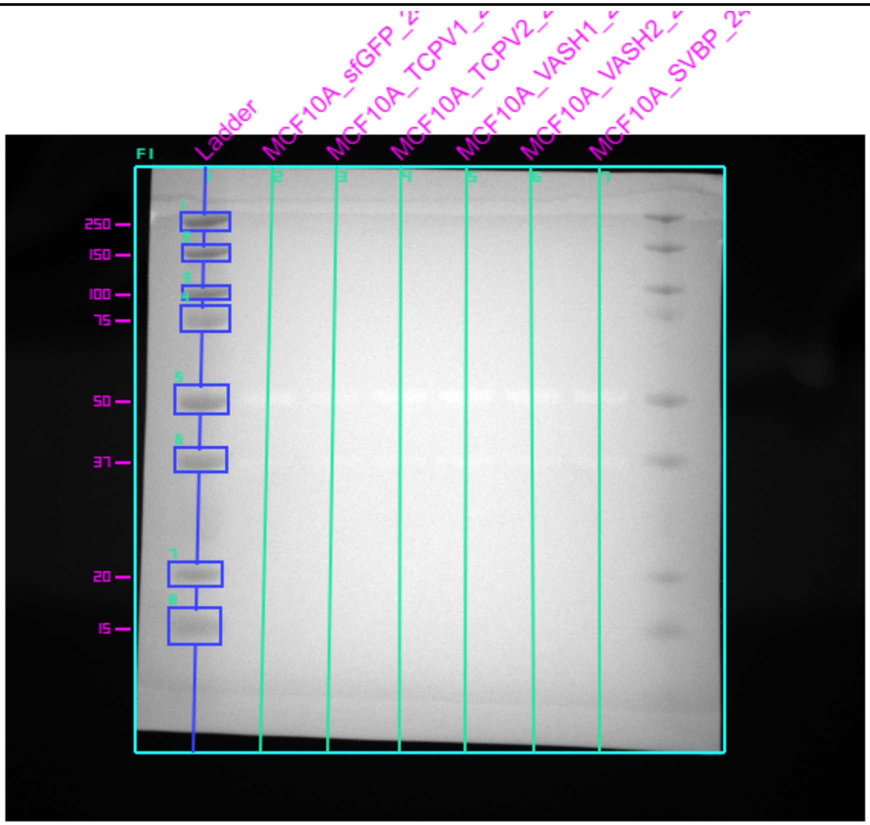

CHEMI_02232022_150731_2s_MCF10A_TCP_ALL24H_TYR_GAPDH

Date:	02-23-2022 03:07:31PM
Mode:	Chemi Blots
Notes:	
Model:	FL1500
Instrument name:	2462619090234
Serial No:	2462619090234
Firmware version:	1.6.0
iBA version:	4.0.1
Image size:	563px X 450px
Image area:	
Optical Zoom:	2x
Digital Zoom:	1.2x
Focus level:	455
Resolution:	5 x 5
Exposure time:	2000 ms
Exposure mode:	Normal

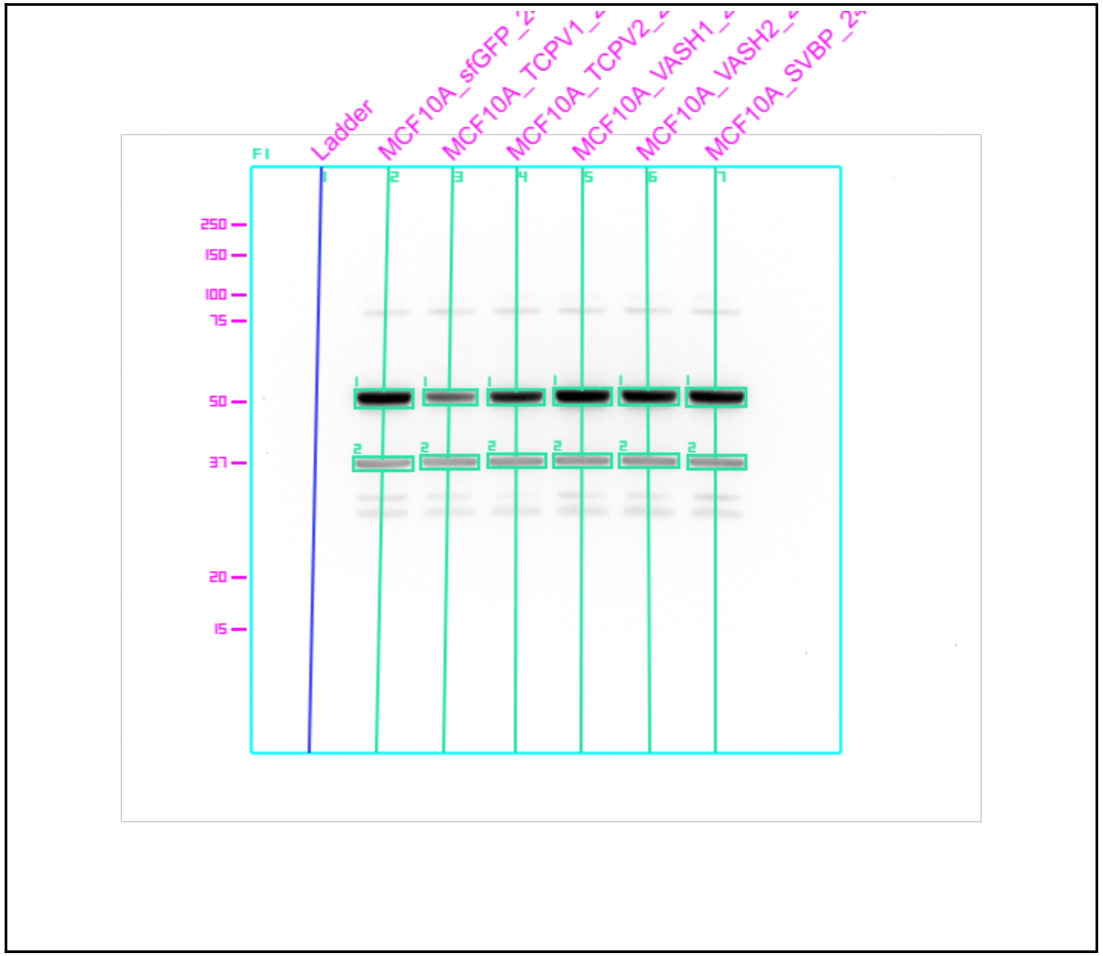

LANE AND BAND ANALYSIS DATA TABLE

CHEMI_02232022_150731_2s_MCF10A_TCP_ALL24H_TYR_GAPDH

Frame: 1
Channel: Membrane
Sensitivity: 100
Molecular Weight Analysis Regression Method : Point to Point

Lane 1 - Ladder

#	Vol. (Int.)	Local Bg. Corr. Vol.	Area	Rf	Density	Local Bg. Corr. Den.	% band purity	% lane purity	Mol. Wt.
1	15,908,618	2,409,811	429	0.094	37,083	5,617.276	15.395	3.548	250
2	13,540,670	1,872,227	384	0.146	35,262	4,875.591	11.96	3.02	150
3	11,157,015	1,756,387	320	0.214	34,865	5,488.71	11.22	2.488	100
4	18,531,395	1,540,390	594	0.258	31,197	2,593.25	9.84	4.133	75
5	22,271,679	2,511,300	720	0.396	30,932	3,487.917	16.043	4.967	50
6	18,444,622	2,033,423	595	0.5	30,999	3,417.518	12.99	4.114	37
7	19,065,960	1,646,260	612	0.695	31,153	2,689.968	10.517	4.252	20
8	27,663,704	1,883,842	875	0.784	31,615	2,152.963	12.035	6.17	15

Frame: 1
Channel: Chemi
Sensitivity: 100
Molecular Weight Analysis Regression Method : Point to Point

Lane 2 - MCF10A_sfGFP_24H

#	Vol. (Int.)	Local Bg. Corr. Vol.	Area	Rf	Density	Local Bg. Corr. Den.	% band purity	% lane purity	Mol. Wt.
1	15,747,029	13,608,329	507	0.396	31,059	26,840	79.217	51.467	50
2	4,404,081	3,570,204	400	0.505	11,010	8,925.512	20.783	14.394	36.547

Lane 3 - MCF10A_TCPV1_24H

#	Vol. (Int.)	Local Bg. Corr. Vol.	Area	Rf	Density	Local Bg. Corr. Den.	% band purity	% lane purity	Mol. Wt.
1	7,141,754	5,848,955	396	0.393	18,034	14,770	63.41	33.163	50.472
2	4,256,223	3,375,102	390	0.503	10,913	8,654.11	36.59	19.764	36.773

Lane 4 - MCF10A_TCPV2_24H

#	Vol. (Int.)	Local Bg. Corr. Vol.	Area	Rf	Density	Local Bg. Corr. Den.	% band purity	% lane purity	Mol. Wt.
1	11,698,326	9,735,328	418	0.393	27,986	23,290	73.154	41.98	50.472
2	4,532,513	3,572,716	390	0.5	11,621	9,160.813	26.846	16.265	37

Lane 5 - MCF10A_VASH1_24H

#	Vol. (Int.)	Local Bg. Corr. Vol.	Area	Rf	Density	Local Bg. Corr. Den.	% band purity	% lane purity	Mol. Wt.
1	15,757,968	12,919,930	468	0.391	33,670	27,606	78.063	46.148	50.943
2	4,701,151	3,630,661	390	0.5	12,054	9,309.39	21.937	13.767	37

Lane 6 - MCF10A_VASH2_24H

#	Vol. (Int.)	Local Bg. Corr. Vol.	Area	Rf	Density	Local Bg. Corr. Den.	% band purity	% lane purity	Mol. Wt.
1	14,795,171	12,034,390	480	0.391	30,823	25,071	76.028	44.897	50.943
2	4,873,989	3,794,604	400	0.5	12,184	9,486.51	23.972	14.791	37

Lane 7 - MCF10A_SVBP_24H

#	Vol. (Int.)	Local Bg. Corr. Vol.	Area	Rf	Density	Local Bg. Corr. Den.	% band purity	% lane purity	Mol. Wt.
1	15,314,985	13,042,162	520	0.393	29,451	25,081	76.997	48.37	50.472
2	4,777,006	3,896,384	390	0.503	12,248	9,990.73	23.003	15.087	36.773

iBright™ Image Analysis Report

28 January 2022

Figure 2C- deTyr-Tub

CHEMI_12012021_122013_100ms_MCF7_24H_TRANS_DETYR_PUCK

Date:	12-01-2021 12:20:13PM
Mode:	Chemi Blots
Notes:	Figure 2C- deTyr-Tub
Model:	FL1500
Instrument name:	2462619090234
Serial No:	2462619090234
Firmware version:	1.6.0
iBA version:	4.0.1
Image size:	676px X 540px
Image area:	
Optical Zoom:	1.9x
Digital Zoom:	1x
Focus level:	430
Resolution:	5 x 5
Exposure time:	100 ms
Exposure mode:	Normal

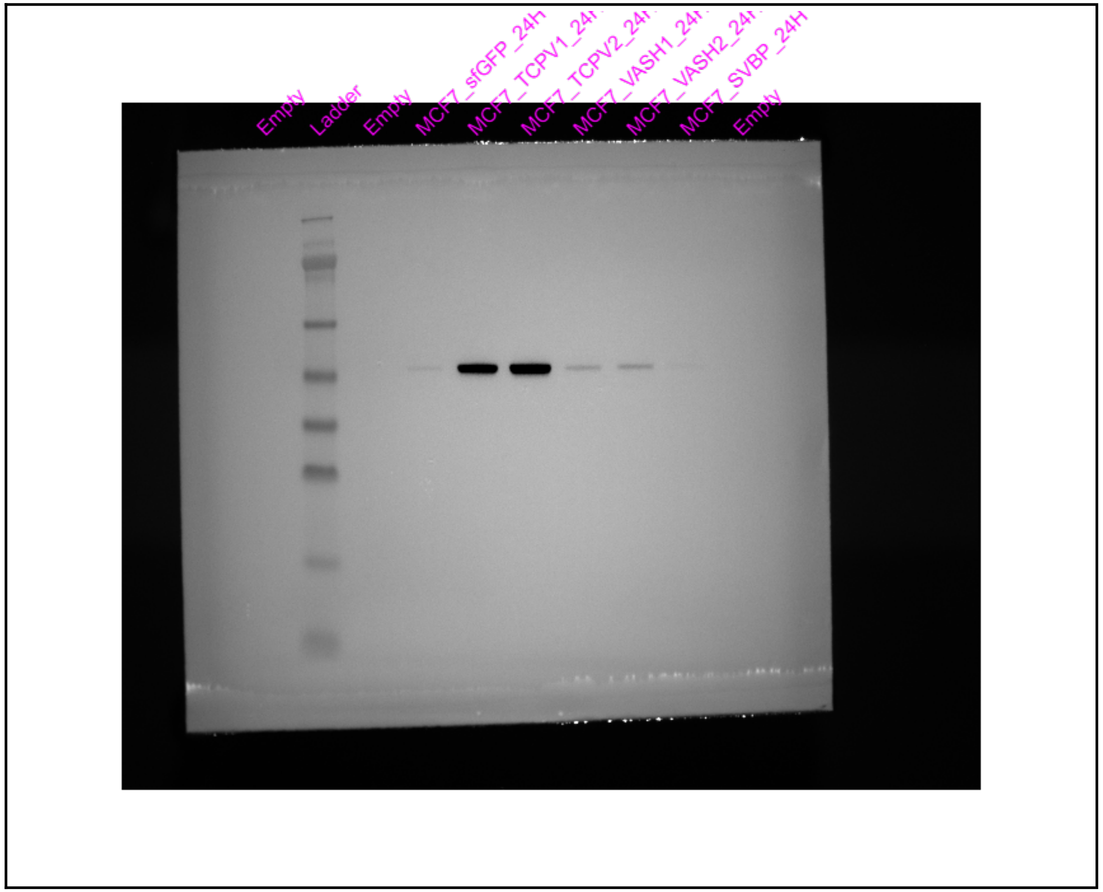

CHEMI_12012021_122013_100ms_MCF7_24H_TRANS_DETYR_PUCK

Date:	12-01-2021 12:20:13PM
Mode:	Chemi Blots
Notes:	Figure 2C- deTyr-Tub
Model:	FL1500
Instrument name:	2462619090234
Serial No:	2462619090234
Firmware version:	1.6.0
iBA version:	4.0.1
Image size:	676px X 540px
Image area:	
Optical Zoom:	1.9x
Digital Zoom:	1x
Focus level:	430
Resolution:	5 x 5
Exposure time:	100 ms
Exposure mode:	Normal

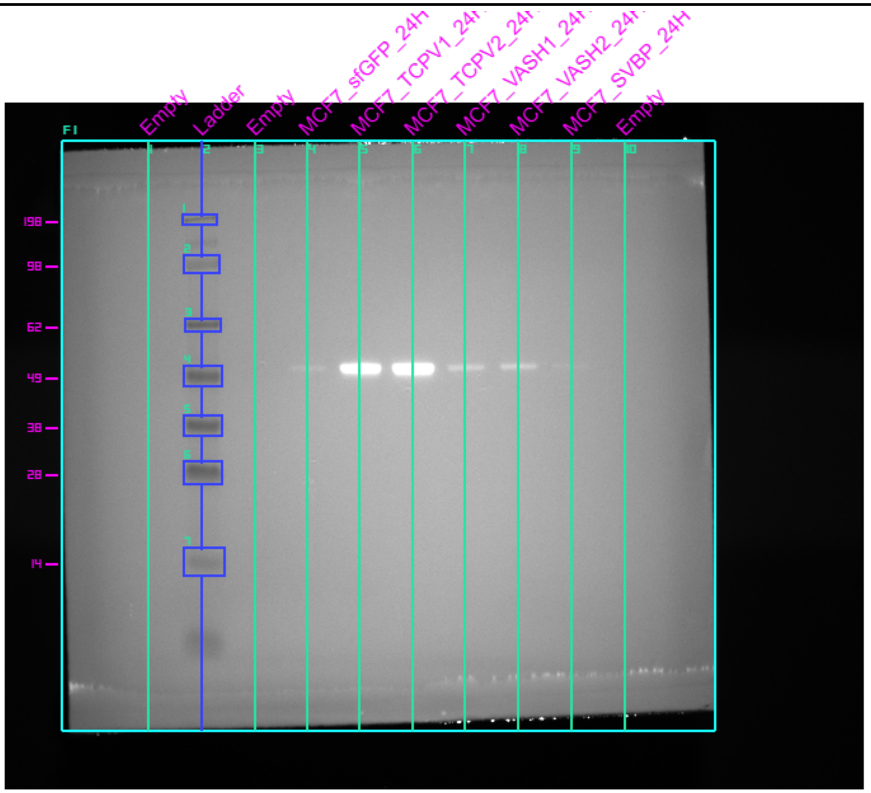

CHEMI_12012021_122013_100ms_MCF7_24H_TRANS_DETYR_PUCK

Date:	12-01-2021 12:20:13PM
Mode:	Chemi Blots
Notes:	Figure 2C- deTyr-Tub
Model:	FL1500
Instrument name:	2462619090234
Serial No:	2462619090234
Firmware version:	1.6.0
iBA version:	4.0.1
Image size:	676px X 540px
Image area:	
Optical Zoom:	1.9x
Digital Zoom:	1x
Focus level:	430
Resolution:	5 x 5
Exposure time:	100 ms
Exposure mode:	Normal

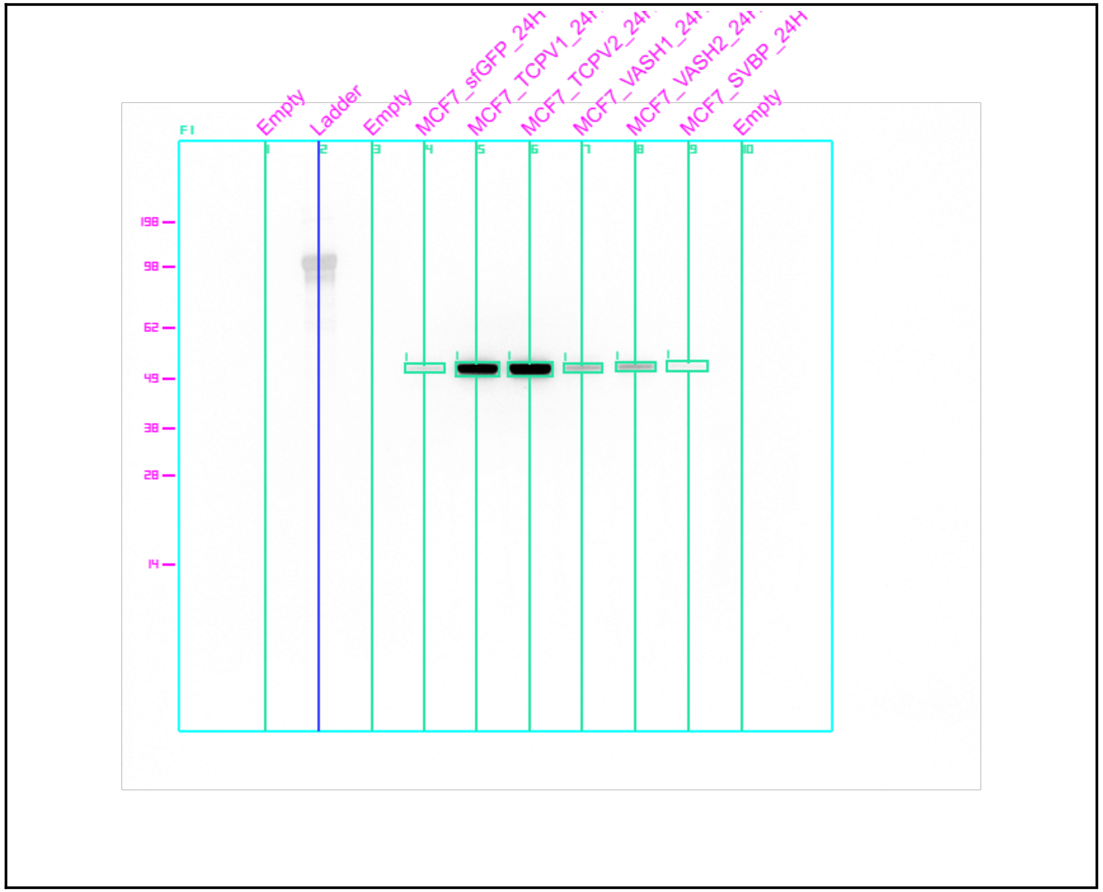

LANE AND BAND ANALYSIS DATA TABLE

CHEMI_12012021_122013_100ms_MCF7_24H_TRANS_DETYR_PUCK

Frame: 1
Channel: Membrane
Sensitivity: 100
Molecular Weight Analysis Regression Method : Point to Point

Lane 2 - Ladder

#	Vol. (Int.)	Local Bg. Corr. Vol.	Area	Rf	Density	Local Bg. Corr. Den.	% band purity	% lane purity	Mol. Wt.
1	7,926,444	638,127	252	0.134	31,454	2,532.253	3.969	1.795	198
2	13,071,799	1,072,019	435	0.209	30,050	2,464.412	6.667	2.96	98
3	10,696,090	1,893,478	319	0.313	33,530	5,935.669	11.776	2.422	62
4	17,466,864	3,097,564	527	0.399	33,143	5,877.732	19.264	3.956	49
5	17,571,424	3,521,160	527	0.483	33,342	6,681.518	21.899	3.979	38
6	19,899,466	4,085,777	589	0.563	33,785	6,936.804	25.41	4.507	28
7	22,644,625	1,771,308	759	0.713	29,834	2,333.739	11.016	5.128	14

Frame: 1
Channel: Chemi
Sensitivity: 100
Molecular Weight Analysis Regression Method : Point to Point

Lane 4 - MCF7_sfGFP_24H

#	Vol. (Int.)	Local Bg. Corr. Vol.	Area	Rf	Density	Local Bg. Corr. Den.	% band purity	% lane purity	Mol. Wt.
1	620,147	395,709	256	0.384	2,422.449	1,545.742	100	15.858	51.275

Lane 5 - MCF7_TCPV1_24H

#	Vol. (Int.)	Local Bg. Corr. Vol.	Area	Rf	Density	Local Bg. Corr. Den.	% band purity	% lane purity	Mol. Wt.
1	9,905,044	8,541,019	420	0.386	23,583	20,335	100	67.433	50.95

Lane 6 - MCF7_TCPV2_24H

#	Vol. (Int.)	Local Bg. Corr. Vol.	Area	Rf	Density	Local Bg. Corr. Den.	% band purity	% lane purity	Mol. Wt.
1	11,731,134	10,152,798	432	0.386	27,155	23,501	100	70.163	50.95

Lane 7 - MCF7_VASH1_24H

#	Vol. (Int.)	Local Bg. Corr. Vol.	Area	Rf	Density	Local Bg. Corr. Den.	% band purity	% lane purity	Mol. Wt.
1	1,281,836	966,071	248	0.384	5,168.694	3,895.449	100	26.569	51.275

Lane 8 - MCF7_VASH2_24H

#	Vol. (Int.)	Local Bg. Corr. Vol.	Area	Rf	Density	Local Bg. Corr. Den.	% band purity	% lane purity	Mol. Wt.
1	1,379,980	1,146,802	256	0.381	5,390.547	4,479.698	100	32.639	51.6

Lane 9 - MCF7_SVBP_24H

#	Vol. (Int.)	Local Bg. Corr. Vol.	Area	Rf	Density	Local Bg. Corr. Den.	% band purity	% lane purity	Mol. Wt.
1	325,573	209,432	297	0.381	1,096.205	705.158	100	12.397	51.6

iBright™ Image Analysis Report

28 January 2022

Figure 2C- Alpha Tubulin

CHEMI_12012021_123201_656ms_MCF7_24H_TRANS_ALPHA_POLY

Date:	12-01-2021 12:32:01PM
Mode:	Chemi Blots
Notes:	Figure2C- Alpha tub
Model:	FL1500
Instrument name:	2462619090234
Serial No:	2462619090234
Firmware version:	1.6.0
iBA version:	4.0.1
Image size:	676px X 540px
Image area:	
Optical Zoom:	1.6x
Digital Zoom:	1x
Focus level:	355
Resolution:	5 x 5
Exposure time:	656 ms
Exposure mode:	Normal

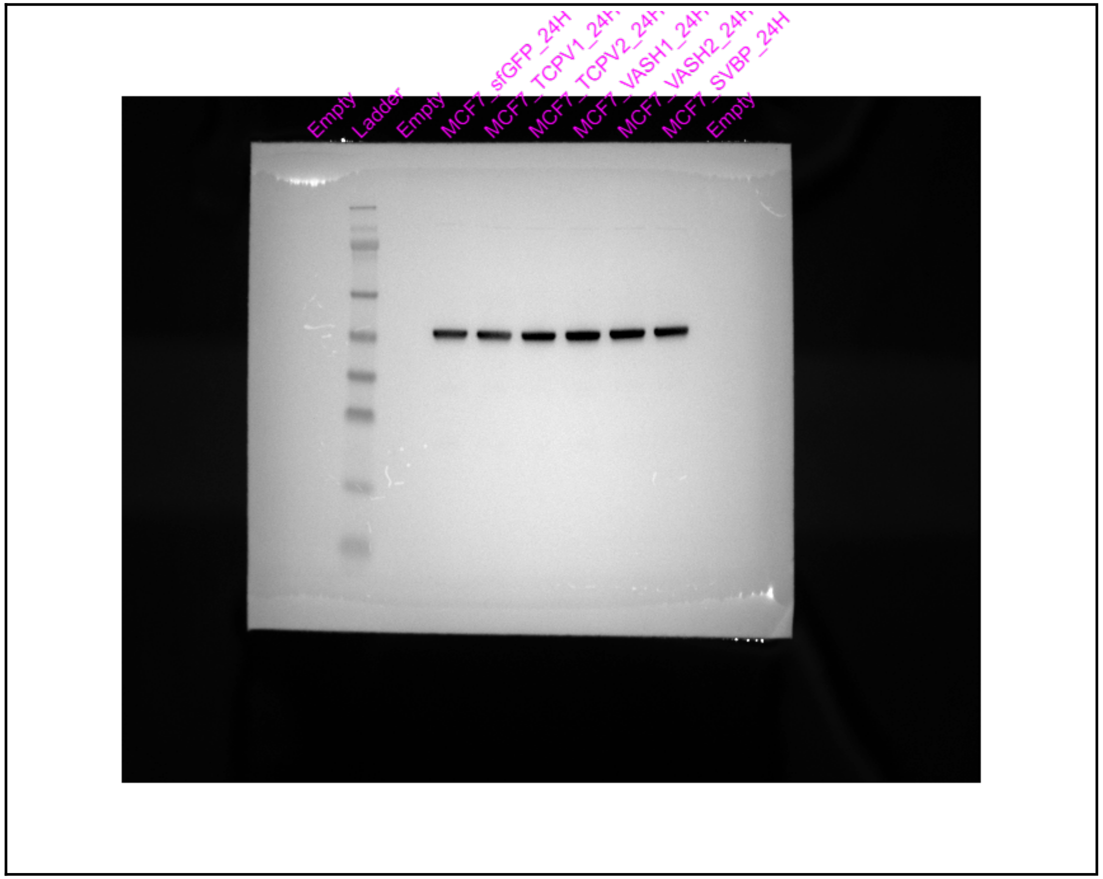

CHEMI_12012021_123201_656ms_MCF7_24H_TRANS_ALPHA_POLY

Date:	12-01-2021 12:32:01PM
Mode:	Chemi Blots
Notes:	Figure2C- Alpha tub
Model:	FL1500
Instrument name:	2462619090234
Serial No:	2462619090234
Firmware version:	1.6.0
iBA version:	4.0.1
Image size:	676px X 540px
Image area:	
Optical Zoom:	1.6x
Digital Zoom:	1x
Focus level:	355
Resolution:	5 x 5
Exposure time:	656 ms
Exposure mode:	Normal

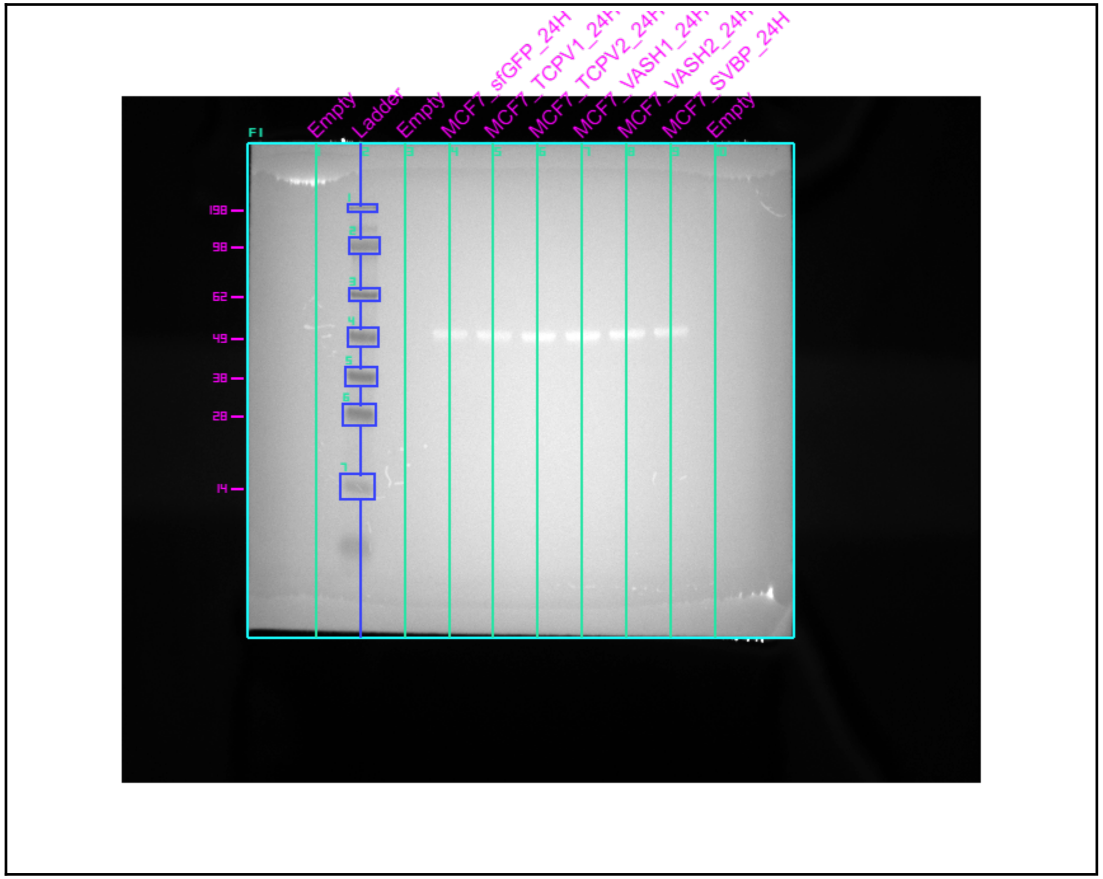

CHEMI_12012021_123201_656ms_MCF7_24H_TRANS_ALPHA_POLY

Date:	12-01-2021 12:32:01PM
Mode:	Chemi Blots
Notes:	Figure2C- Alpha tub
Model:	FL1500
Instrument name:	2462619090234
Serial No:	2462619090234
Firmware version:	1.6.0
iBA version:	4.0.1
Image size:	676px X 540px
Image area:	
Optical Zoom:	1.6x
Digital Zoom:	1x
Focus level:	355
Resolution:	5 x 5
Exposure time:	656 ms
Exposure mode:	Normal

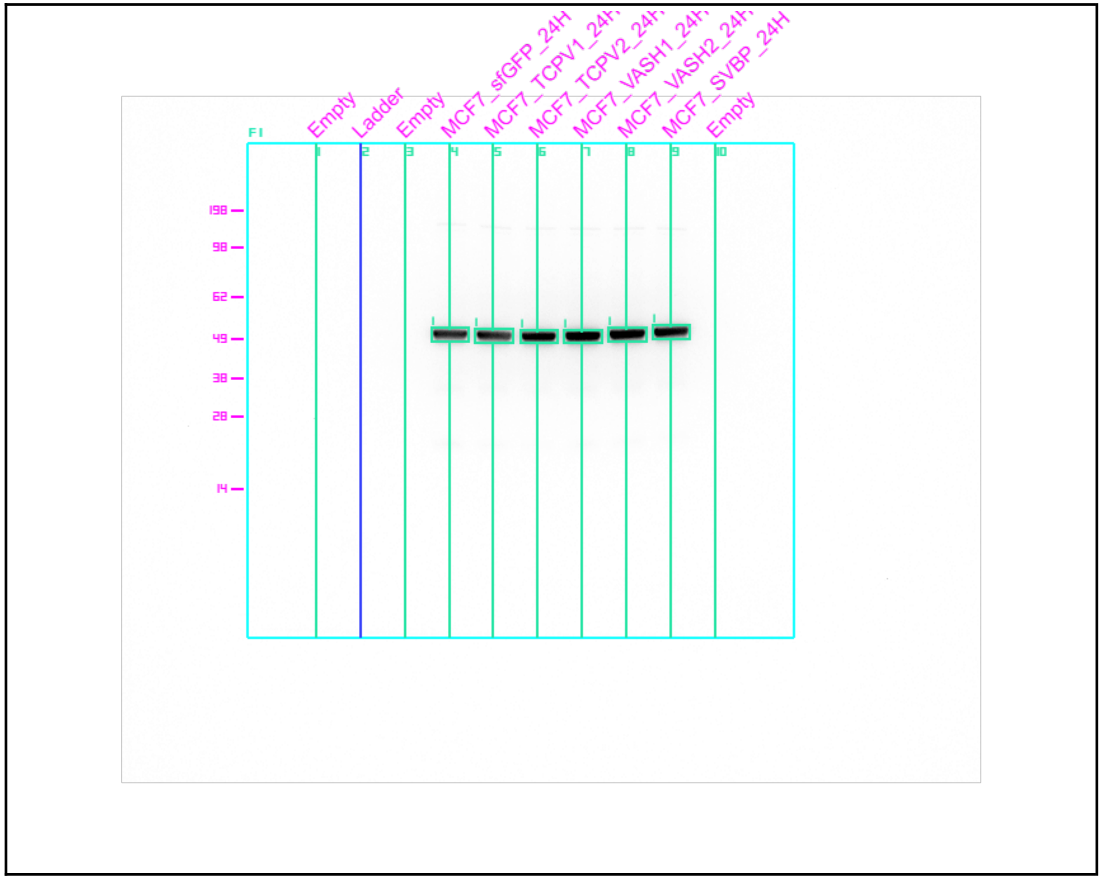

LANE AND BAND ANALYSIS DATA TABLE

CHEMI_12012021_123201_656ms_MCF7_24H_TRANS_ALPHA_POLY

Frame: 1
Channel: Membrane
Sensitivity: 100
Molecular Weight Analysis Regression Method : Point to Point

Lane 2 - Ladder

#	Vol. (Int.)	Local Bg. Corr. Vol.	Area	Rf	Density	Local Bg. Corr. Den.	% band purity	% lane purity	Mol. Wt.
1	5,085,166	529,779	168	0.131	30,268	3,153.448	3.944	1.699	198
2	10,919,134	1,576,851	350	0.206	31,197	4,505.289	11.74	3.648	98
3	8,605,977	1,668,328	275	0.306	31,294	6,066.649	12.421	2.875	62
4	12,260,749	2,379,355	400	0.391	30,651	5,948.388	17.715	4.096	49
5	12,927,656	2,657,547	416	0.47	31,076	6,388.335	19.786	4.319	38
6	14,973,140	3,006,566	486	0.548	30,808	6,186.351	22.384	5.003	28
7	16,107,456	1,613,121	588	0.694	27,393	2,743.403	12.01	5.382	14

Frame: 1
Channel: Chemi
Sensitivity: 100
Molecular Weight Analysis Regression Method : Point to Point

Lane 4 - MCF7_sfGFP_24H

#	Vol. (Int.)	Local Bg. Corr. Vol.	Area	Rf	Density	Local Bg. Corr. Den.	% band purity	% lane purity	Mol. Wt.
1	7,158,891	6,058,301	360	0.386	19,885	16,828	100	59.423	49.788

Lane 5 - MCF7_TCPV1_24H

#	Vol. (Int.)	Local Bg. Corr. Vol.	Area	Rf	Density	Local Bg. Corr. Den.	% band purity	% lane purity	Mol. Wt.
1	7,372,925	6,063,171	372	0.388	19,819	16,298	100	58.048	49.394

Lane 6 - MCF7_TCPV2_24H

#	Vol. (Int.)	Local Bg. Corr. Vol.	Area	Rf	Density	Local Bg. Corr. Den.	% band purity	% lane purity	Mol. Wt.
1	8,558,787	7,071,590	330	0.391	25,935	21,429	100	58.574	49

Lane 7 - MCF7_VASH1_24H

#	Vol. (Int.)	Local Bg. Corr. Vol.	Area	Rf	Density	Local Bg. Corr. Den.	% band purity	% lane purity	Mol. Wt.
1	10,088,941	8,268,313	341	0.391	29,586	24,247	100	59.658	49

Lane 8 - MCF7_VASH2_24H

#	Vol. (Int.)	Local Bg. Corr. Vol.	Area	Rf	Density	Local Bg. Corr. Den.	% band purity	% lane purity	Mol. Wt.
1	10,200,411	8,444,879	372	0.386	27,420	22,701	100	61.988	49.788

Lane 9 - MCF7_SVBP_24H

#	Vol. (Int.)	Local Bg. Corr. Vol.	Area	Rf	Density	Local Bg. Corr. Den.	% band purity	% lane purity	Mol. Wt.
1	8,661,653	7,365,029	360	0.38	24,060	20,458	100	61.522	50.576

iBright™ Image Analysis Report

28 January 2022

Figure 2C- FLAG

CHEMI_12012021_122319_270ms_MCF7_24H_TRANS_FLAG

Date:	12-01-2021 12:23:19PM
Mode:	Chemi Blots
Notes:	Figure 2C- FLAG
Model:	FL1500
Instrument name:	2462619090234
Serial No:	2462619090234
Firmware version:	1.6.0
iBA version:	4.0.1
Image size:	676px X 540px
Image area:	
Optical Zoom:	2x
Digital Zoom:	1x
Focus level:	455
Resolution:	5 x 5
Exposure time:	270 ms
Exposure mode:	Normal

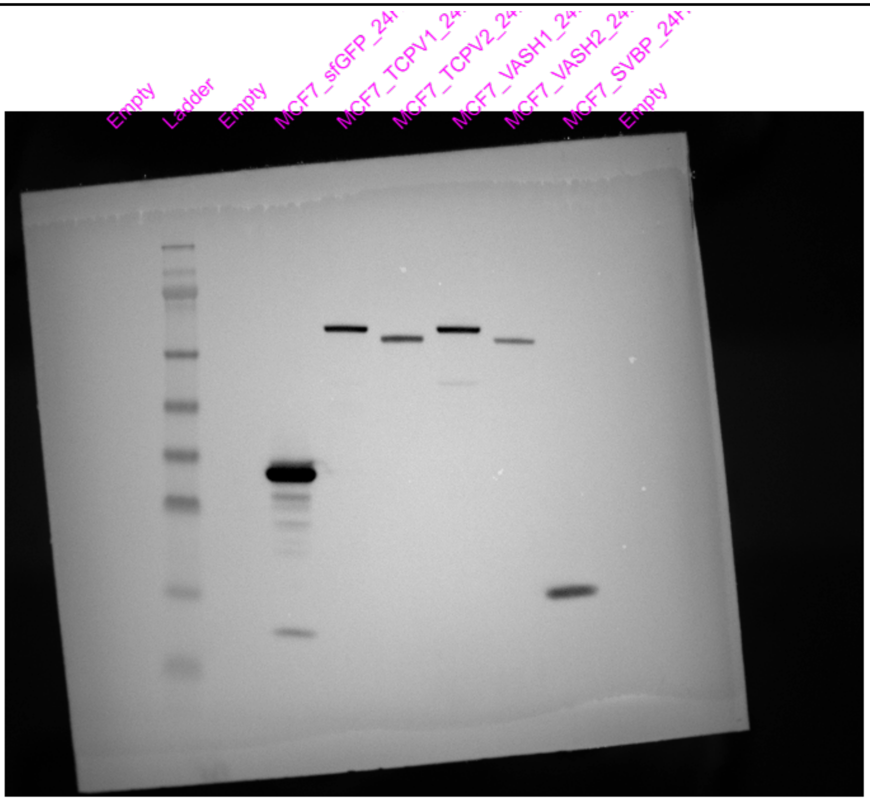

CHEMI_12012021_122319_270ms_MCF7_24H_TRANS_FLAG

Date:	12-01-2021 12:23:19PM
Mode:	Chemi Blots
Notes:	Figure 2C- FLAG
Model:	FL1500
Instrument name:	2462619090234
Serial No:	2462619090234
Firmware version:	1.6.0
iBA version:	4.0.1
Image size:	676px X 540px
Image area:	
Optical Zoom:	2x
Digital Zoom:	1x
Focus level:	455
Resolution:	5 x 5
Exposure time:	270 ms
Exposure mode:	Normal

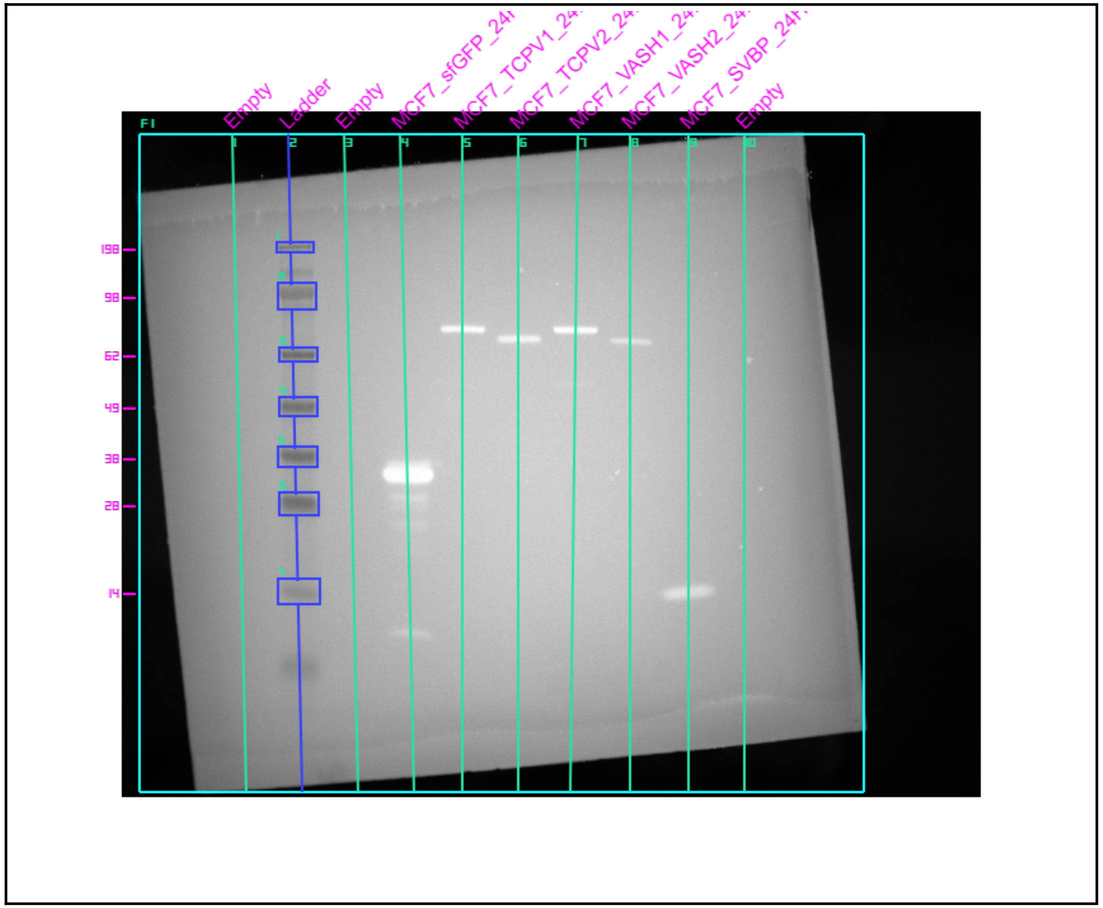

CHEMI_12012021_122319_270ms_MCF7_24H_TRANS_FLAG

Date:	12-01-2021 12:23:19PM
Mode:	Chemi Blots
Notes:	Figure 2C- FLAG
Model:	FL1500
Instrument name:	2462619090234
Serial No:	2462619090234
Firmware version:	1.6.0
iBA version:	4.0.1
Image size:	676px X 540px
Image area:	
Optical Zoom:	2x
Digital Zoom:	1x
Focus level:	455
Resolution:	5 x 5
Exposure time:	270 ms
Exposure mode:	Normal

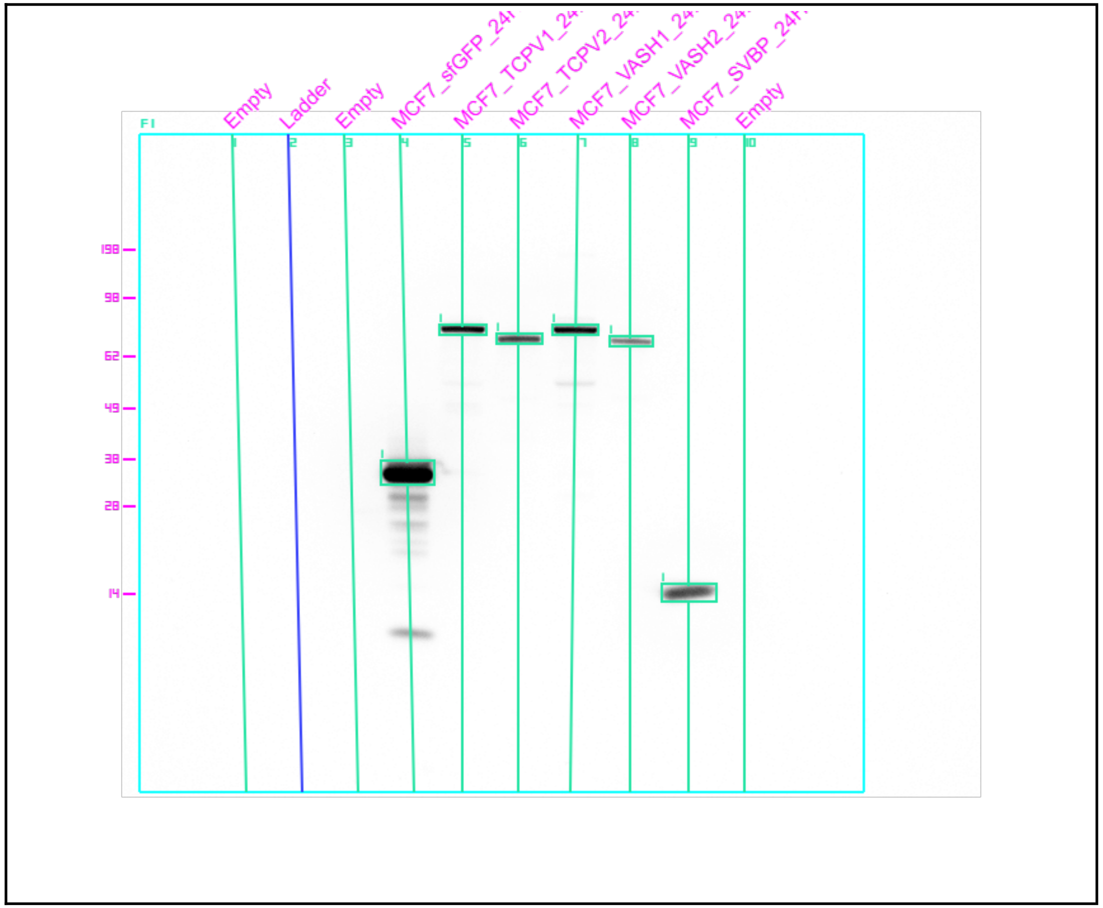

LANE AND BAND ANALYSIS DATA TABLE

CHEMI_12012021_122319_270ms_MCF7_24H_TRANS_FLAG

Frame: 1
Channel: Membrane
Sensitivity: 100
Molecular Weight Analysis Regression Method : Point to Point

Lane 2 - Ladder

#	Vol. (Int.)	Local Bg. Corr. Vol.	Area	Rf	Density	Local Bg. Corr. Den.	% band purity	% lane purity	Mol. Wt.
1	8,970,038	875,388	270	0.172	33,222	3,242.18	4.646	1.539	198
2	22,499,170	2,815,223	682	0.245	32,989	4,127.893	14.941	3.86	98
3	12,815,309	2,447,020	372	0.334	34,449	6,578.011	12.987	2.199	62
4	17,026,033	3,240,415	496	0.413	34,326	6,533.095	17.197	2.921	49
5	18,769,147	3,621,755	544	0.49	34,502	6,657.639	19.221	3.22	38
6	21,112,510	4,007,691	608	0.562	34,724	6,591.598	21.269	3.622	28
7	22,202,698	1,835,122	714	0.695	31,096	2,570.199	9.739	3.81	14

Frame: 1
Channel: Chemi
Sensitivity: 100
Molecular Weight Analysis Regression Method : Point to Point

Lane 4 - MCF7_sfGFP_24H

#	Vol. (Int.)	Local Bg. Corr. Vol.	Area	Rf	Density	Local Bg. Corr. Den.	% band purity	% lane purity	Mol. Wt.
1	26,654,131	22,426,829	860	0.514	30,993	26,077	100	63.263	34.757

Lane 5 - MCF7_TCPV1_24H

#	Vol. (Int.)	Local Bg. Corr. Vol.	Area	Rf	Density	Local Bg. Corr. Den.	% band purity	% lane purity	Mol. Wt.
1	7,200,879	6,723,679	342	0.297	21,055	19,659	100	50.938	76.87

Lane 6 - MCF7_TCPV2_24H

#	Vol. (Int.)	Local Bg. Corr. Vol.	Area	Rf	Density	Local Bg. Corr. Den.	% band purity	% lane purity	Mol. Wt.
1	5,536,343	5,171,802	333	0.311	16,625	15,530	100	57.762	71.391

Lane 7 - MCF7_VASH1_24H

#	Vol. (Int.)	Local Bg. Corr. Vol.	Area	Rf	Density	Local Bg. Corr. Den.	% band purity	% lane purity	Mol. Wt.
1	7,401,391	6,942,075	333	0.297	22,226	20,847	100	62.03	76.87

Lane 8 - MCF7_VASH2_24H

#	Vol. (Int.)	Local Bg. Corr. Vol.	Area	Rf	Density	Local Bg. Corr. Den.	% band purity	% lane purity	Mol. Wt.
1	3,167,951	2,949,324	315	0.315	10,056	9,362.936	100	48.573	69.826

Lane 9 - MCF7_SVBP_24H

#	Vol. (Int.)	Local Bg. Corr. Vol.	Area	Rf	Density	Local Bg. Corr. Den.	% band purity	% lane purity	Mol. Wt.
1	9,723,923	8,949,626	660	0.697	14,733	13,560	100	85.451	NA

iBright™ Image Analysis Report

28 January 2022

Figure 2C- myc-tag

CHEMI_12012021_122716_309ms_MCF7_24H_TRANS_MYC

Date:	12-01-2021 12:27:16PM
Mode:	Chemi Blots
Notes:	Figure2C-myc
Model:	FL1500
Instrument name:	2462619090234
Serial No:	2462619090234
Firmware version:	1.6.0
iBA version:	4.0.1
Image size:	676px X 540px
Image area:	
Optical Zoom:	1.8x
Digital Zoom:	1x
Focus level:	405
Resolution:	5 x 5
Exposure time:	309 ms
Exposure mode:	Normal

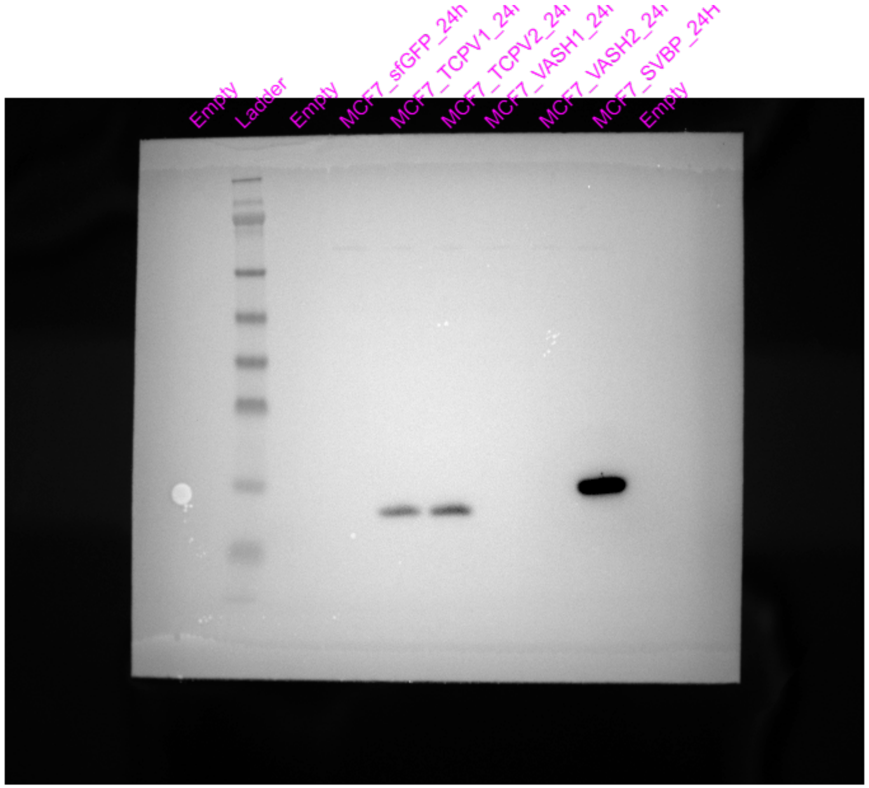

CHEMI_12012021_122716_309ms_MCF7_24H_TRANS_MYC

Date:	12-01-2021 12:27:16PM
Mode:	Chemi Blots
Notes:	Figure2C-myc
Model:	FL1500
Instrument name:	2462619090234
Serial No:	2462619090234
Firmware version:	1.6.0
iBA version:	4.0.1
Image size:	676px X 540px
Image area:	
Optical Zoom:	1.8x
Digital Zoom:	1x
Focus level:	405
Resolution:	5 x 5
Exposure time:	309 ms
Exposure mode:	Normal

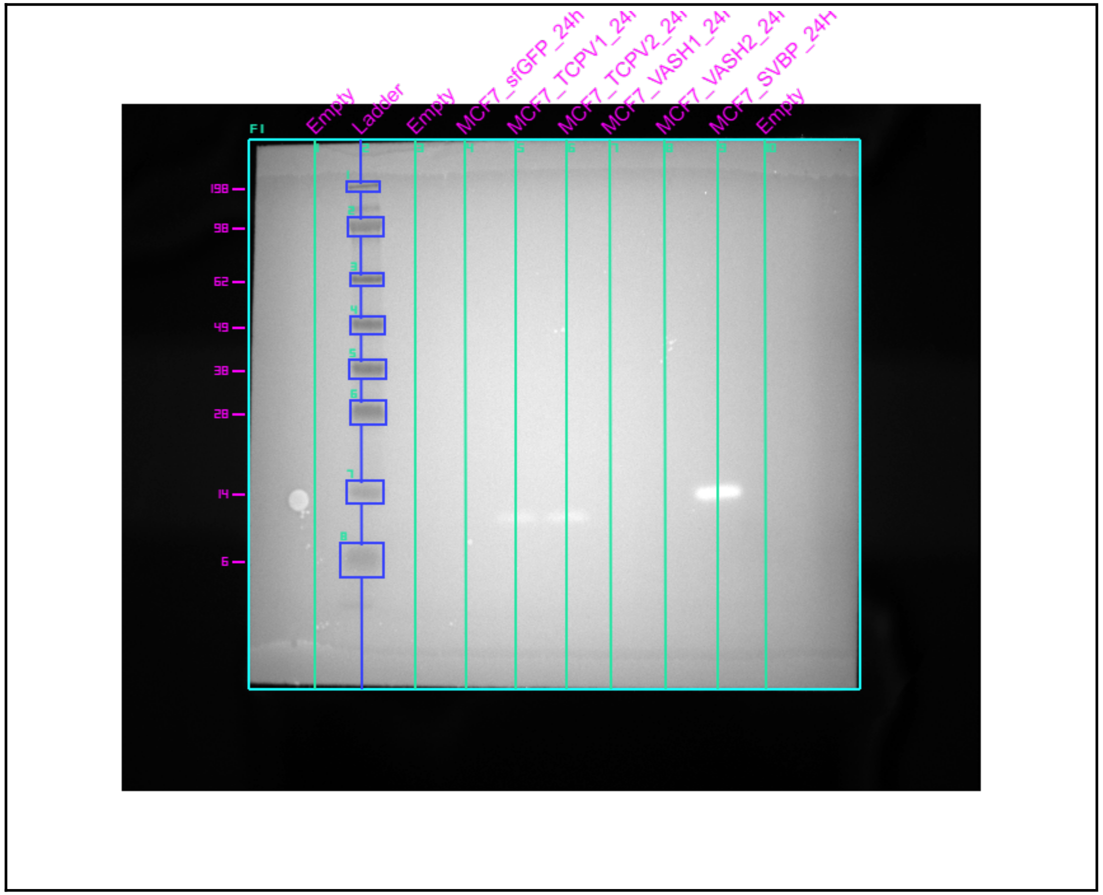

CHEMI_12012021_122716_309ms_MCF7_24H_TRANS_MYC

Date:	12-01-2021 12:27:16PM
Mode:	Chemi Blots
Notes:	Figure2C-myc
Model:	FL1500
Instrument name:	2462619090234
Serial No:	2462619090234
Firmware version:	1.6.0
iBA version:	4.0.1
Image size:	676px X 540px
Image area:	
Optical Zoom:	1.8x
Digital Zoom:	1x
Focus level:	405
Resolution:	5 x 5
Exposure time:	309 ms
Exposure mode:	Normal

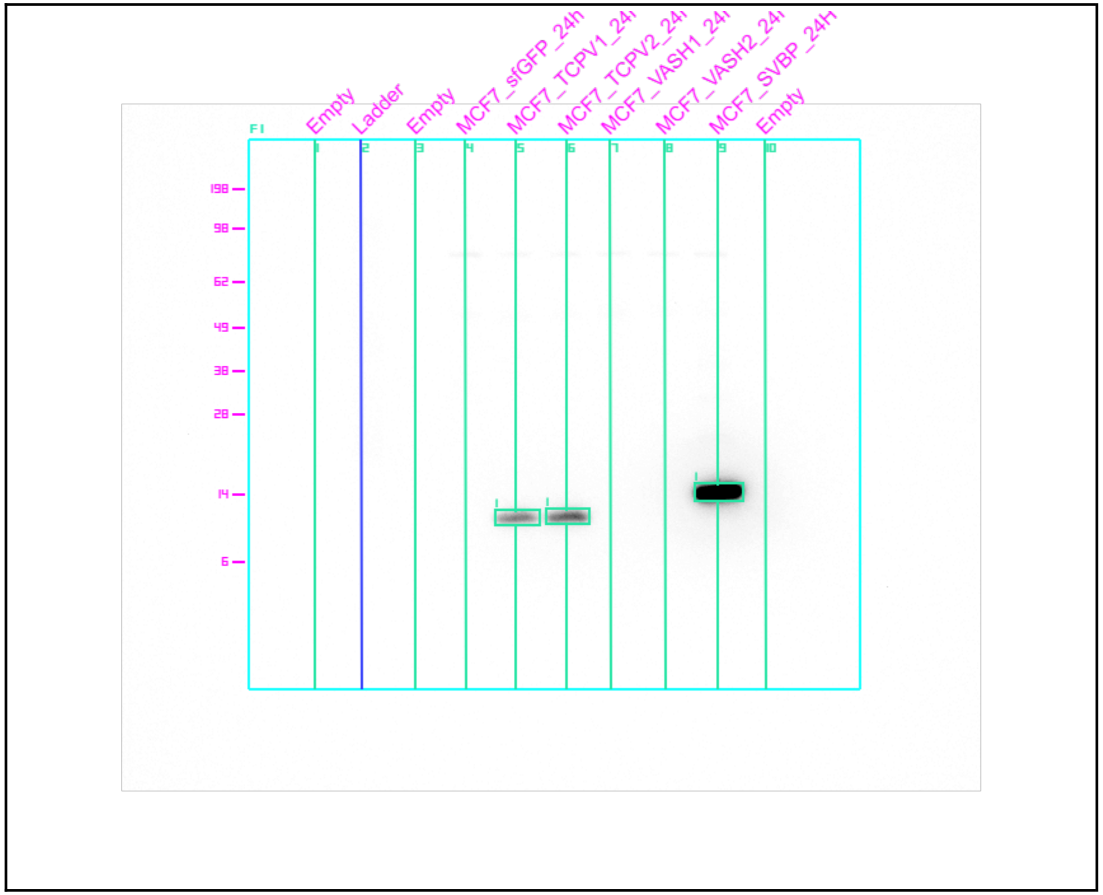

LANE AND BAND ANALYSIS DATA TABLE

CHEMI_12012021_122716_309ms_MCF7_24H_TRANS_MYC

Frame: 1
Channel: Membrane
Sensitivity: 100
Molecular Weight Analysis Regression Method : Point to Point

Lane 2 - Ladder

#	Vol. (Int.)	Local Bg. Corr. Vol.	Area	Rf	Density	Local Bg. Corr. Den.	% band purity	% lane purity	Mol. Wt.
1	8,114,763	743,461	243	0.086	33,394	3,059.511	4.02	1.913	198
2	14,971,195	2,038,501	464	0.157	32,265	4,393.322	11.023	3.529	98
3	9,802,091	2,064,157	297	0.255	33,003	6,950.024	11.161	2.311	62
4	13,365,600	2,711,355	420	0.338	31,822	6,455.608	14.661	3.151	49
5	15,122,292	3,193,679	480	0.417	31,504	6,653.499	17.269	3.565	38
6	17,754,552	3,541,361	580	0.495	30,611	6,105.795	19.149	4.185	28
7	15,767,036	1,703,889	570	0.641	27,661	2,989.281	9.213	3.717	14
8	28,019,399	2,497,498	980	0.764	28,591	2,548.467	13.504	6.605	6

Frame: 1
Channel: Chemi
Sensitivity: 100
Molecular Weight Analysis Regression Method : Point to Point

Lane 5 - MCF7_TCPV1_24H

#	Vol. (Int.)	Local Bg. Corr. Vol.	Area	Rf	Density	Local Bg. Corr. Den.	% band purity	% lane purity	Mol. Wt.
1	2,292,945	1,797,116	468	0.688	4,899.455	3,839.993	100	48.468	10.981

Lane 6 - MCF7_TCPV2_24H

#	Vol. (Int.)	Local Bg. Corr. Vol.	Area	Rf	Density	Local Bg. Corr. Den.	% band purity	% lane purity	Mol. Wt.
1	2,950,651	2,414,078	455	0.685	6,484.947	5,305.668	100	52.246	11.132

Lane 9 - MCF7_SVBP_24H

#	Vol. (Int.)	Local Bg. Corr. Vol.	Area	Rf	Density	Local Bg. Corr. Den.	% band purity	% lane purity	Mol. Wt.
1	16,215,639	13,699,977	585	0.641	27,719	23,418	100	74.359	14

iBright™ Image Analysis Report

28 January 2022

Figure 2C- GAPDH

CHEMI_12022021_121204_5s_817ms_MCF7_24H_TRANS_DETYRBLOT_GAPDH

Date:	12-02-2021 12:12:04PM
Mode:	Chemi Blots
Notes:	Figure 2C-GAPDH on deTyr
Model:	FL1500
Instrument name:	2462619090234
Serial No:	2462619090234
Firmware version:	1.6.0
iBA version:	4.0.1
Image size:	676px X 540px
Image area:	
Optical Zoom:	2x
Digital Zoom:	1x
Focus level:	455
Resolution:	5 x 5
Exposure time:	5817 ms
Exposure mode:	Normal

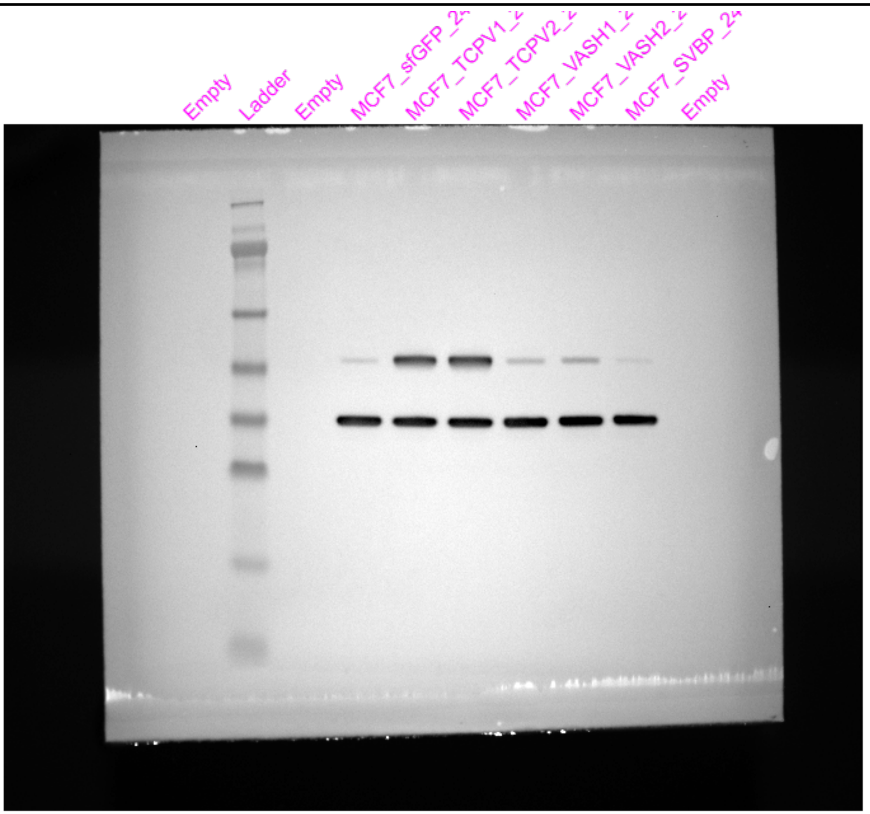

CHEMI_12022021_121204_5s_817ms_MCF7_24H_TRANS_DETYRBLOT_GAPDH

Date:	12-02-2021 12:12:04PM
Mode:	Chemi Blots
Notes:	Figure 2C-GAPDH on deTyr
Model:	FL1500
Instrument name:	2462619090234
Serial No:	2462619090234
Firmware version:	1.6.0
iBA version:	4.0.1
Image size:	676px X 540px
Image area:	
Optical Zoom:	2x
Digital Zoom:	1x
Focus level:	455
Resolution:	5 x 5
Exposure time:	5817 ms
Exposure mode:	Normal

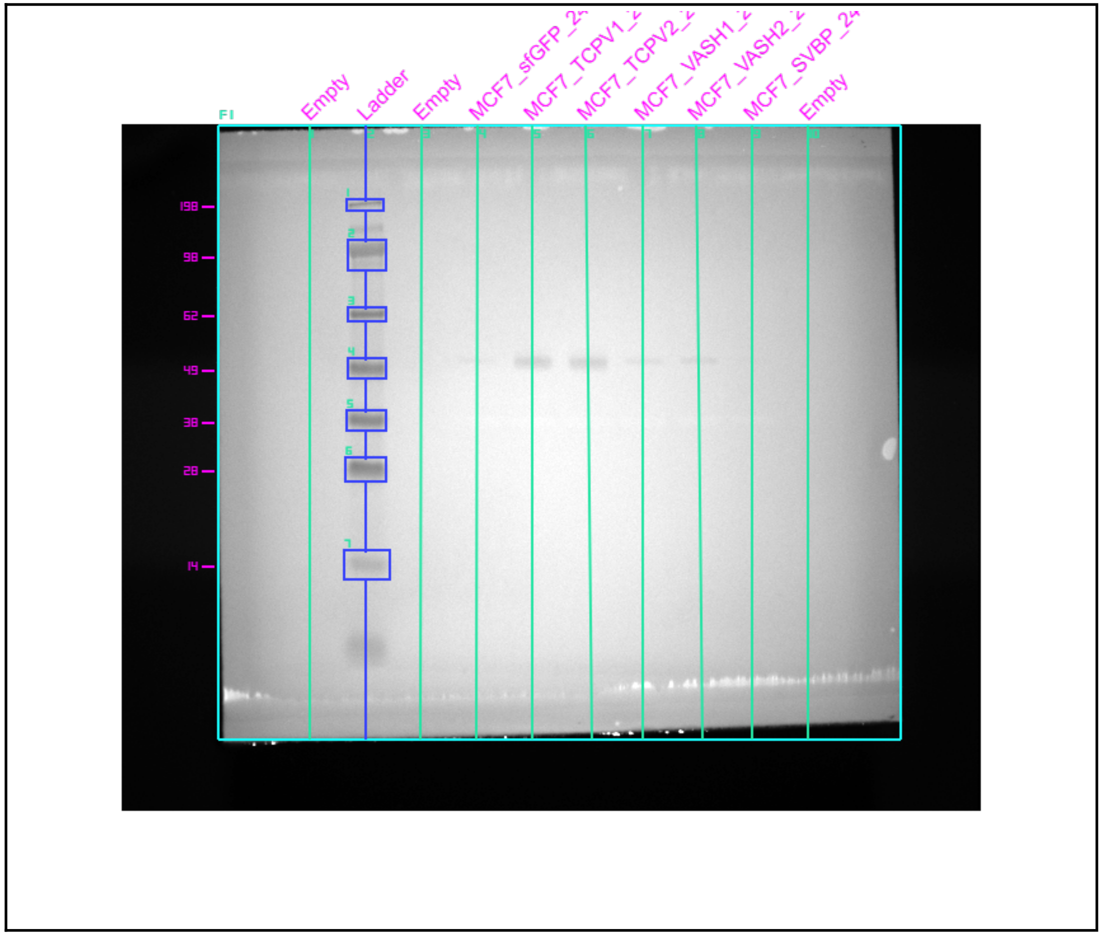

CHEMI_12022021_121204_5s_817ms_MCF7 _24H_TRANS_DETYRBLOT_GAPDH

Date:	12-02-2021 12:12:04PM
Mode:	Chemi Blots
Notes:	Figure 2C-GAPDH on deTyr
Model:	FL1500
Instrument name:	2462619090234
Serial No:	2462619090234
Firmware version:	1.6.0
iBA version:	4.0.1
Image size:	676px X 540px
Image area:	
Optical Zoom:	2x
Digital Zoom:	1x
Focus level:	455
Resolution:	5 x 5
Exposure time:	5817 ms
Exposure mode:	Normal

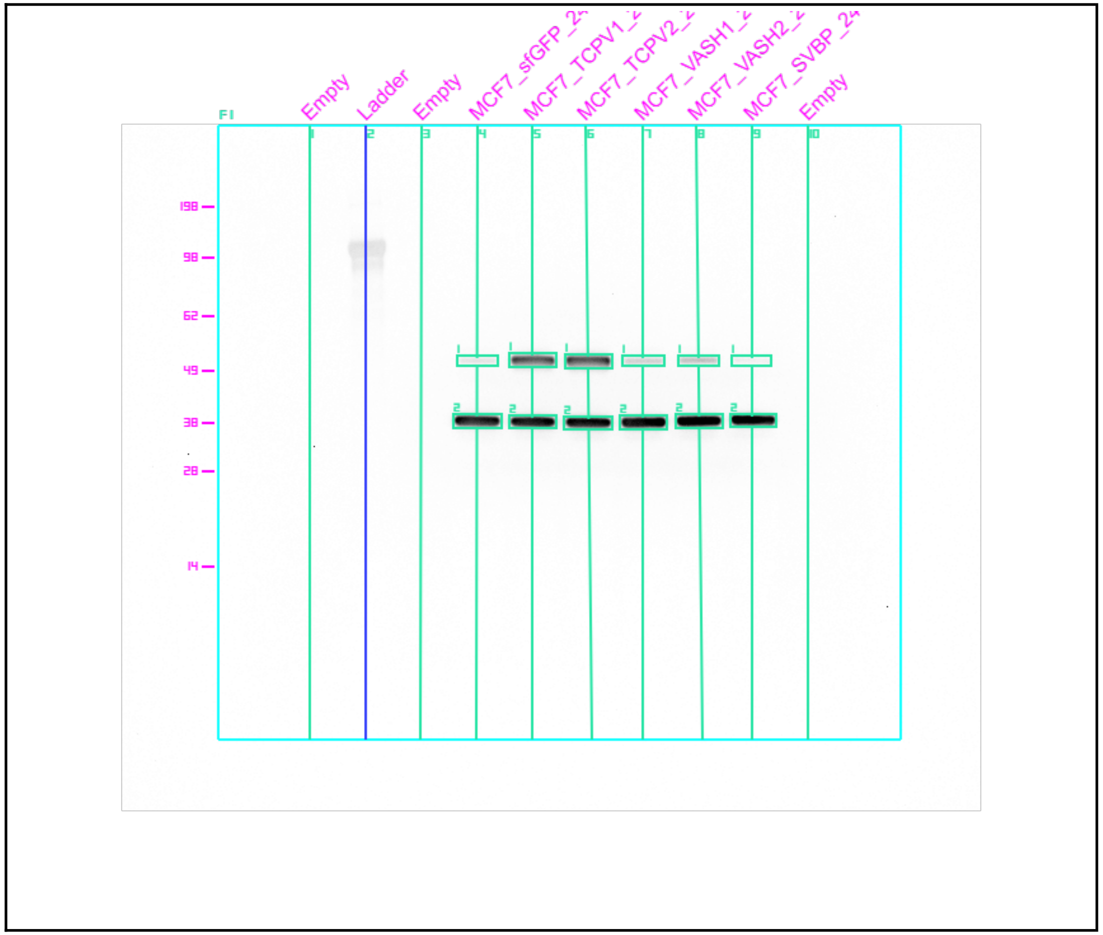

LANE AND BAND ANALYSIS DATA TABLE

CHEMI_12022021_121204_5s_817ms_MCF7_24H_TRANS_DETYRBLOT_GAPDH

Frame: 1
Channel: Membrane
Sensitivity: 100
Molecular Weight Analysis Regression Method : Point to Point

Lane 2 - Ladder

#	Vol. (Int.)	Local Bg. Corr. Vol.	Area	Rf	Density	Local Bg. Corr. Den.	% band purity	% lane purity	Mol. Wt.
1	9,260,505	932,844	300	0.128	30,868	3,109.481	4.139	1.901	198
2	24,501,420	3,912,999	775	0.211	31,614	5,049.031	17.361	5.03	98
3	12,266,990	2,561,216	372	0.306	32,975	6,884.989	11.363	2.518	62
4	17,214,289	3,713,244	527	0.395	32,664	7,046.005	16.475	3.534	49
5	17,638,288	4,156,262	544	0.48	32,423	7,640.189	18.44	3.621	38
6	21,280,439	5,033,500	660	0.559	32,243	7,626.516	22.332	4.368	28
7	24,582,984	2,229,230	888	0.714	27,683	2,510.395	9.89	5.046	14

Frame: 1
Channel: Chemi
Sensitivity: 100
Molecular Weight Analysis Regression Method : Point to Point

Lane 4 - MCF7_sfGFP_24H

#	Vol. (Int.)	Local Bg. Corr. Vol.	Area	Rf	Density	Local Bg. Corr. Den.	% band purity	% lane purity	Mol. Wt.
1	728,357	492,795	297	0.383	2,452.38	1,659.245	4.913	4.527	50.814
2	10,392,503	9,537,353	468	0.48	22,206	20,378	95.087	64.599	38

Lane 5 - MCF7_TCPV1_24H

#	Vol. (Int.)	Local Bg. Corr. Vol.	Area	Rf	Density	Local Bg. Corr. Den.	% band purity	% lane purity	Mol. Wt.
1	7,131,131	6,362,093	456	0.381	15,638	13,951	40.717	29.95	51.116
2	10,235,458	9,263,106	418	0.482	24,486	22,160	59.283	42.988	37.737

Lane 6 - MCF7_TCPV2_24H

#	Vol. (Int.)	Local Bg. Corr. Vol.	Area	Rf	Density	Local Bg. Corr. Den.	% band purity	% lane purity	Mol. Wt.

#	Vol. (Int.)	Local Bg. Corr. Vol.	Area	Rf	Density	Local Bg. Corr. Den.	% band purity	% lane purity	Mol. Wt.
1	7,779,706	6,880,670	456	0.383	17,060	15,089	42.023	30.975	50.814
2	10,557,297	9,492,953	429	0.484	24,609	22,128	57.977	42.034	37.474

Lane 7 - MCF7_VASH1_24H

#	Vol. (Int.)	Local Bg. Corr. Vol.	Area	Rf	Density	Local Bg. Corr. Den.	% band purity	% lane purity	Mol. Wt.
1	1,273,093	958,179	306	0.383	4,160.435	3,131.305	8.29	6.714	50.814
2	11,554,835	10,599,442	456	0.482	25,339	23,244	91.71	60.937	37.737

Lane 8 - MCF7_VASH2_24H

#	Vol. (Int.)	Local Bg. Corr. Vol.	Area	Rf	Density	Local Bg. Corr. Den.	% band purity	% lane purity	Mol. Wt.
1	1,567,633	1,285,602	297	0.383	5,278.226	4,328.626	9.851	7.79	50.814
2	12,743,531	11,765,088	456	0.48	27,946	25,800	90.149	63.325	38

Lane 9 - MCF7_SVBP_24H

#	Vol. (Int.)	Local Bg. Corr. Vol.	Area	Rf	Density	Local Bg. Corr. Den.	% band purity	% lane purity	Mol. Wt.
1	451,647	248,990	288	0.383	1,568.219	864.549	2.242	2.605	50.814
2	11,652,240	10,854,427	407	0.48	28,629	26,669	97.758	67.217	38

iBright™ Image Analysis Report

28 February 2022

Supplemental Figure 3- Tyr-Tub (GAPDH lower band) MCF7s

CHEMI_02232022_151419_2s_MCF7_TCP_A LL24H_TYR_GAPDH

Date:	02-23-2022 03:14:19PM
Mode:	Chemi Blots
Notes:	
Model:	FL1500
Instrument name:	2462619090234
Serial No:	2462619090234
Firmware version:	1.6.0
iBA version:	4.0.1
Image size:	520px X 415px
Image area:	
Optical Zoom:	2x
Digital Zoom:	1.3x
Focus level:	455
Resolution:	5 x 5
Exposure time:	2000 ms
Exposure mode:	Normal

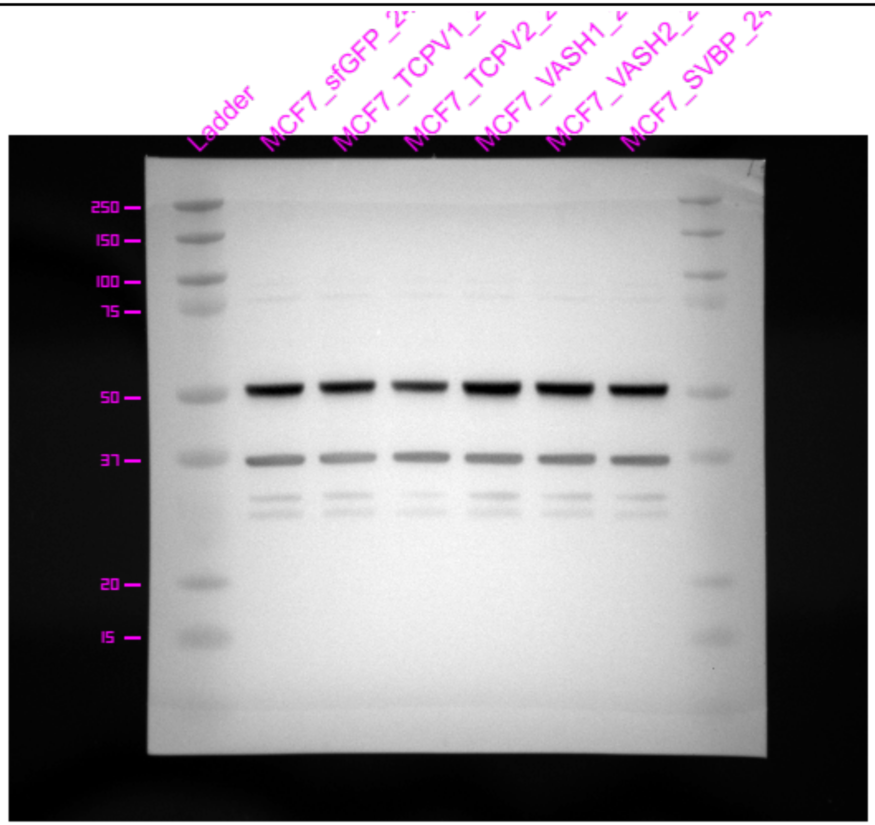

CHEMI_02232022_151419_2s_MCF7_TCP_A LL24H_TYR_GAPDH

Date:	02-23-2022 03:14:19PM
Mode:	Chemi Blots
Notes:	
Model:	FL1500
Instrument name:	2462619090234
Serial No:	2462619090234
Firmware version:	1.6.0
iBA version:	4.0.1
Image size:	520px X 415px
Image area:	
Optical Zoom:	2x
Digital Zoom:	1.3x
Focus level:	455
Resolution:	5 x 5
Exposure time:	2000 ms
Exposure mode:	Normal

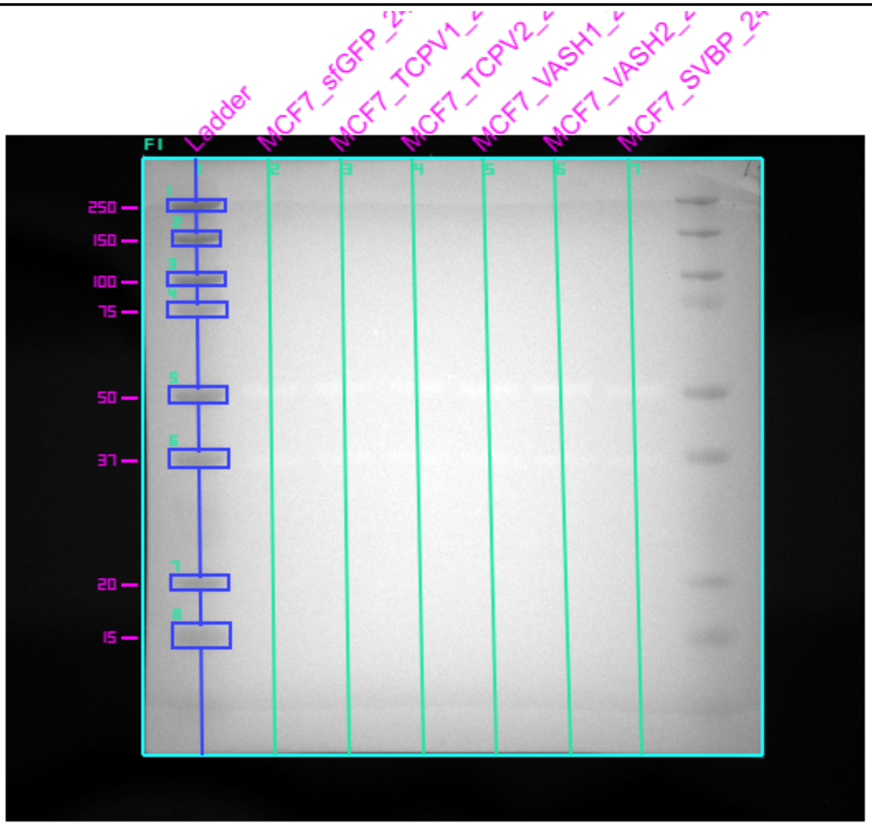

CHEMI_02232022_151419_2s_MCF7_TCP_ALL24H_TYR_GAPDH

Date:	02-23-2022 03:14:19PM
Mode:	Chemi Blots
Notes:	
Model:	FL1500
Instrument name:	2462619090234
Serial No:	2462619090234
Firmware version:	1.6.0
iBA version:	4.0.1
Image size:	520px X 415px
Image area:	
Optical Zoom:	2x
Digital Zoom:	1.3x
Focus level:	455
Resolution:	5 x 5
Exposure time:	2000 ms
Exposure mode:	Normal

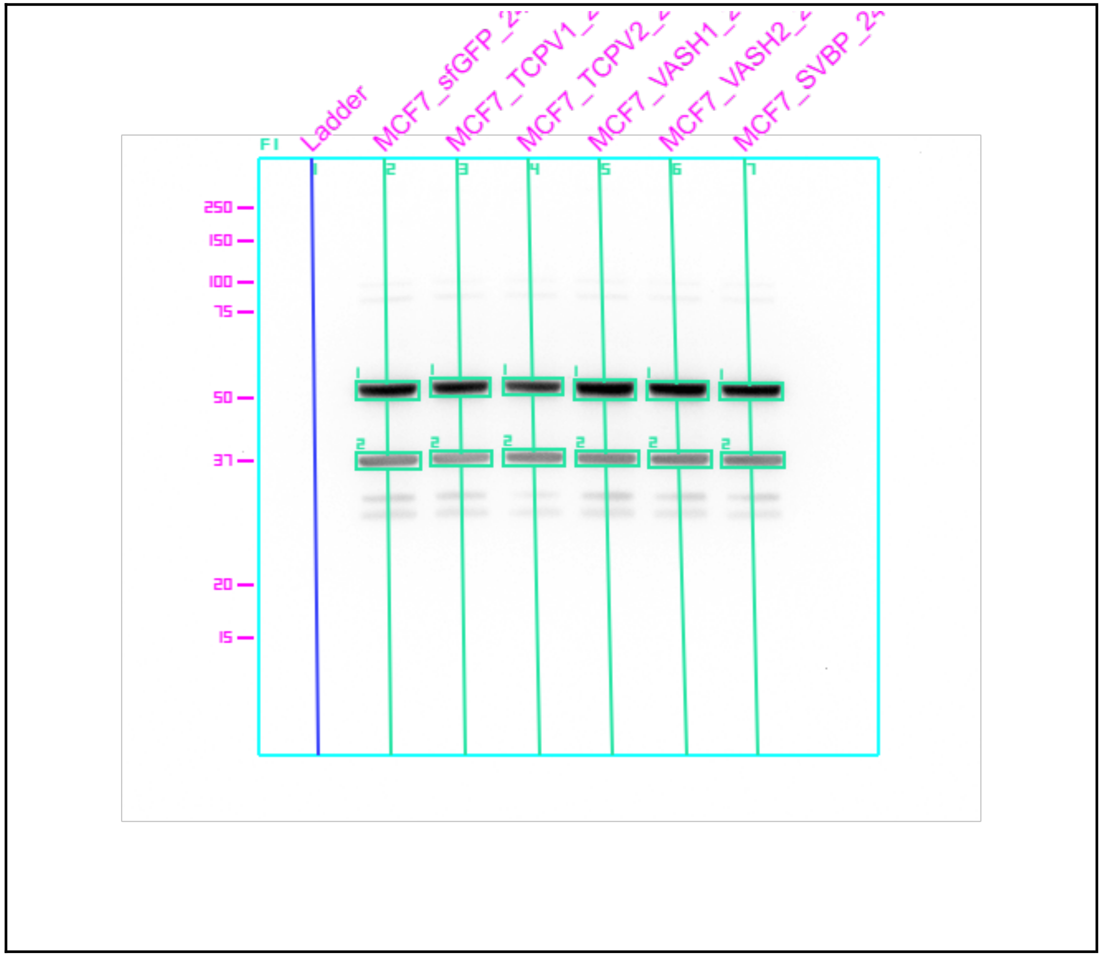

LANE AND BAND ANALYSIS DATA TABLE

CHEMI_02232022_151419_2s_MCF7_TCP_ALL24H_TYR_GAPDH

Frame: 1
Channel: Membrane
Sensitivity: 100
Molecular Weight Analysis Regression Method : Point to Point

Lane 1 - Ladder

#	Vol. (Int.)	Local Bg. Corr. Vol.	Area	Rf	Density	Local Bg. Corr. Den.	% band purity	% lane purity	Mol. Wt.
1	11,289,003	1,658,736	288	0.078	39,197	5,759.502	13.498	NA	250
2	11,267,012	1,530,742	300	0.133	37,556	5,102.474	12.456	NA	150
3	11,611,486	1,654,387	324	0.202	35,837	5,106.135	13.463	NA	100
4	11,977,472	1,007,652	370	0.252	32,371	2,723.384	8.2	NA	75
5	13,199,358	1,841,940	396	0.396	33,331	4,651.365	14.989	NA	50
6	14,253,753	1,735,918	444	0.501	32,103	3,909.725	14.126	NA	37
7	11,669,363	1,195,708	360	0.709	32,414	3,321.412	9.73	NA	20
8	18,678,022	1,663,649	576	0.798	32,427	2,888.28	13.538	NA	15

Frame: 1
Channel: Chemi
Sensitivity: 100
Molecular Weight Analysis Regression Method : Point to Point

Lane 2 - MCF7_sfGFP_24H

#	Vol. (Int.)	Local Bg. Corr. Vol.	Area	Rf	Density	Local Bg. Corr. Den.	% band purity	% lane purity	Mol. Wt.
1	12,288,363	10,513,655	468	0.388	26,257	22,465	68.264	NA	51.442
2	5,902,576	4,887,751	440	0.507	13,414	11,108	31.736	NA	36.547

Lane 3 - MCF7_TCPV1_24H

#	Vol. (Int.)	Local Bg. Corr. Vol.	Area	Rf	Density	Local Bg. Corr. Den.	% band purity	% lane purity	Mol. Wt.
1	11,133,423	9,464,810	444	0.382	25,075	21,317	71.875	NA	52.404
2	4,592,313	3,703,552	380	0.501	12,085	9,746.19	28.125	NA	37

Lane 4 - MCF7_TCPV2_24H

#	Vol. (Int.)	Local Bg. Corr. Vol.	Area	Rf	Density	Local Bg. Corr. Den.	% band purity	% lane purity	Mol. Wt.
1	9,074,853	7,592,171	407	0.382	22,296	18,653	63.26	NA	52.404
2	5,336,006	4,409,337	418	0.501	12,765	10,548	36.74	NA	37

Lane 5 - MCF7_VASH1_24H

#	Vol. (Int.)	Local Bg. Corr. Vol.	Area	Rf	Density	Local Bg. Corr. Den.	% band purity	% lane purity	Mol. Wt.
1	14,577,800	12,395,164	494	0.388	29,509	25,091	73.229	NA	51.442
2	5,573,338	4,531,454	390	0.501	14,290	11,619	26.771	NA	37

Lane 6 - MCF7_VASH2_24H

#	Vol. (Int.)	Local Bg. Corr. Vol.	Area	Rf	Density	Local Bg. Corr. Den.	% band purity	% lane purity	Mol. Wt.
1	13,755,963	11,478,760	456	0.388	30,166	25,172	70.393	NA	51.442
2	5,963,371	4,827,974	429	0.504	13,900	11,254	29.607	NA	36.773

Lane 7 - MCF7_SVBP_24H

#	Vol. (Int.)	Local Bg. Corr. Vol.	Area	Rf	Density	Local Bg. Corr. Den.	% band purity	% lane purity	Mol. Wt.
1	12,296,397	10,385,329	429	0.391	28,662	24,208	68.635	NA	50.962
2	5,712,351	4,745,909	390	0.504	14,647	12,168	31.365	NA	36.773

iBright™ Image Analysis Report

28 January 2022

Figure 5A- deTyr-Tub

CHEMI_11042021_143426_10s_10A_VARIANTS_DETYR_TUB_PUCKOHI

Date:	11-04-2021 02:34:26PM
Mode:	Chemi Blots
Notes:	Figure5A- deTyr-Tub
Model:	FL1500
Instrument name:	2462619090234
Serial No:	2462619090234
Firmware version:	1.6.0
iBA version:	4.0.1
Image size:	676px X 540px
Image area:	
Optical Zoom:	2x
Digital Zoom:	1x
Focus level:	455
Resolution:	5 x 5
Exposure time:	10000 ms
Exposure mode:	Normal

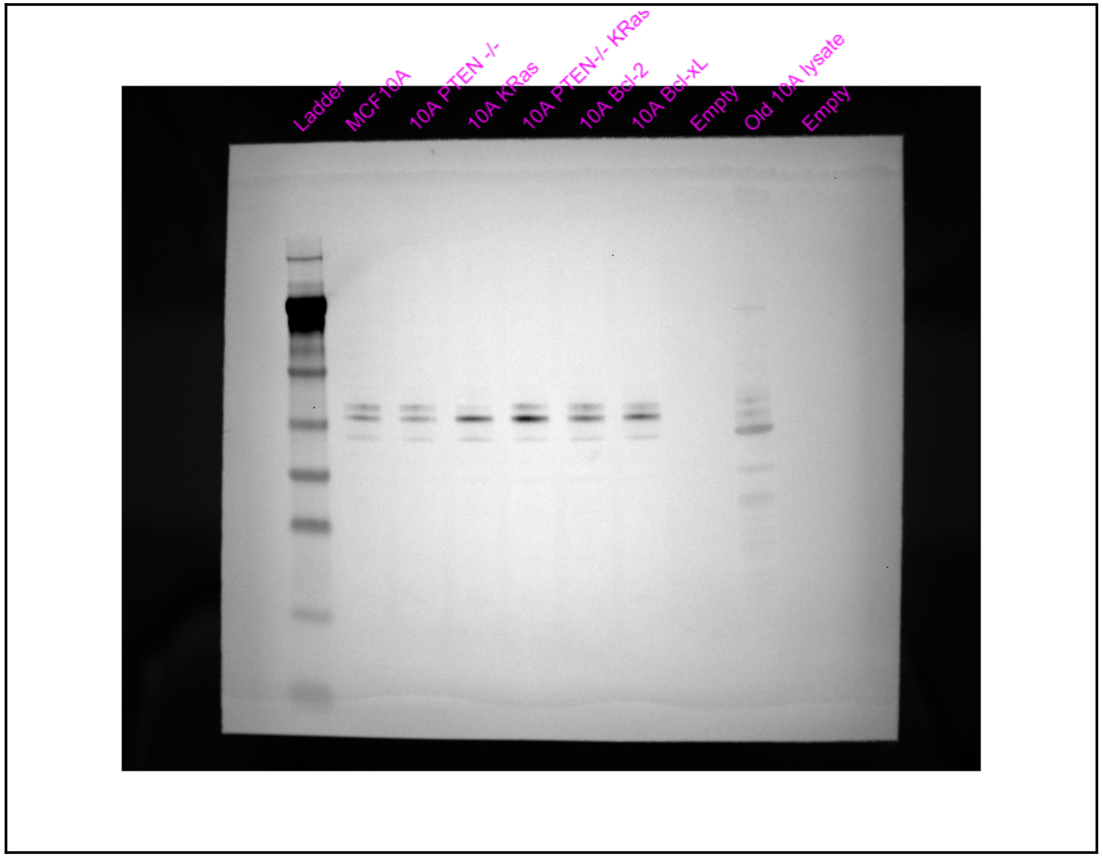

CHEMI_11042021_143426_10s_10A_VARIANTS_DETYR_TUB_PUCKOHI

Date:	11-04-2021 02:34:26PM
Mode:	Chemi Blots
Notes:	Figure5A- deTyr-Tub
Model:	FL1500
Instrument name:	2462619090234
Serial No:	2462619090234
Firmware version:	1.6.0
iBA version:	4.0.1
Image size:	676px X 540px
Image area:	
Optical Zoom:	2x
Digital Zoom:	1x
Focus level:	455
Resolution:	5 x 5
Exposure time:	10000 ms
Exposure mode:	Normal

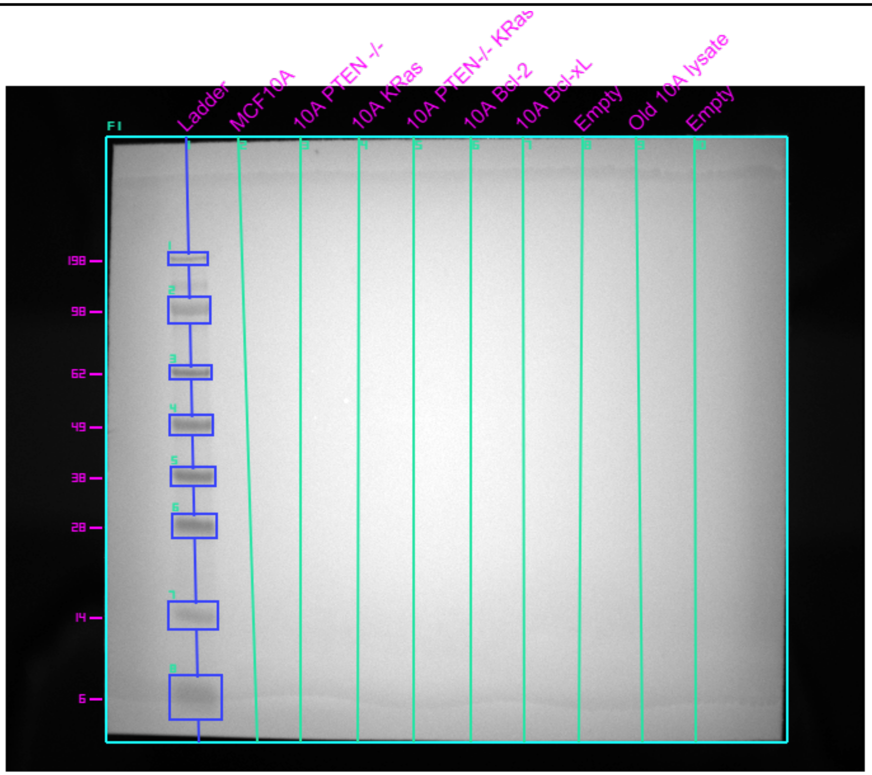

CHEMI_11042021_143426_10s_10A_VARIANTS_DETYR_TUB_PUCKOHI

Date:	11-04-2021 02:34:26PM
Mode:	Chemi Blots
Notes:	Figure5A- deTyr-Tub
Model:	FL1500
Instrument name:	2462619090234
Serial No:	2462619090234
Firmware version:	1.6.0
iBA version:	4.0.1
Image size:	676px X 540px
Image area:	
Optical Zoom:	2x
Digital Zoom:	1x
Focus level:	455
Resolution:	5 x 5
Exposure time:	10000 ms
Exposure mode:	Normal

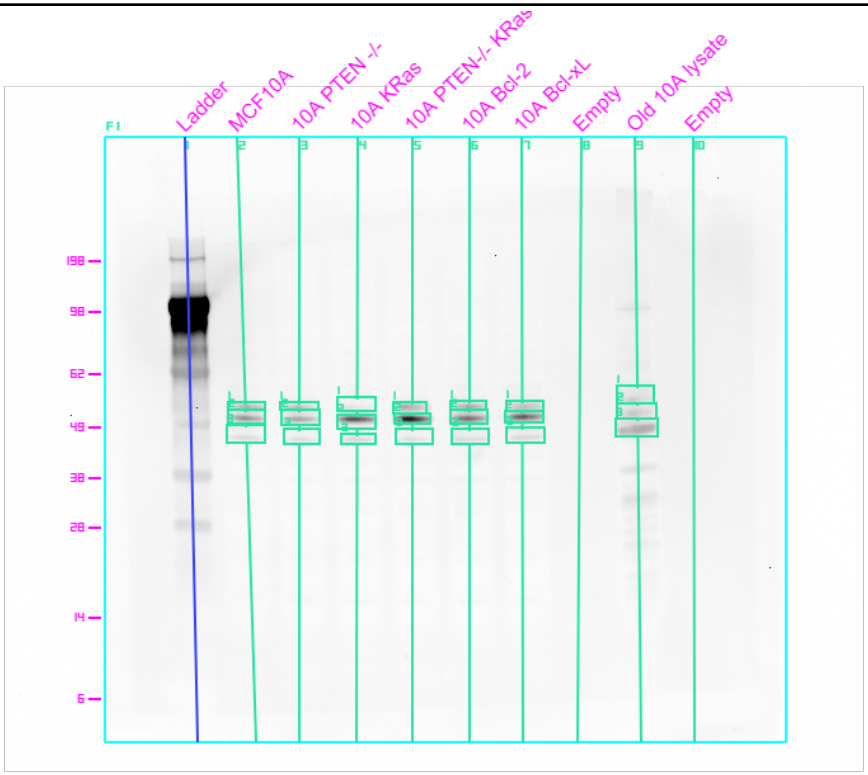

LANE AND BAND ANALYSIS DATA TABLE

CHEMI_11042021_143426_10s_10A_VARIANTS_DETYR_TUB_PUCKOHI

Frame: 1
Channel: Membrane
Sensitivity: 100
Molecular Weight Analysis Regression Method : Point to Point

Lane 1 - Ladder

#	Vol. (Int.)	Local Bg. Corr. Vol.	Area	Rf	Density	Local Bg. Corr. Den.	% band purity	% lane purity	Mol. Wt.
1	10,915,954	826,484	352	0.201	31,011	2,347.967	3.65	2.465	198
2	23,042,204	2,290,125	748	0.285	30,805	3,061.666	10.114	5.204	98
3	13,549,215	2,450,179	408	0.388	33,208	6,005.343	10.821	3.06	62
4	19,695,828	3,469,288	595	0.476	33,102	5,830.737	15.321	4.448	49
5	19,662,939	3,734,224	576	0.56	34,137	6,483.029	16.491	4.441	38
6	24,423,501	4,304,093	720	0.642	33,921	5,977.908	19.008	5.516	28
7	29,775,015	2,140,862	920	0.79	32,364	2,327.024	9.455	6.725	14
8	53,608,673	3,428,073	1,512	0.925	35,455	2,267.244	15.139	12.107	6

Frame: 1
Channel: Chemi
Sensitivity: 100
Molecular Weight Analysis Regression Method : Point to Point

Lane 2 - MCF10A

#	Vol. (Int.)	Local Bg. Corr. Vol.	Area	Rf	Density	Local Bg. Corr. Den.	% band purity	% lane purity	Mol. Wt.
1	2,341,088	1,170,079	210	0.444	11,148	5,571.808	33.169	5.812	53.643
2	4,155,696	2,107,399	360	0.461	11,543	5,853.889	59.74	10.317	51.167
3	2,011,852	250,125	465	0.491	4,326.563	537.904	7.091	4.995	47.075

Lane 3 - 10A PTEN -/-

#	Vol. (Int.)	Local Bg. Corr. Vol.	Area	Rf	Density	Local Bg. Corr. Den.	% band purity	% lane purity	Mol. Wt.
1	1,957,774	982,659	217	0.444	9,022	4,528.383	28.785	6.149	53.643
2	3,870,853	1,943,227	403	0.463	9,605.094	4,821.904	56.923	12.157	50.857
3	1,532,319	487,891	377	0.495	4,064.507	1,294.143	14.292	4.812	46.525

Lane 4 - 10A KRas

#	Vol. (Int.)	Local Bg. Corr. Vol.	Area	Rf	Density	Local Bg. Corr. Den.	% band purity	% lane purity	Mol. Wt.
1	1,683,222	39,685	372	0.44	4,524.79	106.682	0.804	5.135	54.262
2	5,759,402	4,253,645	372	0.47	15,482	11,434	86.195	17.57	49.929
3	1,326,291	641,593	252	0.499	5,263.06	2,546.004	13.001	4.046	45.975

Lane 5 - 10A PTEN-/- KRas

#	Vol. (Int.)	Local Bg. Corr. Vol.	Area	Rf	Density	Local Bg. Corr. Den.	% band purity	% lane purity	Mol. Wt.
1	3,082,160	298,188	270	0.447	11,415	1,104.403	5.213	8.72	53.333
2	6,839,668	5,187,030	261	0.465	26,205	19,873	90.673	19.351	50.548
3	1,643,351	235,355	390	0.495	4,213.721	603.477	4.114	4.649	46.525

Lane 6 - 10A Bcl-2

#	Vol. (Int.)	Local Bg. Corr. Vol.	Area	Rf	Density	Local Bg. Corr. Den.	% band purity	% lane purity	Mol. Wt.
1	2,752,779	1,222,344	232	0.442	11,865	5,268.728	24.248	7.635	53.952
2	5,697,179	3,302,235	360	0.461	15,825	9,172.877	65.507	15.802	51.167
3	1,958,464	516,452	390	0.495	5,021.703	1,324.237	10.245	5.432	46.525

Lane 7 - 10A Bcl-xL

#	Vol. (Int.)	Local Bg. Corr. Vol.	Area	Rf	Density	Local Bg. Corr. Den.	% band purity	% lane purity	Mol. Wt.
1	2,786,426	470,954	270	0.444	10,320	1,744.277	10.176	7.622	53.643
2	5,535,245	3,556,703	310	0.461	17,855	11,473	76.853	15.141	51.167
3	2,062,441	600,291	403	0.493	5,117.72	1,489.556	12.971	5.642	46.8

Lane 9 - Old 10A lysate

#	Vol. (Int.)	Local Bg. Corr. Vol.	Area	Rf	Density	Local Bg. Corr. Den.	% band purity	% lane purity	Mol. Wt.
1	3,214,248	1,006,040	450	0.426	7,142.773	2,235.646	20.418	6.369	56.429
2	3,421,606	729,786	416	0.453	8,225.014	1,754.294	14.811	6.78	52.405
3	5,835,284	3,191,509	510	0.48	11,441	6,257.861	64.771	11.563	48.45

iBright™ Image Analysis Report

28 January 2022

Figure 5A- Alpha Tubulin

CHEMI_08272021_144328_2s_10A_VARIANTS_ALPHA_POLY

Date:	08-27-2021 02:43:28PM
Mode:	Chemi Blots
Notes:	Figure5A- alpha
Model:	FL1500
Instrument name:	2462619090234
Serial No:	2462619090234
Firmware version:	1.6.0
iBA version:	4.0.1
Image size:	676px X 540px
Image area:	
Optical Zoom:	2x
Digital Zoom:	1x
Focus level:	455
Resolution:	5 x 5
Exposure time:	2000 ms
Exposure mode:	Normal

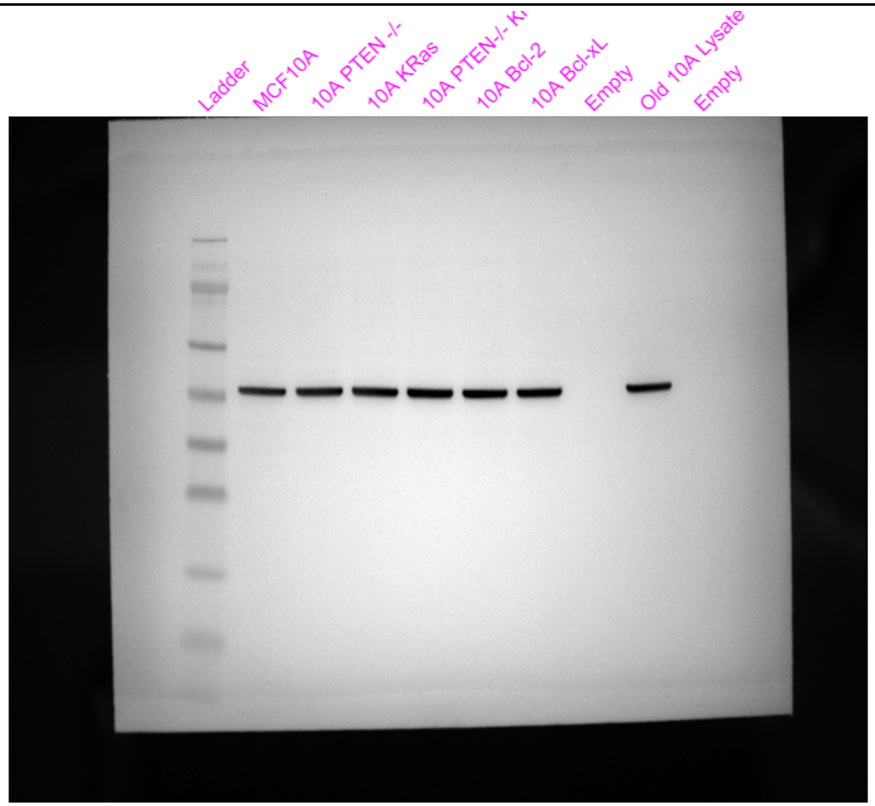

CHEMI_08272021_144328_2s_10A_VARIANTS_ALPHA_POLY

Date:	08-27-2021 02:43:28PM
Mode:	Chemi Blots
Notes:	Figure5A- alpha
Model:	FL1500
Instrument name:	2462619090234
Serial No:	2462619090234
Firmware version:	1.6.0
iBA version:	4.0.1
Image size:	676px X 540px
Image area:	
Optical Zoom:	2x
Digital Zoom:	1x
Focus level:	455
Resolution:	5 x 5
Exposure time:	2000 ms
Exposure mode:	Normal

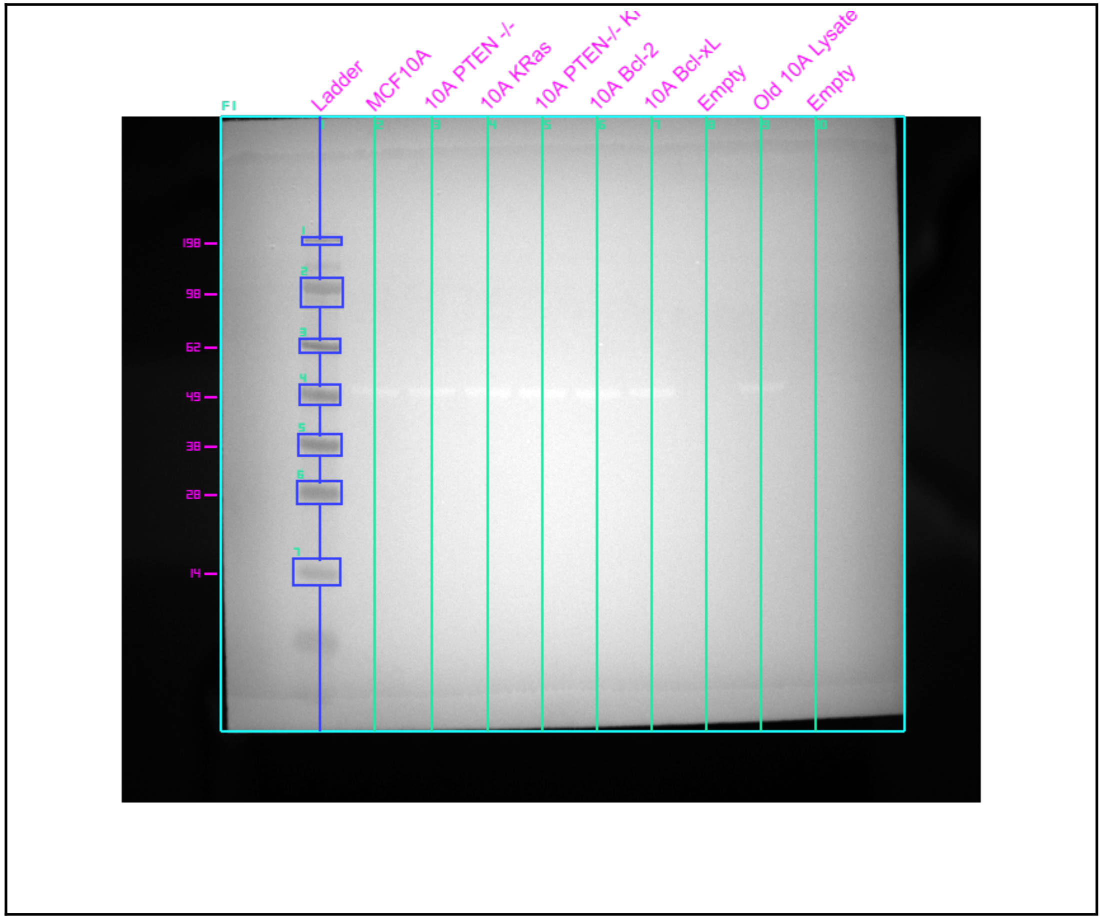

CHEMI_08272021_144328_2s_10A_VARIANTS_ALPHA_POLY

Date:	08-27-2021 02:43:28PM
Mode:	Chemi Blots
Notes:	Figure5A- alpha
Model:	FL1500
Instrument name:	2462619090234
Serial No:	2462619090234
Firmware version:	1.6.0
iBA version:	4.0.1
Image size:	676px X 540px
Image area:	
Optical Zoom:	2x
Digital Zoom:	1x
Focus level:	455
Resolution:	5 x 5
Exposure time:	2000 ms
Exposure mode:	Normal

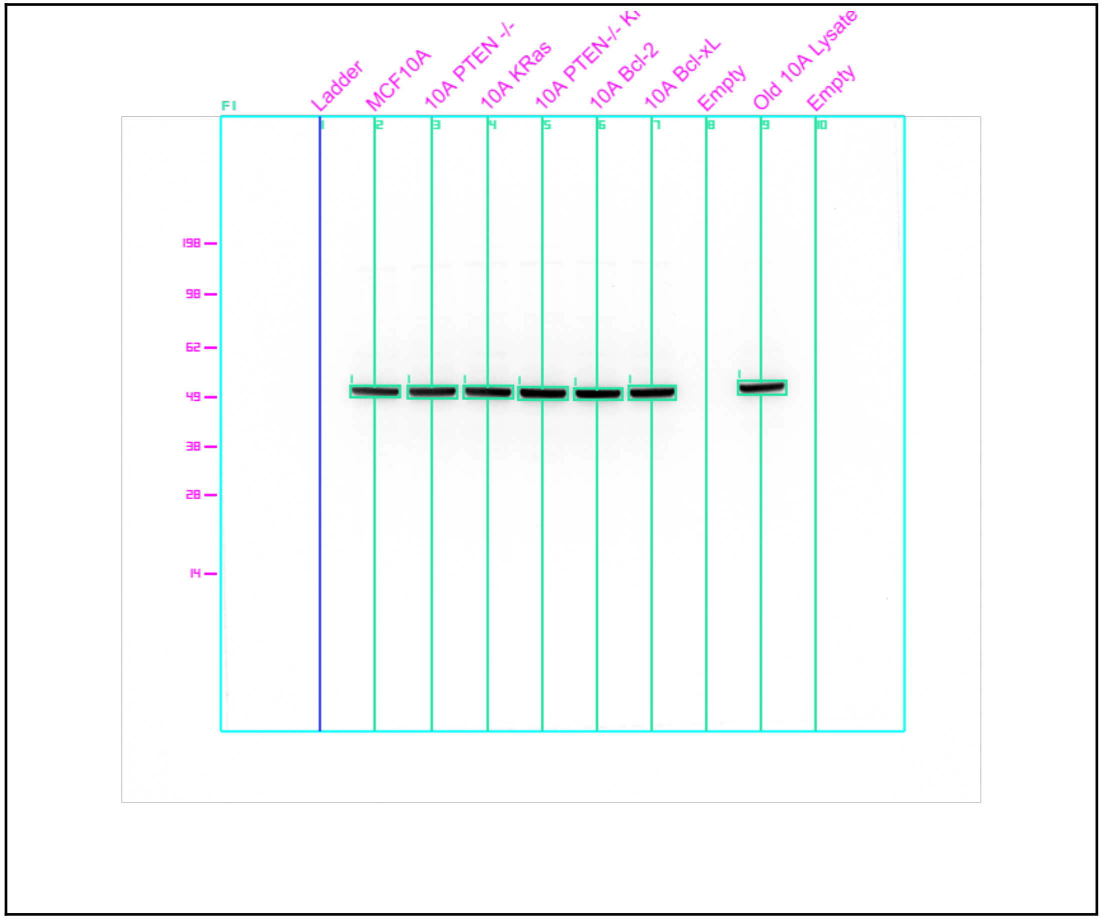

LANE AND BAND ANALYSIS DATA TABLE

CHEMI_08272021_144328_2s_10A_VARIANTS_ALPHA_POLY

Frame: 1
Channel: Membrane
Sensitivity: 100
Molecular Weight Analysis Regression Method : Point to Point

Lane 1 - Ladder

#	Vol. (Int.)	Local Bg. Corr. Vol.	Area	Rf	Density	Local Bg. Corr. Den.	% band purity	% lane purity	Mol. Wt.
1	6,947,515	661,676	224	0.202	31,015	2,953.912	3.872	1.312	198
2	25,662,732	2,744,386	816	0.285	31,449	3,363.219	16.058	4.844	98
3	13,082,627	2,126,851	396	0.372	33,036	5,370.837	12.444	2.47	62
4	18,007,206	2,857,211	561	0.452	32,098	5,093.069	16.718	3.399	49
5	20,313,879	3,396,246	630	0.533	32,244	5,390.867	19.872	3.835	38
6	21,908,878	3,408,821	684	0.612	32,030	4,983.658	19.945	4.136	28
7	24,507,386	1,895,659	836	0.74	29,315	2,267.536	11.092	4.626	14

Frame: 1
Channel: Chemi
Sensitivity: 100
Molecular Weight Analysis Regression Method : Point to Point

Lane 2 - MCF10A

#	Vol. (Int.)	Local Bg. Corr. Vol.	Area	Rf	Density	Local Bg. Corr. Den.	% band purity	% lane purity	Mol. Wt.
1	10,227,512	8,648,101	400	0.446	25,568	21,620	100	56.944	50

Lane 3 - 10A PTEN -/-

#	Vol. (Int.)	Local Bg. Corr. Vol.	Area	Rf	Density	Local Bg. Corr. Den.	% band purity	% lane purity	Mol. Wt.
1	11,743,378	9,719,384	440	0.448	26,689	22,089	100	56.183	49.667

Lane 4 - 10A KRas

#	Vol. (Int.)	Local Bg. Corr. Vol.	Area	Rf	Density	Local Bg. Corr. Den.	% band purity	% lane purity	Mol. Wt.
1	12,446,895	10,133,421	440	0.448	28,288	23,030	100	55.394	49.667

Lane 5 - 10A PTEN-/- KRas

#	Vol. (Int.)	Local Bg. Corr. Vol.	Area	Rf	Density	Local Bg. Corr. Den.	% band purity	% lane purity	Mol. Wt.
1	13,801,853	11,388,999	440	0.45	31,367	25,884	100	57.77	49.333

Lane 6 - 10A Bcl-2

#	Vol. (Int.)	Local Bg. Corr. Vol.	Area	Rf	Density	Local Bg. Corr. Den.	% band purity	% lane purity	Mol. Wt.
1	12,783,738	10,561,607	390	0.45	32,778	27,081	100	55.242	49.333

Lane 7 - 10A Bcl-xL

#	Vol. (Int.)	Local Bg. Corr. Vol.	Area	Rf	Density	Local Bg. Corr. Den.	% band purity	% lane purity	Mol. Wt.
1	13,107,585	11,254,128	456	0.448	28,744	24,680	100	60.276	49.667

Lane 9 - Old 10A Lysate

#	Vol. (Int.)	Local Bg. Corr. Vol.	Area	Rf	Density	Local Bg. Corr. Den.	% band purity	% lane purity	Mol. Wt.
1	11,340,121	9,893,951	468	0.44	24,231	21,140	100	62.478	51

iBright™ Image Analysis Report

28 January 2022

Figure 5A- VASH1

CHEMI_08232021_233032_2s_412ms_10A_VARIANTS_VASH1_MS

Date:	08-23-2021 11:30:32PM
Mode:	Chemi Blots
Notes:	Figure5A- VASH1
Model:	FL1500
Instrument name:	2462619090234
Serial No:	2462619090234
Firmware version:	1.6.0
iBA version:	4.0.1
Image size:	676px X 540px
Image area:	
Optical Zoom:	2x
Digital Zoom:	1x
Focus level:	455
Resolution:	5 x 5
Exposure time:	2412 ms
Exposure mode:	Normal

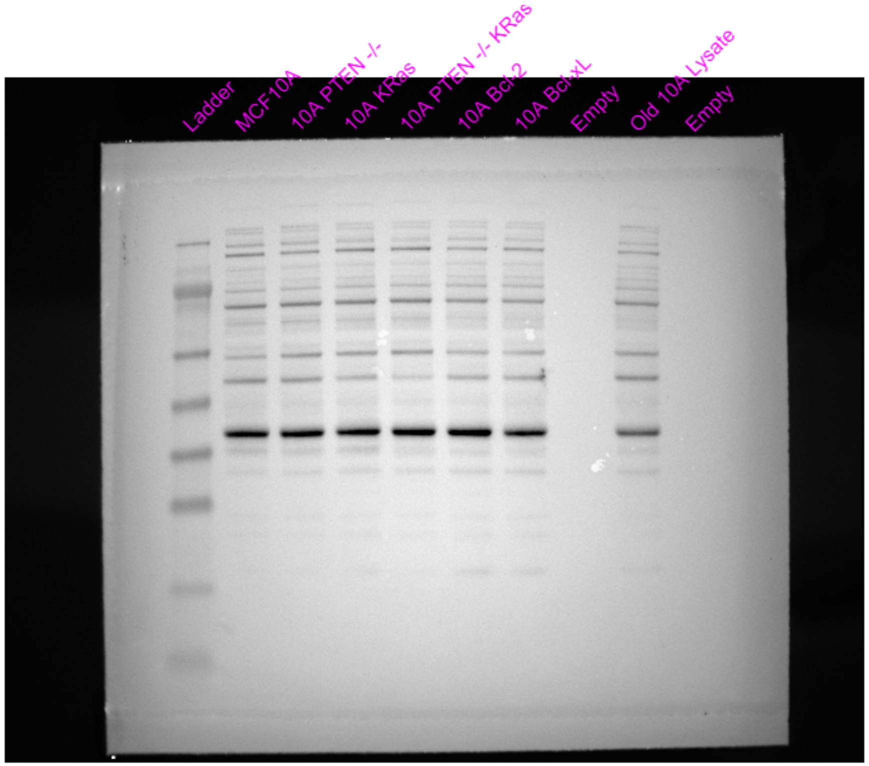

CHEMI_08232021_233032_2s_412ms_10A_VARIANTS_VASH1_MS

Date:	08-23-2021 11:30:32PM
Mode:	Chemi Blots
Notes:	Figure5A- VASH1
Model:	FL1500
Instrument name:	2462619090234
Serial No:	2462619090234
Firmware version:	1.6.0
iBA version:	4.0.1
Image size:	676px X 540px
Image area:	
Optical Zoom:	2x
Digital Zoom:	1x
Focus level:	455
Resolution:	5 x 5
Exposure time:	2412 ms
Exposure mode:	Normal

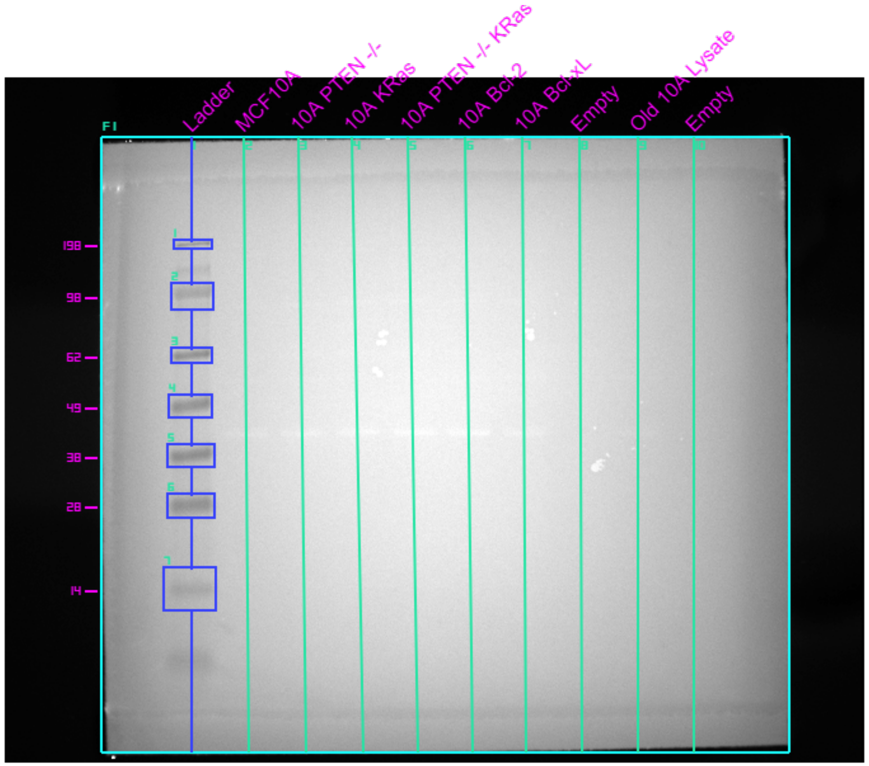

CHEMI_08232021_233032_2s_412ms_10A_ VARIANTS_VASH1_MS

Date:	08-23-2021 11:30:32PM
Mode:	Chemi Blots
Notes:	Figure5A- VASH1
Model:	FL1500
Instrument name:	2462619090234
Serial No:	2462619090234
Firmware version:	1.6.0
iBA version:	4.0.1
Image size:	676px X 540px
Image area:	
Optical Zoom:	2x
Digital Zoom:	1x
Focus level:	455
Resolution:	5 x 5
Exposure time:	2412 ms
Exposure mode:	Normal

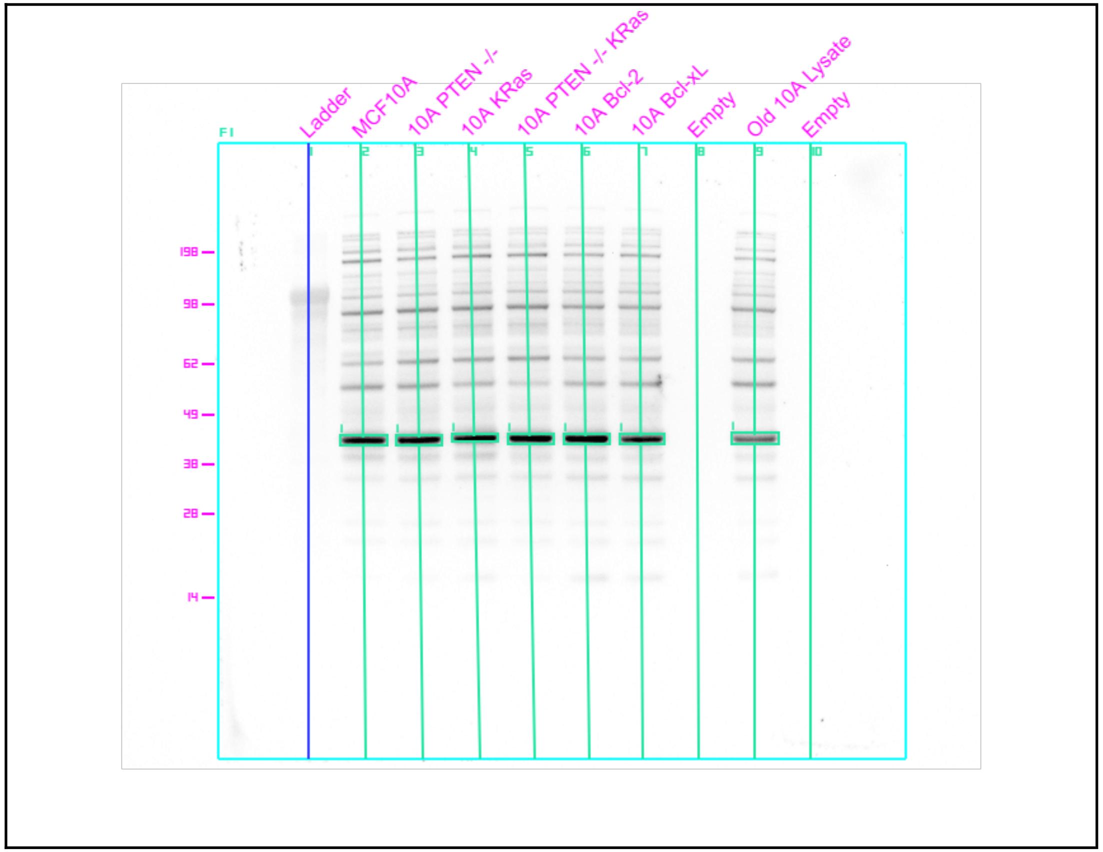

LANE AND BAND ANALYSIS DATA TABLE

CHEMI_08232021_233032_2s_412ms_10A_VARIANTS_VASH1_MS

Frame: 1
Channel: Membrane
Sensitivity: 100
Molecular Weight Analysis Regression Method : Point to Point

Lane 1 - Ladder

#	Vol. (Int.)	Local Bg. Corr. Vol.	Area	Rf	Density	Local Bg. Corr. Den.	% band purity	% lane purity	Mol. Wt.
1	7,576,453	710,702	248	0.173	30,550	2,865.734	4.257	1.516	198
2	22,489,843	2,027,821	748	0.258	30,066	2,710.991	12.146	4.5	98
3	13,620,635	1,998,382	429	0.355	31,749	4,658.234	11.969	2.725	62
4	21,224,309	3,055,471	665	0.437	31,916	4,594.694	18.301	4.246	49
5	23,541,308	3,441,866	722	0.518	32,605	4,767.128	20.615	4.71	38
6	25,263,925	3,559,428	760	0.598	33,242	4,683.458	21.319	5.055	28
7	45,949,651	1,902,240	1,470	0.734	31,258	1,294.041	11.393	9.193	14

Frame: 1
Channel: Chemi
Sensitivity: 100
Molecular Weight Analysis Regression Method : Point to Point

Lane 2 - MCF10A

#	Vol. (Int.)	Local Bg. Corr. Vol.	Area	Rf	Density	Local Bg. Corr. Den.	% band purity	% lane purity	Mol. Wt.
1	7,165,487	5,834,030	342	0.482	20,951	17,058	100	23.819	42.795

Lane 3 - 10A PTEN -/-

#	Vol. (Int.)	Local Bg. Corr. Vol.	Area	Rf	Density	Local Bg. Corr. Den.	% band purity	% lane purity	Mol. Wt.
1	7,482,191	6,089,856	333	0.482	22,469	18,287	100	22.463	42.795

Lane 4 - 10A KRas

#	Vol. (Int.)	Local Bg. Corr. Vol.	Area	Rf	Density	Local Bg. Corr. Den.	% band purity	% lane purity	Mol. Wt.
1	6,780,076	5,391,795	259	0.478	26,177	20,817	100	19.371	43.359

Lane 5 - 10A PTEN -/- KRas

#	Vol. (Int.)	Local Bg. Corr. Vol.	Area	Rf	Density	Local Bg. Corr. Den.	% band purity	% lane purity	Mol. Wt.
1	8,245,735	6,756,541	333	0.48	24,761	20,289	100	24.022	43.077

Lane 6 - 10A Bcl-2

#	Vol. (Int.)	Local Bg. Corr. Vol.	Area	Rf	Density	Local Bg. Corr. Den.	% band purity	% lane purity	Mol. Wt.
1	8,892,608	7,421,981	333	0.48	26,704	22,288	100	25.786	43.077

Lane 7 - 10A Bcl-xL

#	Vol. (Int.)	Local Bg. Corr. Vol.	Area	Rf	Density	Local Bg. Corr. Den.	% band purity	% lane purity	Mol. Wt.
1	6,311,534	5,097,999	324	0.48	19,480	15,734	100	20.444	43.077

Lane 9 - Old 10A Lysate

#	Vol. (Int.)	Local Bg. Corr. Vol.	Area	Rf	Density	Local Bg. Corr. Den.	% band purity	% lane purity	Mol. Wt.
1	4,407,292	3,690,394	380	0.478	11,598	9,711.565	100	19.034	43.359

iBright™ Image Analysis Report

28 January 2022

Figure 5A- VASH2

CHEMI_08232021_233622_10s_10A_VARIANTS_VASH2_EMD_MS

Date:	08-23-2021 11:36:22PM
Mode:	Chemi Blots
Notes:	Figure5A- VASH2
Model:	FL1500
Instrument name:	2462619090234
Serial No:	2462619090234
Firmware version:	1.6.0
iBA version:	4.0.1
Image size:	676px X 540px
Image area:	
Optical Zoom:	2x
Digital Zoom:	1x
Focus level:	455
Resolution:	5 x 5
Exposure time:	10000 ms
Exposure mode:	Normal

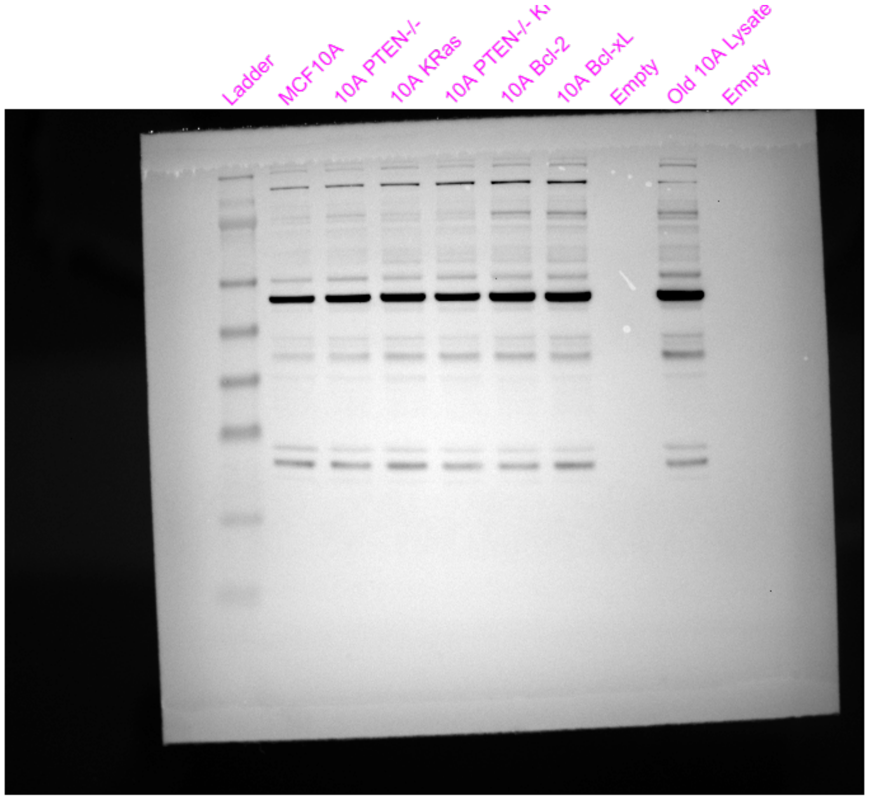

CHEMI_08232021_233622_10s_10A_VARIANTS_VASH2_EMD_MS

Date:	08-23-2021 11:36:22PM
Mode:	Chemi Blots
Notes:	Figure5A- VASH2
Model:	FL1500
Instrument name:	2462619090234
Serial No:	2462619090234
Firmware version:	1.6.0
iBA version:	4.0.1
Image size:	676px X 540px
Image area:	
Optical Zoom:	2x
Digital Zoom:	1x
Focus level:	455
Resolution:	5 x 5
Exposure time:	10000 ms
Exposure mode:	Normal

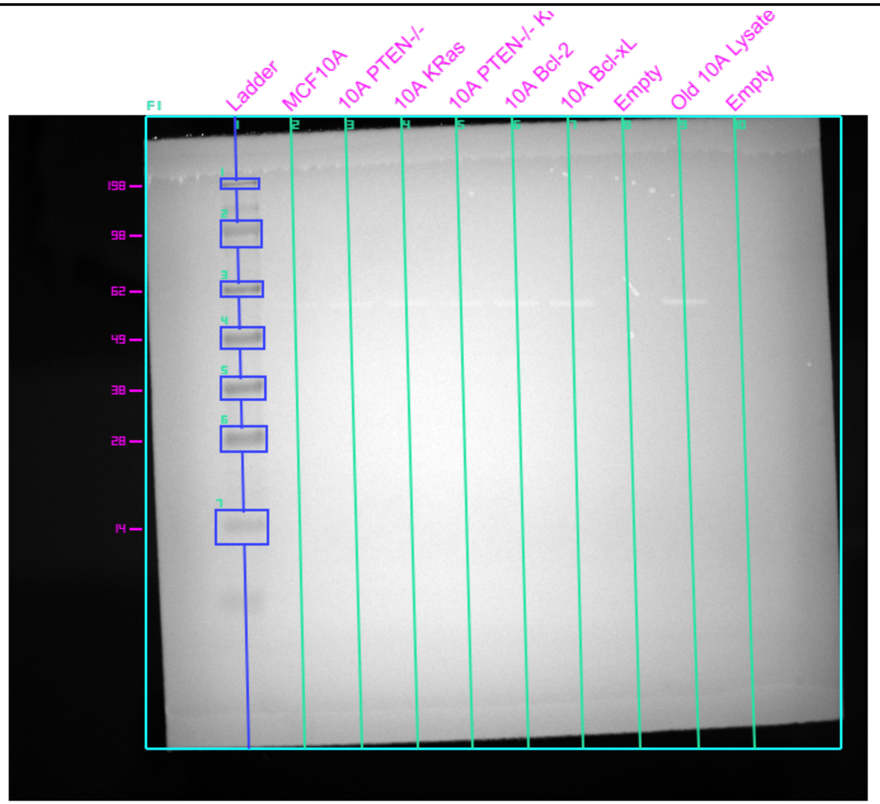

CHEMI_08232021_233622_10s_10A_VARIANTS_VASH2_EMD_MS

Date:	08-23-2021 11:36:22PM
Mode:	Chemi Blots
Notes:	Figure5A- VASH2
Model:	FL1500
Instrument name:	2462619090234
Serial No:	2462619090234
Firmware version:	1.6.0
iBA version:	4.0.1
Image size:	676px X 540px
Image area:	
Optical Zoom:	2x
Digital Zoom:	1x
Focus level:	455
Resolution:	5 x 5
Exposure time:	10000 ms
Exposure mode:	Normal

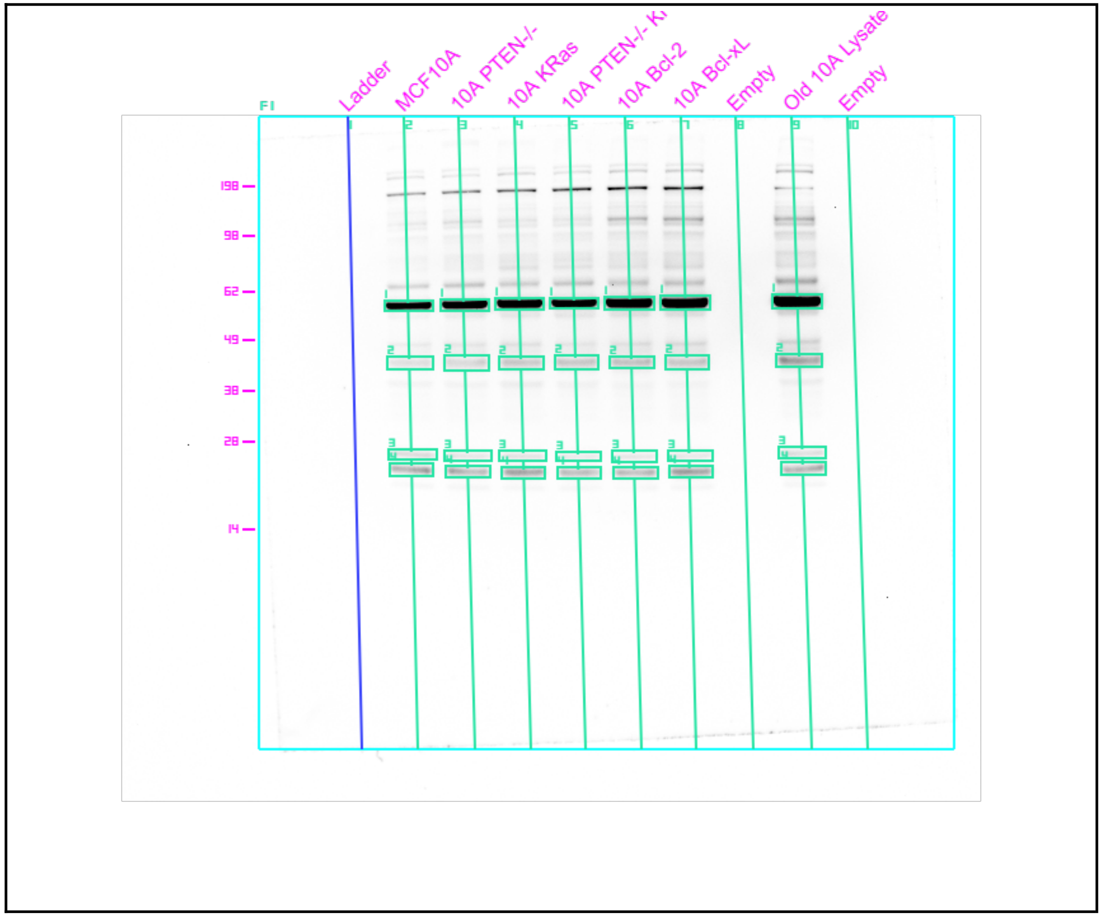

LANE AND BAND ANALYSIS DATA TABLE

CHEMI_08232021_233622_10s_10A_VARIANTS_VASH2_EMD_MS

Frame: 1
Channel: Membrane
Sensitivity: 100
Molecular Weight Analysis Regression Method : Point to Point

Lane 1 - Ladder

#	Vol. (Int.)	Local Bg. Corr. Vol.	Area	Rf	Density	Local Bg. Corr. Den.	% band purity	% lane purity	Mol. Wt.
1	9,318,612	734,870	279	0.106	33,400	2,633.943	4.492	1.85	198
2	22,892,709	2,061,738	726	0.185	31,532	2,839.86	12.602	4.544	98
3	14,141,286	2,039,303	442	0.273	31,993	4,613.809	12.465	2.807	62
4	19,497,619	2,939,895	630	0.349	30,948	4,666.501	17.97	3.87	49
5	20,792,534	3,258,021	684	0.43	30,398	4,763.189	19.914	4.128	38
6	23,445,602	3,612,742	777	0.51	30,174	4,649.605	22.082	4.654	28
7	33,162,028	1,713,842	1,176	0.649	28,199	1,457.349	10.476	6.583	14

Frame: 1
Channel: Chemi
Sensitivity: 100
Molecular Weight Analysis Regression Method : Point to Point

Lane 2 - MCF10A

#	Vol. (Int.)	Local Bg. Corr. Vol.	Area	Rf	Density	Local Bg. Corr. Den.	% band purity	% lane purity	Mol. Wt.
1	13,790,661	12,918,420	351	0.299	39,289	36,804	70.239	39.147	57.553
2	1,946,725	1,277,775	407	0.39	4,783.108	3,139.497	6.947	5.526	43.5
3	1,499,469	913,231	351	0.534	4,271.991	2,601.799	4.965	4.256	25.565
4	3,726,829	3,282,593	385	0.558	9,680.075	8,526.217	17.848	10.579	23.13

Lane 3 - 10A PTEN-/-

#	Vol. (Int.)	Local Bg. Corr. Vol.	Area	Rf	Density	Local Bg. Corr. Den.	% band purity	% lane purity	Mol. Wt.
1	15,720,071	14,211,608	390	0.297	40,307	36,440	73.063	35.967	57.895
2	2,797,395	1,678,757	468	0.39	5,977.34	3,587.088	8.631	6.4	43.5
3	1,139,400	599,004	342	0.536	3,331.579	1,751.474	3.08	2.607	25.362

#	Vol. (Int.)	Local Bg. Corr. Vol.	Area	Rf	Density	Local Bg. Corr. Den.	% band purity	% lane purity	Mol. Wt.
4	3,390,913	2,961,689	360	0.56	9,419.203	8,226.914	15.226	7.758	22.928

Lane 4 - 10A KRas

#	Vol. (Int.)	Local Bg. Corr. Vol.	Area	Rf	Density	Local Bg. Corr. Den.	% band purity	% lane purity	Mol. Wt.
1	16,835,447	14,972,945	390	0.295	43,167	38,392	69.803	33.367	58.237
2	3,257,792	2,180,248	396	0.39	8,226.747	5,505.678	10.164	6.457	43.5
3	1,273,127	688,179	342	0.536	3,722.594	2,012.222	3.208	2.523	25.362
4	4,154,440	3,608,797	350	0.562	11,869	10,310	16.824	8.234	22.725

Lane 5 - 10A PTEN-/- KRas

#	Vol. (Int.)	Local Bg. Corr. Vol.	Area	Rf	Density	Local Bg. Corr. Den.	% band purity	% lane purity	Mol. Wt.
1	15,329,420	13,417,539	351	0.295	43,673	38,226	71.642	30.917	58.237
2	3,516,710	2,323,294	420	0.388	8,373.119	5,531.653	12.405	7.093	43.775
3	883,592	446,228	288	0.536	3,068.028	1,549.404	2.383	1.782	25.362
4	3,015,487	2,541,522	350	0.562	8,615.677	7,261.493	13.57	6.082	22.725

Lane 6 - 10A Bcl-2

#	Vol. (Int.)	Local Bg. Corr. Vol.	Area	Rf	Density	Local Bg. Corr. Den.	% band purity	% lane purity	Mol. Wt.
1	19,260,632	16,363,802	440	0.295	43,774	37,190	75.065	32.263	58.237
2	3,614,014	2,352,915	360	0.388	10,038	6,535.877	10.793	6.054	43.775
3	1,134,425	459,855	360	0.536	3,151.181	1,277.378	2.109	1.9	25.362
4	3,136,860	2,622,920	360	0.562	8,713.5	7,285.89	12.032	5.254	22.725

Lane 7 - 10A Bcl-xL

#	Vol. (Int.)	Local Bg. Corr. Vol.	Area	Rf	Density	Local Bg. Corr. Den.	% band purity	% lane purity	Mol. Wt.
1	20,607,310	17,741,374	480	0.293	42,931	36,961	73.183	32	58.579
2	3,698,107	2,248,491	420	0.388	8,805.017	5,353.551	9.275	5.743	43.775
3	1,075,675	412,649	324	0.536	3,319.985	1,273.611	1.702	1.67	25.362
4	4,405,914	3,840,102	350	0.56	12,588	10,971	15.84	6.842	22.928

Lane 9 - Old 10A Lysate

#	Vol. (Int.)	Local Bg. Corr. Vol.	Area	Rf	Density	Local Bg. Corr. Den.	% band purity	% lane purity	Mol. Wt.
1	22,691,658	19,224,278	480	0.291	47,274	40,050	69.659	34.186	58.921
2	5,921,539	4,304,022	407	0.386	14,549	10,574	15.596	8.921	44.05
3	1,620,976	941,565	380	0.53	4,265.726	2,477.804	3.412	2.442	25.971
4	3,703,951	3,127,801	396	0.556	9,353.412	7,898.488	11.334	5.58	23.333

iBright™ Image Analysis Report

28 January 2022

CHEMI_09302021_143508_30s_10A_VARIANTS_INV_SVBP

Date:	09-30-2021 02:35:08PM
Mode:	Chemi Blots
Notes:	Figure5A- SVBP
Model:	FL1500
Instrument name:	2462619090234
Serial No:	2462619090234
Firmware version:	1.6.0
iBA version:	4.0.1
Image size:	676px X 540px
Image area:	
Optical Zoom:	2x
Digital Zoom:	1x
Focus level:	455
Resolution:	5 x 5
Exposure time:	30000 ms
Exposure mode:	Normal

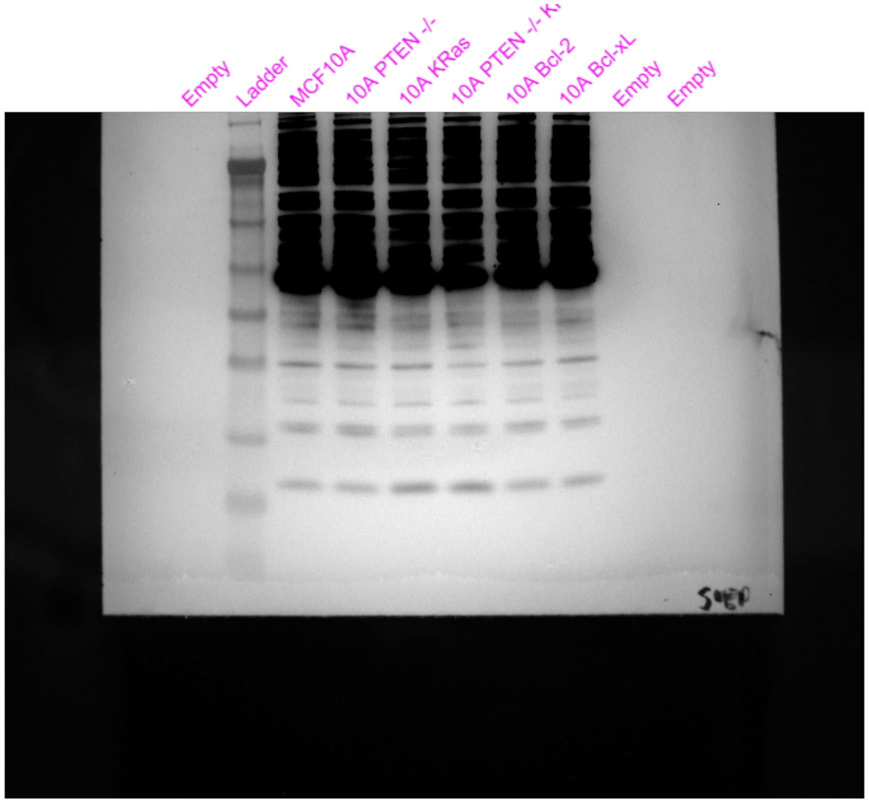

CHEMI_09302021_143508_30s_10A_VARIANTS_INV_SVBP

Date:	09-30-2021 02:35:08PM
Mode:	Chemi Blots
Notes:	Figure5A- SVBP
Model:	FL1500
Instrument name:	2462619090234
Serial No:	2462619090234
Firmware version:	1.6.0
iBA version:	4.0.1
Image size:	676px X 540px
Image area:	
Optical Zoom:	2x
Digital Zoom:	1x
Focus level:	455
Resolution:	5 x 5
Exposure time:	30000 ms
Exposure mode:	Normal

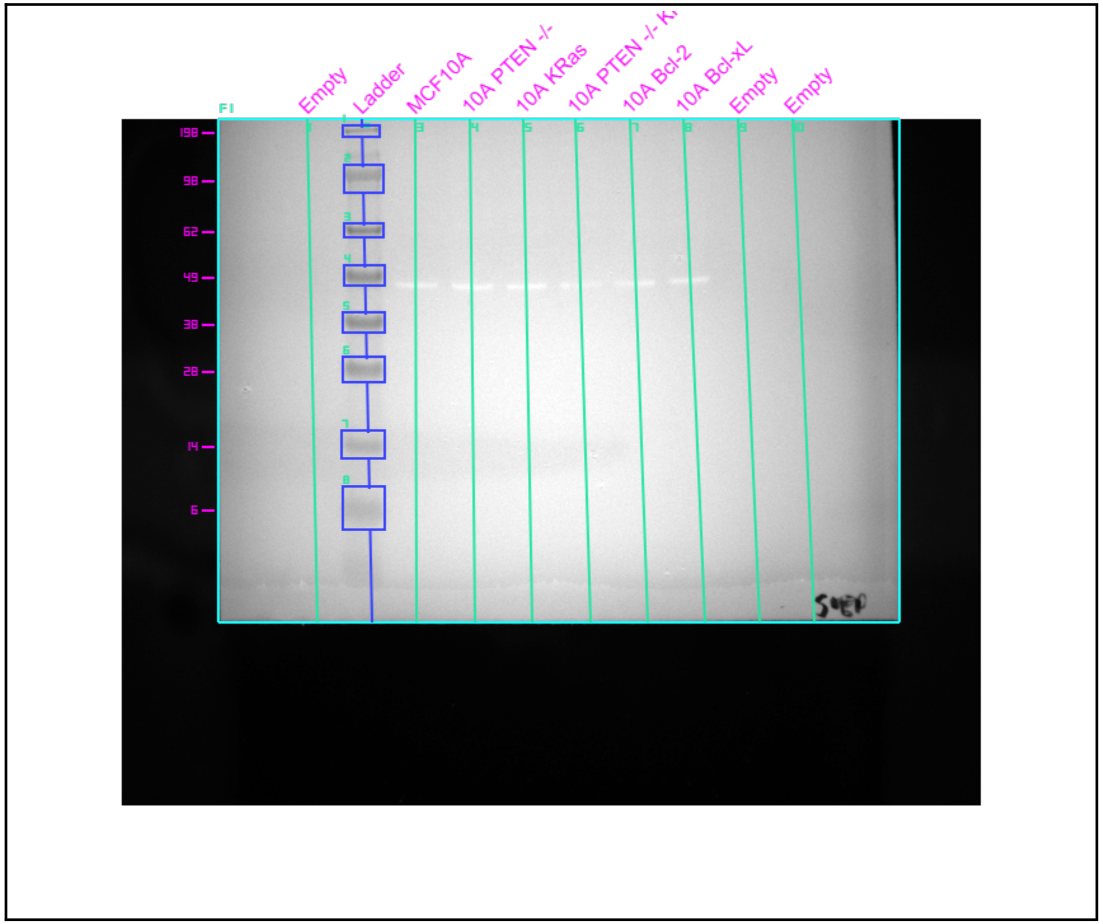

CHEMI_09302021_143508_30s_10A_VARIANTS_INV_SVBP

Date:	09-30-2021 02:35:08PM
Mode:	Chemi Blots
Notes:	Figure5A- SVBP
Model:	FL1500
Instrument name:	2462619090234
Serial No:	2462619090234
Firmware version:	1.6.0
iBA version:	4.0.1
Image size:	676px X 540px
Image area:	
Optical Zoom:	2x
Digital Zoom:	1x
Focus level:	455
Resolution:	5 x 5
Exposure time:	30000 ms
Exposure mode:	Normal

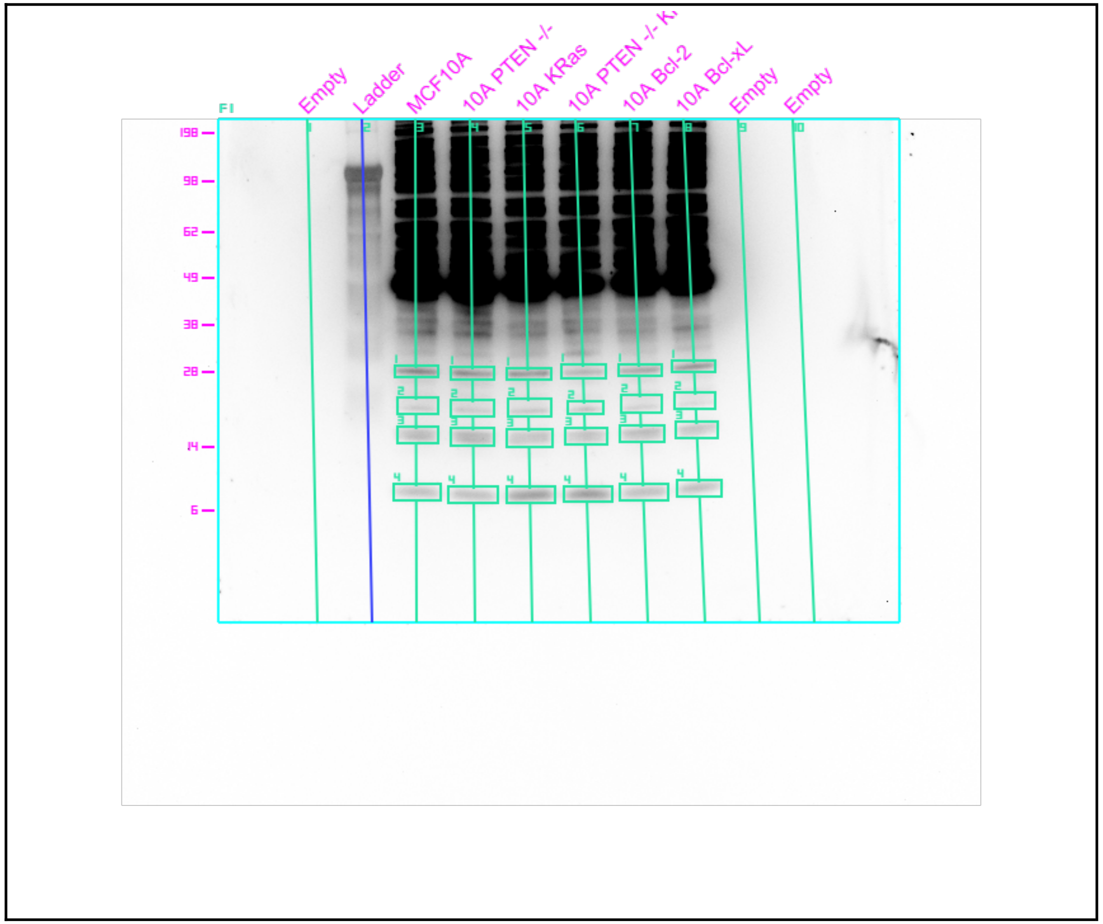

LANE AND BAND ANALYSIS DATA TABLE

CHEMI_09302021_143508_30s_10A_VARIANTS_INV_SVBP

Frame: 1
Channel: Membrane
Sensitivity: 100
Molecular Weight Analysis Regression Method : Point to Point

Lane 2 - Ladder

#	Vol. (Int.)	Local Bg. Corr. Vol.	Area	Rf	Density	Local Bg. Corr. Den.	% band purity	% lane purity	Mol. Wt.
1	9,317,084	769,164	300	0.023	31,056	2,563.88	3.531	2.618	198
2	22,614,437	2,613,967	736	0.119	30,726	3,551.587	12.001	6.355	98
3	12,514,583	2,256,427	384	0.22	32,590	5,876.113	10.359	3.517	62
4	17,959,227	3,237,175	561	0.311	32,012	5,770.366	14.862	5.047	49
5	18,083,372	3,723,117	578	0.404	31,286	6,441.38	17.093	5.082	38
6	20,846,769	3,785,733	714	0.497	29,197	5,302.148	17.38	5.858	28
7	22,454,767	2,369,599	805	0.646	27,894	2,943.602	10.879	6.31	14
8	32,585,632	3,026,704	1,190	0.773	27,382	2,543.45	13.896	9.157	6

Frame: 1
Channel: Chemi
Sensitivity: 100
Molecular Weight Analysis Regression Method : Point to Point

Lane 3 - MCF10A

#	Vol. (Int.)	Local Bg. Corr. Vol.	Area	Rf	Density	Local Bg. Corr. Den.	% band purity	% lane purity	Mol. Wt.
1	6,482,368	2,666,003	350	0.5	18,521	7,617.152	28.596	1.767	27.763
2	4,111,155	1,048,547	462	0.568	8,898.604	2,269.584	11.247	1.12	21.356
3	4,978,248	2,712,464	462	0.626	10,775	5,871.135	29.095	1.357	15.898
4	4,451,206	2,895,820	532	0.74	8,366.929	5,443.272	31.062	1.213	8.08

Lane 4 - 10A PTEN -/-

#	Vol. (Int.)	Local Bg. Corr. Vol.	Area	Rf	Density	Local Bg. Corr. Den.	% band purity	% lane purity	Mol. Wt.
1	6,736,671	2,328,093	385	0.505	17,497	6,046.995	23.163	1.785	27.288
2	4,533,716	1,167,536	490	0.573	9,252.482	2,382.728	11.616	1.201	20.881

#	Vol. (Int.)	Local Bg. Corr. Vol.	Area	Rf	Density	Local Bg. Corr. Den.	% band purity	% lane purity	Mol. Wt.
3	6,273,757	3,502,130	525	0.631	11,950	6,670.725	34.844	1.663	15.424
4	4,864,295	3,053,035	560	0.745	8,686.241	5,451.849	30.376	1.289	7.76

Lane 5 - 10A KRas

#	Vol. (Int.)	Local Bg. Corr. Vol.	Area	Rf	Density	Local Bg. Corr. Den.	% band purity	% lane purity	Mol. Wt.
1	5,915,118	2,421,572	360	0.505	16,430	6,726.591	20.46	1.622	27.288
2	4,508,691	1,458,672	525	0.573	8,587.983	2,778.423	12.325	1.236	20.881
3	5,360,669	2,836,026	555	0.634	9,658.863	5,109.957	23.962	1.47	15.186
4	7,038,900	5,119,088	546	0.745	12,891	9,375.619	43.252	1.93	7.76

Lane 6 - 10A PTEN -/- KRas

#	Vol. (Int.)	Local Bg. Corr. Vol.	Area	Rf	Density	Local Bg. Corr. Den.	% band purity	% lane purity	Mol. Wt.
1	5,145,456	1,437,436	432	0.5	11,910	3,327.4	14.824	1.477	27.763
2	2,603,667	947,188	319	0.573	8,161.966	2,969.242	9.768	0.747	20.881
3	4,416,454	2,525,931	476	0.629	9,278.265	5,306.58	26.05	1.267	15.661
4	6,638,164	4,786,104	507	0.745	13,093	9,440.048	49.358	1.905	7.76

Lane 7 - 10A Bcl-2

#	Vol. (Int.)	Local Bg. Corr. Vol.	Area	Rf	Density	Local Bg. Corr. Den.	% band purity	% lane purity	Mol. Wt.
1	4,821,372	1,889,713	350	0.497	13,775	5,399.18	20.277	1.374	28
2	3,711,165	1,254,334	495	0.566	7,497.303	2,534.009	13.459	1.058	21.593
3	5,035,964	3,093,127	504	0.624	9,991.992	6,137.157	33.19	1.435	16.136
4	4,784,161	3,082,274	546	0.74	8,762.2	5,645.191	33.074	1.363	8.08

Lane 8 - 10A Bcl-xL

#	Vol. (Int.)	Local Bg. Corr. Vol.	Area	Rf	Density	Local Bg. Corr. Den.	% band purity	% lane purity	Mol. Wt.
1	5,232,848	2,475,331	350	0.49	14,950	7,072.376	28.562	1.535	28.811
2	2,931,271	904,424	462	0.558	6,344.742	1,957.628	10.436	0.86	22.305
3	3,917,025	2,407,755	476	0.616	8,229.044	5,058.309	27.782	1.149	16.847
4	4,006,016	2,879,085	504	0.732	7,948.444	5,712.472	33.22	1.175	8.56

iBright™ Image Analysis Report

28 January 2022

Figure 5A- GAPDH

CHEMI_10112021_173757_500ms_10A_VARIANTS_SNTCRZ_GAPDH

Date:	10-11-2021 05:37:57PM
Mode:	Chemi Blots
Notes:	Figure5A- GAPDH
Model:	FL1500
Instrument name:	2462619090234
Serial No:	2462619090234
Firmware version:	1.6.0
iBA version:	4.0.1
Image size:	676px X 540px
Image area:	
Optical Zoom:	2x
Digital Zoom:	1x
Focus level:	455
Resolution:	5 x 5
Exposure time:	500 ms
Exposure mode:	Normal

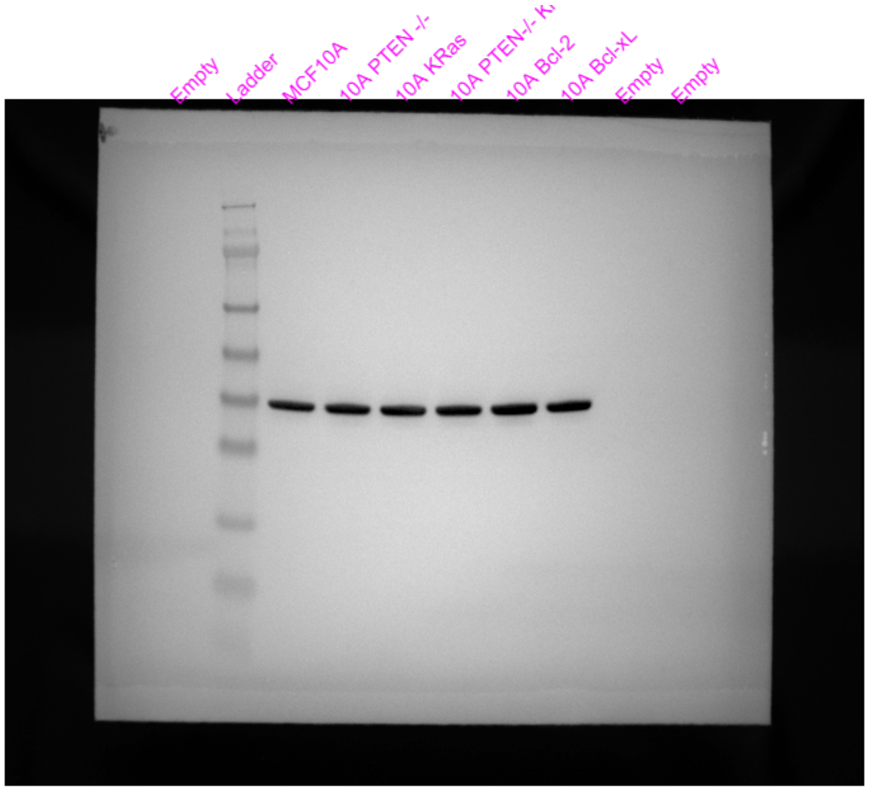

CHEMI_10112021_173757_500ms_10A_VARIANTS_SNTCRZ_GAPDH

Date:	10-11-2021 05:37:57PM
Mode:	Chemi Blots
Notes:	Figure5A- GAPDH
Model:	FL1500
Instrument name:	2462619090234
Serial No:	2462619090234
Firmware version:	1.6.0
iBA version:	4.0.1
Image size:	676px X 540px
Image area:	
Optical Zoom:	2x
Digital Zoom:	1x
Focus level:	455
Resolution:	5 x 5
Exposure time:	500 ms
Exposure mode:	Normal

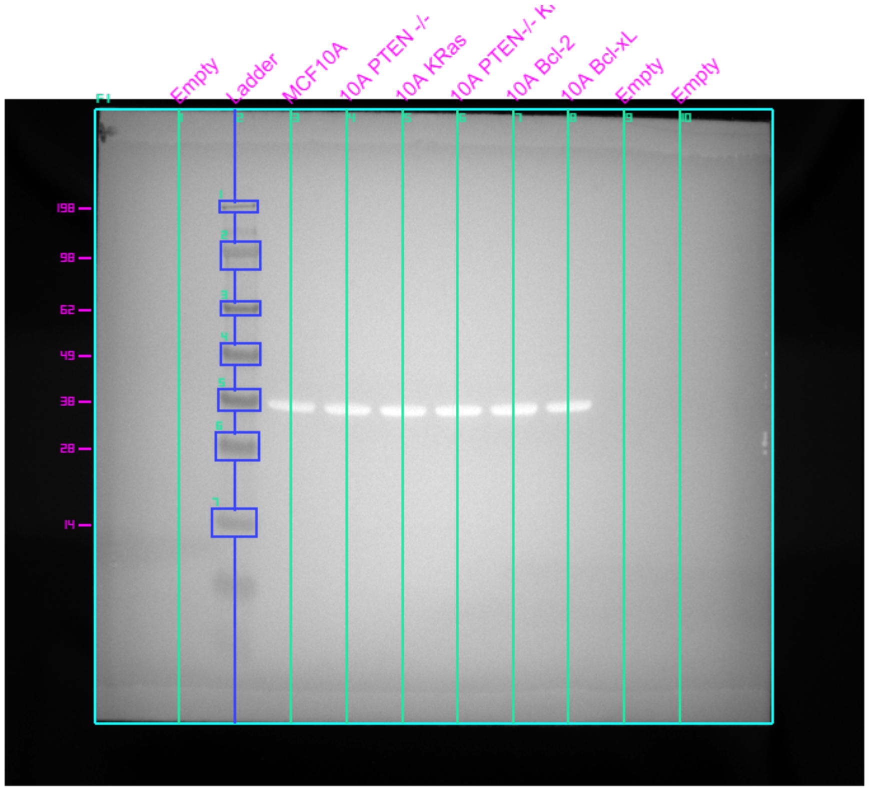

CHEMI_10112021_173757_500ms_10A_VARIANTS_SNTCRZ_GAPDH

Date:	10-11-2021 05:37:57PM
Mode:	Chemi Blots
Notes:	Figure5A- GAPDH
Model:	FL1500
Instrument name:	2462619090234
Serial No:	2462619090234
Firmware version:	1.6.0
iBA version:	4.0.1
Image size:	676px X 540px
Image area:	
Optical Zoom:	2x
Digital Zoom:	1x
Focus level:	455
Resolution:	5 x 5
Exposure time:	500 ms
Exposure mode:	Normal

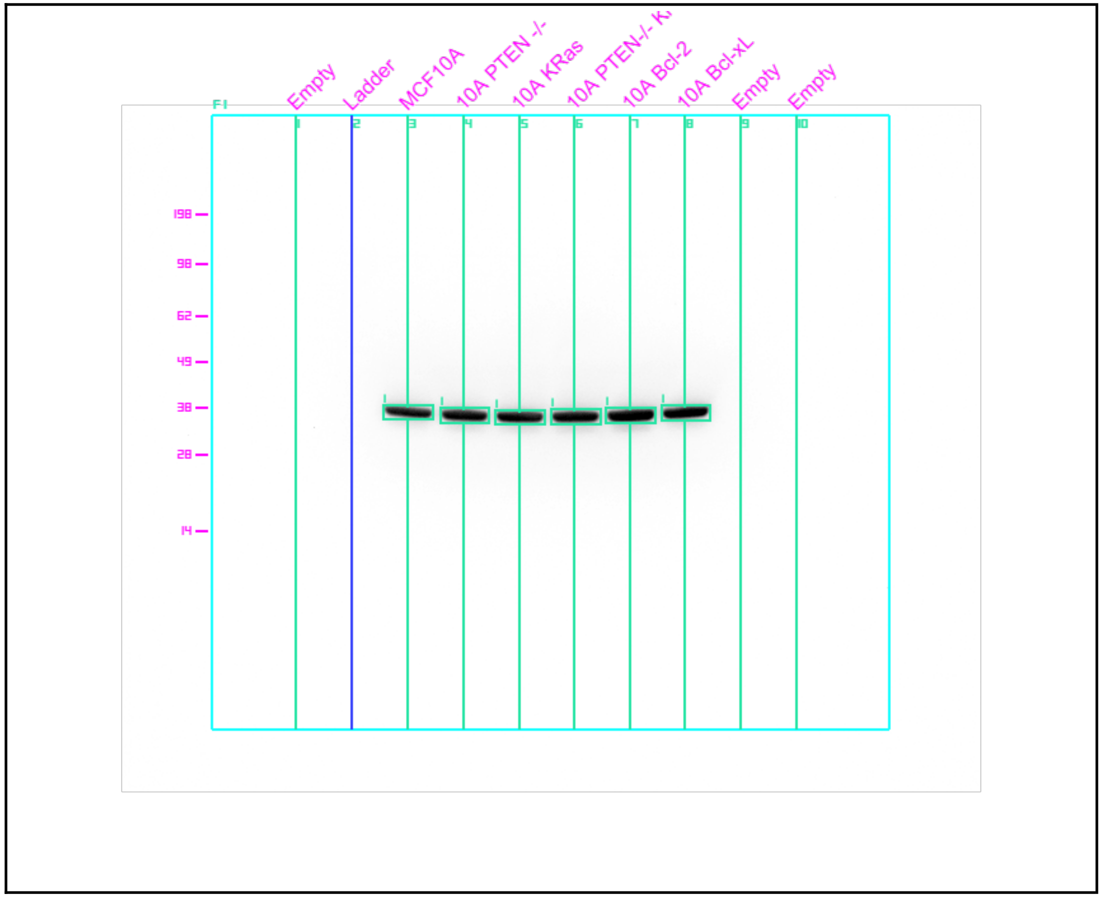

LANE AND BAND ANALYSIS DATA TABLE

CHEMI_10112021_173757_500ms_10A_VARIANTS_SNTCRZ_GAPDH

Frame: 1
Channel: Membrane
Sensitivity: 100
Molecular Weight Analysis Regression Method : Point to Point

Lane 2 - Ladder

#	Vol. (Int.)	Local Bg. Corr. Vol.	Area	Rf	Density	Local Bg. Corr. Den.	% band purity	% lane purity	Mol. Wt.
1	9,261,845	746,570	310	0.157	29,876	2,408.292	4.287	1.808	198
2	21,974,129	2,335,912	736	0.238	29,856	3,173.795	13.412	4.289	98
3	12,311,774	2,177,441	384	0.323	32,061	5,670.421	12.502	2.403	62
4	18,058,267	2,958,264	576	0.398	31,351	5,135.876	16.985	3.525	49
5	19,366,203	3,499,104	612	0.472	31,644	5,717.491	20.091	3.78	38
6	23,946,456	3,600,417	805	0.549	29,747	4,472.568	20.672	4.674	28
7	23,611,444	2,098,871	828	0.673	28,516	2,534.869	12.051	4.609	14

Frame: 1
Channel: Chemi
Sensitivity: 100
Molecular Weight Analysis Regression Method : Point to Point

Lane 3 - MCF10A

#	Vol. (Int.)	Local Bg. Corr. Vol.	Area	Rf	Density	Local Bg. Corr. Den.	% band purity	% lane purity	Mol. Wt.
1	13,202,033	11,010,745	480	0.482	27,504	22,939	100	64.73	36.649

Lane 4 - 10A PTEN -/-

#	Vol. (Int.)	Local Bg. Corr. Vol.	Area	Rf	Density	Local Bg. Corr. Den.	% band purity	% lane purity	Mol. Wt.
1	14,957,109	12,241,484	507	0.489	29,501	24,144	100	63.902	35.838

Lane 5 - 10A KRas

#	Vol. (Int.)	Local Bg. Corr. Vol.	Area	Rf	Density	Local Bg. Corr. Den.	% band purity	% lane purity	Mol. Wt.
1	14,868,426	12,007,252	480	0.491	30,975	25,015	100	61.177	35.568

Lane 6 - 10A PTEN-/- Kras

#	Vol. (Int.)	Local Bg. Corr. Vol.	Area	Rf	Density	Local Bg. Corr. Den.	% band purity	% lane purity	Mol. Wt.
1	15,810,442	12,579,556	520	0.491	30,404	24,191	100	61.704	35.568

Lane 7 - 10A Bcl-2

#	Vol. (Int.)	Local Bg. Corr. Vol.	Area	Rf	Density	Local Bg. Corr. Den.	% band purity	% lane purity	Mol. Wt.
1	17,903,307	14,368,901	520	0.489	34,429	27,632	100	64.807	35.838

Lane 8 - 10A Bcl-xL

#	Vol. (Int.)	Local Bg. Corr. Vol.	Area	Rf	Density	Local Bg. Corr. Den.	% band purity	% lane purity	Mol. Wt.
1	16,300,935	13,624,566	507	0.484	32,151	26,872	100	66.793	36.378

iBright™ Image Analysis Report

28 January 2022

Figure 5B- deTyr-Tub

CHEMI_10212021_154810_1s_445ms_BC_PANEL_DETYR_TUB_PUCKOHI

Date:	10-21-2021 03:48:10PM
Mode:	Chemi Blots
Notes:	Fig5B- deTyr-Tub
Model:	FL1500
Instrument name:	2462619090234
Serial No:	2462619090234
Firmware version:	1.6.0
iBA version:	4.0.1
Image size:	676px X 540px
Image area:	
Optical Zoom:	2x
Digital Zoom:	1x
Focus level:	455
Resolution:	5 x 5
Exposure time:	1445 ms
Exposure mode:	Normal

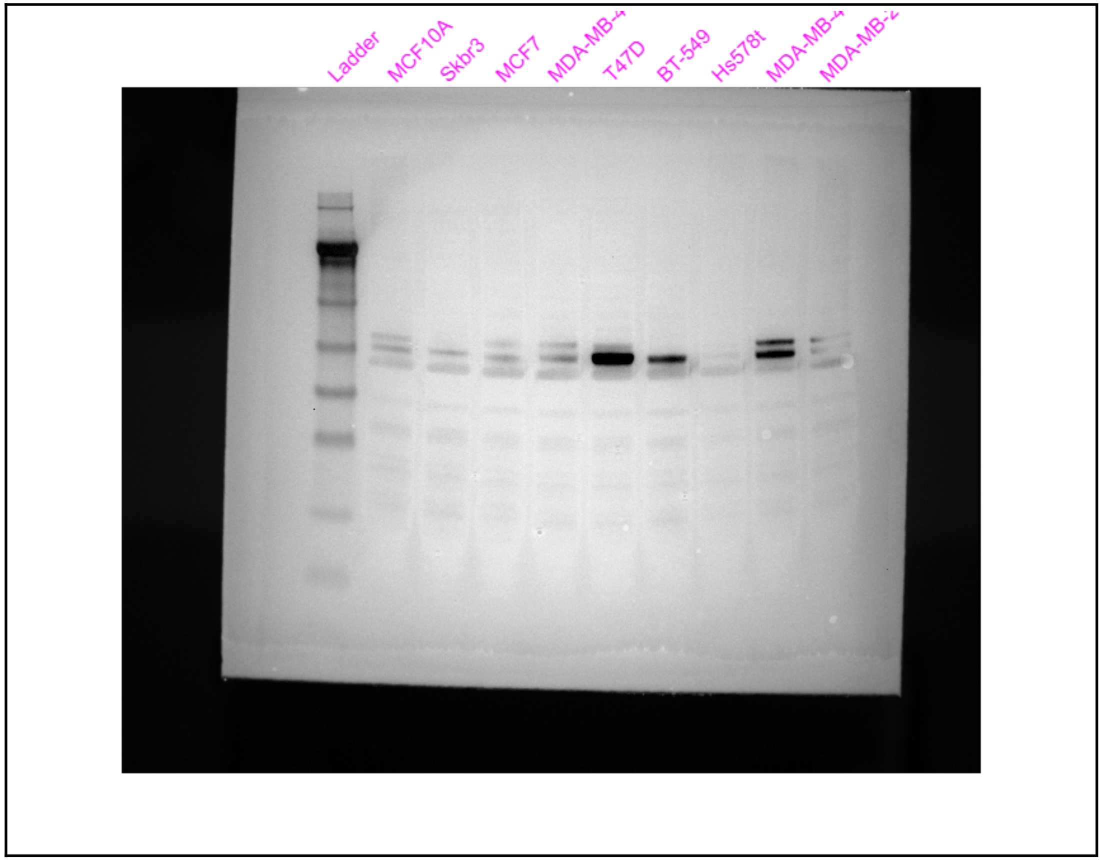

CHEMI_10212021_154810_1s_445ms_BC_PANEL_DETYR_TUB_PUCKOHI

Date:	10-21-2021 03:48:10PM
Mode:	Chemi Blots
Notes:	Fig5B- deTyr-Tub
Model:	FL1500
Instrument name:	2462619090234
Serial No:	2462619090234
Firmware version:	1.6.0
iBA version:	4.0.1
Image size:	676px X 540px
Image area:	
Optical Zoom:	2x
Digital Zoom:	1x
Focus level:	455
Resolution:	5 x 5
Exposure time:	1445 ms
Exposure mode:	Normal

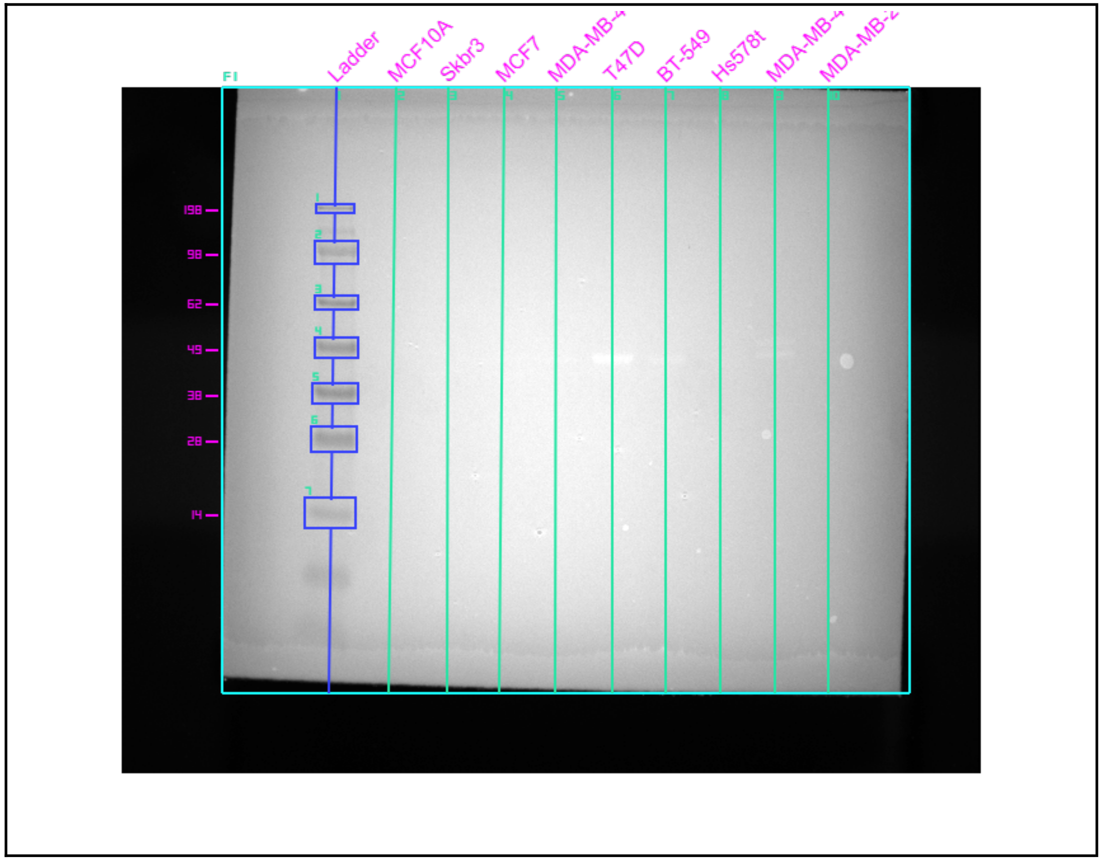

CHEMI_10212021_154810_1s_445ms_BC_PANEL_DETYR_TUB_PUCKOHI

Date:	10-21-2021 03:48:10PM
Mode:	Chemi Blots
Notes:	Fig5B- deTyr-Tub
Model:	FL1500
Instrument name:	2462619090234
Serial No:	2462619090234
Firmware version:	1.6.0
iBA version:	4.0.1
Image size:	676px X 540px
Image area:	
Optical Zoom:	2x
Digital Zoom:	1x
Focus level:	455
Resolution:	5 x 5
Exposure time:	1445 ms
Exposure mode:	Normal

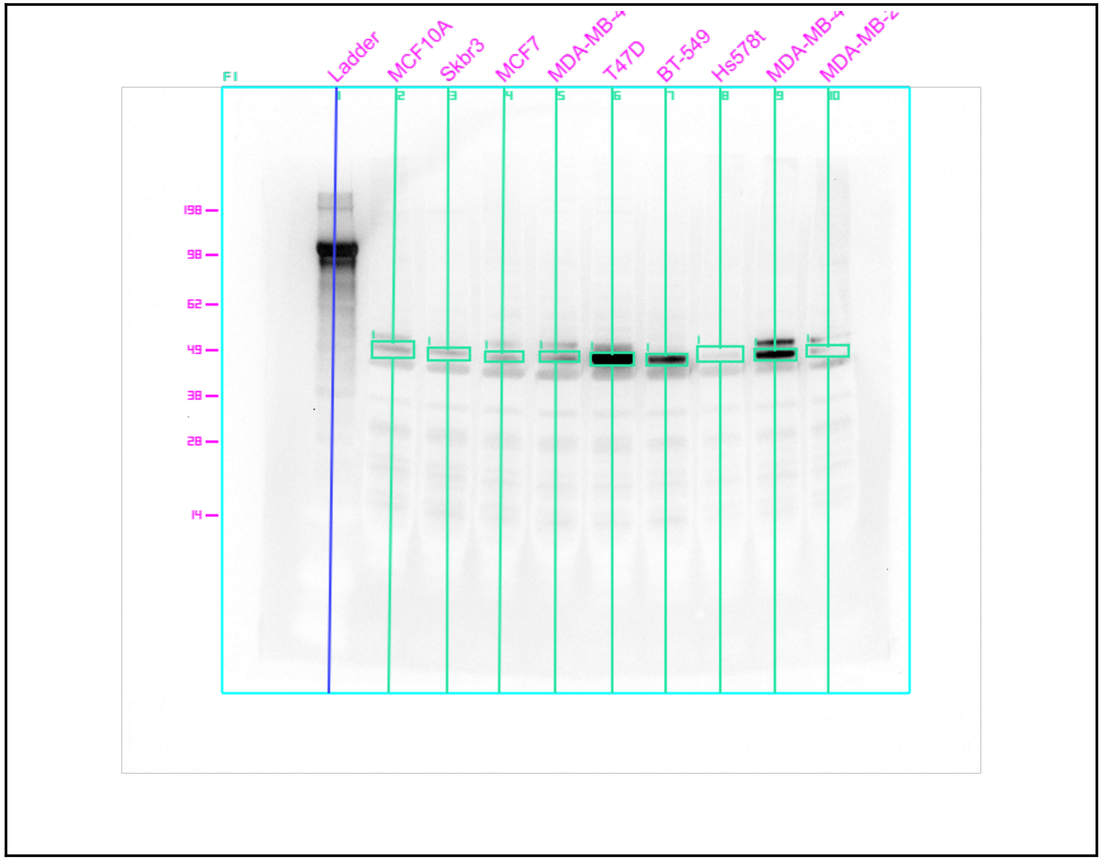

LANE AND BAND ANALYSIS DATA TABLE

CHEMI_10212021_154810_1s_445ms_BC_PANEL_DETYR_TUB_PUCKOHI

Frame: 1
Channel: Membrane
Sensitivity: 100
Molecular Weight Analysis Regression Method : Point to Point

Lane 1 - Ladder

#	Vol. (Int.)	Local Bg. Corr. Vol.	Area	Rf	Density	Local Bg. Corr. Den.	% band purity	% lane purity	Mol. Wt.
1	7,436,474	617,074	248	0.199	29,985	2,488.202	3.94	1.664	198
2	19,564,475	1,799,398	665	0.273	29,420	2,705.862	11.49	4.378	98
3	12,926,914	1,919,557	420	0.354	30,778	4,570.374	12.257	2.892	62
4	18,319,389	2,759,548	595	0.43	30,788	4,637.897	17.62	4.099	49
5	19,848,953	3,208,371	629	0.505	31,556	5,100.749	20.486	4.441	38
6	23,837,090	3,497,511	777	0.581	30,678	4,501.302	22.332	5.334	28
7	29,156,888	1,859,727	1,025	0.702	28,445	1,814.368	11.875	6.524	14

Frame: 1
Channel: Chemi
Sensitivity: 100
Molecular Weight Analysis Regression Method : Point to Point

Lane 2 - MCF10A

#	Vol. (Int.)	Local Bg. Corr. Vol.	Area	Rf	Density	Local Bg. Corr. Den.	% band purity	% lane purity	Mol. Wt.
1	1,955,639	548,416	476	0.432	4,108.485	1,152.136	100	9.685	48.694

Lane 3 - Skbr3

#	Vol. (Int.)	Local Bg. Corr. Vol.	Area	Rf	Density	Local Bg. Corr. Den.	% band purity	% lane purity	Mol. Wt.
1	1,389,431	472,575	374	0.44	3,715.056	1,263.572	100	7.756	47.472

Lane 4 - MCF7

#	Vol. (Int.)	Local Bg. Corr. Vol.	Area	Rf	Density	Local Bg. Corr. Den.	% band purity	% lane purity	Mol. Wt.
1	1,379,087	525,829	279	0.444	4,942.964	1,884.693	100	7.353	46.861

Lane 5 - MDA-MB-468

#	Vol. (Int.)	Local Bg. Corr. Vol.	Area	Rf	Density	Local Bg. Corr. Den.	% band purity	% lane purity	Mol. Wt.
1	2,070,729	852,173	288	0.444	7,190.031	2,958.935	100	10.377	46.861

Lane 6 - T47D

#	Vol. (Int.)	Local Bg. Corr. Vol.	Area	Rf	Density	Local Bg. Corr. Den.	% band purity	% lane purity	Mol. Wt.
1	11,901,025	9,180,442	385	0.449	30,911	23,845	100	39.845	46.25

Lane 7 - BT-549

#	Vol. (Int.)	Local Bg. Corr. Vol.	Area	Rf	Density	Local Bg. Corr. Den.	% band purity	% lane purity	Mol. Wt.
1	4,317,211	2,728,337	330	0.449	13,082	8,267.69	100	20.824	46.25

Lane 8 - Hs578t

#	Vol. (Int.)	Local Bg. Corr. Vol.	Area	Rf	Density	Local Bg. Corr. Den.	% band purity	% lane purity	Mol. Wt.
1	1,173,508	53,635	481	0.44	2,439.726	111.509	100	8.457	47.472

Lane 9 - MDA-MB-436

#	Vol. (Int.)	Local Bg. Corr. Vol.	Area	Rf	Density	Local Bg. Corr. Den.	% band purity	% lane purity	Mol. Wt.
1	5,199,011	3,148,659	340	0.44	15,291	9,260.763	100	23.111	47.472

Lane 10 - MDA-MB-231

#	Vol. (Int.)	Local Bg. Corr. Vol.	Area	Rf	Density	Local Bg. Corr. Den.	% band purity	% lane purity	Mol. Wt.
1	1,020,487	121,424	340	0.434	3,001.432	357.13	100	6.28	48.389

iBright™ Image Analysis Report

28 January 2022

Figure 5B- Alpha Tubulin

CHEMI_09302020_161833_950ms_BC_PANEL_ALPHA_TUB_INV

Date:	09-30-2020 04:18:33PM
Mode:	Chemi Blots
Notes:	Fig5B- alpha tubulin
Model:	FL1500
Instrument name:	2462619090234
Serial No:	2462619090234
Firmware version:	1.5.0
iBA version:	4.0.1
Image size:	676px X 540px
Image area:	
Optical Zoom:	1.8x
Digital Zoom:	1x
Focus level:	405
Resolution:	5 x 5
Exposure time:	950 ms
Exposure mode:	Normal

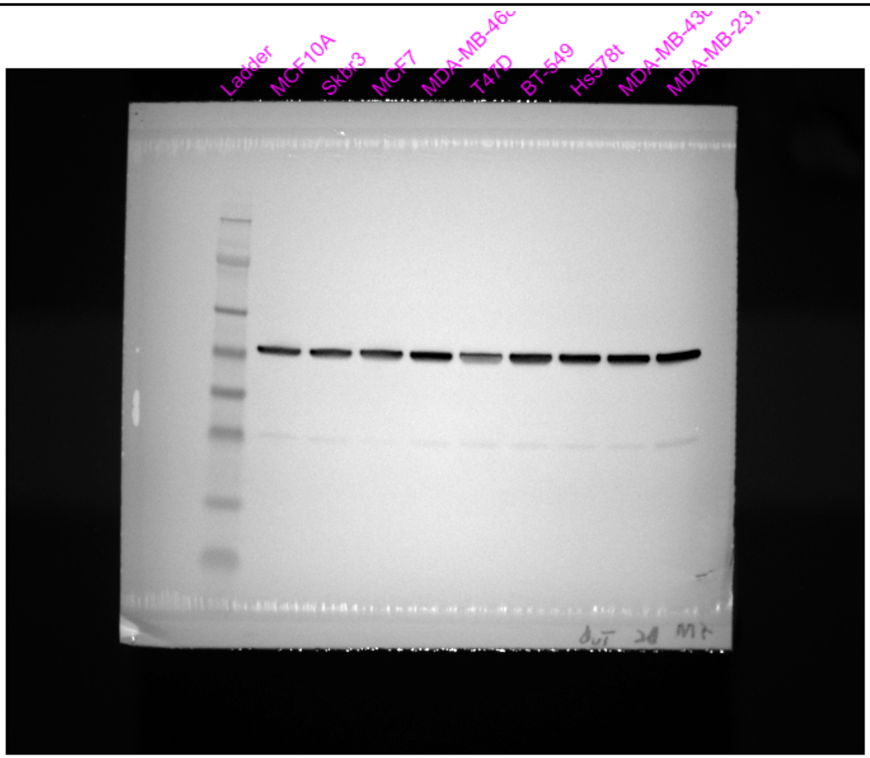

CHEMI_09302020_161833_950ms_BC_PANEL_ALPHA_TUB_INV

Date:	09-30-2020 04:18:33PM
Mode:	Chemi Blots
Notes:	Fig5B- alpha tubulin
Model:	FL1500
Instrument name:	2462619090234
Serial No:	2462619090234
Firmware version:	1.5.0
iBA version:	4.0.1
Image size:	676px X 540px
Image area:	
Optical Zoom:	1.8x
Digital Zoom:	1x
Focus level:	405
Resolution:	5 x 5
Exposure time:	950 ms
Exposure mode:	Normal

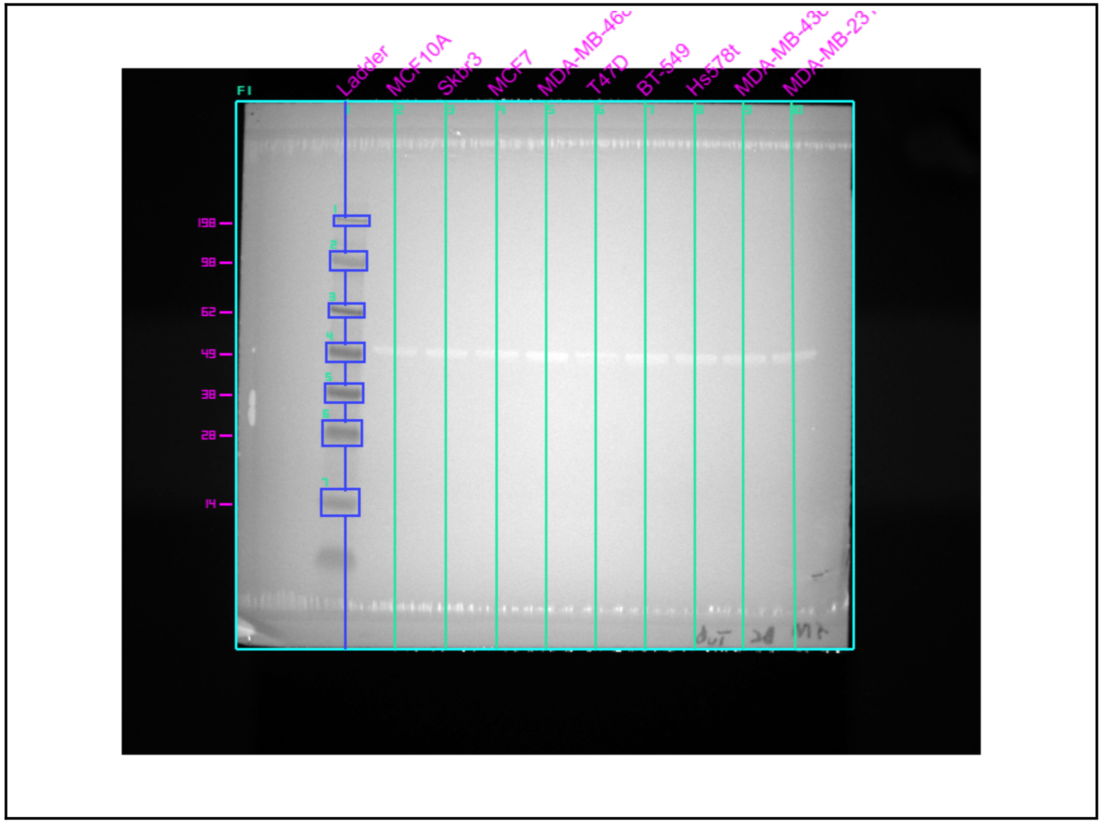

CHEMI_09302020_161833_950ms_BC_PANEL_ALPHA_TUB_INV

Date:	09-30-2020 04:18:33PM
Mode:	Chemi Blots
Notes:	Fig5B- alpha tubulin
Model:	FL1500
Instrument name:	2462619090234
Serial No:	2462619090234
Firmware version:	1.5.0
iBA version:	4.0.1
Image size:	676px X 540px
Image area:	
Optical Zoom:	1.8x
Digital Zoom:	1x
Focus level:	405
Resolution:	5 x 5
Exposure time:	950 ms
Exposure mode:	Normal

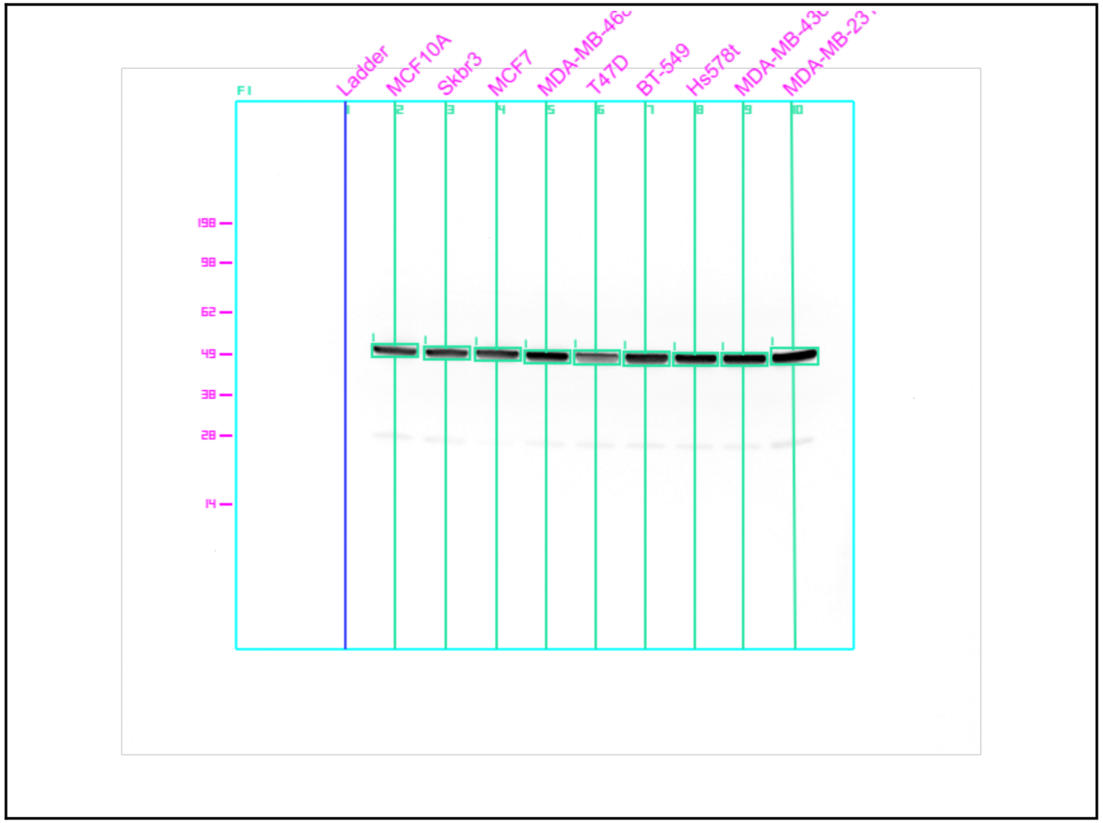

LANE AND BAND ANALYSIS DATA TABLE

CHEMI_09302020_161833_950ms_BC_PANEL_ALPHA_TUB_INV

Frame: 1
Channel: Membrane
Sensitivity: 100
Molecular Weight Analysis Regression Method : Point to Point

Lane 1 - Ladder

#	Vol. (Int.)	Local Bg. Corr. Vol.	Area	Rf	Density	Local Bg. Corr. Den.	% band purity	% lane purity	Mol. Wt.
1	7,876,874	713,102	261	0.218	30,179	2,732.194	4.44	1.777	198
2	14,810,733	1,609,449	480	0.29	30,855	3,353.019	10.021	3.34	98
3	11,394,291	2,009,764	348	0.381	32,742	5,775.187	12.513	2.57	62
4	16,561,949	3,122,458	496	0.457	33,391	6,295.278	19.441	3.735	49
5	16,672,235	3,184,017	496	0.531	33,613	6,419.39	19.824	3.76	38
6	21,913,567	3,392,902	672	0.606	32,609	5,048.962	21.125	4.942	28
7	21,199,779	2,029,620	682	0.731	31,084	2,975.983	12.637	4.781	14

Frame: 1
Channel: Chemi
Sensitivity: 100
Molecular Weight Analysis Regression Method : Point to Point

Lane 2 - MCF10A

#	Vol. (Int.)	Local Bg. Corr. Vol.	Area	Rf	Density	Local Bg. Corr. Den.	% band purity	% lane purity	Mol. Wt.
1	6,545,424	5,997,983	407	0.455	16,082	14,737	100	70.374	49.394

Lane 3 - Skbr3

#	Vol. (Int.)	Local Bg. Corr. Vol.	Area	Rf	Density	Local Bg. Corr. Den.	% band purity	% lane purity	Mol. Wt.
1	6,511,375	5,864,692	407	0.459	15,998	14,409	100	69.691	48.656

Lane 4 - MCF7

#	Vol. (Int.)	Local Bg. Corr. Vol.	Area	Rf	Density	Local Bg. Corr. Den.	% band purity	% lane purity	Mol. Wt.
1	6,934,791	6,017,816	407	0.462	17,038	14,785	100	69.076	48.312

Lane 5 - MDA-MB-468

#	Vol. (Int.)	Local Bg. Corr. Vol.	Area	Rf	Density	Local Bg. Corr. Den.	% band purity	% lane purity	Mol. Wt.
1	8,492,159	7,584,631	407	0.466	20,865	18,635	100	71.42	47.625

Lane 6 - T47D

#	Vol. (Int.)	Local Bg. Corr. Vol.	Area	Rf	Density	Local Bg. Corr. Den.	% band purity	% lane purity	Mol. Wt.
1	5,943,633	5,030,425	444	0.466	13,386	11,329	100	63.253	47.625

Lane 7 - BT-549

#	Vol. (Int.)	Local Bg. Corr. Vol.	Area	Rf	Density	Local Bg. Corr. Den.	% band purity	% lane purity	Mol. Wt.
1	8,597,282	7,599,454	444	0.469	19,363	17,115	100	70.413	47.281

Lane 8 - Hs578t

#	Vol. (Int.)	Local Bg. Corr. Vol.	Area	Rf	Density	Local Bg. Corr. Den.	% band purity	% lane purity	Mol. Wt.
1	8,577,570	7,493,022	396	0.471	21,660	18,921	100	67.338	46.937

Lane 9 - MDA-MB-436

#	Vol. (Int.)	Local Bg. Corr. Vol.	Area	Rf	Density	Local Bg. Corr. Den.	% band purity	% lane purity	Mol. Wt.
1	9,253,256	7,931,439	407	0.471	22,735	19,487	100	70.257	46.937

Lane 10 - MDA-MB-231

#	Vol. (Int.)	Local Bg. Corr. Vol.	Area	Rf	Density	Local Bg. Corr. Den.	% band purity	% lane purity	Mol. Wt.
1	11,189,338	10,408,668	532	0.464	21,032	19,565	100	77.845	47.969

iBright™ Image Analysis Report

28 January 2022

Figure 5B- VASH1

CHEMI_09292020_151642_350ms_BC_PANEL_VASH1

Date:	09-29-2020 03:16:42PM
Mode:	Chemi Blots
Notes:	Fig5B- VASH1
Model:	FL1500
Instrument name:	2462619090234
Serial No:	2462619090234
Firmware version:	1.5.0
iBA version:	4.0.1
Image size:	676px X 540px
Image area:	
Optical Zoom:	1.8x
Digital Zoom:	1x
Focus level:	405
Resolution:	5 x 5
Exposure time:	350 ms
Exposure mode:	Normal

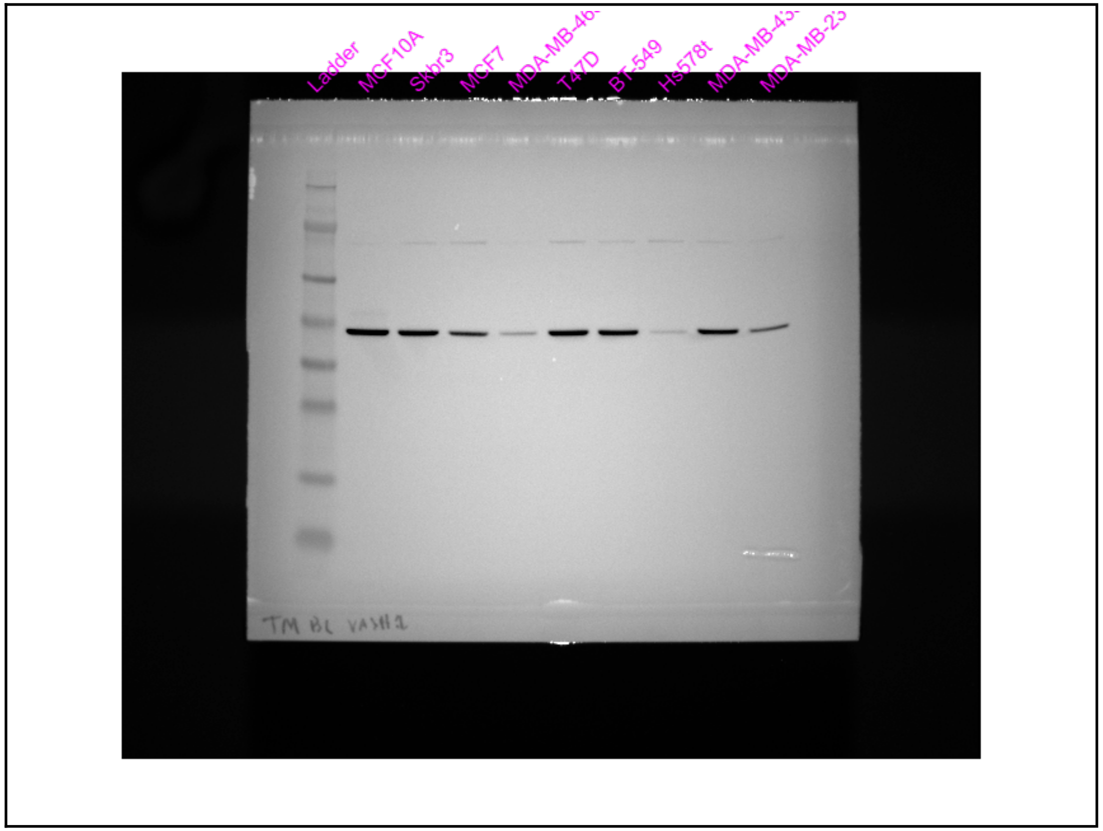

CHEMI_09292020_151642_350ms_BC_PANEL_VASH1

Date:	09-29-2020 03:16:42PM
Mode:	Chemi Blots
Notes:	Fig5B- VASH1
Model:	FL1500
Instrument name:	2462619090234
Serial No:	2462619090234
Firmware version:	1.5.0
iBA version:	4.0.1
Image size:	676px X 540px
Image area:	
Optical Zoom:	1.8x
Digital Zoom:	1x
Focus level:	405
Resolution:	5 x 5
Exposure time:	350 ms
Exposure mode:	Normal

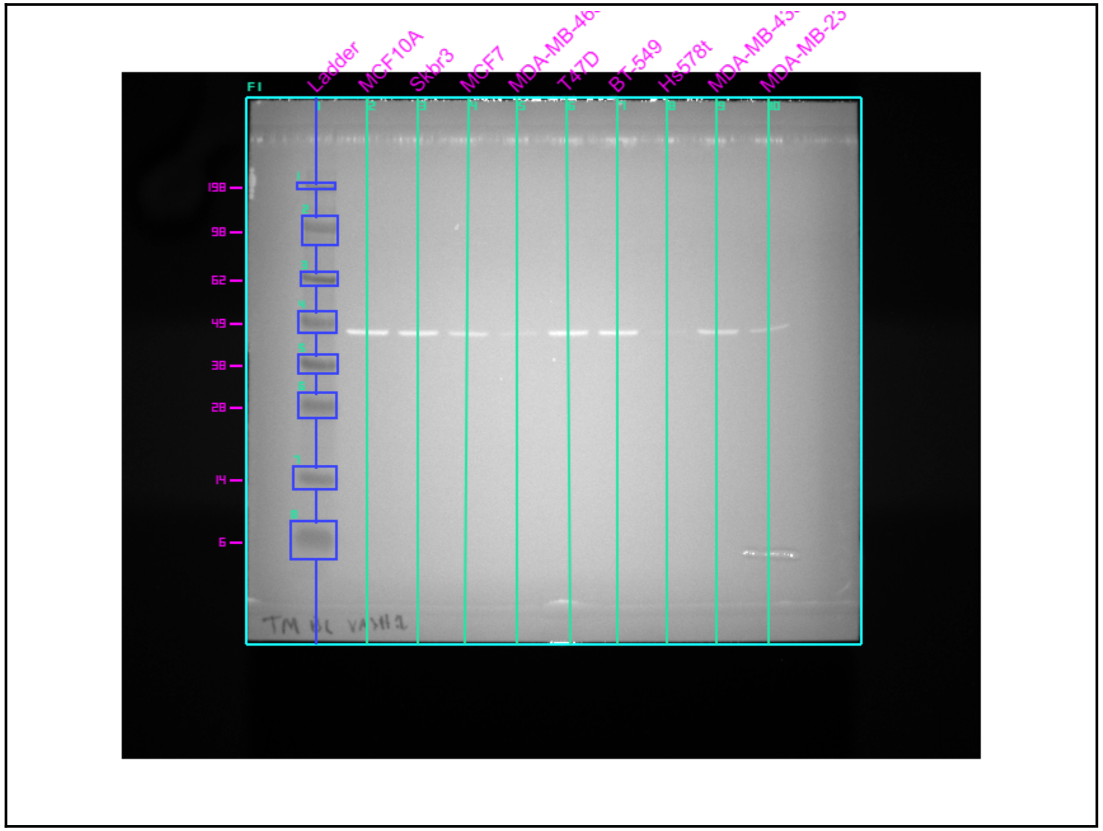

CHEMI_09292020_151642_350ms_BC_PANEL_VASH1

Date:	09-29-2020 03:16:42PM
Mode:	Chemi Blots
Notes:	Fig5B- VASH1
Model:	FL1500
Instrument name:	2462619090234
Serial No:	2462619090234
Firmware version:	1.5.0
iBA version:	4.0.1
Image size:	676px X 540px
Image area:	
Optical Zoom:	1.8x
Digital Zoom:	1x
Focus level:	405
Resolution:	5 x 5
Exposure time:	350 ms
Exposure mode:	Normal

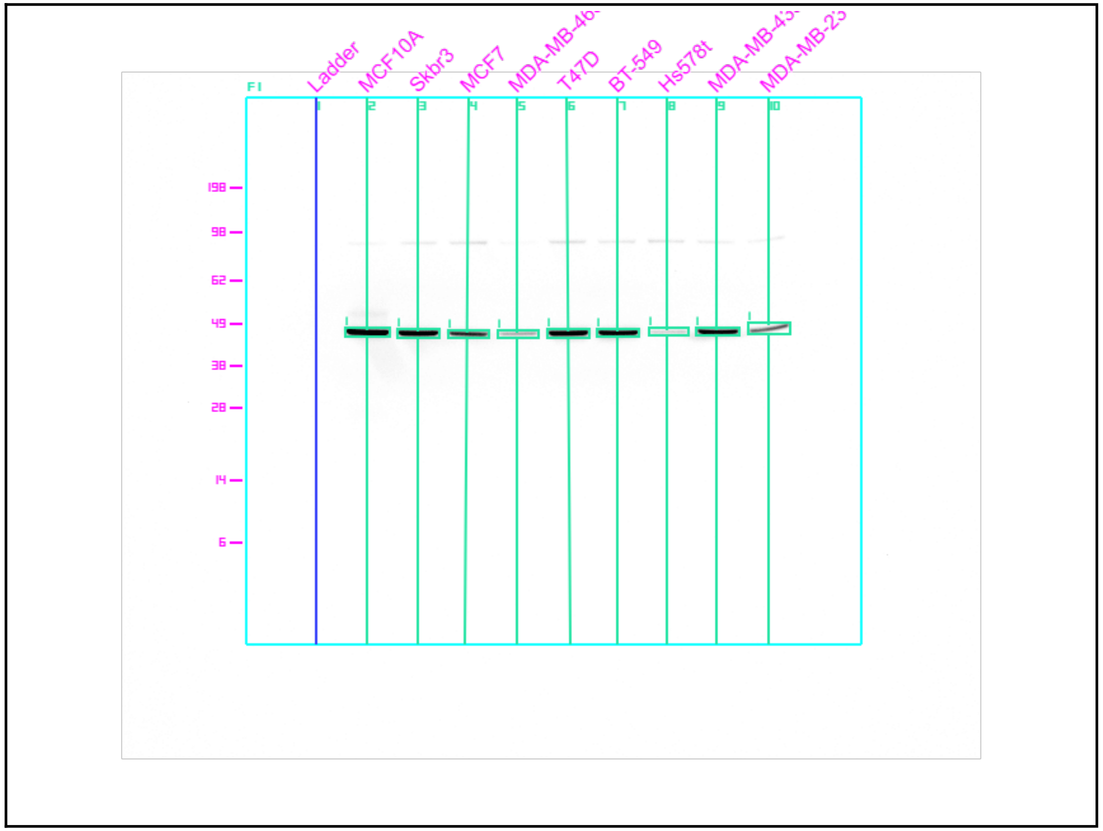

LANE AND BAND ANALYSIS DATA TABLE

CHEMI_09292020_151642_350ms_BC_PANEL_VASH1

Frame: 1
Channel: Membrane
Sensitivity: 100
Molecular Weight Analysis Regression Method : Point to Point

Lane 1 - Ladder

#	Vol. (Int.)	Local Bg. Corr. Vol.	Area	Rf	Density	Local Bg. Corr. Den.	% band purity	% lane purity	Mol. Wt.
1	6,117,315	466,934	186	0.16	32,888	2,510.403	2.723	1.454	198
2	22,416,774	2,064,271	696	0.242	32,208	2,965.908	12.039	5.326	98
3	12,087,073	1,798,545	360	0.33	33,575	4,995.961	10.489	2.872	62
4	17,756,343	2,155,623	558	0.409	31,821	3,863.125	12.571	4.219	49
5	16,718,008	2,588,829	512	0.486	32,652	5,056.308	15.098	3.972	38
6	20,770,022	2,664,767	651	0.563	31,904	4,093.345	15.541	4.935	28
7	20,641,413	2,146,009	665	0.695	31,039	3,227.081	12.515	4.905	14
8	36,241,538	3,261,980	1,147	0.809	31,596	2,843.924	19.024	8.611	6

Frame: 1
Channel: Chemi
Sensitivity: 100
Molecular Weight Analysis Regression Method : Point to Point

Lane 2 - MCF10A

#	Vol. (Int.)	Local Bg. Corr. Vol.	Area	Rf	Density	Local Bg. Corr. Den.	% band purity	% lane purity	Mol. Wt.
1	6,877,167	6,493,415	288	0.428	23,879	22,546	100	74.088	46.333

Lane 3 - Skbr3

#	Vol. (Int.)	Local Bg. Corr. Vol.	Area	Rf	Density	Local Bg. Corr. Den.	% band purity	% lane purity	Mol. Wt.
1	5,165,277	4,830,353	272	0.43	18,989	17,758	100	71.081	46

Lane 4 - MCF7

#	Vol. (Int.)	Local Bg. Corr. Vol.	Area	Rf	Density	Local Bg. Corr. Den.	% band purity	% lane purity	Mol. Wt.
1	2,757,410	2,543,929	231	0.433	11,936	11,012	100	61.822	45.667

Lane 5 - MDA-MB-468

#	Vol. (Int.)	Local Bg. Corr. Vol.	Area	Rf	Density	Local Bg. Corr. Den.	% band purity	% lane purity	Mol. Wt.
1	920,019	797,469	231	0.433	3,982.766	3,452.248	100	41.72	45.667

Lane 6 - T47D

#	Vol. (Int.)	Local Bg. Corr. Vol.	Area	Rf	Density	Local Bg. Corr. Den.	% band purity	% lane purity	Mol. Wt.
1	4,981,337	4,692,317	272	0.43	18,313	17,251	100	73.772	46

Lane 7 - BT-549

#	Vol. (Int.)	Local Bg. Corr. Vol.	Area	Rf	Density	Local Bg. Corr. Den.	% band purity	% lane purity	Mol. Wt.
1	4,608,799	4,310,246	238	0.43	19,364	18,110	100	72.267	46

Lane 8 - Hs578t

#	Vol. (Int.)	Local Bg. Corr. Vol.	Area	Rf	Density	Local Bg. Corr. Den.	% band purity	% lane purity	Mol. Wt.
1	523,844	404,871	224	0.428	2,338.589	1,807.462	100	27.87	46.333

Lane 9 - MDA-MB-436

#	Vol. (Int.)	Local Bg. Corr. Vol.	Area	Rf	Density	Local Bg. Corr. Den.	% band purity	% lane purity	Mol. Wt.
1	3,985,065	3,743,218	245	0.428	16,265	15,278	100	76.759	46.333

Lane 10 - MDA-MB-231

#	Vol. (Int.)	Local Bg. Corr. Vol.	Area	Rf	Density	Local Bg. Corr. Den.	% band purity	% lane purity	Mol. Wt.
1	1,777,596	1,699,076	340	0.421	5,228.224	4,997.284	100	66.696	47.333

iBright™ Image Analysis Report

28 January 2022

Figure 5B- VASH2

CHEMI_09292020_152037_1s_BC_PANEL_VASH2

Date:	09-29-2020 03:20:37PM
Mode:	Chemi Blots
Notes:	Figure5B- VASH2
Model:	FL1500
Instrument name:	2462619090234
Serial No:	2462619090234
Firmware version:	1.5.0
iBA version:	4.0.1
Image size:	676px X 540px
Image area:	
Optical Zoom:	1.8x
Digital Zoom:	1x
Focus level:	405
Resolution:	5 x 5
Exposure time:	1000 ms
Exposure mode:	Normal

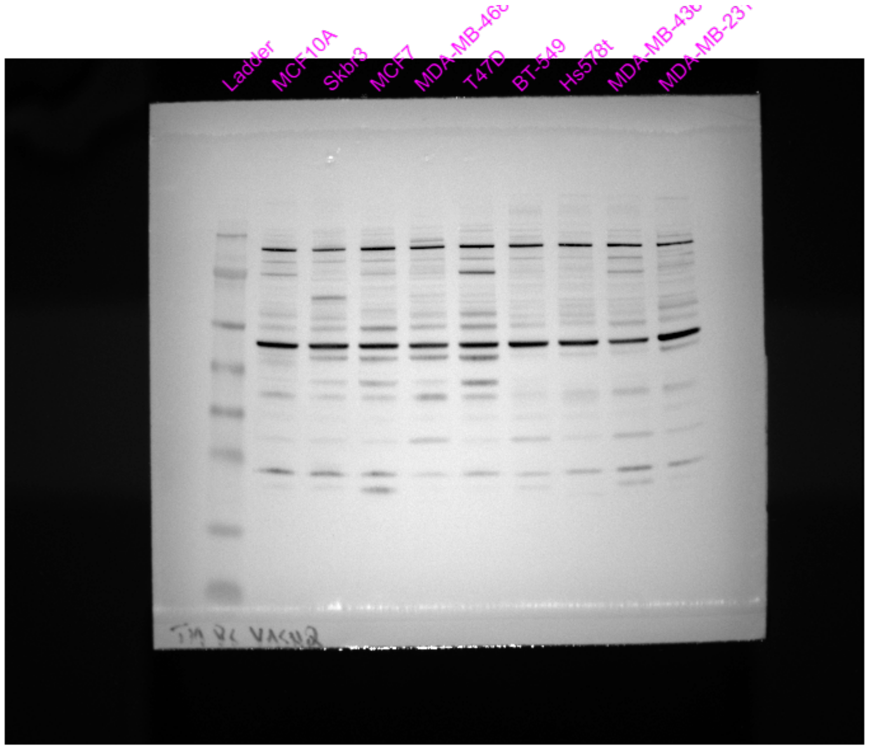

CHEMI_09292020_152037_1s_BC_PANEL_VASH2

Date:	09-29-2020 03:20:37PM
Mode:	Chemi Blots
Notes:	Figure5B- VASH2
Model:	FL1500
Instrument name:	2462619090234
Serial No:	2462619090234
Firmware version:	1.5.0
iBA version:	4.0.1
Image size:	676px X 540px
Image area:	
Optical Zoom:	1.8x
Digital Zoom:	1x
Focus level:	405
Resolution:	5 x 5
Exposure time:	1000 ms
Exposure mode:	Normal

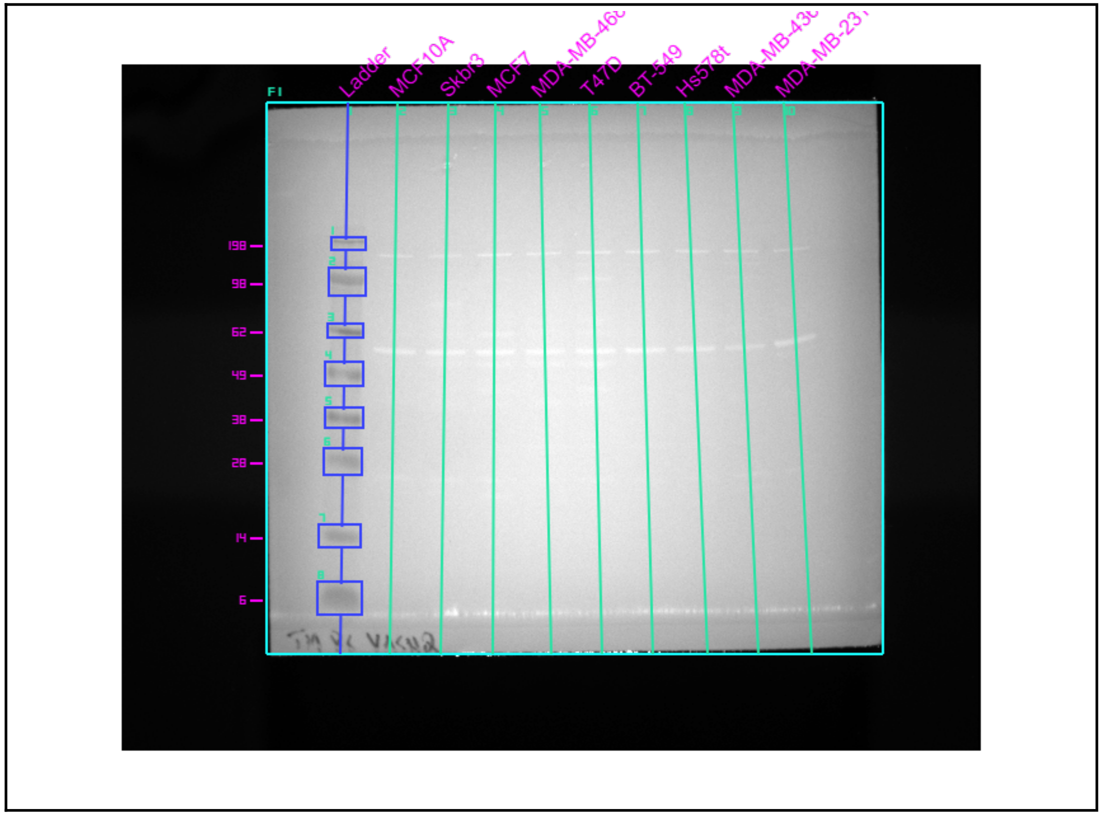

CHEMI_09292020_152037_1s_BC_PANEL_VASH2

Date:	09-29-2020 03:20:37PM
Mode:	Chemi Blots
Notes:	Figure5B- VASH2
Model:	FL1500
Instrument name:	2462619090234
Serial No:	2462619090234
Firmware version:	1.5.0
iBA version:	4.0.1
Image size:	676px X 540px
Image area:	
Optical Zoom:	1.8x
Digital Zoom:	1x
Focus level:	405
Resolution:	5 x 5
Exposure time:	1000 ms
Exposure mode:	Normal

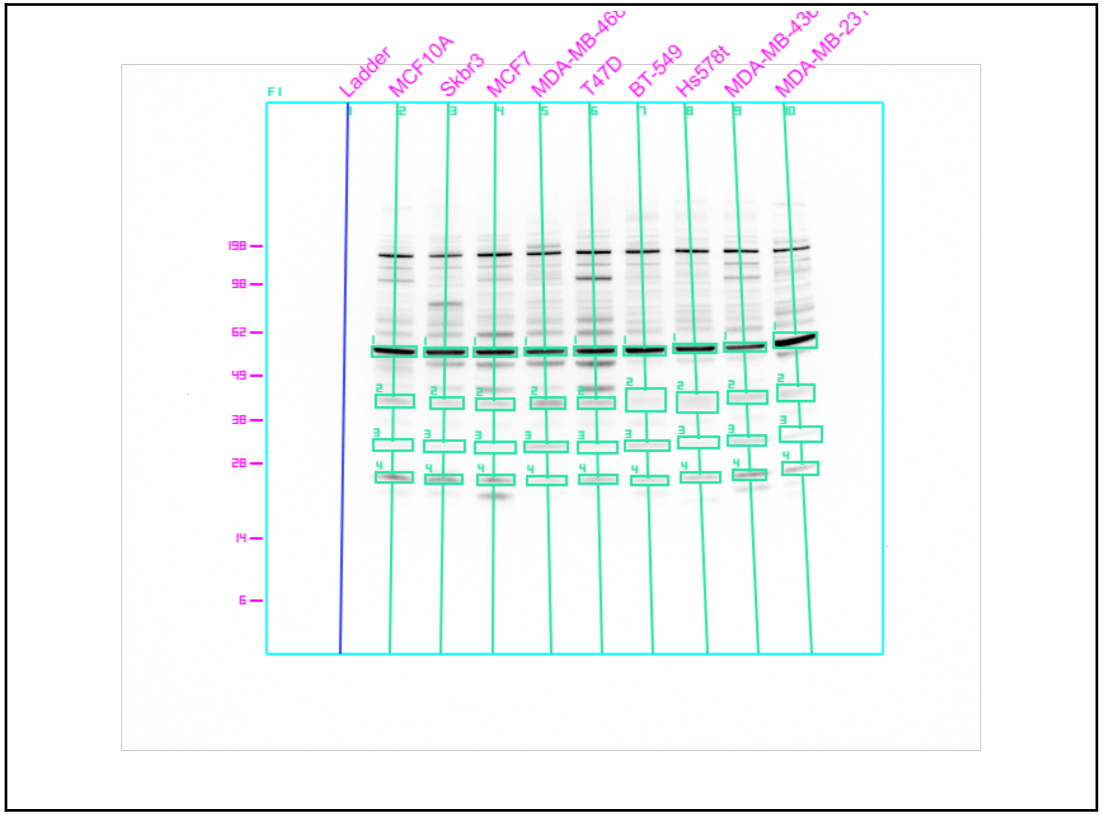

LANE AND BAND ANALYSIS DATA TABLE

CHEMI_09292020_152037_1s_BC_PANEL_VASH2

Frame: 1
Channel: Membrane
Sensitivity: 100
Molecular Weight Analysis Regression Method : Point to Point

Lane 1 - Ladder

#	Vol. (Int.)	Local Bg. Corr. Vol.	Area	Rf	Density	Local Bg. Corr. Den.	% band purity	% lane purity	Mol. Wt.
1	9,221,505	767,014	308	0.256	29,939	2,490.308	5.019	2.435	198
2	20,737,848	2,132,477	690	0.325	30,054	3,090.548	13.954	5.475	98
3	10,878,346	1,700,211	348	0.412	31,259	4,885.666	11.125	2.872	62
4	18,341,913	2,043,888	620	0.491	29,583	3,296.594	13.374	4.843	49
5	16,165,533	2,213,611	527	0.571	30,674	4,200.402	14.485	4.268	38
6	20,331,186	2,042,995	682	0.65	29,811	2,995.594	13.368	5.368	28
7	19,409,284	1,732,167	646	0.786	30,045	2,681.373	11.334	5.125	14
8	31,443,890	2,650,238	972	0.899	32,349	2,726.583	17.342	8.302	6

Frame: 1
Channel: Chemi
Sensitivity: 100
Molecular Weight Analysis Regression Method : Point to Point

Lane 2 - MCF10A

#	Vol. (Int.)	Local Bg. Corr. Vol.	Area	Rf	Density	Local Bg. Corr. Den.	% band purity	% lane purity	Mol. Wt.
1	5,968,243	5,069,413	324	0.452	18,420	15,646	67.741	24.506	55.5
2	1,274,099	842,745	341	0.541	3,736.361	2,471.394	11.261	5.231	42.086
3	394,135	242,814	320	0.62	1,231.672	758.795	3.245	1.618	31.824
4	1,590,473	1,328,576	270	0.68	5,890.641	4,920.654	17.753	6.531	24.915

Lane 3 - Skbr3

#	Vol. (Int.)	Local Bg. Corr. Vol.	Area	Rf	Density	Local Bg. Corr. Den.	% band purity	% lane purity	Mol. Wt.
1	4,905,594	3,934,047	280	0.452	17,519	14,050	68.517	19.573	55.5
2	787,555	426,532	270	0.544	2,916.87	1,579.748	7.429	3.142	41.771

#	Vol. (Int.)	Local Bg. Corr. Vol.	Area	Rf	Density	Local Bg. Corr. Den.	% band purity	% lane purity	Mol. Wt.
3	425,693	238,495	330	0.622	1,289.979	722.713	4.154	1.698	31.529
4	1,429,668	1,142,674	240	0.682	5,956.95	4,761.143	19.901	5.704	24.678

Lane 4 - MCF7

#	Vol. (Int.)	Local Bg. Corr. Vol.	Area	Rf	Density	Local Bg. Corr. Den.	% band purity	% lane purity	Mol. Wt.
1	4,879,125	4,000,845	280	0.452	17,425	14,288	68.774	17.293	55.5
2	979,544	543,969	310	0.546	3,159.819	1,754.739	9.351	3.472	41.457
3	435,622	241,815	330	0.624	1,320.067	732.775	4.157	1.544	31.235
4	1,278,991	1,030,739	270	0.684	4,737.004	3,817.553	17.718	4.533	24.441

Lane 5 - MDA-MB-468

#	Vol. (Int.)	Local Bg. Corr. Vol.	Area	Rf	Density	Local Bg. Corr. Den.	% band purity	% lane purity	Mol. Wt.
1	4,036,832	3,172,264	238	0.452	16,961	13,328	57.887	16.166	55.5
2	1,601,557	1,198,885	280	0.544	5,719.846	4,281.735	21.877	6.414	41.771
3	928,471	732,529	315	0.624	2,947.527	2,325.489	13.367	3.718	31.235
4	504,352	376,461	256	0.684	1,970.125	1,470.553	6.87	2.02	24.441

Lane 6 - T47D

#	Vol. (Int.)	Local Bg. Corr. Vol.	Area	Rf	Density	Local Bg. Corr. Den.	% band purity	% lane purity	Mol. Wt.
1	5,129,311	3,994,221	272	0.449	18,857	14,684	70.755	16.189	55.882
2	1,288,652	696,378	300	0.544	4,295.507	2,321.263	12.336	4.067	41.771
3	408,111	236,920	320	0.624	1,275.347	740.375	4.197	1.288	31.235
4	875,588	717,649	248	0.682	3,530.597	2,893.75	12.713	2.763	24.678

Lane 7 - BT-549

#	Vol. (Int.)	Local Bg. Corr. Vol.	Area	Rf	Density	Local Bg. Corr. Den.	% band purity	% lane purity	Mol. Wt.
1	5,300,534	4,597,684	272	0.449	19,487	16,903	74.658	23.507	55.882
2	1,191,075	545,169	608	0.539	1,959.005	896.66	8.853	5.282	42.4
3	768,452	609,091	324	0.622	2,371.765	1,879.913	9.891	3.408	31.529
4	563,415	406,363	240	0.684	2,347.563	1,693.183	6.599	2.499	24.441

Lane 8 - Hs578t

#	Vol. (Int.)	Local Bg. Corr. Vol.	Area	Rf	Density	Local Bg. Corr. Den.	% band purity	% lane purity	Mol. Wt.
1	4,849,837	4,262,527	280	0.445	17,320	15,223	70.821	23.067	56.647
2	1,360,333	817,736	561	0.544	2,424.836	1,457.64	13.586	6.47	41.771
3	434,366	255,884	330	0.615	1,316.261	775.407	4.251	2.066	32.412
4	810,567	682,610	288	0.68	2,814.469	2,370.175	11.341	3.855	24.915

Lane 9 - MDA-MB-436

#	Vol. (Int.)	Local Bg. Corr. Vol.	Area	Rf	Density	Local Bg. Corr. Den.	% band purity	% lane purity	Mol. Wt.
1	3,731,584	3,166,733	272	0.442	13,719	11,642	56.356	17.487	57.029
2	1,123,508	768,960	352	0.535	3,191.784	2,184.547	13.685	5.265	43.029
3	757,574	600,115	279	0.613	2,715.319	2,150.95	10.68	3.55	32.706
4	1,336,765	1,083,371	243	0.675	5,501.091	4,458.319	19.28	6.264	25.39

Lane 10 - MDA-MB-231

#	Vol. (Int.)	Local Bg. Corr. Vol.	Area	Rf	Density	Local Bg. Corr. Den.	% band purity	% lane purity	Mol. Wt.
1	8,021,843	6,948,948	455	0.431	17,630	15,272	80.991	36.718	58.941
2	974,489	627,029	420	0.525	2,320.212	1,492.927	7.308	4.46	44.286
3	454,125	307,471	442	0.601	1,027.432	695.637	3.584	2.079	34.176
4	834,108	696,430	319	0.664	2,614.759	2,183.166	8.117	3.818	26.576

iBright™ Image Analysis Report

28 January 2022

Figure 5B- SVBP

CHEMI_10212021_154347_15s_BC_PANEL_INV_SVBP

Date:	10-21-2021 03:43:47PM
Mode:	Chemi Blots
Notes:	Fig5B- SVBP
Model:	FL1500
Instrument name:	2462619090234
Serial No:	2462619090234
Firmware version:	1.6.0
iBA version:	4.0.1
Image size:	676px X 540px
Image area:	
Optical Zoom:	1.9x
Digital Zoom:	1x
Focus level:	430
Resolution:	5 x 5
Exposure time:	15000 ms
Exposure mode:	Normal

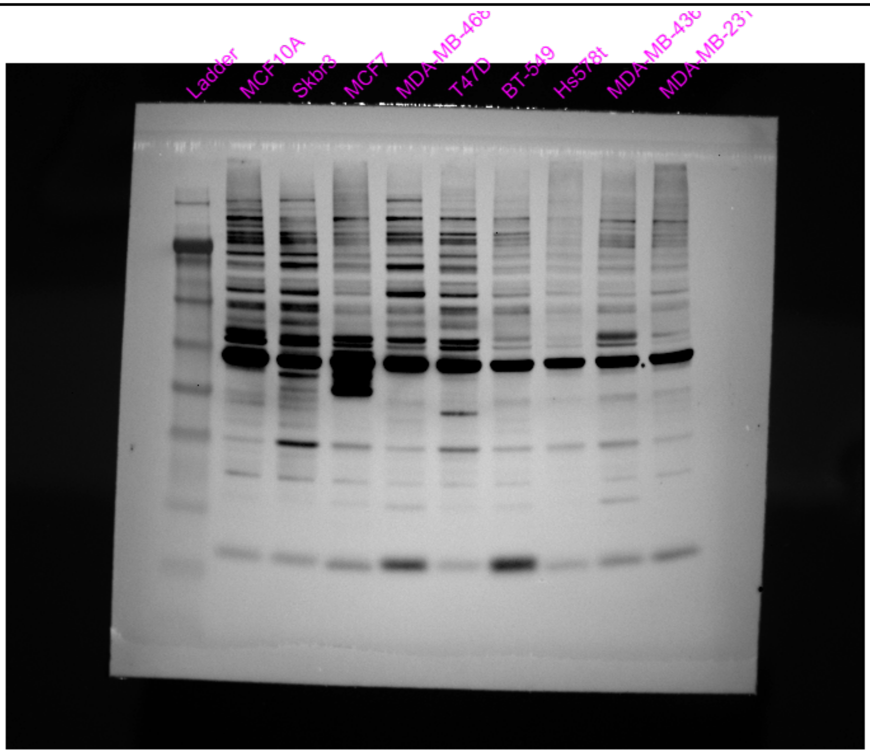

CHEMI_10212021_154347_15s_BC_PANEL_INV_SVBP

Date:	10-21-2021 03:43:47PM
Mode:	Chemi Blots
Notes:	Fig5B- SVBP
Model:	FL1500
Instrument name:	2462619090234
Serial No:	2462619090234
Firmware version:	1.6.0
iBA version:	4.0.1
Image size:	676px X 540px
Image area:	
Optical Zoom:	1.9x
Digital Zoom:	1x
Focus level:	430
Resolution:	5 x 5
Exposure time:	15000 ms
Exposure mode:	Normal

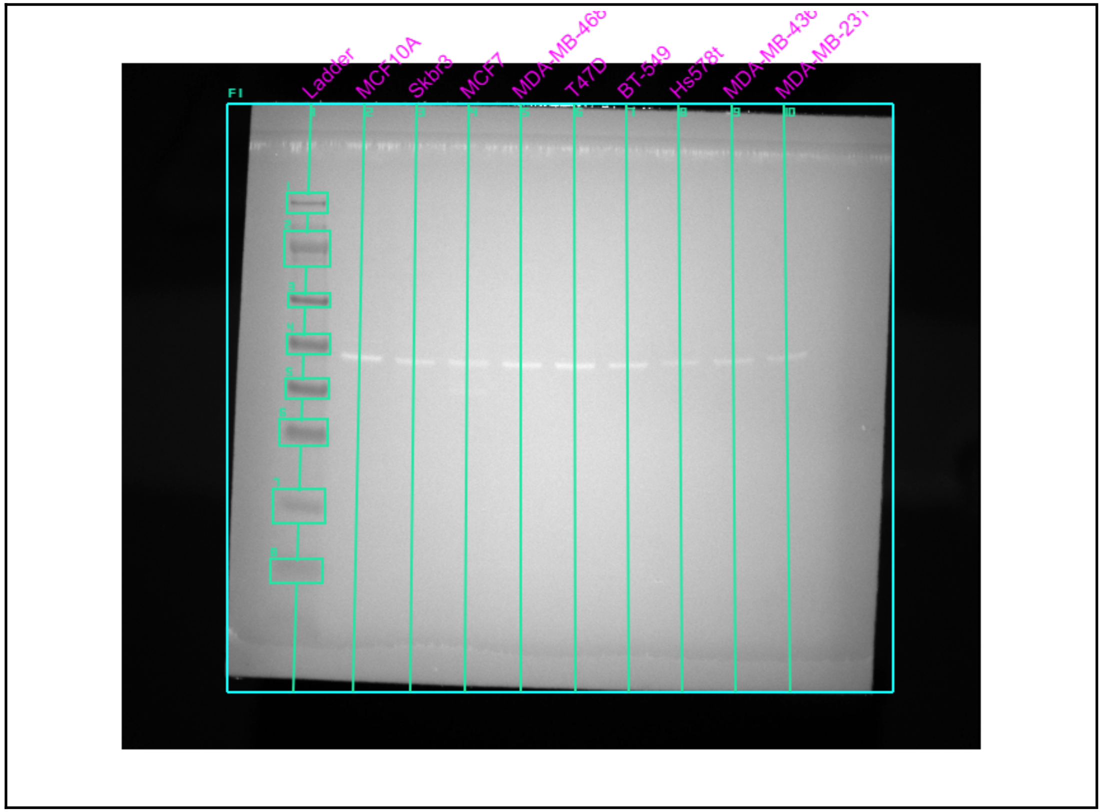

CHEMI_10212021_154347_15s_BC_PANEL_INV_SVBP

Date:	10-21-2021 03:43:47PM
Mode:	Chemi Blots
Notes:	Fig5B- SVBP
Model:	FL1500
Instrument name:	2462619090234
Serial No:	2462619090234
Firmware version:	1.6.0
iBA version:	4.0.1
Image size:	676px X 540px
Image area:	
Optical Zoom:	1.9x
Digital Zoom:	1x
Focus level:	430
Resolution:	5 x 5
Exposure time:	15000 ms
Exposure mode:	Normal

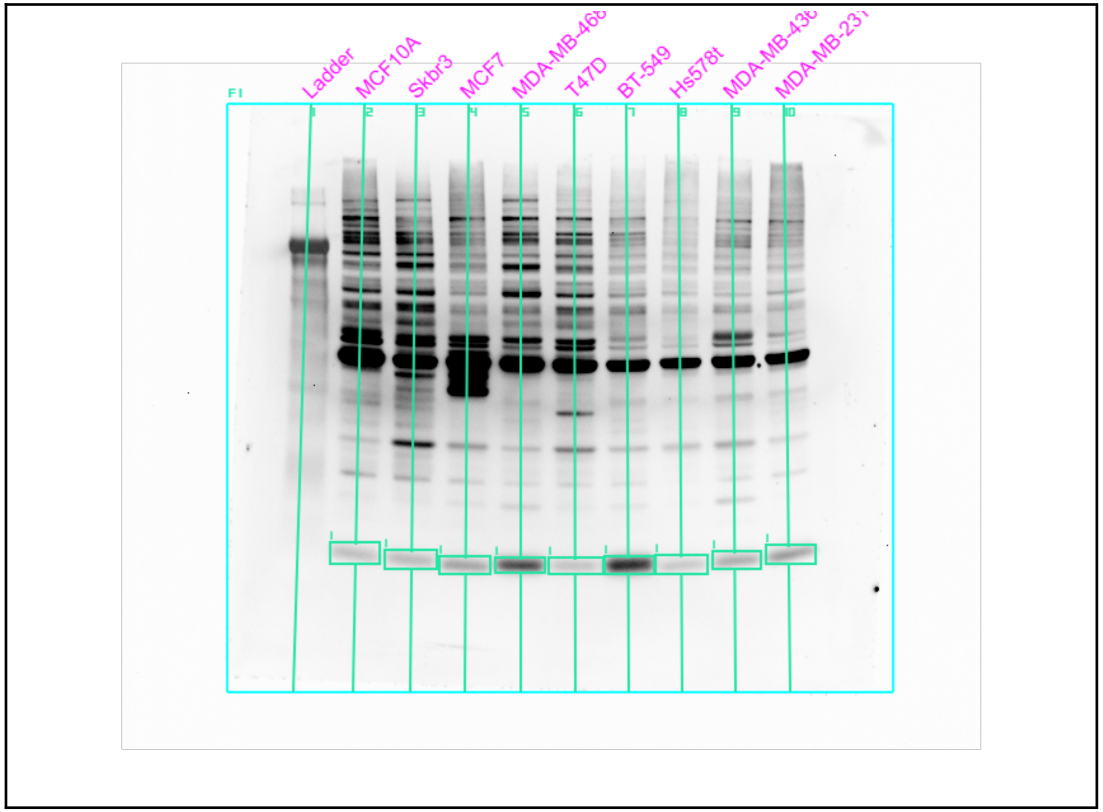

LANE AND BAND ANALYSIS DATA TABLE

CHEMI_10212021_154347_15s_BC_PANEL_INV_SVBP

Frame: 1
Channel: Membrane
Sensitivity: 100

Lane 1 - Ladder

#	Vol. (Int.)	Local Bg. Corr. Vol.	Area	Rf	Density	Local Bg. Corr. Den.	% band purity	% lane purity
1	17,061,728	871,742	561	0.168	30,413	1,553.909	5.003	3.746
2	32,961,571	2,246,057	1,073	0.246	30,719	2,093.25	12.89	7.237
3	13,268,284	2,056,163	408	0.333	32,520	5,039.617	11.801	2.913
4	19,152,516	2,882,359	595	0.408	32,189	4,844.301	16.542	4.205
5	19,493,810	3,015,103	595	0.484	32,762	5,067.401	17.304	4.28
6	27,185,888	3,235,152	858	0.557	31,685	3,770.574	18.567	5.969
7	35,035,506	1,739,506	1,176	0.683	29,792	1,479.172	9.983	7.693
8	26,658,793	1,378,153	840	0.793	31,736	1,640.659	7.909	5.853

Frame: 1
Channel: Chemi
Sensitivity: 100

Lane 2 - MCF10A

#	Vol. (Int.)	Local Bg. Corr. Vol.	Area	Rf	Density	Local Bg. Corr. Den.	% band purity	% lane purity
1	6,459,684	3,531,098	720	0.762	8,971.783	4,904.304	100	2.532

Lane 3 - Skbr3

#	Vol. (Int.)	Local Bg. Corr. Vol.	Area	Rf	Density	Local Bg. Corr. Den.	% band purity	% lane purity
1	6,693,966	3,475,275	672	0.773	9,961.259	5,171.541	100	2.467

Lane 4 - MCF7

#	Vol. (Int.)	Local Bg. Corr. Vol.	Area	Rf	Density	Local Bg. Corr. Den.	% band purity	% lane purity
1	8,063,504	4,525,414	615	0.784	13,111	7,358.397	100	3.308

Lane 5 - MDA-MB-468

#	Vol. (Int.)	Local Bg. Corr. Vol.	Area	Rf	Density	Local Bg. Corr. Den.	% band purity	% lane purity
1	14,415,048	10,390,877	520	0.784	27,721	19,982	100	6.333

Lane 6 - T47D

#	Vol. (Int.)	Local Bg. Corr. Vol.	Area	Rf	Density	Local Bg. Corr. Den.	% band purity	% lane purity
1	5,881,704	1,530,141	616	0.784	9,548.221	2,483.996	100	2.789

Lane 7 - BT-549

#	Vol. (Int.)	Local Bg. Corr. Vol.	Area	Rf	Density	Local Bg. Corr. Den.	% band purity	% lane purity
1	19,217,061	14,553,767	600	0.784	32,028	24,256	100	12.281

Lane 8 - Hs578t

#	Vol. (Int.)	Local Bg. Corr. Vol.	Area	Rf	Density	Local Bg. Corr. Den.	% band purity	% lane purity
1	5,226,224	1,740,529	672	0.782	7,777.119	2,590.074	100	5.008

Lane 9 - MDA-MB-436

#	Vol. (Int.)	Local Bg. Corr. Vol.	Area	Rf	Density	Local Bg. Corr. Den.	% band purity	% lane purity
1	6,367,732	4,200,663	585	0.775	10,885	7,180.622	100	4.569

Lane 10 - MDA-MB-231

#	Vol. (Int.)	Local Bg. Corr. Vol.	Area	Rf	Density	Local Bg. Corr. Den.	% band purity	% lane purity
1	7,486,061	5,475,573	640	0.765	11,696	8,555.584	100	6.091

iBright™ Image Analysis Report

28 January 2022

Figure 5B- GAPDH

CHEMI_09302020_162150_1s_100ms_BC_PANEL_GAPDH

Date:	09-30-2020 04:21:50PM
Mode:	Chemi Blots
Notes:	Fig5B- GAPDH on VASH1
Model:	FL1500
Instrument name:	2462619090234
Serial No:	2462619090234
Firmware version:	1.5.0
iBA version:	4.0.1
Image size:	676px X 540px
Image area:	
Optical Zoom:	1.9x
Digital Zoom:	1x
Focus level:	430
Resolution:	5 x 5
Exposure time:	1100 ms
Exposure mode:	Normal

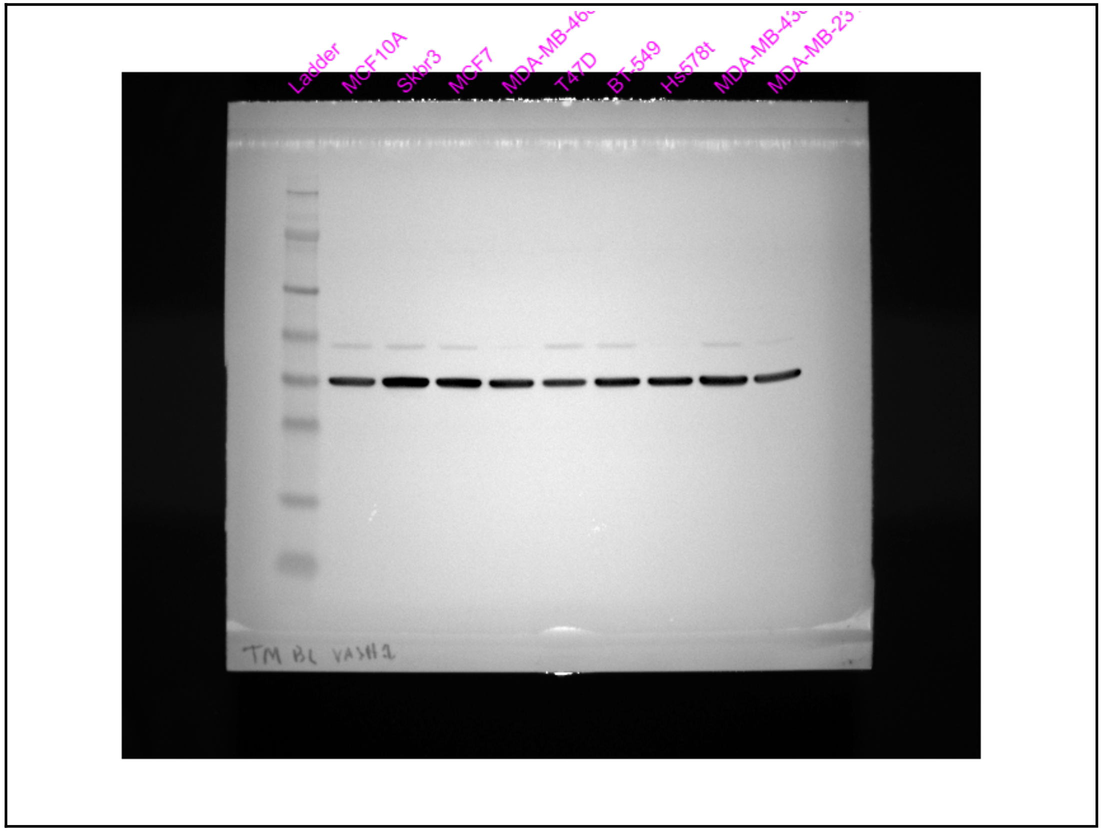

CHEMI_09302020_162150_1s_100ms_BC_PANEL_GAPDH

Date:	09-30-2020 04:21:50PM
Mode:	Chemi Blots
Notes:	Fig5B- GAPDH on VASH1
Model:	FL1500
Instrument name:	2462619090234
Serial No:	2462619090234
Firmware version:	1.5.0
iBA version:	4.0.1
Image size:	676px X 540px
Image area:	
Optical Zoom:	1.9x
Digital Zoom:	1x
Focus level:	430
Resolution:	5 x 5
Exposure time:	1100 ms
Exposure mode:	Normal

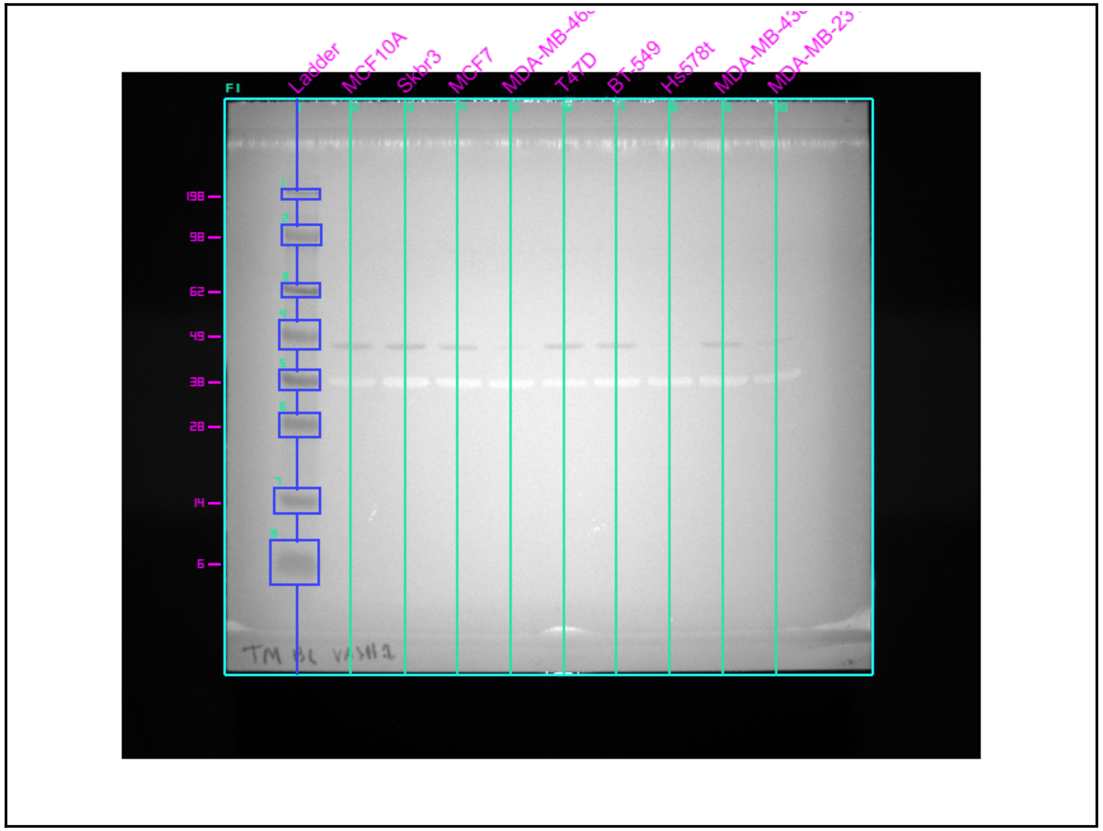

CHEMI_09302020_162150_1s_100ms_BC_PANEL_GAPDH

Date:	09-30-2020 04:21:50PM
Mode:	Chemi Blots
Notes:	Fig5B- GAPDH on VASH1
Model:	FL1500
Instrument name:	2462619090234
Serial No:	2462619090234
Firmware version:	1.5.0
iBA version:	4.0.1
Image size:	676px X 540px
Image area:	
Optical Zoom:	1.9x
Digital Zoom:	1x
Focus level:	430
Resolution:	5 x 5
Exposure time:	1100 ms
Exposure mode:	Normal

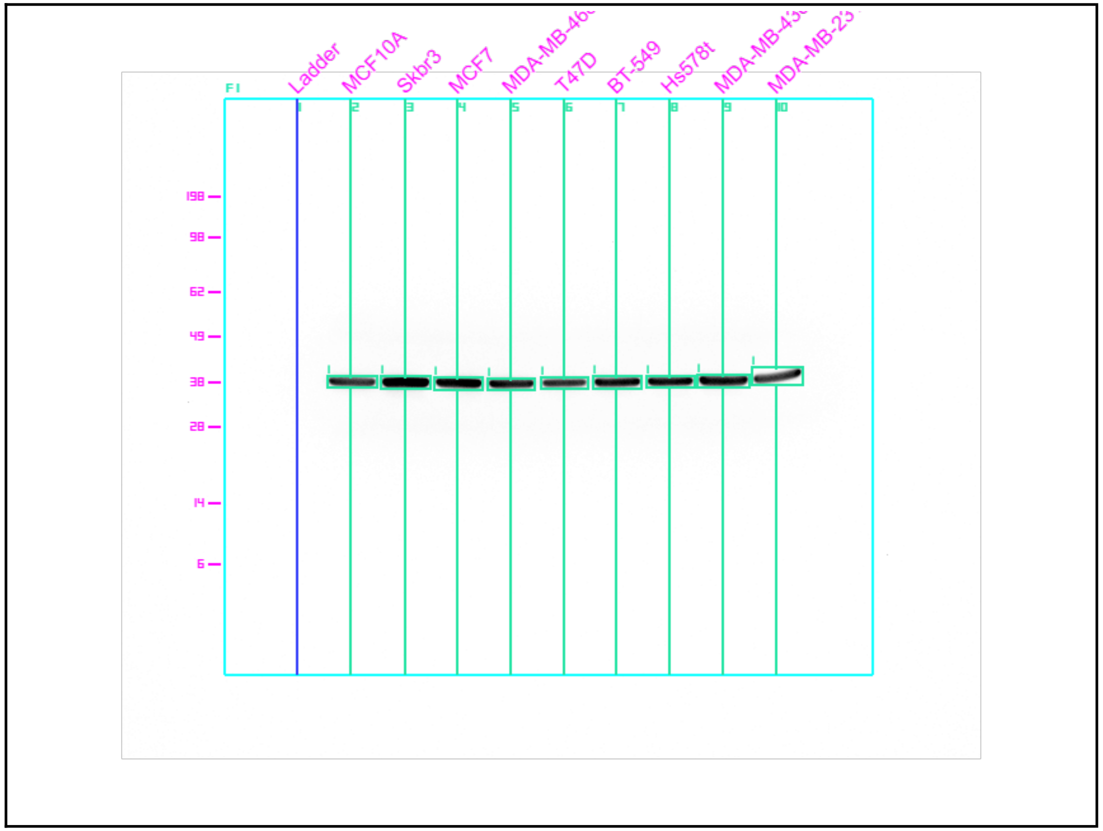

LANE AND BAND ANALYSIS DATA TABLE

CHEMI_09302020_162150_1s_100ms_BC_PANEL_GAPDH

Frame: 1
Channel: Membrane
Sensitivity: 100
Molecular Weight Analysis Regression Method : Point to Point

Lane 1 - Ladder

#	Vol. (Int.)	Local Bg. Corr. Vol.	Area	Rf	Density	Local Bg. Corr. Den.	% band purity	% lane purity	Mol. Wt.
1	8,966,646	655,206	279	0.166	32,138	2,348.411	3.709	1.735	198
2	17,588,409	1,520,281	544	0.236	32,331	2,794.634	8.607	3.403	98
3	12,540,592	1,867,135	372	0.331	33,711	5,019.18	10.571	2.426	62
4	24,701,361	2,573,443	792	0.408	31,188	3,249.297	14.569	4.779	49
5	18,417,436	2,772,757	561	0.488	32,829	4,942.527	15.698	3.564	38
6	21,252,960	2,738,198	660	0.565	32,201	4,148.786	15.502	4.112	28
7	24,186,476	2,269,469	777	0.698	31,128	2,920.811	12.849	4.68	14
8	44,533,051	3,266,773	1,404	0.804	31,718	2,326.762	18.495	8.617	6

Frame: 1
Channel: Chemi
Sensitivity: 100
Molecular Weight Analysis Regression Method : Point to Point

Lane 2 - MCF10A

#	Vol. (Int.)	Local Bg. Corr. Vol.	Area	Rf	Density	Local Bg. Corr. Den.	% band purity	% lane purity	Mol. Wt.
1	8,385,003	7,402,987	400	0.49	20,962	18,507	100	75.45	37.714

Lane 3 - Skbr3

#	Vol. (Int.)	Local Bg. Corr. Vol.	Area	Rf	Density	Local Bg. Corr. Den.	% band purity	% lane purity	Mol. Wt.
1	13,767,070	12,584,558	440	0.492	31,288	28,601	100	79.439	37.429

Lane 4 - MCF7

#	Vol. (Int.)	Local Bg. Corr. Vol.	Area	Rf	Density	Local Bg. Corr. Den.	% band purity	% lane purity	Mol. Wt.
1	11,261,703	10,217,864	429	0.494	26,251	23,817	100	76.282	37.143

Lane 5 - MDA-MB-468

#	Vol. (Int.)	Local Bg. Corr. Vol.	Area	Rf	Density	Local Bg. Corr. Den.	% band purity	% lane purity	Mol. Wt.
1	8,797,884	8,053,535	380	0.494	23,152	21,193	100	72.502	37.143

Lane 6 - T47D

#	Vol. (Int.)	Local Bg. Corr. Vol.	Area	Rf	Density	Local Bg. Corr. Den.	% band purity	% lane purity	Mol. Wt.
1	7,017,107	6,432,084	380	0.492	18,466	16,926	100	70.644	37.429

Lane 7 - BT-549

#	Vol. (Int.)	Local Bg. Corr. Vol.	Area	Rf	Density	Local Bg. Corr. Den.	% band purity	% lane purity	Mol. Wt.
1	9,490,367	8,793,052	429	0.492	22,122	20,496	100	77.084	37.429

Lane 8 - Hs578t

#	Vol. (Int.)	Local Bg. Corr. Vol.	Area	Rf	Density	Local Bg. Corr. Den.	% band purity	% lane purity	Mol. Wt.
1	9,225,735	8,278,202	390	0.49	23,655	21,226	100	75.319	37.714

Lane 9 - MDA-MB-436

#	Vol. (Int.)	Local Bg. Corr. Vol.	Area	Rf	Density	Local Bg. Corr. Den.	% band purity	% lane purity	Mol. Wt.
1	11,021,069	9,940,518	462	0.49	23,855	21,516	100	81.642	37.714

Lane 10 - MDA-MB-231

#	Vol. (Int.)	Local Bg. Corr. Vol.	Area	Rf	Density	Local Bg. Corr. Den.	% band purity	% lane purity	Mol. Wt.
1	8,326,511	7,812,233	615	0.481	13,539	12,702	100	83.639	38.917

iBright™ Image Analysis Report

28 January 2022

Figure S2A- VASH1

CHEMI_11092021_150448_348ms_10A_24H_TRANS_VASH1_ERP_RB

Date:	11-09-2021 03:04:48PM
Mode:	Chemi Blots
Notes:	FigureS2A- VASH1
Model:	FL1500
Instrument name:	2462619090234
Serial No:	2462619090234
Firmware version:	1.6.0
iBA version:	4.0.1
Image size:	676px X 540px
Image area:	
Optical Zoom:	2x
Digital Zoom:	1x
Focus level:	455
Resolution:	5 x 5
Exposure time:	348 ms
Exposure mode:	Normal

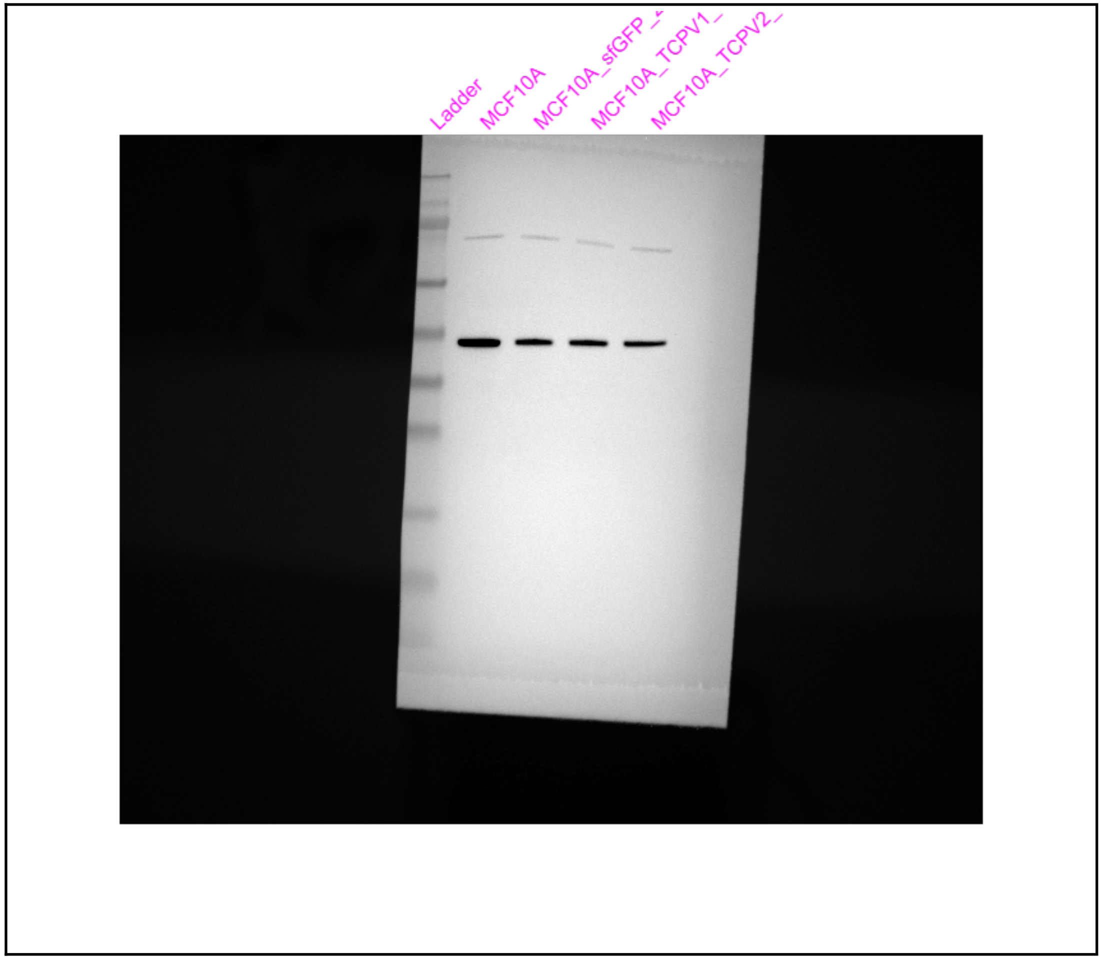

CHEMI_11092021_150448_348ms_10A_24H _TRANS_VASH1_ERP_RB

Date:	11-09-2021 03:04:48PM
Mode:	Chemi Blots
Notes:	FigureS2A- VASH1
Model:	FL1500
Instrument name:	2462619090234
Serial No:	2462619090234
Firmware version:	1.6.0
iBA version:	4.0.1
Image size:	676px X 540px
Image area:	
Optical Zoom:	2x
Digital Zoom:	1x
Focus level:	455
Resolution:	5 x 5
Exposure time:	348 ms
Exposure mode:	Normal

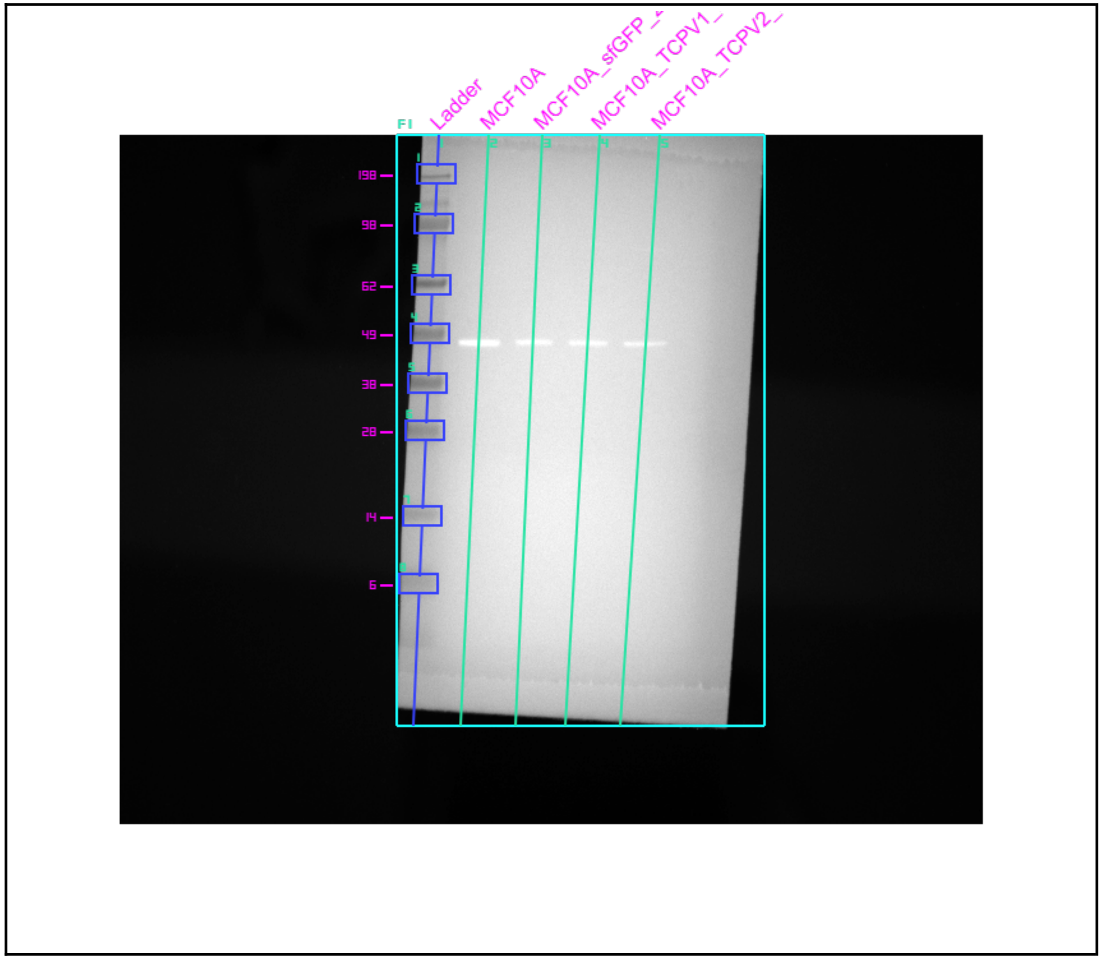

CHEMI_11092021_150448_348ms_10A_24H_TRANS_VASH1_ERP_RB

Date:	11-09-2021 03:04:48PM
Mode:	Chemi Blots
Notes:	FigureS2A- VASH1
Model:	FL1500
Instrument name:	2462619090234
Serial No:	2462619090234
Firmware version:	1.6.0
iBA version:	4.0.1
Image size:	676px X 540px
Image area:	
Optical Zoom:	2x
Digital Zoom:	1x
Focus level:	455
Resolution:	5 x 5
Exposure time:	348 ms
Exposure mode:	Normal

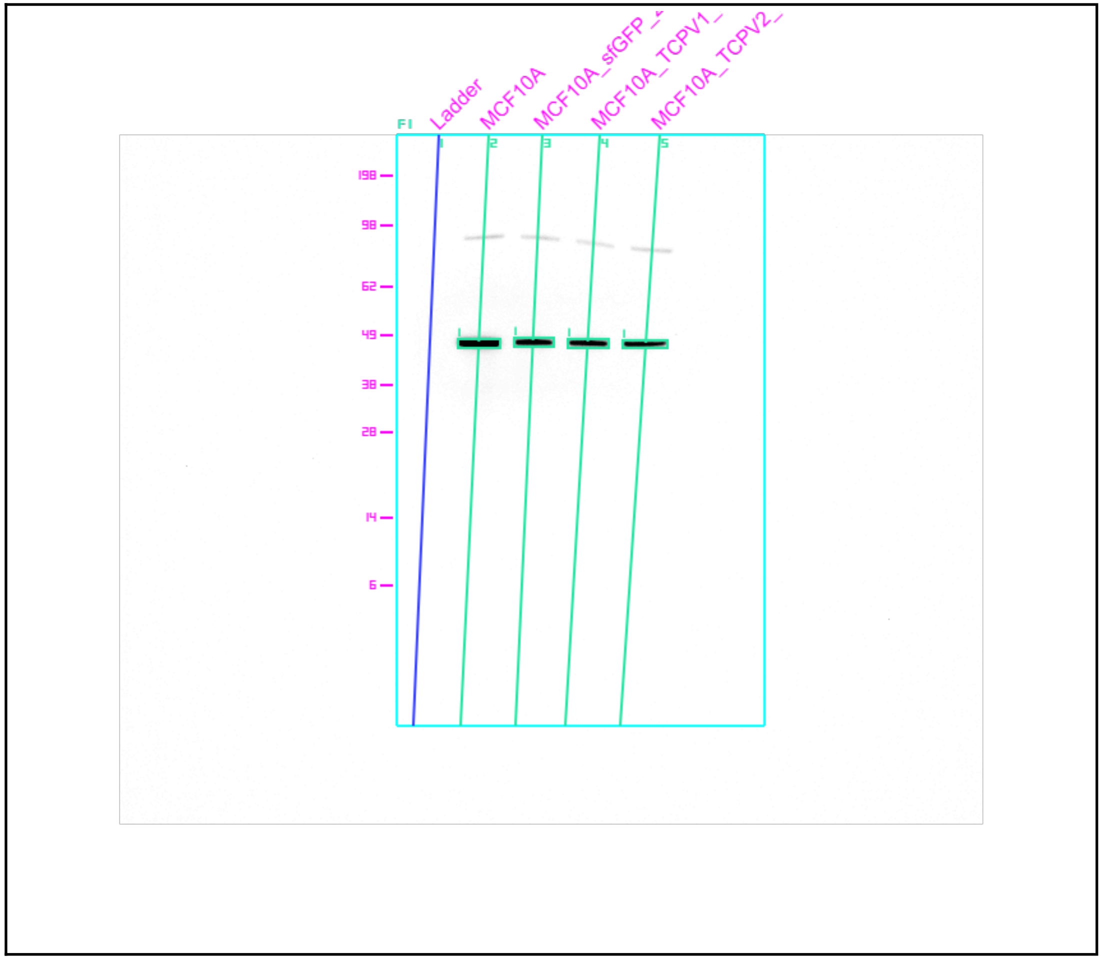

LANE AND BAND ANALYSIS DATA TABLE

CHEMI_11092021_150448_348ms_10A_24H_TRANS_VASH1_ERP_RB

Frame: 1
Channel: Membrane
Sensitivity: 100
Molecular Weight Analysis Regression Method : Point to Point

Lane 1 - Ladder

#	Vol. (Int.)	Local Bg. Corr. Vol.	Area	Rf	Density	Local Bg. Corr. Den.	% band purity	% lane purity	Mol. Wt.
1	18,507,993	1,015,882	496	0.065	37,314	2,048.15	15.627	3.855	198
2	19,474,923	1,216,851	496	0.149	39,263	2,453.33	18.719	4.056	98
3	18,974,809	1,405,055	496	0.253	38,255	2,832.773	21.614	3.952	62
4	18,538,003	967,576	496	0.335	37,375	1,950.76	14.884	3.861	49
5	18,413,680	986,285	496	0.419	37,124	1,988.479	15.172	3.835	38
6	17,646,771	525,105	496	0.499	35,578	1,058.681	8.078	3.676	28
7	15,693,158	34,069	496	0.644	31,639	68.688	0.524	3.269	14
8	16,874,536	349,876	496	0.758	34,021	705.395	5.382	3.515	6

Frame: 1
Channel: Chemi
Sensitivity: 100
Molecular Weight Analysis Regression Method : Point to Point

Lane 2 - MCF10A

#	Vol. (Int.)	Local Bg. Corr. Vol.	Area	Rf	Density	Local Bg. Corr. Den.	% band purity	% lane purity	Mol. Wt.
1	6,542,523	6,124,537	272	0.352	24,053	22,516	100	75.942	46.744

Lane 3 - MCF10A_sfGFP_24H

#	Vol. (Int.)	Local Bg. Corr. Vol.	Area	Rf	Density	Local Bg. Corr. Den.	% band purity	% lane purity	Mol. Wt.
1	2,620,902	2,462,881	256	0.35	10,237	9,620.631	100	60.607	47.026

Lane 4 - MCF10A_TCPV1_24H

#	Vol. (Int.)	Local Bg. Corr. Vol.	Area	Rf	Density	Local Bg. Corr. Den.	% band purity	% lane purity	Mol. Wt.
1	2,440,277	2,295,157	264	0.352	9,243.473	8,693.779	100	63.675	46.744

Lane 5 - MCF10A_TCPV2_24H

#	Vol. (Int.)	Local Bg. Corr. Vol.	Area	Rf	Density	Local Bg. Corr. Den.	% band purity	% lane purity	Mol. Wt.
1	2,265,298	2,154,029	252	0.354	8,989.278	8,547.736	100	66.929	46.462

iBright™ Image Analysis Report

28 January 2022

Figure S2B- VASH2

CHEMI_11092021_145546_421ms_10A_24H_TRANS_VASH2_EMD

Date:	11-09-2021 02:55:46PM
Mode:	Chemi Blots
Notes:	Figure S2B- VASH2
Model:	FL1500
Instrument name:	2462619090234
Serial No:	2462619090234
Firmware version:	1.6.0
iBA version:	4.0.1
Image size:	676px X 540px
Image area:	
Optical Zoom:	2x
Digital Zoom:	1x
Focus level:	455
Resolution:	5 x 5
Exposure time:	421 ms
Exposure mode:	Normal

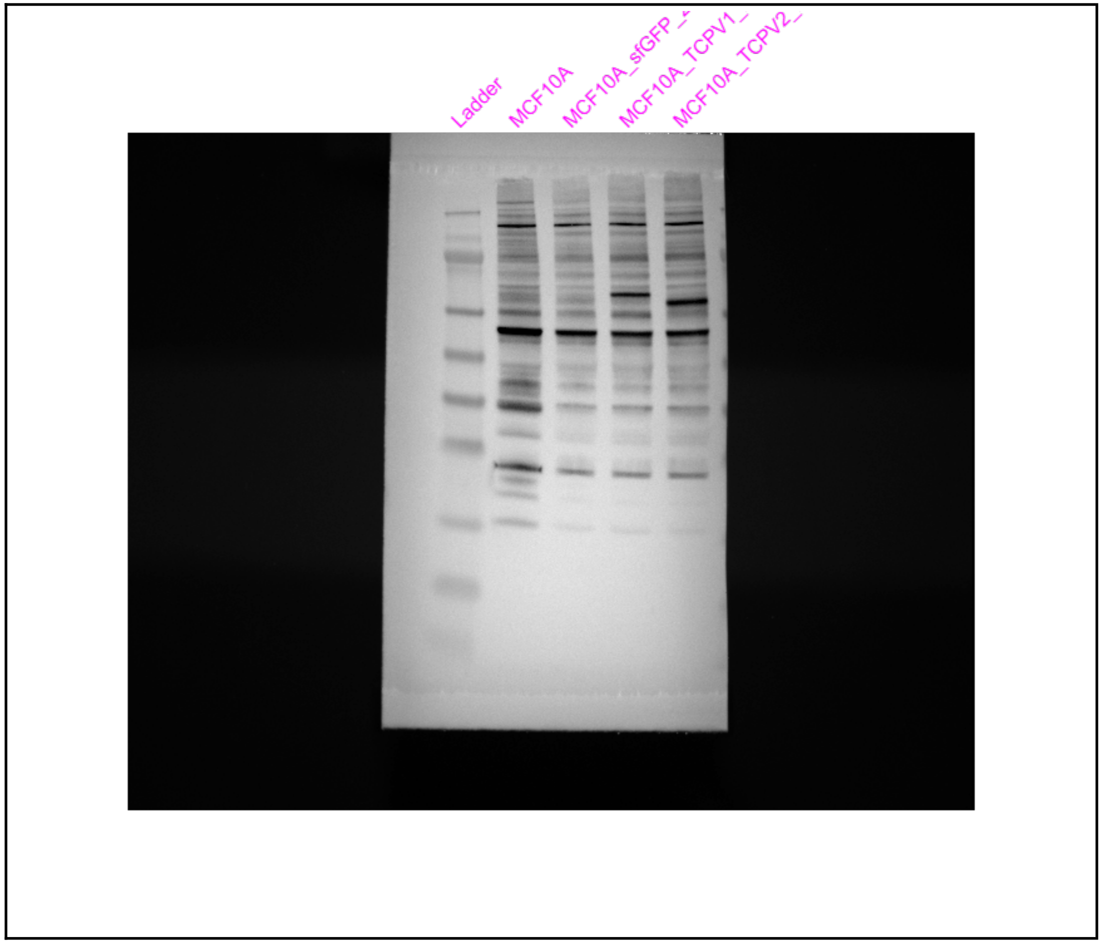

CHEMI_11092021_145546_421ms_10A_24H _TRANS_VASH2_EMD

Date:	11-09-2021 02:55:46PM
Mode:	Chemi Blots
Notes:	Figure S2B- VASH2
Model:	FL1500
Instrument name:	2462619090234
Serial No:	2462619090234
Firmware version:	1.6.0
iBA version:	4.0.1
Image size:	676px X 540px
Image area:	
Optical Zoom:	2x
Digital Zoom:	1x
Focus level:	455
Resolution:	5 x 5
Exposure time:	421 ms
Exposure mode:	Normal

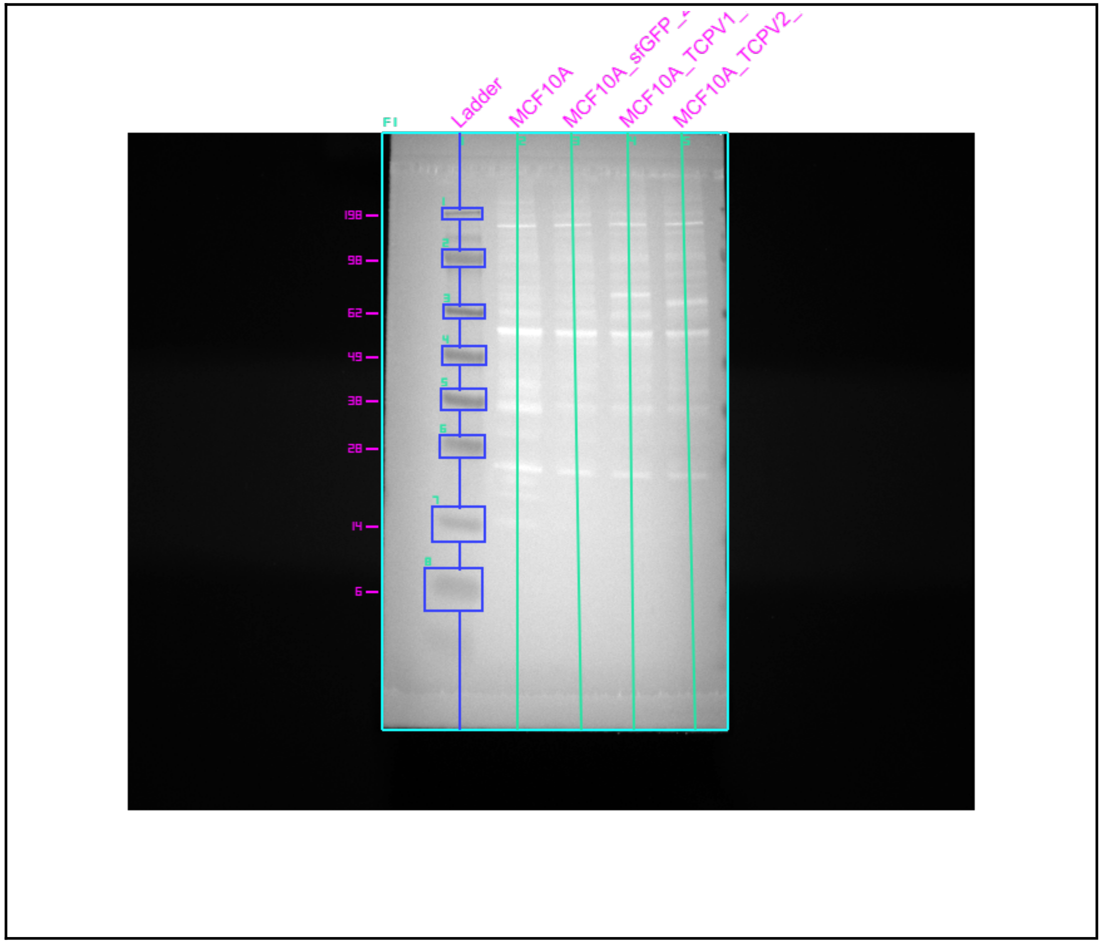

CHEMI_11092021_145546_421ms_10A_24H_TRANS_VASH2_EMD

Date:	11-09-2021 02:55:46PM
Mode:	Chemi Blots
Notes:	Figure S2B- VASH2
Model:	FL1500
Instrument name:	2462619090234
Serial No:	2462619090234
Firmware version:	1.6.0
iBA version:	4.0.1
Image size:	676px X 540px
Image area:	
Optical Zoom:	2x
Digital Zoom:	1x
Focus level:	455
Resolution:	5 x 5
Exposure time:	421 ms
Exposure mode:	Normal

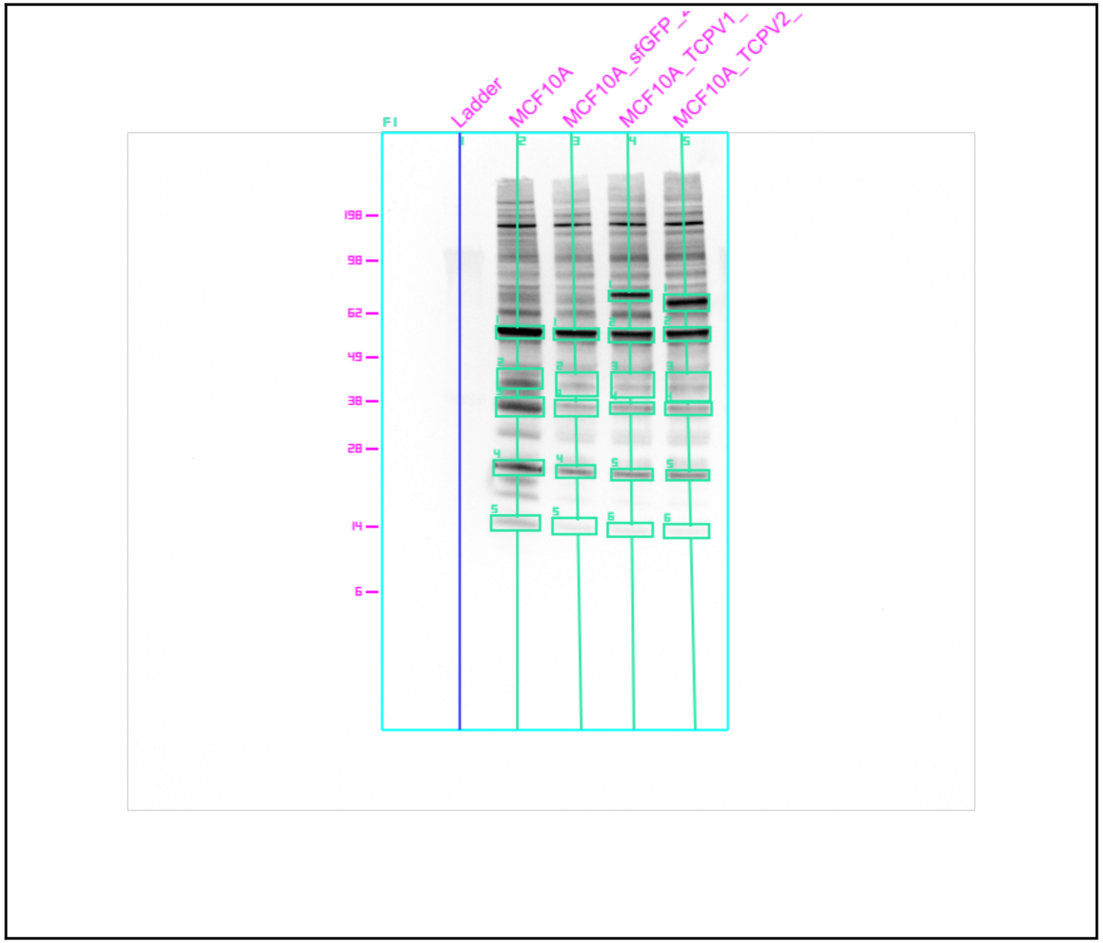

LANE AND BAND ANALYSIS DATA TABLE

CHEMI_11092021_145546_421ms_10A_24H_TRANS_VASH2_EMD

Frame: 1
Channel: Membrane
Sensitivity: 100
Molecular Weight Analysis Regression Method : Point to Point

Lane 1 - Ladder

#	Vol. (Int.)	Local Bg. Corr. Vol.	Area	Rf	Density	Local Bg. Corr. Den.	% band purity	% lane purity	Mol. Wt.
1	10,049,840	801,551	330	0.134	30,454	2,428.944	3.747	2.249	198
2	16,631,647	2,195,640	525	0.21	31,679	4,182.172	10.265	3.722	98
3	12,988,090	2,445,141	408	0.298	31,833	5,992.994	11.431	2.907	62
4	17,691,223	3,270,916	576	0.372	30,713	5,678.674	15.292	3.959	49
5	20,103,801	3,714,721	666	0.445	30,185	5,577.66	17.367	4.499	38
6	20,792,161	3,347,315	703	0.525	29,576	4,761.473	15.649	4.653	28
7	32,317,732	2,367,322	1,247	0.655	25,916	1,898.414	11.067	7.233	14
8	45,264,751	3,247,277	1,645	0.765	27,516	1,974.029	15.181	10.13	6

Frame: 1
Channel: Chemi
Sensitivity: 100
Molecular Weight Analysis Regression Method : Point to Point

Lane 2 - MCF10A

#	Vol. (Int.)	Local Bg. Corr. Vol.	Area	Rf	Density	Local Bg. Corr. Den.	% band purity	% lane purity	Mol. Wt.
1	12,167,387	8,570,716	429	0.334	28,362	19,978	35.321	11.42	55.686
2	8,624,693	4,003,699	629	0.412	13,711	6,365.182	16.5	8.095	43.029
3	9,024,372	5,764,671	624	0.458	14,462	9,238.256	23.757	8.47	36.421
4	6,854,914	4,323,561	533	0.561	12,861	8,111.748	17.818	6.434	24.161
5	2,222,107	1,602,907	520	0.653	4,273.283	3,082.514	6.606	2.086	14.226

Lane 3 - MCF10A_sfGFP_24H

#	Vol. (Int.)	Local Bg. Corr. Vol.	Area	Rf	Density	Local Bg. Corr. Den.	% band purity	% lane purity	Mol. Wt.
1	8,109,576	5,592,661	370	0.336	21,917	15,115	48.384	11.732	55.314

#	Vol. (Int.)	Local Bg. Corr. Vol.	Area	Rf	Density	Local Bg. Corr. Den.	% band purity	% lane purity	Mol. Wt.
2	4,296,882	1,622,442	680	0.42	6,318.944	2,385.945	14.036	6.216	41.771
3	3,098,479	1,851,150	490	0.46	6,323.427	3,777.857	16.015	4.483	36.158
4	2,841,771	2,016,445	352	0.567	8,073.213	5,728.538	17.445	4.111	23.484
5	767,555	476,259	504	0.658	1,522.927	944.96	4.12	1.11	13.846

Lane 4 - MCF10A_TCPV1_24H

#	Vol. (Int.)	Local Bg. Corr. Vol.	Area	Rf	Density	Local Bg. Corr. Den.	% band purity	% lane purity	Mol. Wt.
1	6,289,764	3,821,024	315	0.273	19,967	12,130	23.482	7.888	72.286
2	8,930,363	6,050,482	444	0.338	20,113	13,627	37.183	11.199	54.943
3	4,796,111	1,925,141	735	0.422	6,525.321	2,619.24	11.831	6.014	41.457
4	2,862,145	1,931,706	360	0.46	7,950.403	5,365.852	11.871	3.589	36.158
5	2,682,351	2,066,785	340	0.571	7,889.268	6,078.78	12.701	3.364	23.032
6	677,217	476,847	444	0.664	1,525.264	1,073.98	2.93	0.849	13.385

Lane 5 - MCF10A_TCPV2_24H

#	Vol. (Int.)	Local Bg. Corr. Vol.	Area	Rf	Density	Local Bg. Corr. Den.	% band purity	% lane purity	Mol. Wt.
1	10,359,253	6,482,622	518	0.284	19,998	12,514	33.909	12.346	68
2	9,046,849	6,165,040	456	0.336	19,839	13,519	32.248	10.782	55.314
3	5,539,939	1,923,495	864	0.424	6,411.966	2,226.268	10.061	6.602	41.143
4	3,176,445	2,118,216	418	0.462	7,599.151	5,067.504	11.08	3.786	35.895
5	2,653,470	2,071,438	315	0.574	8,423.714	6,575.996	10.835	3.162	22.806
6	510,975	356,781	444	0.666	1,150.845	803.562	1.866	0.609	13.231

iBright™ Image Analysis Report

28 January 2022

Figure S2C- SVBP SHORT Exposure

CHEMI_11092021_151004_660ms_10A_24H _TRANS_SVBP_INV

Date:	11-09-2021 03:10:04PM
Mode:	Chemi Blots
Notes:	FigureS2C- SVBP short
Model:	FL1500
Instrument name:	2462619090234
Serial No:	2462619090234
Firmware version:	1.6.0
iBA version:	4.0.1
Image size:	676px X 540px
Image area:	
Optical Zoom:	2x
Digital Zoom:	1x
Focus level:	455
Resolution:	5 x 5
Exposure time:	660 ms
Exposure mode:	Normal

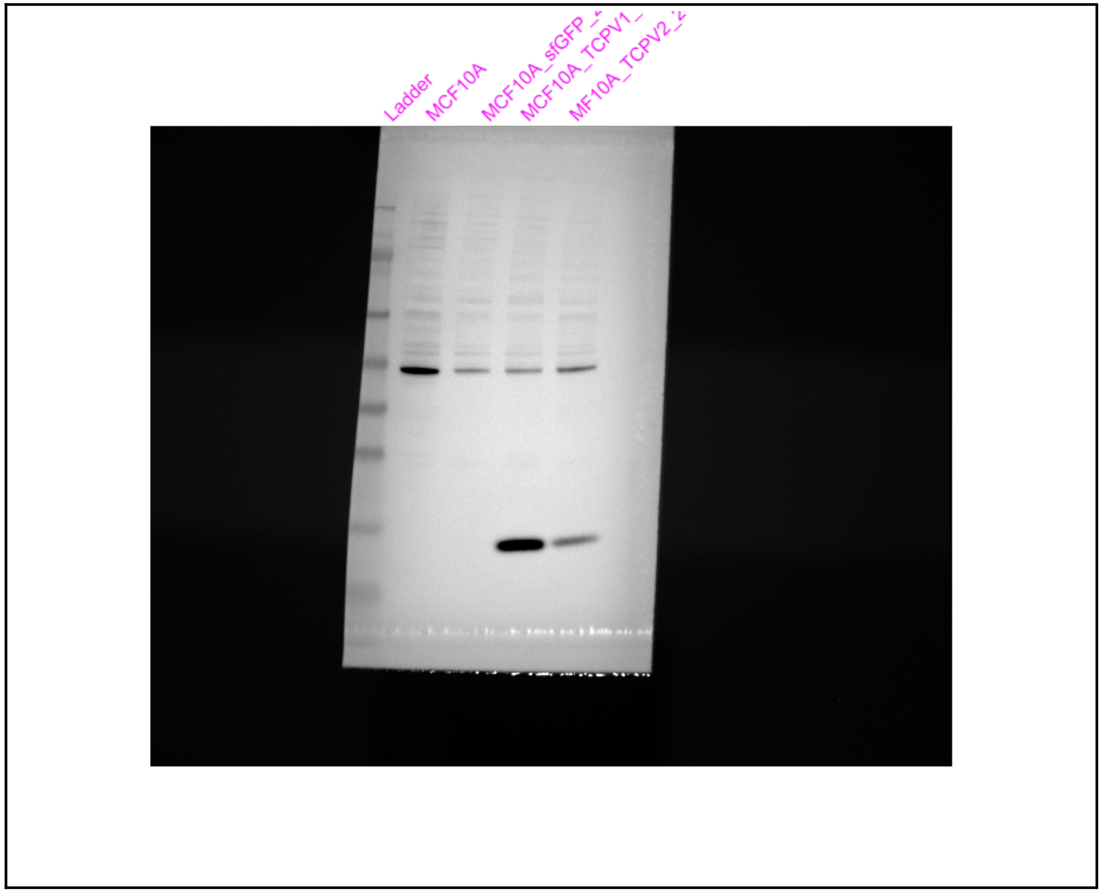

CHEMI_11092021_151004_660ms_10A_24H _TRANS_SVBP_INV

Date:	11-09-2021 03:10:04PM
Mode:	Chemi Blots
Notes:	FigureS2C- SVBP short
Model:	FL1500
Instrument name:	2462619090234
Serial No:	2462619090234
Firmware version:	1.6.0
iBA version:	4.0.1
Image size:	676px X 540px
Image area:	
Optical Zoom:	2x
Digital Zoom:	1x
Focus level:	455
Resolution:	5 x 5
Exposure time:	660 ms
Exposure mode:	Normal

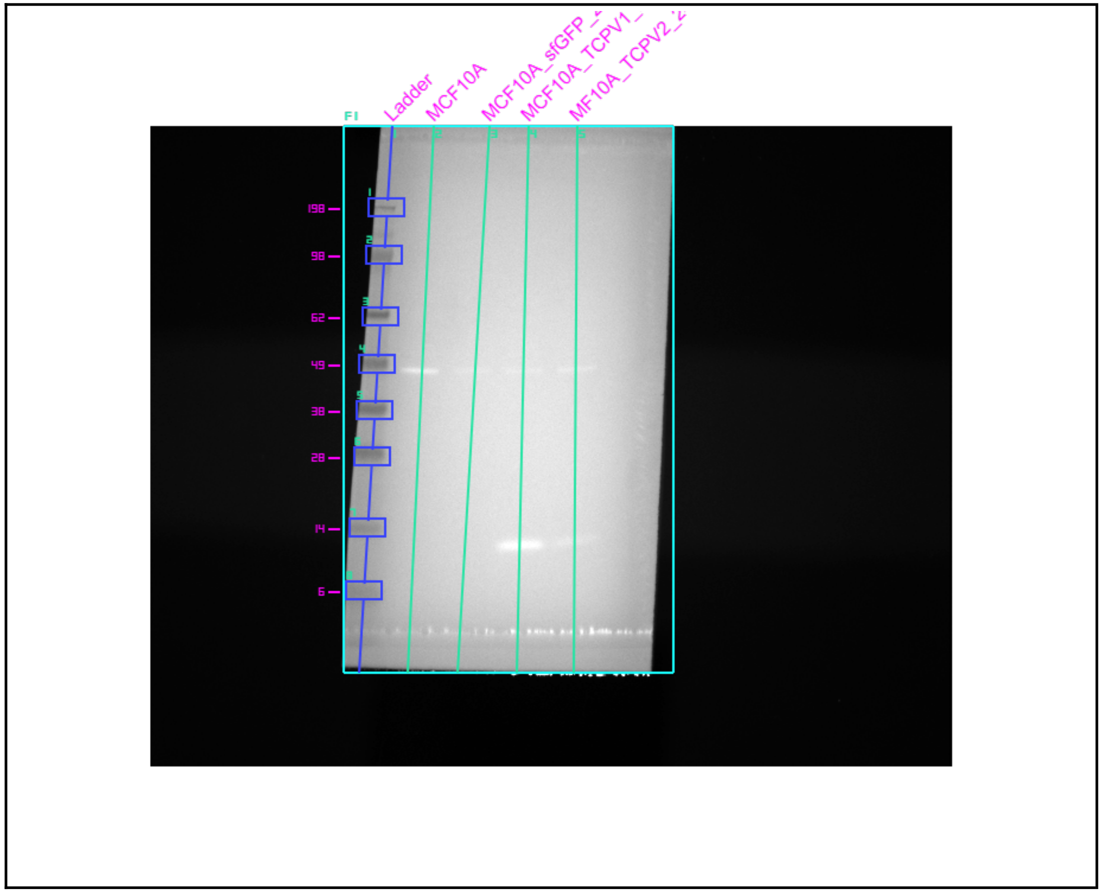

CHEMI_11092021_151004_660ms_10A_24H_TRANS_SVBP_INV

Date:	11-09-2021 03:10:04PM
Mode:	Chemi Blots
Notes:	FigureS2C- SVBP short
Model:	FL1500
Instrument name:	2462619090234
Serial No:	2462619090234
Firmware version:	1.6.0
iBA version:	4.0.1
Image size:	676px X 540px
Image area:	
Optical Zoom:	2x
Digital Zoom:	1x
Focus level:	455
Resolution:	5 x 5
Exposure time:	660 ms
Exposure mode:	Normal

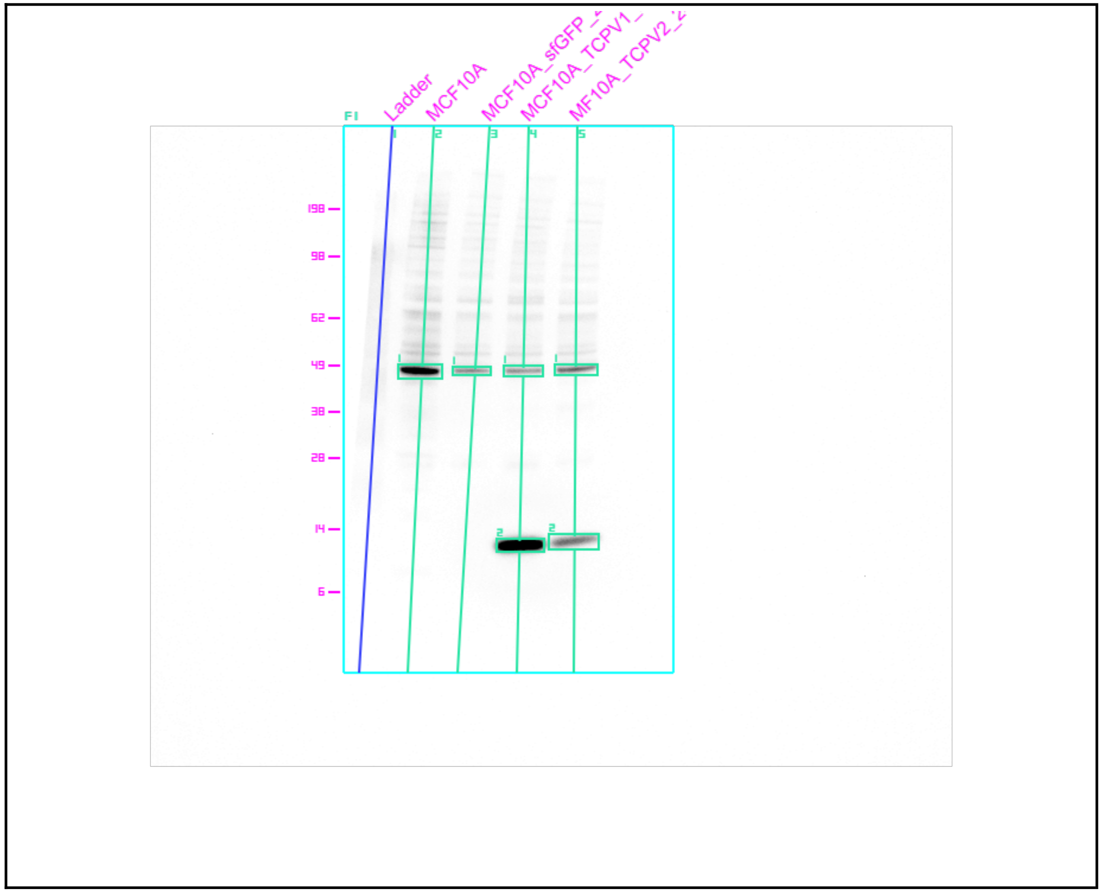

LANE AND BAND ANALYSIS DATA TABLE

CHEMI_11092021_151004_660ms_10A_24H_TRANS_SVBP_INV

Frame: 1
Channel: Membrane
Sensitivity: 100
Molecular Weight Analysis Regression Method : Point to Point

Lane 1 - Ladder

#	Vol. (Int.)	Local Bg. Corr. Vol.	Area	Rf	Density	Local Bg. Corr. Den.	% band purity	% lane purity	Mol. Wt.
1	19,373,935	1,626,578	496	0.148	39,060	3,279.391	23.095	3.497	198
2	19,493,800	1,421,774	496	0.234	39,302	2,866.48	20.187	3.518	98
3	18,891,708	1,325,275	496	0.347	38,088	2,671.927	18.817	3.41	62
4	19,037,136	1,175,601	496	0.434	38,381	2,370.164	16.692	3.436	49
5	18,640,488	932,025	496	0.518	37,581	1,879.083	13.233	3.364	38
6	17,967,669	515,858	496	0.603	36,225	1,040.036	7.324	3.243	28
7	17,125,122	35,758	496	0.733	34,526	72.093	0.508	3.091	14
8	17,760,137	10,071	496	0.848	35,806	20.305	0.143	3.206	6

Frame: 1
Channel: Chemi
Sensitivity: 100
Molecular Weight Analysis Regression Method : Point to Point

Lane 2 - MCF10A

#	Vol. (Int.)	Local Bg. Corr. Vol.	Area	Rf	Density	Local Bg. Corr. Den.	% band purity	% lane purity	Mol. Wt.
1	7,614,275	6,679,924	494	0.449	15,413	13,522	100	37.242	47.026

Lane 3 - MCF10A_sfGFP_24H

#	Vol. (Int.)	Local Bg. Corr. Vol.	Area	Rf	Density	Local Bg. Corr. Den.	% band purity	% lane purity	Mol. Wt.
1	1,542,093	1,254,200	264	0.447	5,841.261	4,750.758	100	17.692	47.308

Lane 4 - MCF10A_TCPV1_24H

#	Vol. (Int.)	Local Bg. Corr. Vol.	Area	Rf	Density	Local Bg. Corr. Den.	% band purity	% lane purity	Mol. Wt.
1	1,697,665	1,392,426	340	0.447	4,993.132	4,095.373	10.545	7.437	47.308

#	Vol. (Int.)	Local Bg. Corr. Vol.	Area	Rf	Density	Local Bg. Corr. Den.	% band purity	% lane purity	Mol. Wt.
2	13,302,079	11,811,656	492	0.766	27,036	24,007	89.455	58.276	11.736

Lane 5 - MF10A_TCPV2_24H

#	Vol. (Int.)	Local Bg. Corr. Vol.	Area	Rf	Density	Local Bg. Corr. Den.	% band purity	% lane purity	Mol. Wt.
1	2,349,640	2,040,500	370	0.445	6,350.378	5,514.866	39.932	19.843	47.59
2	3,737,750	3,069,478	602	0.759	6,208.887	5,098.801	60.068	31.565	12.189

iBright™ Image Analysis Report

28 January 2022

Figure S2C- SVBP LONG Exposure

CHEMI_11092021_151102_30s_10A_24H_TRANS_SVBP_INV

Date:	11-09-2021 03:11:02PM
Mode:	Chemi Blots
Notes:	FigureS2D- SVBP long
Model:	FL1500
Instrument name:	2462619090234
Serial No:	2462619090234
Firmware version:	1.6.0
iBA version:	4.0.1
Image size:	676px X 540px
Image area:	
Optical Zoom:	2x
Digital Zoom:	1x
Focus level:	455
Resolution:	5 x 5
Exposure time:	30000 ms
Exposure mode:	Normal

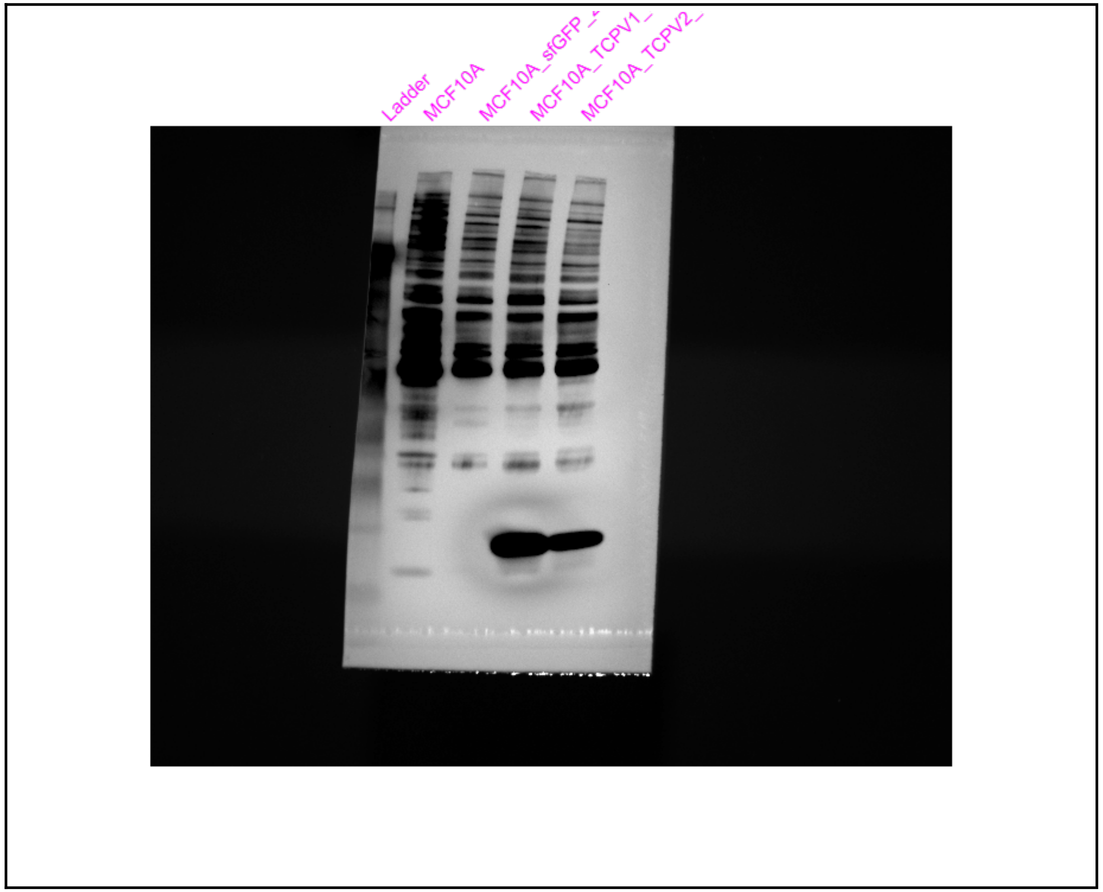

CHEMI_11092021_151102_30s_10A_24H_TRANS_SVBP_INV

Date:	11-09-2021 03:11:02PM
Mode:	Chemi Blots
Notes:	FigureS2D- SVBP long
Model:	FL1500
Instrument name:	2462619090234
Serial No:	2462619090234
Firmware version:	1.6.0
iBA version:	4.0.1
Image size:	676px X 540px
Image area:	
Optical Zoom:	2x
Digital Zoom:	1x
Focus level:	455
Resolution:	5 x 5
Exposure time:	30000 ms
Exposure mode:	Normal

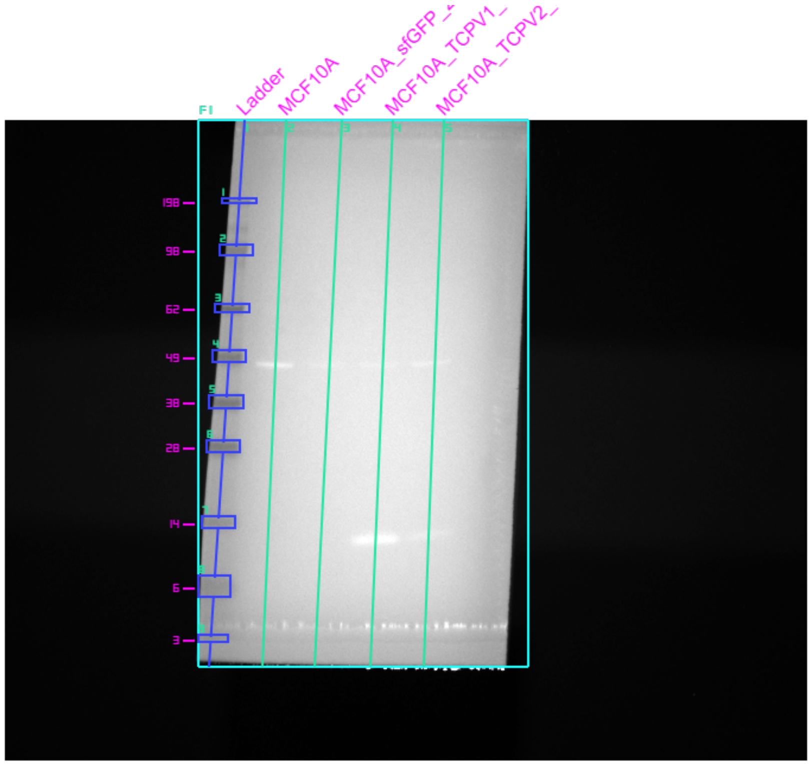

CHEMI_11092021_151102_30s_10A_24H_TRANS_SVBP_INV

Date:	11-09-2021 03:11:02PM
Mode:	Chemi Blots
Notes:	FigureS2D- SVBP long
Model:	FL1500
Instrument name:	2462619090234
Serial No:	2462619090234
Firmware version:	1.6.0
iBA version:	4.0.1
Image size:	676px X 540px
Image area:	
Optical Zoom:	2x
Digital Zoom:	1x
Focus level:	455
Resolution:	5 x 5
Exposure time:	30000 ms
Exposure mode:	Normal

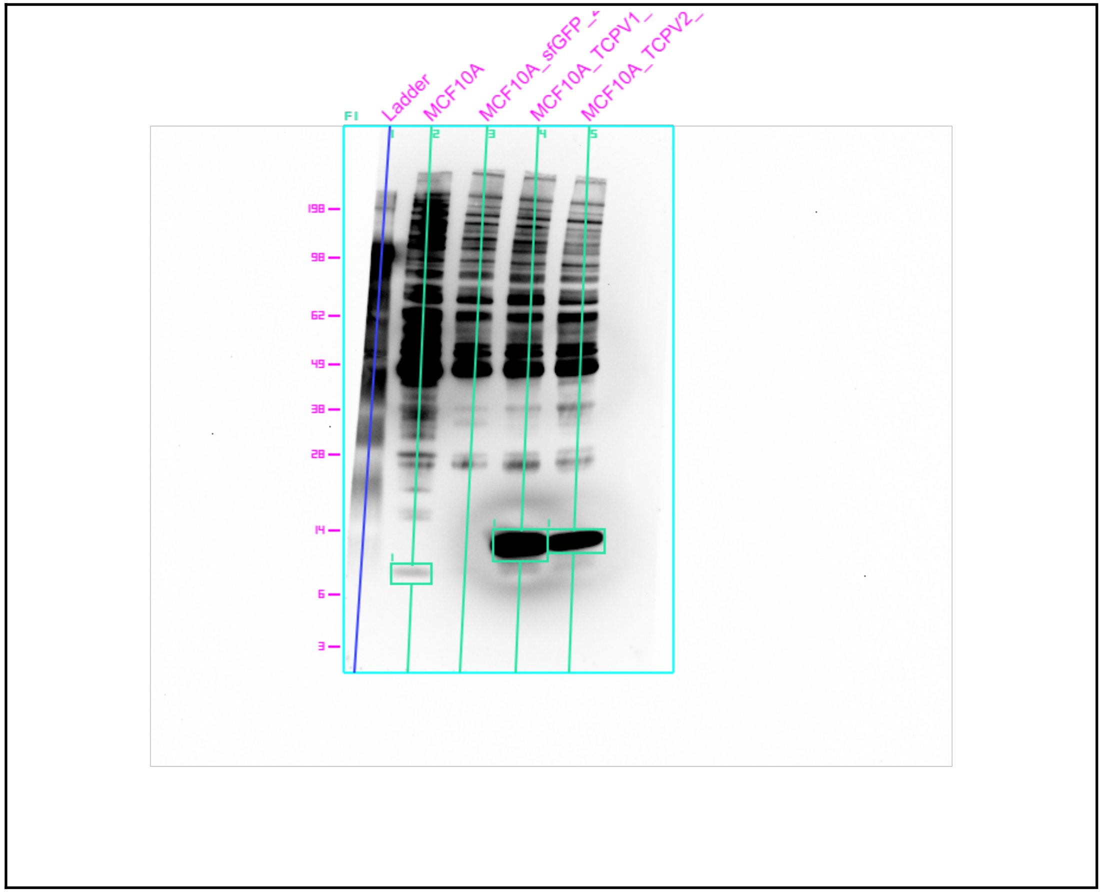

LANE AND BAND ANALYSIS DATA TABLE

CHEMI_11092021_151102_30s_10A_24H_TRANS_SVBP_INV

Frame: 1
Channel: Membrane
Sensitivity: 100
Molecular Weight Analysis Regression Method : Point to Point

Lane 1 - Ladder

#	Vol. (Int.)	Local Bg. Corr. Vol.	Area	Rf	Density	Local Bg. Corr. Den.	% band purity	% lane purity	Mol. Wt.
1	6,179,014	629,583	150	0.148	41,193	4,197.225	10.085	1.224	198
2	12,002,252	1,066,104	290	0.236	41,387	3,676.223	17.078	2.377	98
3	10,213,870	1,031,505	240	0.343	42,557	4,297.941	16.523	2.023	62
4	13,045,467	977,526	319	0.432	40,894	3,064.345	15.659	2.584	49
5	14,692,492	1,083,283	360	0.514	40,812	3,009.12	17.353	2.91	38
6	12,715,669	612,740	319	0.597	39,861	1,920.815	9.815	2.518	28
7	11,573,277	246,236	319	0.735	36,279	771.902	3.944	2.292	14
8	19,882,206	573,526	532	0.852	37,372	1,078.057	9.187	3.938	6
9	6,611,868	22,167	182	0.948	36,328	121.797	0.355	1.309	3

Frame: 1
Channel: Chemi
Sensitivity: 100
Molecular Weight Analysis Regression Method : Point to Point

Lane 2 - MCF10A

#	Vol. (Int.)	Local Bg. Corr. Vol.	Area	Rf	Density	Local Bg. Corr. Den.	% band purity	% lane purity	Mol. Wt.
1	4,940,404	2,930,266	630	0.818	7,841.911	4,651.217	100	1.225	8.37

Lane 4 - MCF10A_TCPV1_24H

#	Vol. (Int.)	Local Bg. Corr. Vol.	Area	Rf	Density	Local Bg. Corr. Den.	% band purity	% lane purity	Mol. Wt.
1	71,963,908	40,052,923	1,316	0.766	54,683	30,435	100	20.831	11.926

Lane 5 - MCF10A_TCPV2_24H

#	Vol. (Int.)	Local Bg. Corr. Vol.	Area	Rf	Density	Local Bg. Corr. Den.	% band purity	% lane purity	Mol. Wt.

#	Vol. (Int.)	Local Bg. Corr. Vol.	Area	Rf	Density	Local Bg. Corr. Den.	% band purity	% lane purity	Mol. Wt.
1	49,614,879	31,893,744	1,029	0.759	48,216	30,994	100	18.068	12.37
