# Supplementary material for: Tubulin Carboxypeptidase Activity Promotes Focal Gelatin Degradation in Breast Tumor Cells and Induces Apoptosis in Breast Epithelial Cells That Is Overcome by Oncogenic Signaling
Source: Cancers (Basel). 2022 Mar 28;14(7):1707. doi: 10.3390/cancers14071707 (PMC8996877; doi:10.3390/cancers14071707)
Supplement: Supplementary file 1 [file cancers-14-01707-s001.zip › cancers-1658953-suppl-3.22.pdf]

cancers

MDPI

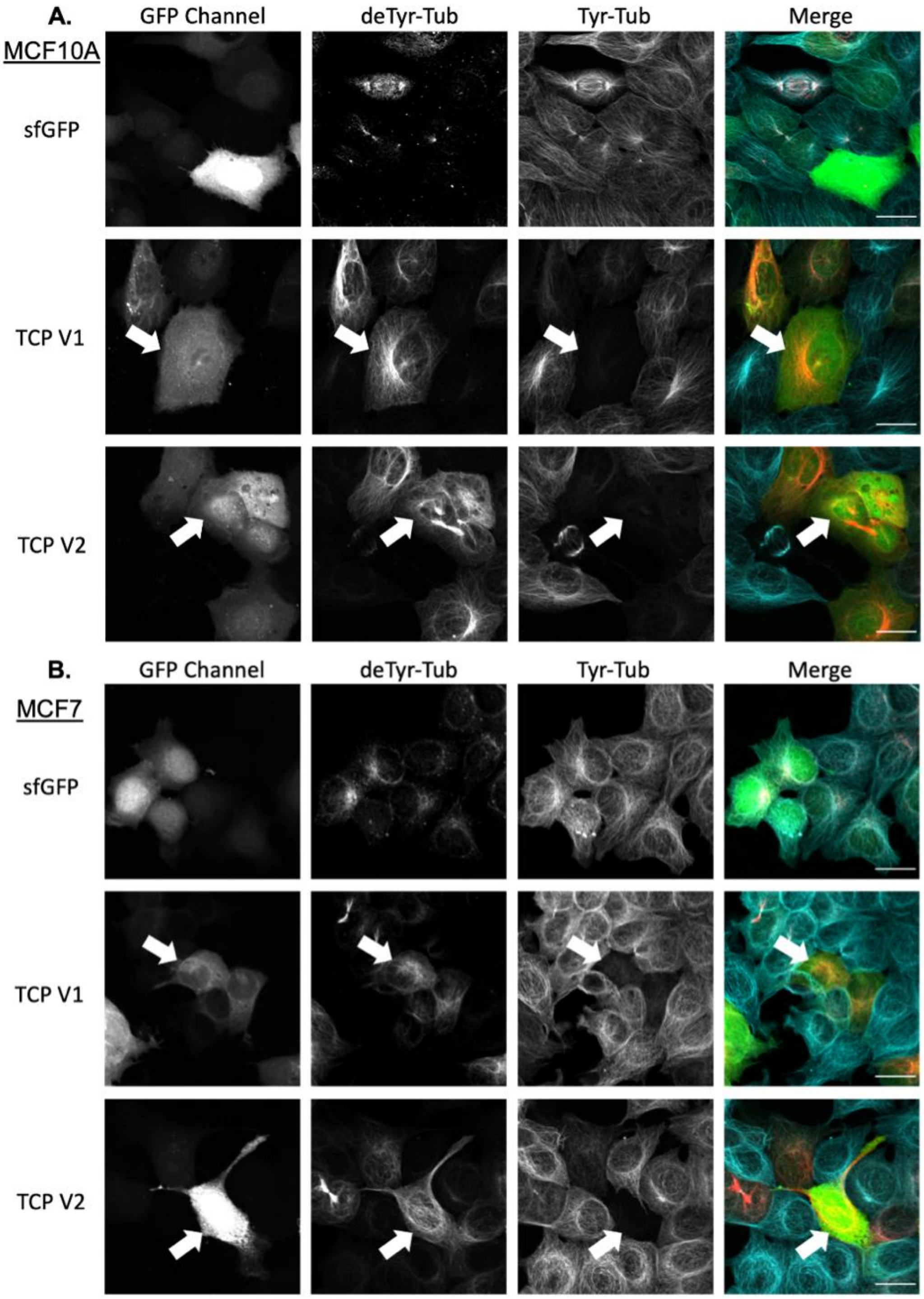

Supplemental Figure S1. TCP constructs effectively convert Tyr-Tub to deTyr-Tub. A. MCF10A cells were transfected with sfGFP, TCPV1 or TCPV2 constructs for 24 hours prior to fixation and staining for Tyr-Tub (cyan) or deTyr-Tub (red). Cells transfected with TCP constructs (green) show a pro-found increased in deTyr-Tub and decrease in Tyr-Tub. B. MCF7 cells transfected with the same constructs as in panel A show an increase in deTyr-Tub (red) and corresponding decrease in Tyr-Tub(cyan) only in cells transfected with TCP constructs (green). Scale bar = 20μm.

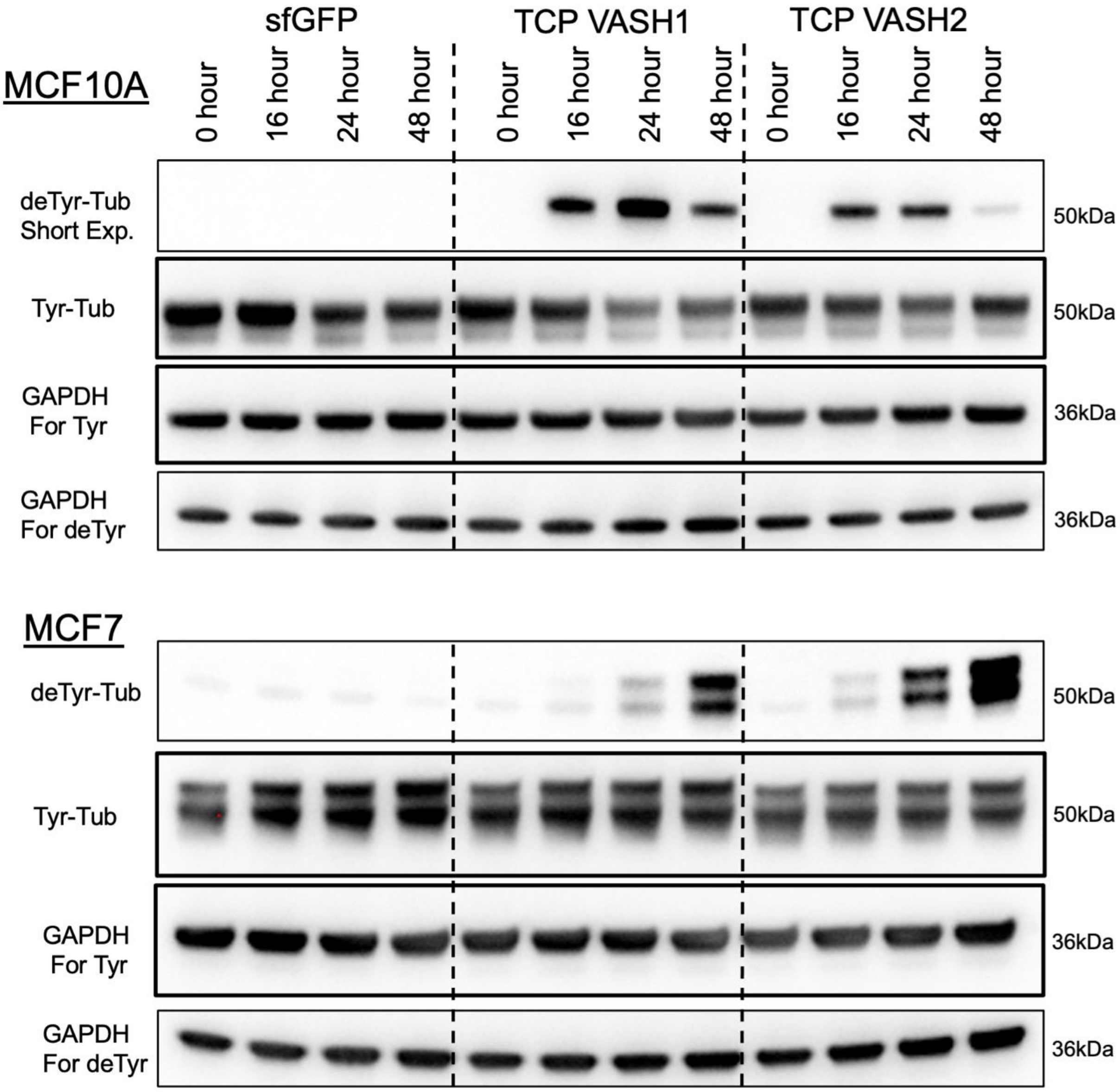

Supplemental Figure S2. *VASH1*/*SVBP* and *VASH2*/*SVBP* increase deTyr-Tub and decrease Tyr-Tub in a specific and time dependent manner. MCF10A and MCF7 cells were transfected with the same plasmid constructs as in Figure 1, *sfGFP*, *VASH1-sfGFP*-IRES-*SVBP*, or *VASH2-sfGFP*-IRES-*SVBP*, and lysed at times 0, 16h, 24h, and 48h post transfection. Primary antibodies against deTyr-Tub, Tyr-Tub, and GAPDH were incubated with HRP-conjugated secondary antibodies.

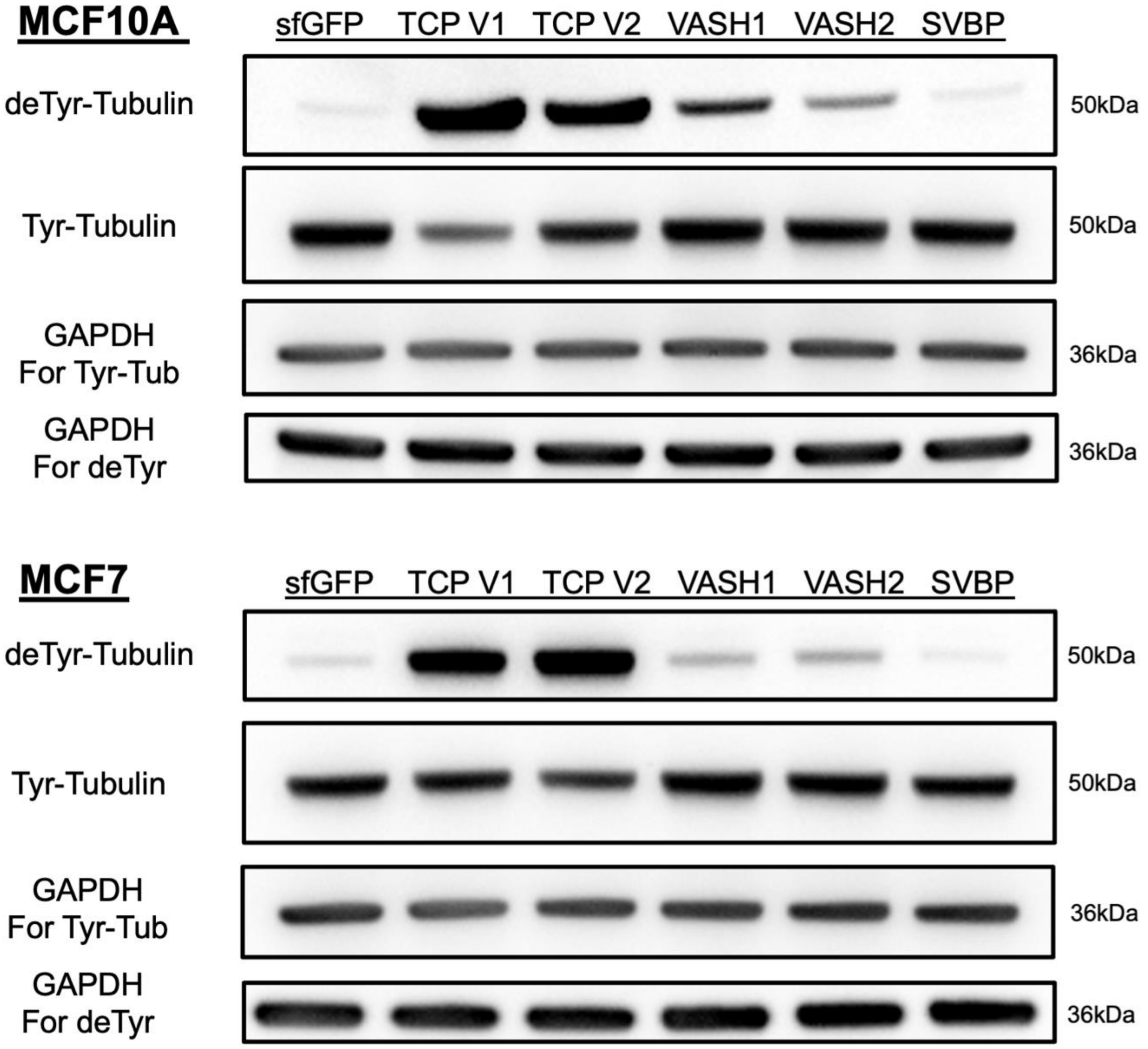

Supplemental Figure S3. *VASH1*/*SVBP* and *VASH2*/*SVBP* increase deTyr-Tub and decrease Tyr-Tub. MCF10A and MCF7 cells were transfected with the same plasmid constructs as in Figure 2, *sfGFP*, *VASH1-sfGFP*-IRES-*SVBP*, or *VASH2-sfGFP*-IRES-*SVBP*, *VASH1-sfGFP*, *VASH2-sfGFP*, and *SVBP* alone, and lysed at 24 hours post transfection. Primary antibodies against deTyr-Tub, Tyr-Tub, and GAPDH were incubated with HRP-conjugated secondary antibodies.

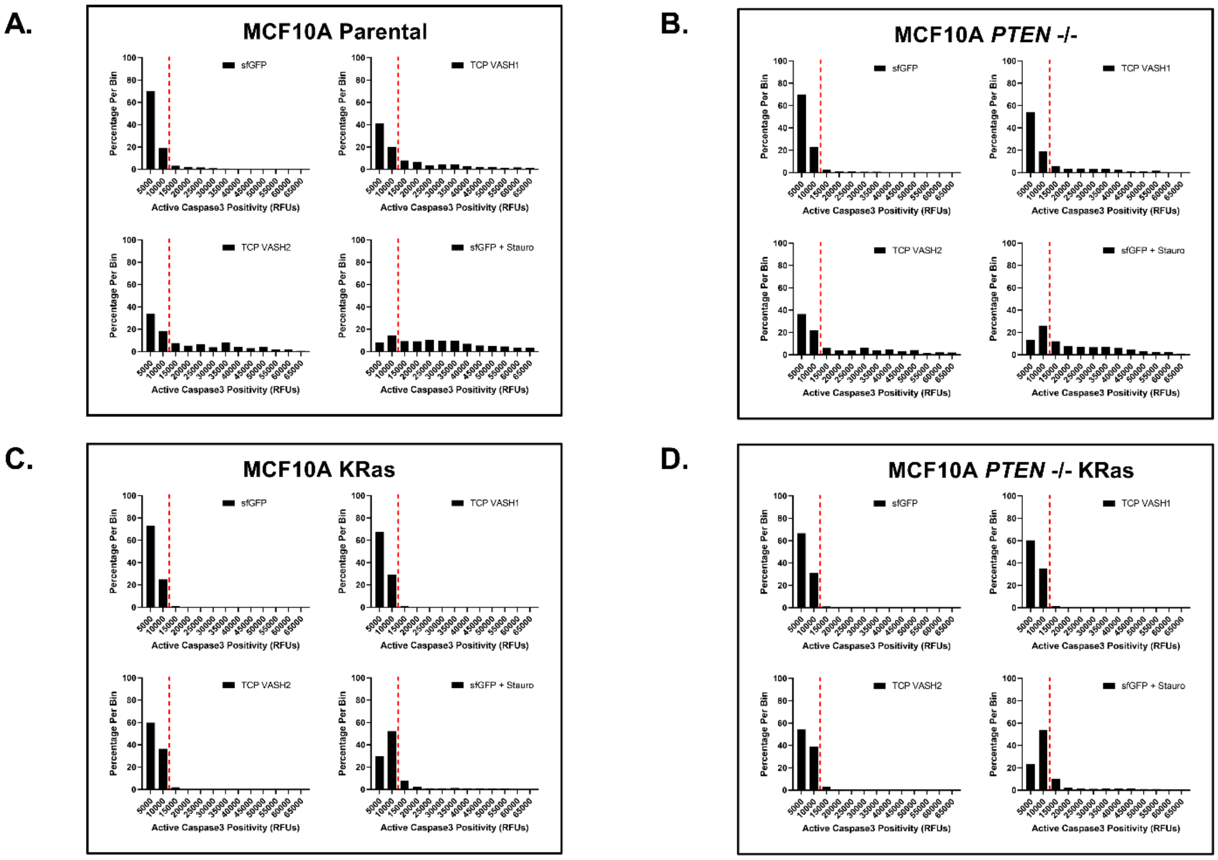

Supplemental Figure S4. Distributions of Active Caspase3 Intensities Across Transfected MCF10A Cell Lines. A distribution of active Caspase3 intensities for GFP positive transfected cells were plotted as histograms. MCF10A (A), MCF10A Pten-/- (B), MCF10A KRas (C), and MCF10A Pten-/- KRas (D) cell lines were transfected with sfGFP control, TCP VASH1, TCP VASH2, or sfGFP treated with 1μM staurosporin. The distribution of active Casapase3 intensities for GFP+ object intensities were plotted as the percentage of cells in the given bin across the range of pixel intensities. The red dashed line shows the threshold utilized to determine active Caspase3 for a given cell, with cells having less than 10,000 RFU being considered negative, and objects greater than 10,000 RFU considered positive.

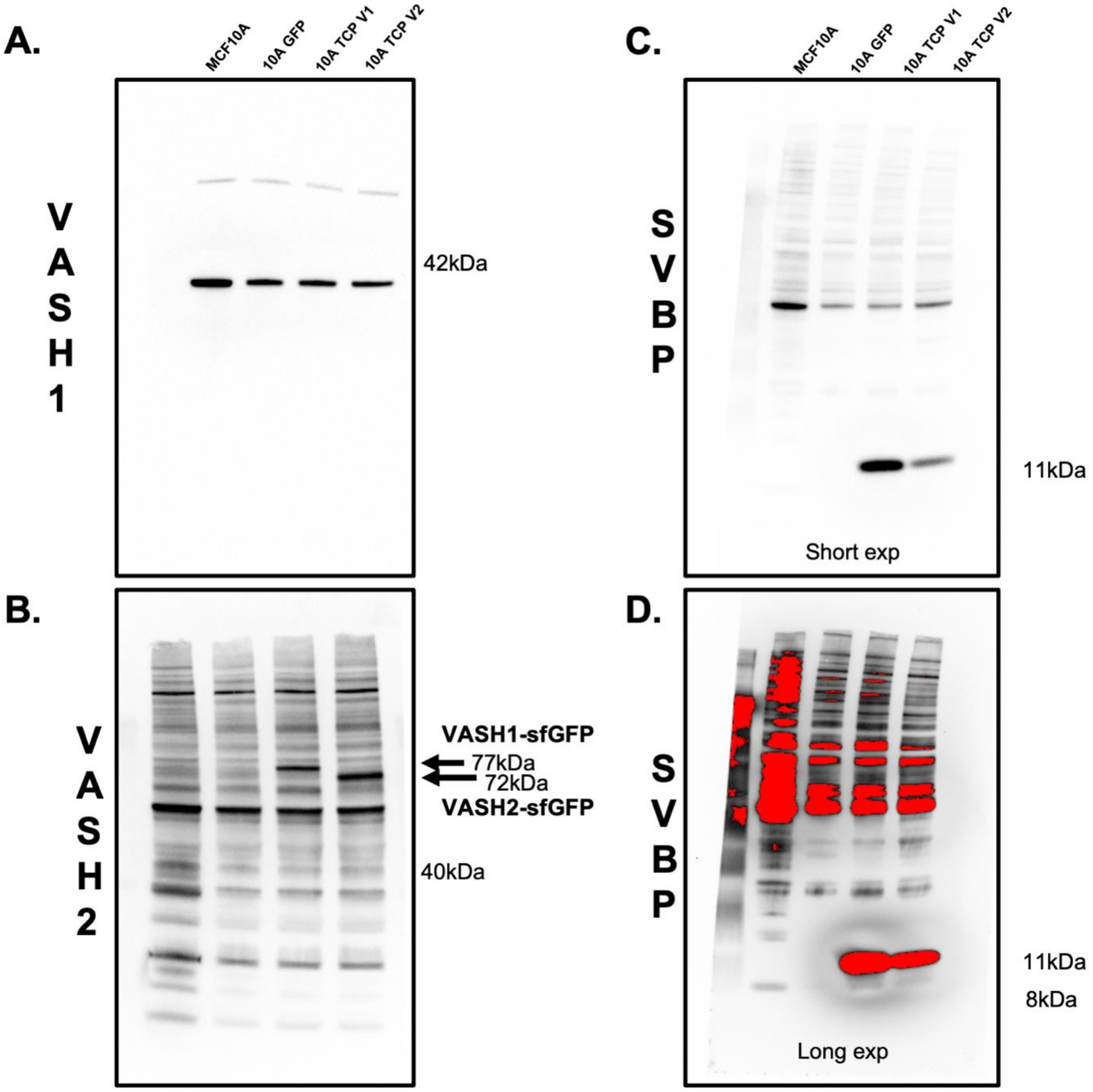

Supplemental Figure S5. Detection of TCP components by Western blot. Full image Western blots for the antibodies used to detect endogenous components of the TCP. Samples run were Lane 1- Ladder; Lane 2- Untransfected MCF10As; Lane 3- sfGFP Transfected MCF10As 24hrs post transfection; Lane 4- TCP VASH1 Transfected MCF10As 24hrs post transfec-tion; Lane 5- TCP VASH2 Transfected MCF10As 24hrs post transfection. **A**. The monoclonal anti-body against VASH1 detects a band at ~42kDa, but fails to detect the sfGFP-VASH1 fusion protein. **B**. The VASH2 antibody is able to detect both the sfGfP-VASH1 and sfGFP-VASH2 fusion proteins, as well as multiple bands around 40kDa where endogenous VASH1 and VASH2 should be located. **C**. The SVBP antibody detects a band at ~11kDa associated with the SVBP-myc expressed under the IRES. **D**. A longer exposure reveals a band at ~8kDa associated with the expected size of endogenous SVBP.

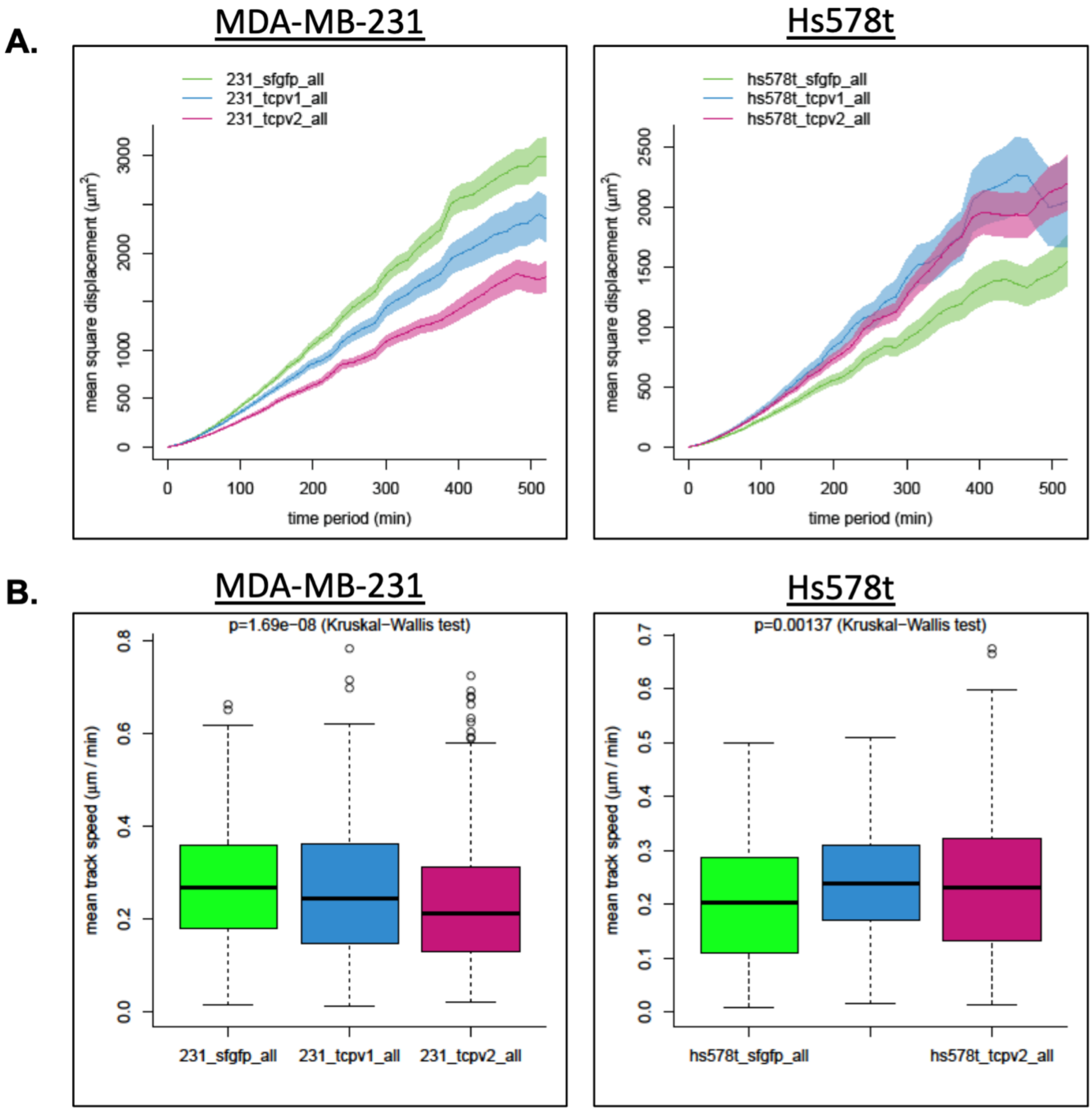

Supplemental Figure S6. The effect of TCP constructs on single-cell migration in MDA-MB-231 and Hs578t breast cancer cells. **A.** The mean squared displacement (MSD) was calculated for GFP+ MDA-MB-231 (n=3) or HS578t (n=2) cells transfected with *sfGFP*, *VASH1-sfGFP*-IRES-*SVBP*, or *VASH2-sfGFP*-IRES-*SVBP* constructs. The data show a decrease in MSD with the TCP constructs in MDA-MB-231s and an increase in MSD for Hs578ts. **B.** Mean track speed was calculated for MDA-MB-231 and Hs578ts from the same data set from panel A. These data revealed a decrease in speed for TCP transfected MDA-MB-231s and an increase in speed for TCP transfected Hs578ts. An aver-age of >100 cells were tracked per replicate.

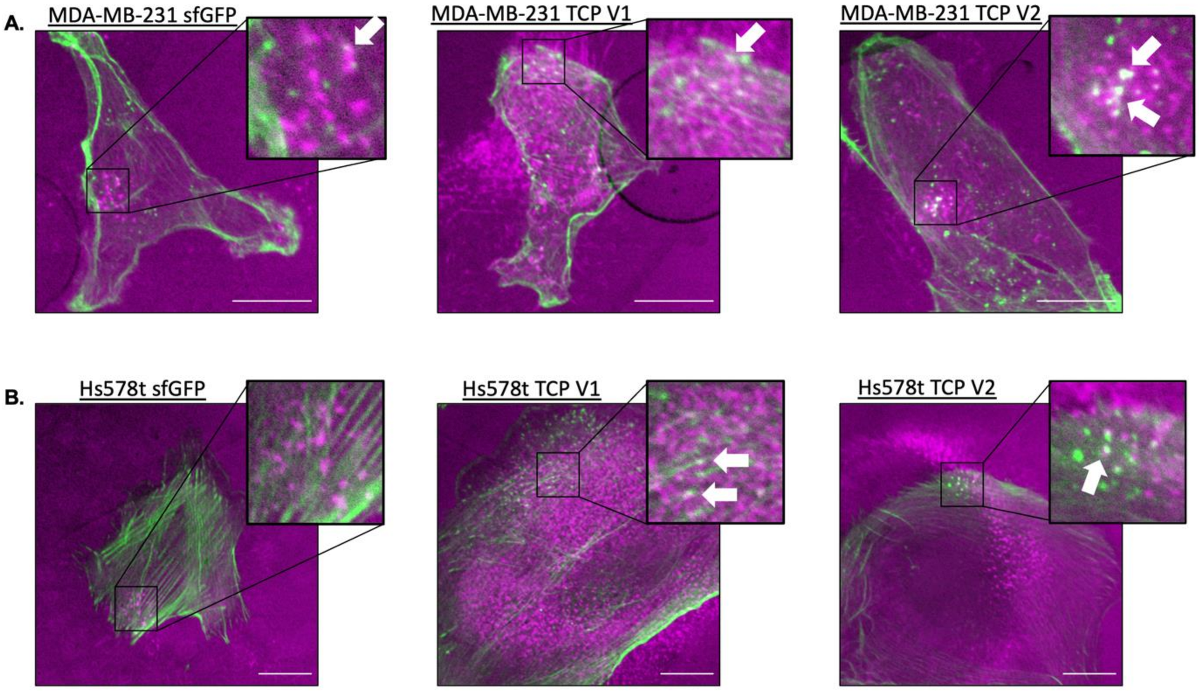

Supplemental Figure S7. Breast cancer cells in a gelatin degradation assay stained with phalloidin to show f-actin. **A.** MDA-MB-231 cells transfected with *sfGFP*, *VASH1*-*sfGFP*-IRES-*SVBP*, or *VASH2*-*sfGFP*-IRES-*SVBP* constructs were transfected and 24 hours later plated on gelatin for 24 hours. Cells were fixed, permeabilized, and stained for phalloidin to visualize f-actin (green). The inversion of gelatin image (magenta) shows bright areas where the gelatin has been degraded by the cell. When actin foci colocalizes with the absence of gelatin, white spots can be visualized (white arrow). **B.** Hs578t cells were transfected in the same manner as MDA-MB-231s in panel A, but allowed to degrade the gelatin for 48 hours prior to fixation, permeabilization, and staining with phalloidin for f-actin (green). Scale bar = 10μm.
